# An Immunosenescent CD8+ T Cell Subset in Patients with Axial Spondyloarthritis and Psoriatic Arthritis Links Spontaneous Motility to Telomere Shortening and Dysfunction

**DOI:** 10.1002/art.43109

**Published:** 2025-02-18

**Authors:** Giorgia Paldino, Valentina Tedeschi, Valentina Proganò, Erica Salvati, Valerio Licursi, Eleonora Vertecchi, Alexandru L. Bivolaru, Emanuele Molteni, Rossana Scrivo, Mattia Congia, Alberto Cauli, Rosalba Caccavale, Marino Paroli, Martina Kunkl, Loretta Tuosto, Rosa Sorrentino, Maria Teresa Fiorillo

**Affiliations:** ^1^ Sapienza University of Rome Rome Italy; ^2^ Institute of Molecular Biology and Pathology, National Research Council Rome Italy; ^3^ Azienda Ospedaliero‐Universitaria di Cagliari Cagliari Italy; ^4^ Sapienza University of Rome Polo Pontino Latina Italy; ^5^ Sapienza University of Rome and Scientific Institute for Research, Hospitalization and Healthcare Santa Lucia Foundation Rome Italy

## Abstract

**Objective:**

A pathogenetic role of CD8+ T lymphocytes in radiographic axial spondyloarthritis (r‐axSpA) and other spondyloarthritis (SpA) is sustained by genome‐wide association studies and by the expansion of public T cell clonotypes in the target tissues. This study investigates the migration of CD8+ T cells along with their phenotype and functions in patients with r‐axSpA and psoriatic arthritis (PsA).

**Methods:**

Peripheral blood CD8+ and CD4+ T cells were isolated from patients with r‐axSpA (n = 128), PsA (n = 60), and rheumatoid arthritis (RA) (n = 74) and healthy donors (HDs) (n = 79). Transwell migration assay was performed in the presence of different chemokines. CD8+ T cell immunoprofiling and effector functions were assessed by multiparametric flow cytometry. Transcriptome signature was evaluated by RNA sequencing analysis, whereas telomere length and dysfunction were measured by reverse transcriptase–polymerase chain reaction and immunofluorescence‐fluorescence in situ hybridization, respectively.

**Results:**

A significantly higher number of CD8+ T cells migrating in the absence of chemokine stimuli was found in patients with SpA compared with HDs and patients with RA. This subset, producing cytotoxic (granzyme B, perforin, granulysin) and proinflammatory molecules (tumor necrosis factor), was significantly enriched in terminally differentiated (CCR7−CD45RA+) and senescent (CD28−CD57+) cells having a gene expression profile characterized by cytolytic signature and natural killer markers. Remarkably, these spontaneously migrating CD8+ T cells showed DNA damage response activation, telomere shortening, and dysfunction.

**Conclusion:**

These data describe a terminally differentiated CD8+ T cell subset with a senescent and cytotoxic/proinflammatory profile and an intrinsic invasive potential enriched in patients with SpA that represents a possible player in disease pathogenesis.

## INTRODUCTION

Experimental animal models and clinical data strongly point at T lymphocytes as key players in the pathogenesis of radiographic axial spondyloarthritis (r‐axSpA), historically termed ankylosing spondylitis.^1^ R‐axSpA is the prototype of a group of chronic inflammatory rheumatic diseases, named spondyloarthritis (SpA), that share pathogenic mechanisms and clinical features.^2^, ^3^ These disorders also comprise psoriatic arthritis (PsA), arthritis associated with inflammatory bowel disease (IBD), reactive arthritis, and undifferentiated peripheral SpA.^1^, ^2^

R‐axSpA primarily involves sacroiliac joints and spine; however, inflammation at the peripheral joints and extra‐musculoskeletal manifestations (acute anterior uveitis, psoriasis, and IBD) may also occur.^4^ The strong association with the human leucocyte antigen (HLA)‐B*27 and the endoplasmic reticulum associated aminopeptidases 1 and 2 genes supports a guilty involvement of (auto)antigen presentation to CD8+ T cells.^5^, ^6^, ^7^ Additionally, other genes related to CD8+ T cell development and functions (*TBX21*, *EOMES*, *RUNX3*, and *ZMIZ1*) were associated with r‐axSpA susceptibility by genome‐wide association studies.^6^, ^8^ Recently, r‐axSpA–associated CD8+ T cell clonotypes with public T cell receptors (TRBV9+) and cross‐reactive to self and microbial HLA‐B*27–restricted antigens were found expanded in the synovial and ocular fluids of patients with SpA.^9^, ^10^ Importantly, selective antibody‐targeted depletion of these expanded TRBV9+ CD8+ T cells has been proven successful in r‐axSpA treatment.^11^

However, mechanisms and anatomic sites where these CD8+ T cell clonotypes are activated are currently unknown.^12^ It has been hypothesized that damage to dermal or mucosal barriers (as observed in psoriasis or IBD, respectively), along with the exposure of the immune system to microorganisms, may have a pathogenetic relevance.^13^ Accordingly, a gut‐joint axis of inflammation in SpA has been recently proposed; however, the CD8+ T cell migration profile and the chemokines involved are little‐known.^14^, ^15^

In addition, the physiological exposure during a lifetime to latent pathogens leads to persistent stimulation of CD8+ T cells that induces a premature immunosenescence.^16^, ^17^ Nevertheless, the contribution of this event as a possible cause or effect in rheumatic immune‐mediated inflammatory diseases remains unresolved.

In this study, we describe a subset of circulating CD8+ T cells enriched in patients with SpA compared with HDs and patients with rheumatoid arthritis (RA) with an intrinsic migratory capability independent of chemotactic stimuli. These cells exhibit an immunosenescent phenotype with telomere shortening, DNA damage response (DDR) activation, and telomere attrition, along with cytolytic and proinflammatory properties.

## MATERIALS AND METHODS

### Study participants

A total of 128 patients with r‐axSpA, 60 patients with PsA, 74 patients with RA, and 79 age‐matched healthy donors (HDs) were enrolled in this study (Table 1). Patients with r‐axSpA, PsA, and RA were classified according to standard criteria.^18^, ^19^, ^20^ Patients and controls were recruited at the Rheumatology Units of Sapienza University of Rome (Policlinico Umberto I, Roma, and ICOT Hospital, Latina) and the University of Cagliari (Azienda Ospedaliero‐Universitaria, Cagliari). HLA‐B*27 expression was checked both by serological analysis using ME1 monoclonal antibody (mAb) and by genomic analysis using Micro SSP Allele‐specific HLA class I DNA typing tray B*27 (ONE LAMBDA, Thermo Fisher) according to the manufacturer's instructions. The study received approval by the Ethics Committees of Sapienza University of Rome (0018614/2019 and 6893/2022) and the University of Cagliari (PG/2018/16312). All participants provided written informed consent before enrollment.

**Table 1:** Characteristics of patients with radiographic r‐axSpA, PsA, and RA, and HD enrolled in this study*

Patient characteristics	r‐axSpA (n = 128)	PsA (n = 60)	RA (n = 74)	HD (n = 79)	r‐axSpA vs HD, *P* value	PsA vs HD, *P* value	RA vs HD, *P* value
Age, mean ± SD, y	50 ± 14.1	55 ± 14.0	56 ± 13.6	51 ± 15.0	ns	ns	ns
Disease duration, mean ± SD, y	14.1 ± 12.1	10.3 ± 8.9	10.5 ± 7.7	na	—	—	—
Sex ratio (% of men)	72	53	24	49	0.0011	ns	0.0014
HLA‐B*27 (% positivity)	80	18	12	8	< 0.0001	ns	ns
BASDAI, mean ± SD	3 ± 2.4	na	na	na	—	—	—
ASDAS‐CRP, mean ± SD	2.1 ± 1.1	nd	na	na	—	—	—
DAS28‐CRP, mean ± SD	na	na	3 ± 1.3	na	—	—	—
DAPSA, mean ± SD	na	11 ± 9.9	na	na	—	—	—
CRP, mean ± SD, mg/l	5 ± 11.3	4 ± 7.9	2 ± 4.3	nd	—	—	—
ESR, mean ± SD, mm/h	18 ± 17.7	15 ± 14.1	21 ± 18.5	nd	—	—	—
bDMARDs, %	77	77	80	nd	—	—	—
NSAIDs, %	9	0	0	nd	—	—	—
csDMARDs, %	9	17	28	nd	—	—	—
None, %	10	7	4	nd	—	—	—
Glucocorticoids, %	2	5	8	nd	—	—	—
Other, %	2	2	3	nd	—	—	—

* *P* values were determined by Kruskal‐Wallis test, except for sex ratio and HLA‐B*27 positivity, where chi‐square test has been applied; *P* values < 0.05 were considered significant. ASDAS‐CRP, ankylosing spondylitis disease activity score with C‐reactive protein, determined in 104 out 128 patients with r‐axSpA; BASDAI, Bath Ankylosing Spondylitis Disease Activity Index; bDMARDs, biologic disease‐modifying antirheumatic drugs (anti‐TNFα, anti‐IL17, anti‐IL6 receptor agents); CRP, C‐reactive protein; csDMARDs, conventional synthetic disease‐modifying antirheumatic drugs; DAS28‐CRP, disease activity score in 28 joints with C‐reactive protein; DAPSA, disease activity in psoriatic arthritis; ESR, erythrocyte sedimentation rate; HD, healthy donor; HLA‐B*27, human leucocyte antigen; IL, interleukin; na, not applicable; nd, not determined; none, not in therapy; ns, not significant; other, beta‐blockers/angiotensin‐converting enzyme; inhibitors/antidiabetics (several patients take more drugs simultaneously); NSAIDs, nonsteroidal anti‐inflammatory drugs; PsA, psoriatic arthritis; RA, rheumatoid arthritis; r‐axSpA, radiographic axial spondyloarthritis; TNFα, tumor necrosis factor alpha.

### Peripheral blood mononuclear cells separation and T cells isolation

Peripheral blood mononuclear cells were isolated on density gradient Lympholyte solution (Cederlane Laboratories) from blood samples in sodium citrate within 24 hours from blood draw. CD8+ and CD4+ T lymphocytes were positively isolated by the respective isolation kits (Miltenyi Biotec). Each subset was resuspended at 10^6^ cells/mL density in Roswell Park Memorial Institute Medium (RPMI) (Euroclone) supplemented with 5% fetal bovine serum (Biochrom), glutamine 2 mM (Euroclone), amphotericin B 2.5 μg/mL (Euroclone), and penicillin/streptomycin 100 U/mL/100 μg/mL (Euroclone). All experiments were performed after overnight culture.

### Transwell migration assay

CD8+ or CD4+ T cells were resuspended at 1.5 × 10^5^ cells in chemotaxis buffer (CB) (0.5% bovine serum albumin [BSA], 25 mM 4‐(2‐hydroxyethyl)‐1‐piperazin ethanesulfonic acid in RPMI) at room temperature (RT) and subjected to migration using 96‐well transwell plates (Transwell, Corning) in the presence of the chemokine (CXCL9, CXCL10, and CXCL12 at 100 nM; CXCL11 and CCL20 at 300 nM) or in CB alone as control. Afterward, the plate was left at 37°C with 5% CO_2_ for 90 minutes. A further condition (Input) was used to normalize the number of migrated cells as follows: (1.5 × 10^5^ × number of migrated cells)/number of Input cells. Then, migrated cells, non‐migrated cells, and the Input were fixed in 1% weight/volume (w/v) paraformaldehyde/phosphate buffered saline (PBS) 1× and counted by FACSCalibur (Becton Dickinson) before the analysis by FlowJo software V.10.9.0 (Tree Star Inc).

### CD8+ T cell immunophenotype

The immunoprofile of migrated and non‐migrated CD8+ T cells was assessed after 20 seconds of incubation at 4°C with fluorochrome‐conjugated antibodies (Supplementary Table 1). Cells were then washed in PBS 1× and fixed in 2% w/v paraformaldehyde/PBS 1× for subsequent flow cytometry acquisition and analysis, as previously described.

### Proinflammatory and cytolytic molecules production

Migrated and non‐migrated cells were treated with brefeldin A (10 μg/mL) at 37°C for 16 hours. Cells were stained with anti‐CD3 (Supplementary Table 1) for 20 seconds on ice, fixed with 4% paraformaldehyde for 20 seconds on ice, and permeabilized with 1% BSA/0.1% saponin/PBS 1× for 5 seconds at RT. Finally, cells were stained by anti‐granzyme B, anti‐perforin, anti‐granulysin, and anti‐tumor necrosis factor alpha (TNFα) mAbs (Supplementary Table 1). Cells fixed in 2% w/v paraformaldehyde/PBS 1× were then acquired with FACSCalibur and analyzed as previously described.

### RNA isolation, RNA library construction, and RNA sequencing

RNA from the migrated and non‐migrated CD8+ T cells of eight patients with r‐axSpA was extracted by QIAGEN RNeasy Plus Micro Kit according to the manufacturer's instructions. Ovation SoLo RNA‐seq Library Preparation kit (Tecan Genomics) was used for library preparation following the manufacturer's instructions. After quantification, RNA quality was tested by Agilent 2100 Bioanalyzer RNA assay (Agilent technologies) or Caliper (PerkinElmer). Final libraries were checked with Qubit 2.0 Fluorometer (Invitrogen) and Agilent Bioanalyzer DNA assay or Caliper (PerkinElmer). The libraries were sequenced on paired‐end 150 bp mode on NovaSeq 6000 (Illumina). Detailed information about RNA sequencing is provided in the Supplementary Materials.

### Telomere length analysis

Genomic DNA from migrated and non‐migrated cells was extracted by QIAGEN QIAamp DNA Micro Kit following the manufacturer's instructions. DNA was quantified by Nanodrop (ThermoScientific) and used for telomere length analysis. The telomeric ends repeat and the single copy gene β‐globin were measured by quantitative reverse transcriptase–polymerase chain reaction (RT‐PCR). RT‐PCR details are reported in the Supplementary Materials.

### Fluorescence in situ hybridization and immunofluorescence

Migrated and non‐migrated CD8+ T cells were plated on poly‐L‐lysine coated coverslips. Cells were then fixed (2% formaldehyde), permeabilized (0.1% triton X‐100/PBS 1X), and blocked in 5% BSA/PBS 1×. Samples were incubated with an anti‐phosphohistone H2AX antibody (Supplementary Table 1) and then with anti‐mouse IgG Alexa fluor 488 conjugate secondary antibody (Cell Signaling). Samples were then refixed in 2% formaldehyde, dehydrated with ethanol series, air‐dried and co‐denaturated for three seconds at 80°C with a Cy3‐labeled peptide nucleic acid probe, telomere sequence specific (TelC‐Cy3, Panagene), and incubated for two hours in a humidified chamber at RT in the dark. After hybridization, coverslips were washed with 70% formamide, 10 mM Tris‐HCl pH 7.2, BSA 0.1%, and then in Tris buffered saline–Tween 0.08%, dehydrated with ethanol series, counterstained with 4',6‐diamidino‐2‐phenylindole (DAPI) (0.5 μg/mL, Sigma‐Aldrich), and mounted on specimen slides in mounting medium (Gelvatol Moviol, Sigma‐Aldrich). Fluorescence signals were acquired by a Nikon Crest Spinning disk at 60× magnitude. Z‐stacks were acquired at 0.6 μm steps and then processed with a Nikon imaging software. For lamin B1 detection, fixed and permeabilized cells were incubated with anti‐lamin B1 antibody (Supplementary Table 1) and then with an anti‐rabbit IgG Alexa fluor 555, counterstained with DAPI and mounted as previously described. Fluorescence signals were acquired as previously described. Nuclear circularity and area were calculated by ImageJ software.

### Statistical analysis

Differences among the cohorts were evaluated by the Kruskal‐Wallis test, with Dunn's correction for multiple comparisons, whereas comparison within the same group was done by Wilcoxon test. The comparison between the number of migrated CD8+ and CD4+ T lymphocytes, circularity index (CI), and nuclear area of non‐migrated versus migrated CD8+ T cells were performed by the Mann–Whitney test. Correlation between age and telomere length (T/S ratio) was evaluated by simple linear regression analysis. Statistical significance was accepted for *P* values less than 0.05. Results were analyzed using Prism software V.8.0 (GraphPad). RNA‐seq analysis was performed using R v.4.3.1. Differentially expressed genes (DEGs) were assessed by comparing migrated with non‐migrated CD8+ T cells using a Wald test and a false discovery rate (FDR) under the 0.05 threshold^21^ by applying an independent data filtering based on the mean of normalized counts for each gene and optimizing the number of genes having an adjusted *P* value (FDR) of less than 0.05. R packages ggplot2 v.3.5.0 and Complex Heatmap v.2.16.0 were used to generate volcano plot and heatmaps, respectively.

## RESULTS

### CD8+ but not CD4+ T cells in patients with SpA exhibit high migration in the absence of chemokine stimuli

**Figure 1:**
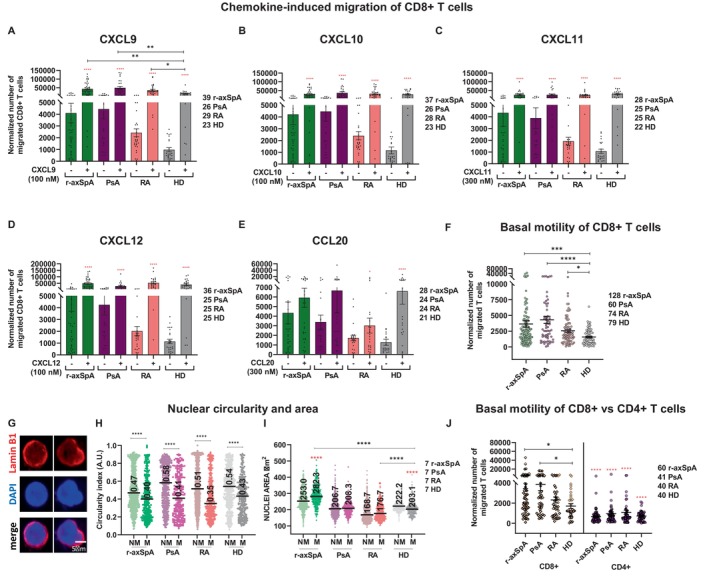
CD8+ but not CD4+ T cells in patients with SpA possess an intrinsic higher motility compared with HD. (A–E) CD8+ T cell migration ability toward the indicated chemokines is shown. (F) The number of cells spontaneously migrating is higher in patients with immune‐mediated diseases, especially those with SpA, than in HD. Red asterisks, Wilcoxon test for analysis within the same cohort, * *P* value < 0.05; **** *P* value < 0.0001; black asterisks, Kruskal‐Wallis test for differences among cohorts; * *P* value < 0.05; ** *P* value < 0.01; *** *P* value < 0.001; **** *P* value < 0.0001. (G) Upper and middle panels show the nuclear shape of migrated CD8+ T cells stained by anti‐lamin B1 mAb and DAPI, respectively; merge is shown in the bottom panel. Left and right panels are representative of cells with high and low CI, respectively. (H) The migrated cells from all cohorts exhibited a lower CI, whereas the nuclear area (I) is larger in migrated versus non‐migrated cells of all patients whereas the opposite is found in HD. (J) Comparison of spontaneous migration in CD8+ (on the left) and CD4+ T cells (on the right) of patients and HD. Mann–Whitney test **** *P* value < 0.0001. The difference in the motility of CD8+ and CD4+ T among the cohorts was analyzed by the Kruskal‐Wallis test, *P value < 0.05. Mean ± SEM in (A–F) and (J) and median in (H) and (I) are reported. A.U., arbitrary units; CI, circularity index; HD, healthy donor; M, migrated CD8+ T cells; mAb, monoclonal antibody; NM, non‐migrated; PsA, psoriatic arthritis; RA, rheumatoid arthritis; r‐axSpA, radiographic axial spondyloarthritis; SpA, spondyloarthritis.

Chronic inflammatory conditions in SpA are sustained by continuous trafficking and recruitment of immune cells at target sites. Although a role for CD8+ T lymphocytes in SpA pathogenesis has not been fully clarified, recent findings have relaunched their involvement.^8^, ^9^, ^10^, ^11^ Herein, we asked whether an altered CD8+ T migratory pattern might occur in SpA by testing the chemotactic properties of peripheral CD8+ T lymphocytes from patients with r‐axSpA and PsA, who both belong to the SpA cluster, compared with age‐matched HD. Moreover, to deepen the chronic inflammation impact on the migratory pattern, in a cohort of patients with RA, a rheumatic SpA‐unrelated, chronic inflammatory disease was also analyzed. The migration was assessed by transwell assays toward the proinflammatory chemokines CXCL9, CXCL10, and CXCL11, which bind the CXC chemokine receptor 3^22^ (Figure 1A–C), or toward the homeostatic chemokine CXCL12, a ligand of CXCR4^23^ (Figure 1D). Additionally, the CCL20‐CCR6 axis (Figure 1E) was evaluated given its involvement in T cell recruitment to gut mucosa and skin.^24^

CD8+ T cells from all cohorts properly migrated upon chemokine induction. Those from patients with SpA and RA exhibited a significantly higher response to CXCL9 compared with HD (Figure 1A), whereas the four cohorts showed a comparable chemotaxis to CXCL10, CXCL11, and CXCL12 (Figure 1B–D). Migration in response to CCL20 was generally low, especially in the cohort with RA compared with HD (Figure 1E). However, the observed differences seem to not be related to an altered expression of the cognate receptors (Supplementary Figure 1A–E).

Intriguingly, CD8+ T cells from patients with SpA disclosed an intrinsic higher motility in the absence of chemotactic stimuli when compared with HD and patients with RA (Figure 1A–E). To exclude a biased effect of the small cohorts analyzed, the sample size was increased up to 128 patients with r‐axSpA, 60 with PsA, 74 with RA, and 79 HD (Figure 1F). Our data confirmed a higher spontaneous migration of CD8+ T cells in patients with SpA (both HLA‐B*27‐positive and ‐negative carriers) (Supplementary Figure 2) versus HDs (mean ± SD for patients with r‐axSpA 3,638 ± 518.6; PsA 4,296 ± 574.5; and HD 1,572 ± 137.9). Although less pronounced, this finding extended to patients with RA (mean ± SD 2,575 ± 249.9), which suggests that a chronic inflammatory state could influence the basal motility of CD8+ T cells. We asked whether drug treatment could affect spontaneous migration. Notably, no significant differences of CD8+ T cell basal motility were found in untreated versus biologic disease‐modifying antirheumatic drug (bDMARD)–treated patients within cohorts of SpA (r‐axSpA and PsA) and RA (Supplementary Figure 3).

To verify whether the migration detected by transwell assay was affected by the cellular ability to deform while crossing the membrane pores or by a nuclear size decrease, the nuclear shape and dimension were evaluated. Hence, migrated and non‐migrated CD8+ T cells from seven patients with r‐axSpA, PsA, and RA, and HD were stained with anti‐lamin B1 mAb and DAPI and analyzed for both nuclear CI and area. Two representative cell types of high (left panels) and low (right panels) nuclear CI are reported (Figure 1G). The nuclear CI was lower in migrated versus non‐migrated CD8+ T cells in all cohorts (Figure 1H), which suggests that migration is indeed affected by nuclear deformability. Contrarily, the nuclear area was larger in migrated versus non‐migrated CD8+ T cells from r‐axSpA, unchanged in cells from PsA and RA cohorts, and reduced in migrated cells from HD (Figure 1I). These findings suggested that, in HDs, cell migration could be favored by a reduced nuclear size, whereas the higher migratory profile in patient cohorts mainly relied on nuclear deformability.

Given the involvement of CD4+ T cells in SpA,^25^ we concurrently assessed the basal motility of CD4+ and CD8+ T cells from patients (60 patients with r‐axSpA, 41 with PsA, 40 with RA) and 40 HDs. Within all cohorts, CD8+ T cells displayed a higher intrinsic motility compared with the CD4+ counterpart (Figure 1J). Overall, these observations might suggest that the inflammatory state, caused by both disease and/or aging (mean ± SD for age: 50 ± 14.1 in r‐axSpA; 55 ± 14.0 in PsA; 56 ± 13.6 in RA; and 51 ± 15.0 in HD), has a stronger impact on the basal migratory capabilities of CD8+ than CD4+ T cells.

### Transcriptional profiling of spontaneously motile CD8+ T cells

**Figure 2:**
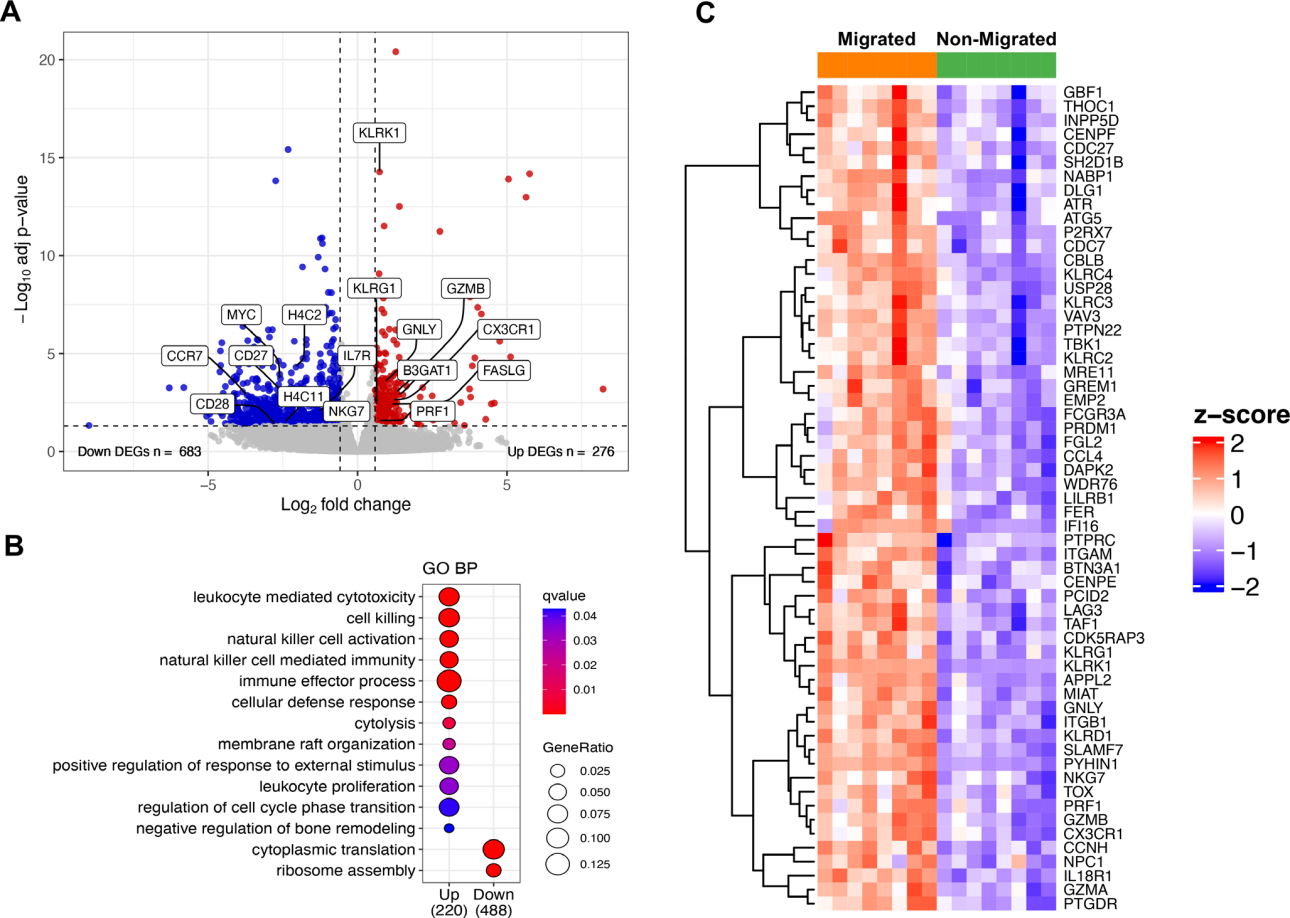
Transcriptome analysis of non‐migrated versus migrated CD8+ T cells. (A) Volcano plot showing the distribution of log_10_ (adjusted *P* values) values (*y‐*axis) relative to log_2_ (fold changes) values (*x‐*axis) resulting from the comparison of gene expression levels in migrated versus non‐migrated CD8+ T cells of eight patients with r‐axSpA. Red dots indicate up‐regulated DEGs associated with FDR < 0.05 and FC > 1.5 and blue dots indicate DEGs with FDR < 0.05 and FC < −1.5. Selected DEGs are highlighted in the plot. The number of up‐ and down‐regulated genes is reported. (B) Dot plot showing the GO terms enriched in the up‐regulated (left) and the down‐regulated (right) DEGs. The size of the dots is based on the ratio between the gene number belonging to a GO BP category and the total number of up‐ or down‐regulated genes. The color of the dots shows the *q* value associated with each term. (C) Heatmap showing row z‐score scaled gene expression levels of DEGs belonging to the up‐regulated GO BP enriched categories. Genes were hierarchically clustered using Euclidean distances. BP, biological process; DEG, differentially expressed gene; FC, Foldchange; FDR, false discovery rate; GO, gene ontology; r‐axSpA, radiographic axial spondyloarthritis.

To highlight DEGs correlated to the high intrinsic motility observed in CD8+ T cells of patients with SpA, transcriptomic analysis of migrated versus non‐migrated cells was performed (Supplementary Table 2 at accession number GSE266295 and Figure 2). RNA samples were collected from non‐migrated and migrated CD8+ T cells from eight patients with r‐axSpA. Overall, 276 protein coding genes were up‐regulated and 683 genes were down‐regulated in migrated versus non‐migrated CD8+ T cells (FDR < 0.05; foldchange [FC] > 1.5) (Figure 2A). Interestingly, gene ontology (GO) analysis (Figure 2B) highlighted several enriched biologic processes (BPs) in migrated CD8+ T cells pointing out a cytolytic signature and natural killer (NK) features (Figure 2B and C). Indeed, many up‐regulated transcripts were related to the activation and cell killing activity shared by NK and CD8+ T cells (*SLAMF7*, *PRDM1*, *PTPRC*, *NKG7*, *SH2D1B*, *IL18R1*, *PTPN22*, *TOX*, *FCGR3A*, *EMP2*, *P2RX7*, *CX3CR1*, *GZMA*, *GZMB*, *GNLY*, *PRF1*, *ITGB1*, *INPP5D*) and others to NK receptors (*KLRD1*, *KLRC4*, *KLRC2, KLRC3*, *KLRK1*, *LILRB1*) (Figure 2C). In addition, the down‐modulation of GO BPs related to cytoplasmic translation and ribosome assembly (Figure 2B and Supplementary Table 2) and the up‐regulation of classical senescence and exhaustion‐related genes (*KLRG1*, *B3GAT1*, *LAG3*) (Figure 2C) suggested that the migrated subset could be less metabolically active and features a terminally differentiated/senescent T cell compartment.

### Spontaneously migrating CD8+ T cells possess a terminally differentiated/senescent‐like phenotype

**Figure 3:**
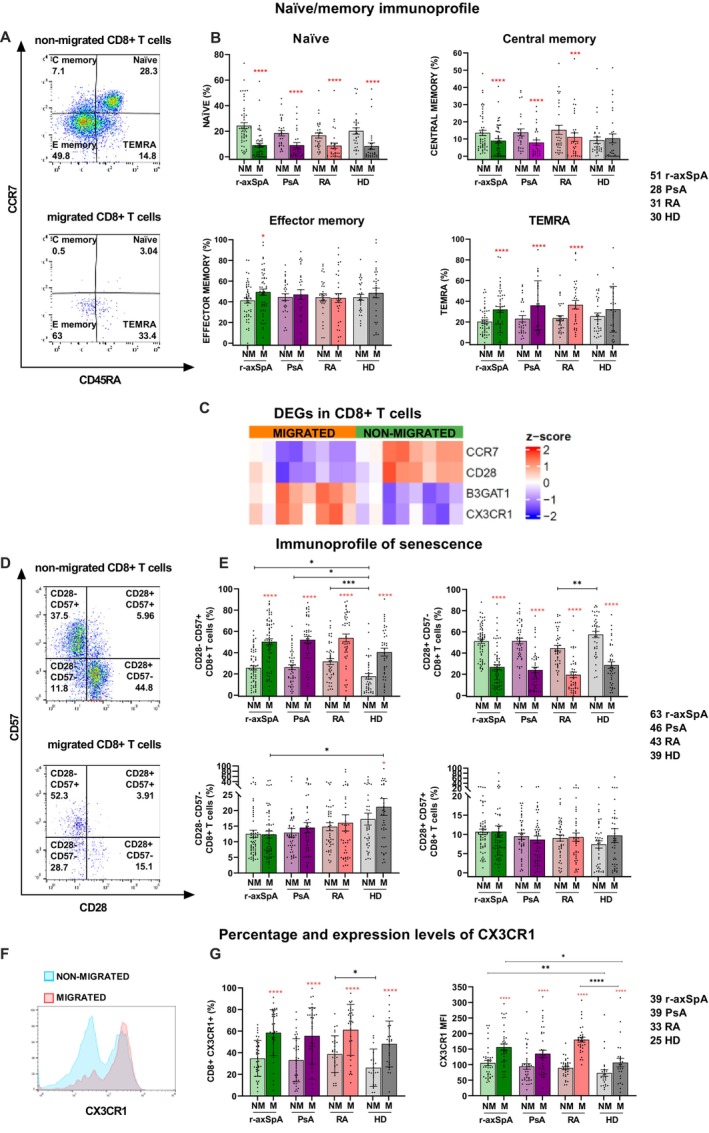
Spontaneously migrating CD8+ T cells are enriched in terminally differentiated/senescent cells. (A) Representative flow cytometry plots of naive/memory composition in non‐migrated versus migrated CD8+ T cells. (B) After basal migration there was a decrease of CD8+ T naive and central memory cells; the former was in all cohorts and the latter was only in patients, with a parallel enrichment of effector memory cells in the migrated fraction of patients with r‐axSpA and TEMRA subset in migrated cells of all patients but not in HD. (C) Heatmap of relevant DEGs (FC > 1.5; FDR < 0.05) in migrated versus non‐migrated CD8+ T cells (r‐axSpA, n = 8). (D) and (E) The analysis of senescence status, which was more pronounced in migrated cells compared with non‐migrated ones, as reported in the representative plot (D), has been done by CD28 and CD57 staining. (F) Representative plot showing overlapped CX3CR1 expression in migrated (red line) and non‐migrated (blue line) CD8+ T cells. (G) The CX3CR1 amount, expressed as percentage and MFI, increased after the migration in the absence of stimuli within all cohorts. Red asterisks; Wilcoxon test pre‐ and post‐migration within the same cohort, * *P* value < 0.05; *** *P* value < 0.001; **** *P* value < 0.0001; black asterisks; Kruskal‐Wallis test for the comparison among the four cohorts, * *P* value < 0.05; ** *P* value < 0.01; *** *P* value < 0.001. Mean ± SEM is reported in B, E, and G. DEG, differentially expressed gene; FC, Foldchange; FDR, false discovery rate; HD, healthy donor; NM, non‐migrated; M, migrated CD8+ T cells; MFI, mean level per cell; PsA, psoriatic arthritis; RA, rheumatoid arthritis; r‐axSpA, radiographic axial spondyloarthritis.

To validate the observations from transcriptomics, naive/memory and senescent profiles of non‐migrated versus spontaneously migrated CD8+ T cells were analyzed by flow cytometry (Figure 3 and Supplementary Figure 4). CCR7 and CD45RA expression (Figure 3A) allows for the distinction of four CD8+ T cell subsets: naive (CCR7+CD45RA+), central memory (CCR7+CD45RA−), effector memory (CCR7−CD45RA−), and effector memory cells re‐expressing CD45RA (TEMRA) (CCR7−CD45RA+). No difference was found among the four cohorts in terms of naive/memory composition either in migrated or non‐migrated cells (Figure 3B). Conversely, the frequencies of each subset before and after migration showed the lowering of naive cells in the migrated CD8+ T cell pool from all cohorts (Figure 3B). This was consistent with the CCR7 down‐modulation observed in the migrated fraction by RNA‐seq analysis (Figure 3C). The same trend was shown by the central memory subset that decreased in migrated CD8+ T cells of all cohorts except HDs (Figure 3B). Additionally, an enrichment of effector memory cells in migrated CD8+ T cells from patients with r‐axSpA (Figure 3B) and of TEMRA in the migrated cells from all patients was observed, but not in HDs (Figure 3B).

For the immunosenescent profile, CD28 and CD57 were detected that allowed for the identification of four activation/senescence levels: nonactivated/early‐activated CD28+CD57−, activated CD28+CD57+, activated/early‐senescent CD28‐CD57−, and terminally differentiated‐senescent‐like CD28−CD57+ T cells (Figure 3D). Consistent with the enrichment of effector memory and TEMRA cells found upon cell migration, an increased percentage of CD28−CD57+ T cells in migrated versus non‐migrated cells was observed (Figure 3E). Accordingly, RNA‐seq analysis showed CD28 down‐modulation and CD57 up‐regulation in migrated versus non‐migrated CD8+ T cells from patients with r‐axSpA who were analyzed (Figure 3C). Interestingly, terminally differentiated‐senescent‐like CD28−CD57+ T cells were more abundant in non‐migrated CD8+ T cells from all patients compared with HDs (Figure 3E). This enrichment was also found before migration in patients with SpA compared with HDs (Supplementary Figure 5). Of note, patients with RA showed a lower occurrence of nonactivated/early‐activated CD28+CD57− T subset in non‐migrated cells compared with HD (Figure 3E). Additionally, CD28−CD57− cells were found enriched exclusively in migrated cells from HD and more abundant compared with patients with r‐axSpA (Figure 3E). No significant differences were observed for CD28+CD57+ cells (Figure 3E). Notably, the comparison of spontaneously migrated versus chemokine‐treated cells in patients with SpA showed a higher percentage of TEMRA and senescent cells in the former pool (Supplementary Figure 6). A low percentage of CD8+ T cells expressed PD1, a T cell exhaustion marker, without differences between migrated and non‐migrated pools, as anticipated by transcriptomic data (Supplementary Figure 7).

Furthermore, the expression of CX3CR1, a marker of CD8+ T cell differentiation state, highly expressed by long‐lived CD8+ T effector memory cells with cytolytic properties,^26^, ^27^, ^28^ was evaluated after migration (Figure 3F and G). Consistently with RNA‐seq data, we found a higher CD8+CX3CR1+ T cell percentage in the migrated pool from all cohorts (Figure 3C, F, and G). Notably, the CD8+CX3CR1+ percentage was higher in non‐migrated cells from patients with RA than in HDs (Figure 3G). These data positively correlated with age of patients, which suggests that physiologic aging with chronic inflammation strongly affect the CD8+ T cell differentiation state. Accordingly, CX3CR1 amount, measured through the mean fluorescence intensity (MFI), was higher in migrated cells from all groups compared with non‐migrated cells (Figure 3G). Interestingly, both non‐migrated and migrated CD8+ T cells from patients with r‐axSpA and migrated cells from patients with RA showed higher CX3CR1 MFI than HD (Figure 3G). Altogether, these data link the higher spontaneous migration with a terminally differentiated/senescent T cell status.

### Spontaneously migrating CD8+ T cells exhibit increased cytolytic and proinflammatory functions

**Figure 4:**
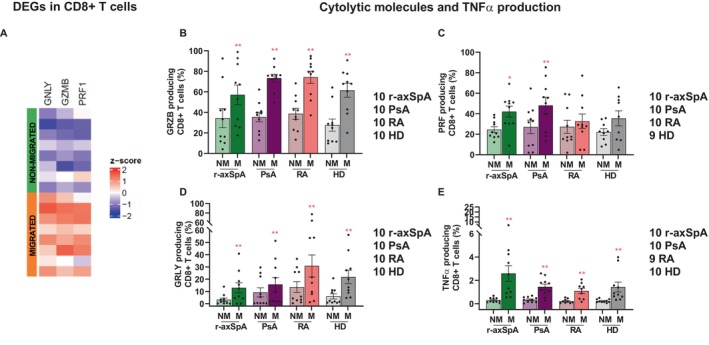
Senescent spontaneously migrating CD8+ T cells exhibit increased cytolytic and proinflammatory functions. (A) Heatmap of up‐regulated genes involved in cytolytic functions in migrated versus non‐migrated CD8+ T cells (FC > 1.5; FDR < 0.05) of the eight patients with r‐axSpA. (B–E). Migrated cells exhibited higher production of granzyme B (B), granulysin (D), and TNFα (E) in all cohorts, whereas a significant increase of perforin production (C) was detected only in migrated cells of patients with SpA; (red asterisks; Wilcoxon test before and after the migration within the same cohort of individuals, **** *P* value < 0.0001; ** *P* value < 0.01; * *P* value < 0.05); (Kruskal‐Wallis test for the comparison among the four cohorts, *P* value ns). Mean ± SEM is reported. DEG, differentially expressed gene; FC, Foldchange; FDR, false discovery rate; GRLY, granulysin; GRZB, granzyme B; HD, healthy donor; M, migrated CD8+ T cells; NM, non‐migrated; ns, not significant; PRF, perforin; PsA, psoriatic arthritis; RA, rheumatoid arthritis; r‐axSpA, radiographic axial spondyloarthritis; SpA, spondyloarthritis; TNFα, tumor necrosis factor alpha.

Afterward, we asked whether data from RNA‐seq analysis could be confirmed at protein level and translated into functional properties. The production of cytolytic molecules, such as granzyme B, perforin, and granulysin, which have genes that are up‐regulated from transcriptomics (Figure 4A–D and Supplementary Figure 8), was then evaluated. Additionally, the TNFα secretion was also assessed (Figure 4D and Supplementary Figure 8); however, its transcript was not among DEGs discriminating non‐migrated from migrated cells (Supplementary Table 2) because it represents one major senescence‐associated secretory phenotype component and a crucial therapeutic target in SpA. Consistently, granzyme B production was higher in migrated versus non‐migrated cells (Figure 4B) in all cohorts, whereas the frequency of perforin‐secreting CD8+ T cells increased on migration only in patients with SpA (Figure 4C). Moreover, an increase of granulysin (Figure 4D) and TNFα production (Figure 4E) was observed in migrated versus non‐migrated cells both in patients and HDs with no significant differences among the cohorts. Overall, these data indicate that, even without stimulation, the basally migrating CD8+ T cell fraction was capable of producing a higher amount of proteins with cell killing and proinflammatory potential.

### Senescent spontaneously migrating CD8+ T cells display shorter and damaged telomeres especially in patients with SpA

**Figure 5:**
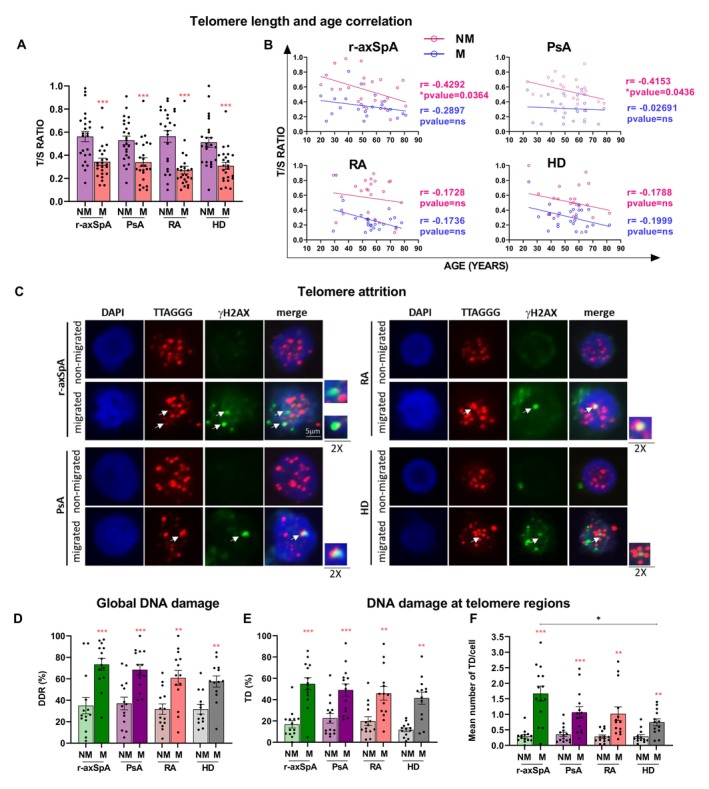
Higher TD in migrated versus non‐migrated CD8+ T cells. (A) The analysis of telomere length, expressed as T/S ratio, in non‐migrated and migrated CD8+ T cells (r‐axSpA, n = 24; PsA, n = 24; RA, n = 24; HD, n = 24) showed a shortening in the latter groups in all cohorts. (B) Negative trend of correlation of telomere length with the age both in migrated and non‐migrated cells (Spearman r correlation, * *P* value < 0.05). Cohorts were age‐matched (the mean ± SD age was 52 ± 17.2 in r‐axSpA; 53.5 ± 14.6 in PsA; 55.3 ± 13.2 in RA, and 53.4 ± 13.6 in HD). (C) Each panel shows IF‐FISH analysis of non‐migrated and migrated CD8+ T cells in 1 representative out of 14 patients with r‐axSpA, 14 with PsA, 14 with RA, and 13 HD. The merge highlights the DNA damage in the telomeric regions (TD). (D) and (E) panels show the percentage of DNA damage in the whole DNA (% DDR) (D) and in the telomeric regions (% TD) (E), respectively. (F) The telomere dysfunction, expressed in terms of mean number of TD per cell, is reported. Red asterisks, Wilcoxon test before and after the migration within the same cohort of individuals, ** *P* value < 0.01; *** *P* value < 0.001; **** *P* value < 0.0001; black asterisks, Kruskal‐Wallis test for the comparison among the four cohorts, * *P* value < 0.05. Mean ± SEM is reported in (A) and (D–F). DDR, DNA damage response; HD, healthy donor; IF‐FISH, immunofluorescence‐fluorescence in situ hybridization; NM, non‐migrated; M, migrated CD8+ T cells; PsA, psoriatic arthritis; RA, rheumatoid arthritis; r‐axSpA, radiographic axial spondyloarthritis; TD, telomere dysfunction.

Recent studies highlighted the occurrence of premature T cell senescence in axial SpA and patients with RA, but the underlying mechanisms and the contribution to inflammation at target tissues have not been elucidated as of yet.^17^, ^29^, ^30^, ^31^ Herein, we evaluated the telomere length in migrated versus non‐migrated CD8+ T cells from 24 patients with r‐axSpA, 24 with PsA, 24 with RA, and 24 HDs. Figure 5A reports the length of telomeres as a T/S ratio that indicates the number of copies of telomere repeats (T) compared with a single copy control gene (S; β‐globin). In line with the senescence state detected by flow cytometry (Figure 3), the migrated CD8+ T cells were characterized by shorter telomeres compared with the non‐migrated counterpart in all groups. The telomere shortening displayed a trend of positive correlation with the age in migrated and non‐migrated cells that reached a statistical significance in non‐migrated CD8+ T cells from patients with r‐axSpA and patients with PsA (Figure 5B).

Data from telomere length measurement (Figure 5A) raised the question of whether shortened telomeres found in spontaneously migrating CD8+ T cells were still able to maintain capping structures that prevent DDR activation and telomere dysfunction (TD). Therefore, the expression of the phosphorylated form of γ‐H2AX, a DDR marker, was analyzed in migrated versus non‐migrated CD8+ T cells from 14 patients with r‐axSpA, 14 with PsA, 14 with RA, and 13 HDs. In parallel, to assess if DDR activation was due to telomere attrition, we analyzed the percentage of cells displaying telomeric signals and γ‐H2AX colocalization by immunofluorescence‐fluorescence in situ hybridization (IF‐FISH) experiments. In all cohorts, a higher percentage of DDR and TD positive cells was detected in migrated cells (Figure 5C–F). Accordingly, the TD number estimation per nucleus revealed an increase of the TD average number in migrated versus non‐migrated CD8+ T cells in all cohorts (Figure 5F). More importantly, among the migrated cells, only CD8+ T cells from patients with r‐axSpA displayed a significant increase in the TD number compared with HDs (Figure 5F). Overall, these results correlate the spontaneous migration of CD8+ T cells with an advanced senescent state characterized by telomere shortening, DDR activation, and TD, which was more pronounced in migrated CD8+ T cells from patients with r‐axSpA.

## DISCUSSION

In this study, we describe a subpopulation of peripheral CD8+ T cells significantly increased in patients of the SpA cluster (r‐axSpA and PsA) (Figure 1) that spontaneously migrates regardless of specific chemokine gradients. Notably, this CD8+ T cell subset shows a senescent/terminally differentiated immunoprofile, DDR activation, telomere shortening, and dysfunction and retains cytotoxic and proinflammatory functions (Figures 2, 3, 4, 5) and is also detectable with lower frequency in patients with RA and elderly HD participants (mean ± SD age: 51 ± 15). Thus, it is conceivable to speculate that these intrinsically “hypermotile” CD8+ T cells could be a byproduct of the inflammaging, which is the basal state of inflammation naturally increasing during aging, and, more importantly, in immune‐mediated disorders.

Many studies linked the chronic inflammation associated with rheumatic and other disorders to alterations of the telomere/telomerase system through molecular mechanisms that are still unclear.^31^, ^32^ Yet, nuclear factor kappa B (NF‐κB), a key regulator of inflammation, may induce changes in the telomere length and telomerase activity through the regulation of several telomere components.^32^ Moreover, TNFα and p38 MAPK signaling pathways can induce telomere shortening either through direct NF‐κB activation or by promoting the release of activating transcription factor 7 (ATF7) and telomerase from telomeres on ATF7 phosphorylation by p38.^33^

A cross‐sectional study reported a significantly longer telomere length in PBMCs from patients with r‐axSpA, PsA, and RA than in HDs.^34^ Later on, the same authors described a PBMC telomere length decay more accelerated in patients with PsA than in patients with r‐axSpA.^35^ This finding was attributed to different PBMC telomere physiology dynamics influenced by turnover rate and oxidative stress‐induced telomere damage between the two forms of SpA.

In axial SpA, a premature CD8+ and CD4+ T cell senescence was documented in patients younger than 35 years who presented inappropriate telomere shortening, shrinkage of thymic output, and altered telomerase activity; however, the question remains as to whether these alterations precede or follow the clinical disease onset.^17^ Our data do not show telomere length differences between patients and controls, neither comparing the spontaneously migrating CD8+ T cells nor the bulky non‐migrating cells (Figure 5A). Instead, we find shorter telomeres in migrated compared with non‐migrated cells in all groups and a general trend of inverse correlation between telomere length and age that reached statistical significance in the non‐migrated but not in the migrated CD8+ T cell pool from patients with SpA. This suggests that other factors besides the age impact the telomere status in the latter (Figure 5A and B).

Interestingly, spontaneously migrating CD8+ T cells from all cohorts exhibited a more pronounced DDR activation and a higher number of telomeric damage foci/cell that outlines a senescence phenotype (Figure 5C–F). In particular, the highest DDR and telomere attrition were found in migrating CD8+ T cells from patients with SpA that also displayed the highest rate of motility (Figures 1 and 5C–F). Notably, the nuclear circularity analysis revealed a certain deformity due to a nonuniform lamin B1 distribution at the nuclear periphery in migrating versus non‐migrating CD8+ T cells in all cohorts (Figure 1G and H).

Recently, it has been shown that lamin isoforms bind the linker of nucleoskeleton and cytoskeleton complex and their loss softens the cell nucleus and enhances constricted cell migration that contributes to migration‐induced DNA damage.^36^ Lamin B1 has been implicated in inflammation, cellular senescence, age‐associated organ dysfunctions, and human disorders.^37^ Moreover, growing evidence reports a direct interaction between telomere components and lamins that suggests a possible link between telomere attrition/dysfunction and nuclear architecture.^38^ Although in migrating CD8+ T cells lamin B1 gene is not modulated, its uneven distribution could alter the stoichiometric assembly of the nuclear lamina inducing alterations of cell mechano‐sensitivity and motility.^39^

Our data revealed a terminally differentiated and senescent profile of CD8+ T cells endowed with intrinsic motion (Figures 2 and 3). Importantly, an increase of TEMRA subset and a decrease of central memory subset was found in the pool of migrated CD8+ T cells from all patients, but not from HD (Figure 3A–C). These immunophenotypic differences suggest that the spontaneously migrated fraction from patients might be enriched in cells chronically stimulated by self and/or microbial antigens from persistent viruses or altered microbiota.^16^, ^40^, ^41^, ^42^ However, an intestinal origin of these spontaneously migrating CD8+ T cells is not supported by RNA‐seq data because α_4_ (ITGA4) and β_7_ (ITGB7) integrin genes are not up‐regulated (data not shown).^17^ In patients with r‐axSpA, an expansion of circulating cytotoxic CD8+CD28− T cells has been documented and their percentage correlated with the disease status but not with the age.^43^ A strong enrichment of CD8+CD28−CD57+ T cells, classically defined as terminally differentiated‐senescent‐like cells, was found in the migrated pool from all cohorts (Figure 3C–E). Remarkably, the percentage of such cells was already higher in the bulky non‐migrated cells of patients compared with HDs (Figure 3E), which supports a role for chronic inflammation in accelerating T cell immunosenescence.^30^, ^44^ In addition, the up‐regulation of genes implicated in cytotoxic functions and NK‐like trait (Figure 2) and the production of cytolytic and proinflammatory proteins (Figure 4) in spontaneously migrated CD8+ T cells delineated a cytotoxic profile. Accordingly, recent single cell cellular indexing of transcriptomes and epitopes by sequencing (CITE‐seq) data showed that a hallmark of peripheral effector memory CD8+ T cells in patients with r‐axSpA is the overexpression of cytotoxicity‐related genes, including GZMH, GZMB, and NKG7.^45^

Our data also evidence a significantly higher expression of the fractalkine receptor, CX3CR1, in spontaneously migrating compared with nonmigrating CD8+ T cells (Figure 3F and G). CX3CR1 graded transcript levels have been proven to reflect the CD8+ T cell differentiation continuum with higher expression levels correlated to stronger cytotoxicity.^26^, ^27^, ^28^ Recently, a circulating CD8+CCR4+ subpopulation displaying up‐regulation of genes promoting osteogenesis has been described in patients with r‐axSpA with active disease.^46^ Consistently with our data, those CD8+CCR4+ cells expressing CX3CR1 exhibited an enhanced cytotoxicity. Integrated meta‐analyses of gene expression profiles identified CX3CR1 as a marker for rheumatic diseases.^47^ Additionally, a study to develop a predictive disease risk model for r‐axSpA acknowledged CX3CR1 as a potential biomarker for early disease diagnosis and progression.^48^

A mechanistic explanation linking CD8+ T cell spontaneous motility with the senescent/cytotoxic phenotype is still lacking. In line with the observed functional phenotype, transcriptomic analysis highlighted in migrated CD8+ T cells, several key genes involved in cell motility, cytoskeleton organization, DNA damage, and telomere stress‐induced senescence, but they were not implicated in GO BPs significantly enriched (Figure 2B and Supplementary Table 2). In patients with RA, Li et al associated premature aging of CD4+ T cells showing a hypermotile, tissue‐invasive, and highly arthritogenic in vivo phenotype with a defective expression of the DNA double strand break repair nuclease MRE11A.^29^ In our study, migrated CD8+ T cells from patients with r‐axSpA showed up‐regulation of *MRE11* (Figure 2B), which indicates that in our context other mechanisms induce DDR activation. Moreover, our data underlined a low basal motility of CD4+ T cells that might reflect their lower susceptibility to senescence due to a different metabolism.^30^

The CD8+ T cell subset, which is characterized here, although intrinsically “hypermotile,” is also able to efficiently migrate following a chemotactic gradient (Figure 1A–E). Therefore, we can speculate that these circulating CD8+ T cells could reach the target tissues in patients with SpA attracted by inflammatory chemokine gradients or even by CX3CL1, which has an increased level in inflamed gut and synovial tissues but not in the blood of patients with r‐axSpA.^49^ Once in the target tissues, these cells could randomly move in the extracellular matrix supported by their intrinsic motility and create channels that may favor the spreading of other immune cells.^50^ Accordingly, intravital imaging of cytotoxic CD8+ T cells in pancreatic islets of diabetic mice showed that the cell homing kinetics is a stochastic process, which argues against a dominant influence of chemotactic gradients.^51^

Overall, these results shed light on a subpopulation of circulating CD8+ T cells that are more frequent in patients in the SpA cluster and that may contribute to chronic inflammation by coupling the intrinsic motion with a cytotoxic/proinflammatory profile. Deeper investigations will be required to understand the mechanistic relationship between TD/senescence and hypermotility, as well as the possible modulatory effects of bDMARDs on the immune and migratory phenotype of this T cell subset.

## AUTHOR CONTRIBUTIONS

All authors contributed to at least one of the following manuscript preparation roles: conceptualization AND/OR methodology, software, investigation, formal analysis, data curation, visualization, and validation AND drafting or reviewing/editing the final draft. As corresponding author, Dr Fiorillo confirms that all authors have provided the final approval of the version to be published, and takes responsibility for the affirmations regarding article submission (eg, not under consideration by another journal), the integrity of the data presented, and the statements regarding compliance with institutional review board/Declaration of Helsinki requirements.

## Supporting information


**Disclosure Form**:

**Appendix S1.** Supporting Information

**Supplementary Table 2** Patients with r‐axSpA (n = 8) are indicated as R1‐R8. Migrated and non‐migrated CD8+ T cells are reported as migrated and not_migrated, respectively. Genes information are taken from NCBI Gene Database (https://ftp.ncbi.nlm.nih.gov/gene/DATA/gene_info.gz) and from Ensembl/GENCODE annotations.
