# Supplementary material for: An Immunosenescent CD8+ T Cell Subset in Patients with Axial Spondyloarthritis and Psoriatic Arthritis Links Spontaneous Motility to Telomere Shortening and Dysfunction
Source: Arthritis Rheumatol. 2025 Feb 18;77(7):854–66. doi: 10.1002/art.43109 (PMC12209751; doi:10.1002/art.43109)
Supplement: Supplementary file 2 — Appendix S1. Supporting Information [file ART-77-854-s002.docx]

**Supplementary material**

**An immunosenescent CD8+ T cell subset in patients with axial Spondyloarthritis and Psoriatic Arthritis links spontaneous motility to telomere shortening and dysfunction**

Giorgia Paldino^1, †^, PhD, Valentina Tedeschi^1, †^, PhD, Valentina Proganò^1^, MSc, Erica Salvati^2^, PhD, Valerio Licursi^2^, PhD, Eleonora Vertecchi^2^, PhD, Alexandru L. Bivolaru^1^, MSc, Emanuele Molteni^3^, MD, Rossana Scrivo^3^, MD, PhD, Mattia Congia^4^, MD, Alberto Cauli^4^, MD, PhD, Rosalba Caccavale^5^, MD, Marino Paroli^5^, MD, Martina Kunkl^1, 6^, PhD, Loretta Tuosto^1^, PhD, Rosa Sorrentino^1^, PhD, and Maria Teresa Fiorillo^1^, PhD

^1^Department of Biology and Biotechnologies “Charles Darwin”, Sapienza University of Rome, Rome, Italy

^2^Institute of Molecular Biology and Pathology (IBPM), National Research Council (CNR), Rome, Italy

^3^Rheumatology Unit, Department of Clinical Internal, Anesthesiological and Cardiovascular Sciences, Policlinico Umberto I, Sapienza University of Rome, Rome, Italy

^4^Rheumatology Unit, Azienda Ospedaliero-Universitaria di Cagliari, Cagliari, Italy

^5^Clinical Immunology Unit, Department of Clinical Internal, Anesthesiological and Cardiovascular Sciences, ICOT Hospital, Sapienza University of Rome c/o Polo Pontino, Latina, Italy

^6^Neuroimmunology Unit, IRCCS Santa Lucia Foundation, Rome, Italy

^†^These authors contributed equally to this study

| **Supplementary Table 1.** Antibodies employed in flow cytometry and IF-FISH analyses | | | | |
| --- | --- | --- | --- | --- |
| Targeted human antigen | Fluorochrome | Clone | Isotype | Source |
| CXCR3 | PE | 1C6/CXCR3 | Mouse BALB/c IgG1, κ | BD Biosciences |
| CXCR4 | PE | 12G5 | Mouse BALB/c IgG2a, κ | BD Biosciences |
| CCR6 | PE | GO34E3 | Mouse IgG2b, κ | Sony Biotechnology |
| CCR7 | PE | 3D12 | Rat IgG2a, κ | BD Biosciences |
| CX3CR1 | PE | 2A9-1 | Rat IgG2b, κ | BD Biosciences |
| CD3 | PE | UCHT-1 | Mouse IgG1, κ | Immunotools |
| CD3 | APC | OKT3 | Mouse IgG2a, κ | Biolegend |
| CD45RA | FITC | HI100 | Mouse IgG2b, κ | BD Biosciences |
| CD28 | FITC | CD28.2 | Mouse IgG1, κ | BD Biosciences |
| CD57 | APC | HNK-1 | Mouse IgM, κ | Sony Biotechnology |
| GRANZYME B | FITC | QA16A02 | Mouse IgG1, κ | Sony Biotechnology |
| PERFORIN | PE | B-D48 | Mouse IgG1 | Sony Biotechnology |
| GRANULYSIN | APC | DH2 | Mouse IgG1, κ | Sony Biotechnology |
| TNFα  PD1 | PE  PE | Mab11  NAT105 | Mouse IgG1, κ  Mouse IgG1, κ | BD Biosciences  BD Biosciences |
| phospho-histone H2AX (Ser139) | none | JBW301 | Mouse IgG1 | Merck Millipore |
| LAMIN B1 | none | polyclonal | Rabbit IgG | Abcam |

**Supplementary Figure 1. CD8+ T motility towards the chemokines is independent of the frequency and expression levels of cognate receptors.** Percentage (A) and expression level, as relative MFI (rMFI) (B) of CXCR3 on CD8+ T cells analysed in the migration assay towards CXCL9, CXCL10 or CXCL11 chemokines are reported. No difference has been found among the cohorts (Kruskal-Wallis test, p value ns). (C) The amount of CXCR4, receptor for CXCL12, is reported only as rMFI, since all CD8+ T cells are positive for this receptor. CD8+ T cells from PsA patients showed lower levels of CXCR4 compared to the other groups (Kruskal-Wallis test, * p value < 0.05; ** p value < 0.01; *** p value < 0.001). In (D) the percentage of CCR6, receptor for CCL20, is shown; the frequency of CD8+CCR6+ T cells is higher in PsA patients compared to the other cohorts (Kruskal-Wallis test, * p value < 0.05; ** p value < 0.01; *** p value < 0.001). The levels of CCR6 receptor, reported as rMFI, are shown in (E); the amount of this receptor is higher in CD8+ T cells from r-axSpA patients than in PsA and RA patients (Kruskal-Wallis test, * p value < 0.05). The number of patients and controls analysed in each experiment is reported in the graph. Mean ± SEM is reported.

**Supplementary Figure 2. Spontaneous migration in HLA-B*27-positive and HLA-B*27-negative patients with SpA.** Basal migration of CD8+ T cells from patients with r-axSpA grouped in accordance with being HLA-B*27 carriers (n=102) or HLA-B*27-negative (n=26) on the left and PsA grouped in accordance with being HLA-B*27 carriers (n=11) or HLA-B*27-negative (n=49) on the right. Mann-Whitney test; p value = NS (0.1263 and 0.1015 in r-axSpA and PsA cohort, respectively). Mean ± SEM is reported.

**Supplementary Figure 3.** **Spontaneous migration of CD8+ T cells correlated to drug treatment.** Basal motility of CD8+ T cells from patients with SpA (r-axSpA and PsA), on the left, and patients with RA, on the right, grouped in accordance with being bDMARDs-treated or untreated. Mann-Whitney test; p value = NS (0.096 and 0.4394 in SpA and RA cohort, respectively). Mean ± SEM is reported.


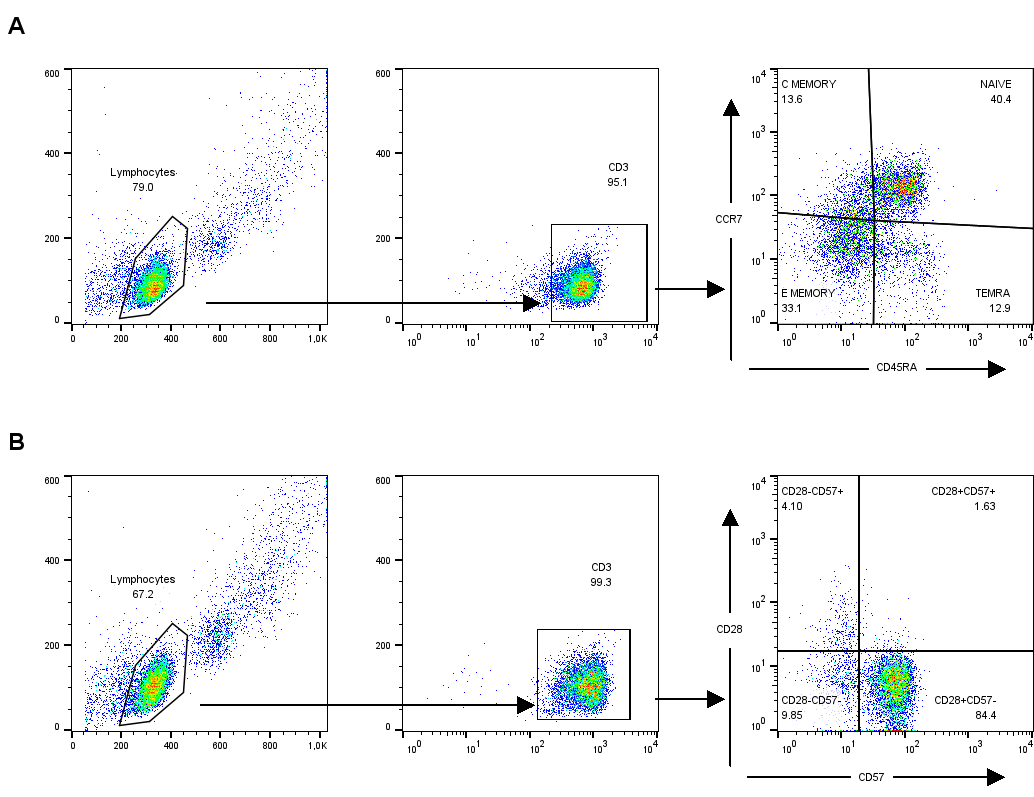
**Supplementary Figure 4. Gating strategy for naïve/memory (A) and senescent (B) CD8+ T cell immunoprofiling.** (A) To evaluate the naïve/memory immunoprofile of migrated and non-migrated CD8+ T cells, anti-CD3-APC, anti-CCR7-PE and anti-CD45RA-FITC mAbs were used. (B) To distinguish between early/activated and senescent CD8+ T cells in migrated and non-migrated subsets, anti-CD3-PE, anti-CD28-FITC and anti-CD57-APC mAbs were used.

*
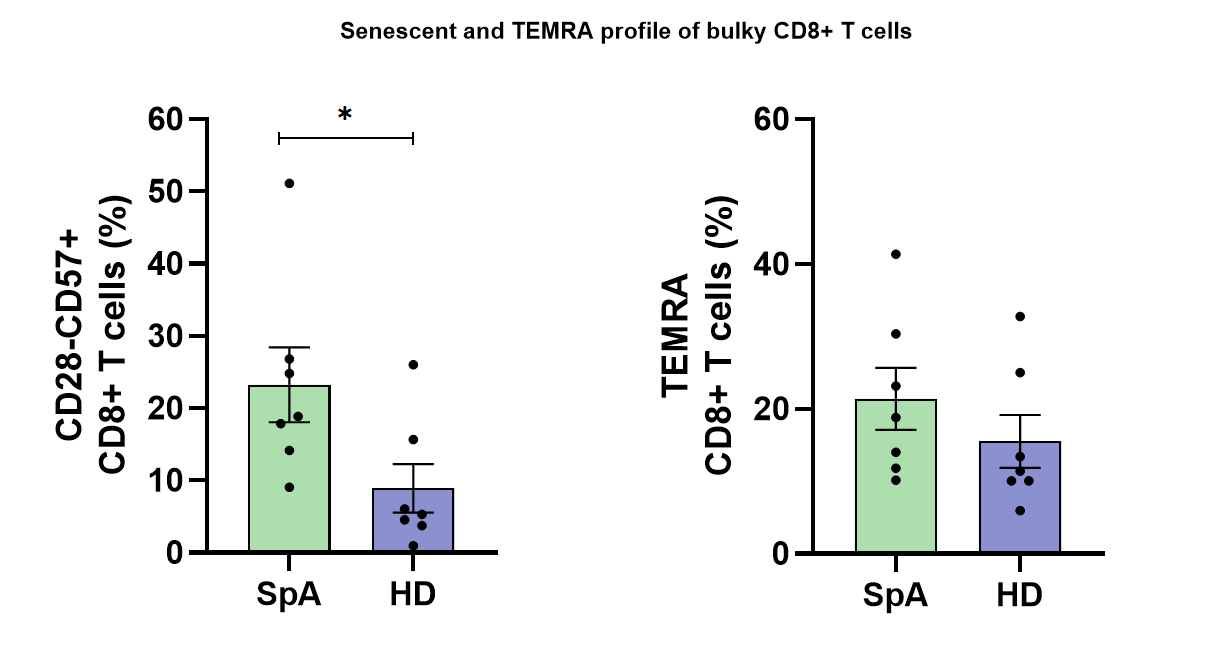
*

**Supplementary Figure 5. Senescent and TEMRA profile of *in toto* CD8+ T cells before migration in SpA patients *vs* healthy subjects.** Percentage of senescent CD28-CD57+ (on the left) and TEMRA CCR7-CD45RA+ (on the right) of *in toto* CD8+ T cells from 7 SpA patients compared to 7 healthy donors (HD). Mann-Whitney test; *p value<0.05. Mean ± SEM is reported.

*
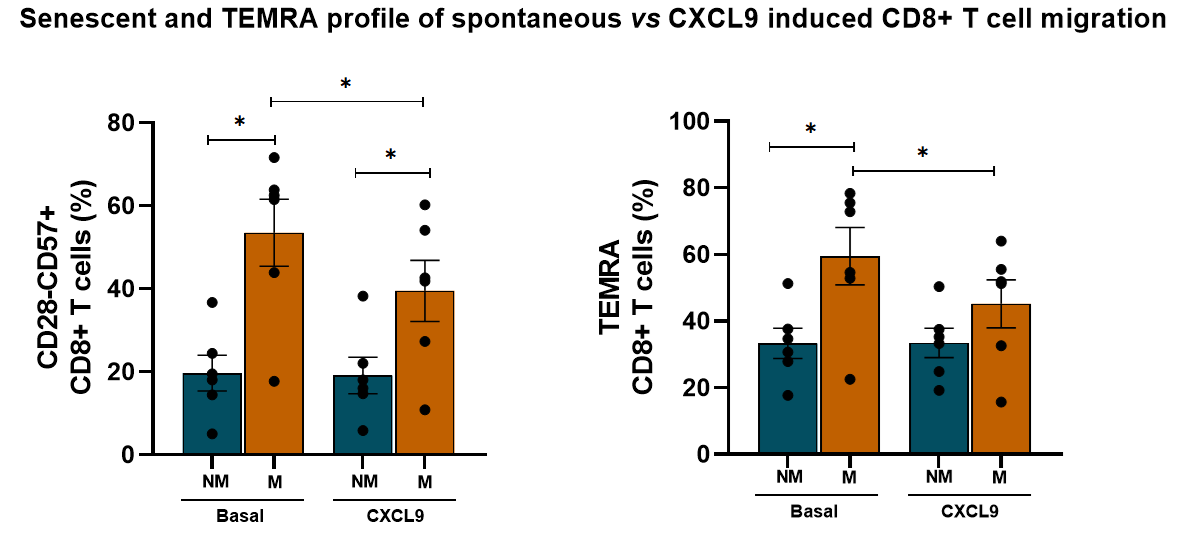
*

**Supplementary Figure 6.** **Comparison between basal and chemokine induced migration.** Percentage of senescent CD28-CD57+ (on the left) and TEMRA CCR7-CD45RA+ (on the right) in non-migrated (NM) *vs* migrated (M) CD8+ T cells from patients with SpA (n=6) both in absence and presence of CXCL9 (100 nM). Wilcoxon test; *p value <0.05. Mean ± SEM is reported.

**Supplementary Figure 7. Expression of PD1 by CD8+ T cells.** Percentage of PD1 expressing CD8+ T cells in non-migrated (NM) *vs* migrated (M) fractions in SpA patients (n=9). A low proportion of CD8+ T cells expresses PD1 and the differences between the two subsets are not statistically significant. Wilcoxon test. p value = NS (0.9102). Mean ± SEM is reported.


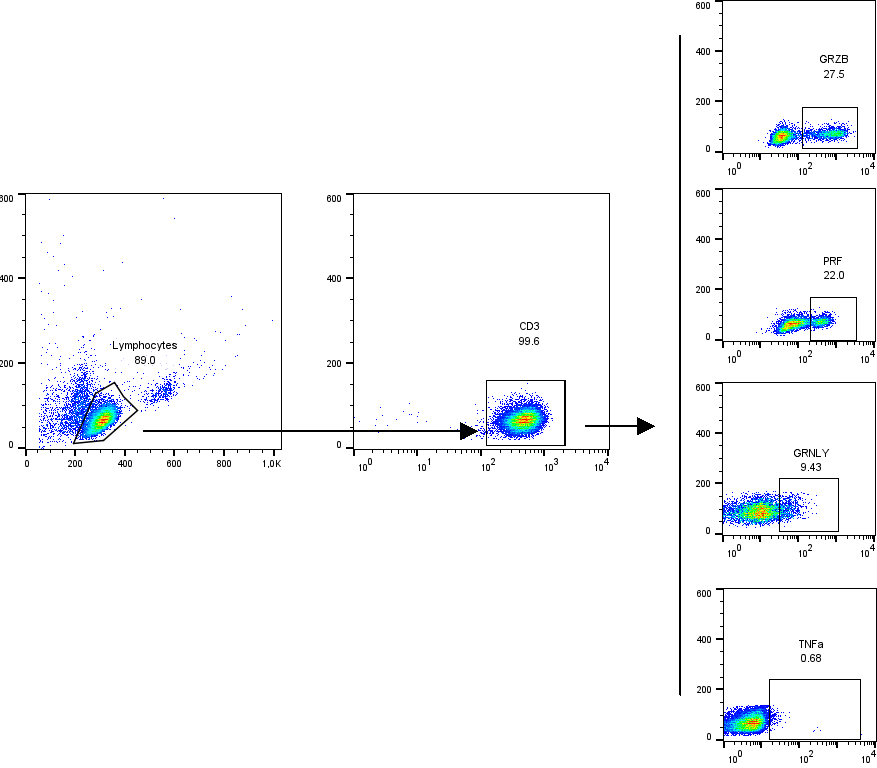
 **Supplementary Figure 8. Gating strategy to assess CD8+ T cells cytolytic and pro-inflammatory activity.** The cytolytic activity of CD8+ T cells has been analysed by staining with anti-CD3-APC, anti-GRZB-FITC and anti-PRF-PE mAbs or with anti-CD3-PE and anti-GNLY-APC mAbs; the pro-inflammatory cytokine has been detected by using an anti-CD3-APC and anti-TNFα-PE mAbs.

**Supplemental methods**

**RNA sequencing**

RNA-seq libraries were sequenced on paired-end 150 bp mode on NovaSeq 6000 (Illumina, San Diego, CA). Library preparation and sequencing were executed by IGATech (Udine, Italy). The resulting raw reads were assessed for quality by FastQC (v.0.12.0, Babraham Institute Cambridge, UK) and pre-processed with FASTQ Toolkit v.2.2.5 for adapter trimming and low quality (Q<30) filtering of the reads. Later on, reads were aligned to the reference Genome assembly GRCh38 and GENCODE Gene Set v.38 for gene annotation using salmon aligner (v.1.5)^1^ using a decoy-aware transcriptome index with k-mers of length 31 and normalizing for local GC content. Transcripts were merged to genes using Bioconductor^2^ R (v.4.3.1) package tximport (v1.28.0)^3^. Gene-level normalization and identification of differentially expressed genes (DEGs) were performed with R package DESeq2 v.1.40.2^4^. DEGs in migrated vs non-migrated CD8+ T cell samples were clustered by functional annotation in gene ontology (GO) and pathway enrichment analysis using Bioconductor R package clusterProfiler^5^ v.4.8.0 with annotation from GO Biological Process database.

**Analysis of telomere length by rtPCR**

Reference DNA sample of known concentration (10 ng/μl; Human Male DNA, Thermoscientific) is serially diluted to yield 5 concentrations of DNA in 20 μl final volume (17 ng, 8.5 ng, 4.2 ng, 2.1 ng, 1 ng), required for subsequent normalization.

Each DNA sample is amplified in triplicate in a final volume of 20 μl containing:

- Sensimix 2x SYBR Hi-ROX (Bioline): 10 μl

- Primers (Eurofins):

- Tel Fw (5’ – CGG TTT GTT TGG GTT TGG GTT TGG GTT TGG GTT TGG GTT – 3’) 0,2 µM
- Tel Rev (5’ – GGC TTG CCT TAC CCT TAC CCT TAC CCT TAC CCT TAC CCT – 3’) 1,8 µM
- β-globin Fw (5’ – CAA CTT CAT CCA CGT TCA CC – 3’) 0,5 µM
- β -globin Rev (3’ – GCC ATC TAT TGC TTA CAT TTG C – 3’) 0,2 µM

- H_2_O distilled by volume

The Real-Time PCR amplification reaction is performed using the 7300 Real Time PCR System (Applied Biosystem) instrument. The amplification program includes the following cycles:

-Stage 1: Step 1: 95°C per 10’ (1x)

-Stage 2: Step 1: Step 1: 95°C per 15’’ (40x)

Step 2: 60°C per 1’ (40x)

-Stage 3: Step 1: 95°C per 15’’ (1x)

Step 2: 60°C per 1’ (1x)

Step 3: 95°C per 15’’ (1x)

Data are reported as T/S ratio indicating the number of copies of telomere repeats (T) on a control gene expressed in single copy (β -globin) normalized with a standard curve at known concentrations of DNA.

**Supplemental references**

1. Patro R, Duggal G, Love MI, et al. Salmon provides fast and bias-aware quantification of transcript expression. Nat Methods 2017;14:417-419.
2. Gentleman RC, Carey VJ, Bates DM, et al. Bioconductor: open software development for computational biology and bioinformatics. Genome Biol 2004;5:R80.
3. Soneson C, Love MI, Robinson MD. Differential analyses for RNA-seq: transcript-level estimates improve gene-level inferences. F1000Res 2015;4:1521.
4. Love MI, Huber W, Anders S. Moderated estimation of fold change and dispersion for RNA-seq data with DESeq2. Genome Biol 2014;15:550.
5. Wu T, Hu E, Xu S, et al. clusterProfiler 4.0: A universal enrichment tool for interpreting omics data. Innovation (Camb) 2021;2:100141.
