# Supplementary material for: An Immunosenescent CD8+ T Cell Subset in Patients with Axial Spondyloarthritis and Psoriatic Arthritis Links Spontaneous Motility to Telomere Shortening and Dysfunction
Source: Arthritis Rheumatol. 2025 Feb 18;77(7):854–66. doi: 10.1002/art.43109 (PMC12209751; doi:10.1002/art.43109)
Supplement: Supplementary file 3 — Supplementary Table 2 Patients with r‐axSpA (n = 8) are indicated as R1‐R8. Migrated and non‐migrated CD8+ T cells are reported as migrated and not_migrated, respectively. Genes information are taken from NCBI Gene Database (https://ftp.ncbi.nlm.nih.gov/gene/DATA/gene_info.gz) and from Ensembl/GENCODE annotations. [file ART-77-854-s001.pdf]

Symbol	Gene	Entrez	Average expression level	log2FoldChange	stat	pvalue	FDR	migrated_R1	migrated_R2	migrated_R3	migrated_R4	migrated_R5	migrated_R6	migrated_R7	migrated_R8	not_migrate	not_migrate	not_migrate	not_migrate	not_migrate	not_migrated_R6	not_migrate	not_migrated_R8	Synonyms	dbXrefs	chromosome	map_locatio	description	type_of_gen	Symbol_fron	Full_name_f	Nomenclatur	Other_desig	Modification_
LINC01317	ENSG00000228262	104355287	459.33	4.42	12.28	1.23385E-34	2.34875E-30	16.8768687	16.7969175	558.196347	230.455031	5051.62649	737.664564	302.205229	248.57981	0	1.14561232	37.0853926	11.0880213	67.9736607	22.07642967	24.2091062	23.283448	-	HGNC:HGNC:	2	2p22.3	long intergen	ncRNA	LINC01317	long intergen	O	-	20210302
PYHIN1	ENSG00000163564	149628	1981.31	1.28	10.35	4.10235E-25	3.90462E-21	1355.71546	2295.34124	3938.88965	3145.75596	3012.34147	2325.39532	3543.27224	2814.77565	528.016599	1138.28409	1530.4737	1270.90047	1566.00979	967.442624	1010.92456	1257.473786	IFIX	MIM:612677	1	1q23.1	pyrin and HIN	protein-codir	PYHIN1	pyrin and HIN	O	pyrin and HIN	20210302
LTB	ENSG00000227507	4050	183.94	-2.32	-9.14	6.03475E-20	3.82925E-16	183.073846	108.653162	11.5769631	44.5645692	9.40617715	123.618074	39.8315113	21.3995783	780.520287	398.198779	89.4626285	177.20521	129.352676	458.33062	217.54597	150.3581494	TNFC|TNFSF3	MIM:600978	6	6p21.33	lymphotoxin	protein-codir	LTB	lymphotoxin	O	lymphotoxin-	20210302
KLRK1	ENSG00000213809	22914	3192.95	0.74	8.82	1.1282E-18	5.36911E-15	3313.29018	4451.71873	5284.9536	4375.75051	2697.56465	4306.24376	4050.3846	3332.32032	1615.83632	2827.95987	3180.60551	2709.77802	1553.48016	2757.695621	2587.00582	2042.602316	CD314|D12S	MIM:611817	12	12p13.2	killer cell lect	protein-codir	KLRK1	killer cell lect	O	NKG2-D type	20210307
GPR85	ENSG00000164604	54329	331.83	5.76	8.77	1.75029E-18	6.66371E-15	27.2735758	19.647871	1135.28068	222.371122	3202.32661	324.014264	143.541252	106.187254	66.7909157	0.6816171	6.27300205	1.17023829	44.3308256	3.887714517	3.25894836	2.295973912	SREB|SREB2	MIM:605188	7	7q31.1	G protein-cou	protein-codir	GPR85	G protein-cou	O	probable G-p	20210302
RGPD4-AS1	ENSG00000230651	729121	212.93	5.05	8.68	3.91515E-18	1.24215E-14	52.9445844	50.2837669	1884.87663	1194.46192	40.7276159	30.1037371	14.5829386	13.8513136	0	0.58002392	94.2173026	24.2088938	4.78641888	0	0	1.224519333	-	HGNC:HGNC:	2	2q12.3	RGPD4 antise	ncRNA	RGPD4-AS1	RGPD4 antise	O	-	20210302
	ENSG00000280800		129712.92	-2.74	-8.64	5.53661E-18	1.50564E-14	54188.6417	249737.195	810.296284	1103.11157	5938.41258	2551.2905	3810.06714	6351.47949	665885.053	886132.53	2518.74644	2521.42188	32890.4685	46861.71248	39279.9004	74826.36857											
	ENSG00000280244		112.36	5.64	8.40	4.37539E-17	1.04112E-13	0	0	39.5894954	3.80859138	1523.77253	122.75745	53.1173277	34.1346067	0	0	1.70257078	0	17.5428857	0.62528543	0.63556778	0											
CCDC144BP	ENSG00000154874	284047	389.65	1.40	8.26	1.46532E-16	3.09931E-13	291.502641	185.746981	1304.73426	877.956642	484.933652	952.352414	161.625165	321.9121	111.546686	62.9467016	372.207465	346.805062	131.090317	363.2709905	77.6371436	188.0869095											
SYNE2	ENSG00000054654	23224	18380.00	0.89	7.97	1.62067E-15	3.08511E-12	19275.3446	20527.5721	26229.8827	28842.6949	25415.3545	18332.9146	26297.637	25419.551	9219.20302	13086.7233	15097.674	14922.9992	17842.7266	10514.45754	11520.029	11535.30497	EDMD5|KASH	MIM:608442	14	14q23.2	spectrin repe	protein-codir	SYNE2	spectrin repe	O	nesprin-2|KA	20210306
DOCK7	ENSG00000116641	85440	1732.42	2.75	7.88	3.36332E-15	5.82038E-12	838.965169	353.53673	2525.0347	1428.89973	9890.38448	5018.48412	2484.13826	2222.45249	422.2881	165.232814	269.270716	287.508771	1043.84259	260.1978496	143.648501	364.8017393	DEE23|EIEE2	MIM:615730	1	1p31.3	dedicator of	protein-codir	DOCK7	dedicator of	O	dedicator of	20210307
	ENSG00000196205		1015.14	-1.19	-7.77	7.75654E-15	1.23045E-11	126.122356	148.556989	151.57776	91.5964415	917.878446	1291.91947	1076.86152	1231.96285	284.947991	413.332738	227.46401	318.161465	1760.0176	2690.395731	2251.37124	3260.028441											
RPLP0	ENSG00000089157	6175	5065.88	-1.24	-7.75	9.2137E-15	1.34917E-11	5222.64659	5247.14858	3537.04812	3025.61528	1167.28165	2774.57822	1912.27257	2358.34164	8089.56717	8596.54904	7038.86647	9375.96726	3747.07294	6641.613386	5122.47233	7197.110704	L10E|LP0|P0	MIM:180510	12	12q24.23	ribosomal pro	protein-codir	RPLP0	ribosomal pro	O	60S acidic rib	20210404
EEF1G	ENSG00000254772	1937	5284.91	-1.18	-7.66	1.80645E-14	2.45626E-11	4352.69641	3760.64877	3913.11802	2965.37688	1288.83326	4326.54356	2515.02358	3233.04959	8586.49965	5836.88348	6832.7092	9702.80231	3759.45858	8106.070587	6653.81695	8725.024908	EF1G|GIG35	MIM:130593	11	11q12.3	eukaryotic tra	protein-codir	EEF1G	eukaryotic tra	O	elongation fa	20210417
RMRP	ENSG00000269900	6023	18125.95	-1.32	-7.45	9.54262E-14	1.21102E-10	11299.8181	11453.1346	16621.6758	21802.8006	2755.25288	9774.30595	7006.71642	6418.67568	25140.3229	35564.51	25272.5737	49701.7451	12406.5615	17390.74315	18237.6003	19168.7292	CHH|NME1|	MIM:157660	9	9p13.3	RNA compon	ncRNA	RMRP	RNA compon	O	-	20210410
	ENSG00000227766		200.50	-1.84	-7.29	3.17673E-13	3.77951E-10	93.9031817	129.354921	2.9278083	6.85604527	140.667436	75.4221393	93.939341	167.475993	284.999594	457.272712	28.2378516	26.1396252	455.957886	501.0075027	209.868493	534.0184318											
RPSA	ENSG00000168028	3921	3263.07	-1.09	-7.24	4.32842E-13	4.84656E-10	2721.62676	2421.39969	1835.46995	2036.19348	1196.43997	2363.6661	1795.43171	2125.36367	6892.13223	2932.62855	3345.525	5535.10706	2769.71653	4737.067568	4219.98827	5281.348438	37LRP|67LR|	MIM:150370	3	3p22.1	ribosomal pro	protein-codir	RPSA	ribosomal pro	O	40S ribosoma	20210418
LINC02511	ENSG00000248869	105377441	148.17	4.50	7.24	4.58279E-13	4.84656E-10	0	1.60629778	0	1.7823627	1079.1196	612.505382	269.29929	276.498893	0	0	0	0	106.870454	8.42199324	3.30529325	11.25165463	-	HGNC:HGNC:	4	4q28.3	long intergen	ncRNA	LINC02511	long intergen	O	-	20210302
VPS13A	ENSG00000197969	23230	2387.97	0.72	7.16	8.35281E-13	8.36863E-10	2793.379	2729.4348	3061.44804	2850.57684	3702.56092	2781.7279	3093.89321	2722.99505	1404.62694	1877.88824	1873.41011	1776.20262	1989.51329	1896.584054	2187.66287	1465.646587	CHAC|CHORE	MIM:605978	9	9q21.2	vacuolar prot	protein-codir	VPS13A	vacuolar prot	O	vacuolar prot	20210418
RPS9	ENSG00000170889	6203	1088.66	-0.98	-6.84	7.97307E-12	7.58877E-09	1461.80684	817.937627	651.983125	772.472503	428.556993	526.989473	539.125309	733.488686	1830.48214	1832.19622	1304.74039	2145.68969	770.563995	1018.22322	1122.3125	1462.031281	S9	MIM:603631	19	19q13.42	ribosomal pro	protein-codir	RPS9	ribosomal pro	O	40S ribosoma	20210404
RPL10A	ENSG00000198755	4736	2631.91	-0.87	-6.83	8.62264E-12	7.81622E-09	3030.15544	2384.75121	1634.72408	1826.6782	1042.15692	2063.80253	1356.08972	1543.44804	5213.13489	3242.12779	2822.16033	4937.47441	1891.77175	3317.447378	2707.54195	3097.07377	CSA19|Csa-1	MIM:615660	6	6p21.31	ribosomal pro	protein-codir	RPL10A	ribosomal pro	O	60S ribosoma	20210404
TRPC3	ENSG00000138741	7222	911.61	3.76	6.75	1.50222E-11	1.29983E-08	9.545	6.73565319	88.5369764	137.228696	6653.99361	3512.82768	1593.17216	1773.05827	3.42506156	20.8494344	5.02189342	8.86294187	587.157169	61.58591244	50.8670778	72.96821686	SCA41|TRP3	MIM:602345	4	4q27	transient rece	protein-codir	TRPC3	transient rece	O	short transien	20210329
SYNE1	ENSG00000131018	23345	8420.63	0.86	6.72	1.7932E-11	1.48415E-08	5600.45845	6648.57305	9828.01585	12207.0259	15321.8711	10163.9072	14870.51	12096.9557	2277.40434	5190.66796	6495.73423	5980.28772	9097.34937	5822.57701	6798.10721	6330.706829	8B|AMC3|AI	MIM:608441	6	6q25.2	spectrin repe	protein-codir	SYNE1	spectrin repe	O	nesprin-1|CP	20210408
PIK3IP1	ENSG00000100100	113791	1318.55	-1.07	-6.55	5.7427E-11	4.37272E-08	1128.96278	1070.10327	914.031833	801.643864	867.743142	896.715033	671.411317	531.955705	1369.49159	1836.79449	1861.90269	2243.71892	1807.85327	1800.858465	1899.14801	1394.541769	HGFL|TrIP|h	MIM:619158	22	22q12.2	phosphoinosi	protein-codir	PIK3IP1	phosphoinosi	O	phosphoinosi	20210302
KCNJ3	ENSG00000162989	3760	42.05	4.02	6.56	5.54166E-11	4.37272E-08	0	2.76600917	42.7607115	4.81544607	407.511253	113.917282	38.7984246	36.903059	0	0.72451942	1.43522455	0.6647488	14.2857705	2.087644024	2.77684536	3.300142395	GIRK1|KGA|	MIM:601534	2	2q24.1	potassium in	protein-codir	KCNJ3	potassium in	O	G protein-act	20210403
CBLB	ENSG00000114423	868	1801.88	0.81	6.51	7.45821E-11	5.46056E-08	1010.45739	1440.31914	4117.24768	2470.53202	2240.96969	2277.52501	1961.18738	3126.84537	732.833025	1073.00267	1751.95997	1501.2031	1123.8876	1173.771403	1135.03663	1693.302826	Cbl-b|Nbla00	MIM:604491	3	3q13.11	Cbl proto-onc	protein-codir	CBLB	Cbl proto-onc	O	E3 ubiquitin-	20210406
RPL13	ENSG00000167526	6137	9275.63	-0.95	-6.43	1.24516E-10	8.61573E-08	7563.78195	8704.68886	7494.92183	5771.70774	3269.10857	7735.59312	4987.34652	6033.58083	13947.9328	12436.9754	10043.0195	11915.3071	8890.22277	12940.05939	11727.2701	14948.53668	BBC1|D16S4	MIM:113703	16	16q24.3|17p	ribosomal pro	protein-codir	RPL13	ribosomal pro	O	60S ribosoma	20210404
RPS10	ENSG00000124614	6204	2907.84	-0.85	-6.43	1.31255E-10	8.61573E-08	2721.46247	2045.29893	1791.62729	1822.75706	1383.13719	2623.7051	1891.91573	2040.41665	6577.7258	2675.66097	2887.98044	4069.33339	2468.73408	3782.952728	3503.44184	4239.247813	DBA9|S10	MIM:603632	6	6p21.31	ribosomal pro	protein-codir	RPS10	ribosomal pro	O	40S ribosoma	20210410
SAMD3	ENSG00000164483	154075	1322.79	0.88	6.43	1.30932E-10	8.61573E-08	972.272323	1159.11087	2027.16635	1318.62785	2175.79468	1548.06862	2065.62271	2272.79351	367.0741	760.736584	1089.0816	767.717143	1367.7638	745.1083547	898.93939	1628.717016	-	HGNC:HGNC:	6	6q23.1	sterile alpha	protein-codir	SAMD3	sterile alpha	O	sterile alpha	20210302
EMX2	ENSG00000170370	2018	61.63	4.14	6.40	1.51721E-10	9.62723E-08	3.74830683	1.16148457	28.2797577	16.7430515	553.482557	180.979457	81.6914068	70.0249142	3.82645205	0	1.70830972	0	30.446971	2.955285111	4.59668116	6.475660564	-	MIM:600035	10	10q26.11	empty spirac	protein-codir	EMX2	empty spirac	O	homeobox pr	20210405
SNORA73A	ENSG00000274266	6080	563.53	-1.72	-6.39	1.6819E-10	1.0328E-07	1125.06191	577.769499	95.305812	268.887679	40.0579995	146.460779	99.3682379	217.799021	1329.90281	1748.61236	761.075618	993.323978	199.45826	339.6769261	446.147952	627.6363263	E1|E1-7|E1b	MIM:180645	1	1p35.3	small nucleol	snoRNA	SNORA73A	small nucleol	O	RNA, U17a sr	20210302
EEF1B2	ENSG00000114942	1933	1582.80	-1.02	-6.38	1.75607E-10	1.04464E-07	1499.45053	1544.19649	1097.59467	936.597971	452.432531	1272.99763	763.331701	965.133443	2787.1332	2044.97034	1969.37817	2887.08597	1150.41695	2043.857881	1962.32463	1947.880806	EEF1B|EEF1B	MIM:600655	2	2q33.3	eukaryotic tra	protein-codir	EEF1B2	eukaryotic tra	O	elongation fa	20210417
RPL13A	ENSG00000142541	23521	14879.24	-0.74	-6.29	3.19984E-10	1.84582E-07	12388.2943	13438.275	9099.61917	9722.8359	8235.19871	11564.8603	9976.1124	14366.238	27254.2302	16688.6929	13916.5301	19754.9566	14338.9881	17186.98094	15882.6858	24253.36581	L13A|TSTA1	MIM:619225	19	19q13.33	ribosomal pro	protein-codir	RPL13A	ribosomal pro	O	60S ribosoma	20210404
	ENSG00000273149		163.37	-3.84	-6.16	7.39952E-10	4.14286E-07	10.0148036	11.0219407	0	0	9.50465608	5.83442942	8.86850976	11.0107314	1245.57993	1071.77876	4.33004401	3.1122102	43.7324164	64.22358845	34.8745443	89.95475006											
EEF1A1	ENSG00000156508	1915	63317.52	-0.73	-6.14	8.16709E-10	4.44197E-07	56454.6577	61985.3163	51106.7168	50607.6657	30785.4811	47123.5705	38067.6001	43021.9933	97868.2443	77287.2003	73502.7018	111082.094	47781.8034	70246.30045	68946.5557	87212.36626	CCS-3|CCS3|	MIM:130590	6	6q13	eukaryotic tra	protein-codir	EEF1A1	eukaryotic tra	O	elongation fa	20210406
RPL8	ENSG00000161016	6132	3661.47	-0.81	-6.10	1.04998E-09	5.55205E-07	3709.32879	2782.67773	3358.34858	2983.4926	1239.71194	3025.07953	2180.97462	1971.72641	7676.14649	4148.62667	3985.71666	6756.12575	2493.12146	4577.290768	3895.54244	3799.638153	L8	MIM:604177	8	8q24.3	ribosomal pro	protein-codir	RPL8	ribosomal pro	O	60S ribosoma	20210404
	ENSG00000267731		335.37	1.06	6.09	1.11231E-09	5.7227E-07	201.597836	322.208273	418.972412	489.452029	590.896433	473.834844	654.988802	443.527581	59.779504	193.682856	263.55831	158.412587	287.04338	266.1916121	293.684663	248.0857525											
SPINT2	ENSG00000167642	10653	79.56	-2.85	-6.08	1.20896E-09	6.05628E-07	35.3875612	29.3246892	0.97863195	13.7381654	2.39028451	34.6444365	31.9583093	4.91010118	598.229985	98.4650979	45.3894027	83.7045624	35.1586888	131.0976163	73.9839997	53.63511106	DIAR3|HAI-2	MIM:605124	19	19q13.2	serine peptid	protein-codir	SPINT2	serine peptid	O	kunitz-type p	20210302
RCAN3	ENSG00000117602	11123	1660.05	-2.99	-6.07	1.29902E-09	6.18205E-07	2865.64062	1803.03791	107.538386	112.810668	105.775237	1154.18367	229.941457	79.4864963	2034.07004	2760.1296	1645.69456	3301.58427	1522.8182	3849.336454	3076.06942	1912.634947	DSCR1L2|MC	MIM:605860	1	1p36.11	RCAN family	protein-codir	RCAN3	RCAN family	O	calcipressin-3	20210418
VAV3	ENSG00000134215	10451	435.49	1.28	6.07	1.28855E-09	6.18205E-07	529.812163	285.335794	1602.98494	552.722128	646.855704	447.594021	555.669023	444.27284	237.95932	177.317721	323.903321	210.110182	284.301542	151.8129951	207.159613	310.1071207	-	MIM:605541	1	1p13.3	vav guanine r	protein-codir	VAV3	vav guanine r	O	guanine nucl	20210302
ZFC3H1	ENSG00000133858	196441	1742.91	0.83	6.03	1.65864E-09	7.70095E-07	2681.73099	1620.6238	2595.12988	2459.52102	2297.06756	2216.56123	2190.26097	1754.29603	971.571433	986.646021	1452.99308	1129.11723	1468.73289	1561.166048	1493.0128	1008.08011	CCDC131|CSI	HGNC:HGNC:	12	12q21.1	zinc finger C3	protein-codir	ZFC3H1	zinc finger C3	O	zinc finger C3	20210302
GOLGA8O	ENSG00000206127	728047	294.79	1.42	6.00	2.00342E-09	9.08027E-07	155.932036	294.159063	1120.34485	131.263042	1043.16462	145.650126	267.489858	271.594386	72.8438006	125.427953	223.176835	85.2368425	569.232128	51.1369785	101.986876	57.98697252	-	HGNC:HGNC:	15	15q13.3	golgin A8 fam	protein-codir	GOLGA8O	golgin A8 fam	O	golgin subfan	20210302
ANKRD36C	ENSG00000174501	400986	1259.83	0.92	5.95	2.63282E-09	1.16554E-06	1500.89499	834.393487	3106.29984	1359.51849	1888.51438	1691.08841	1520.3001	1439.7513	681.064937	639.047811	1029.928	699.920306	1143.14658	945.7388299	1017.54586	660.1962632	-	HGNC:HGNC:	2	2q11.1	ankyrin repea	protein-codir	ANKRD36C	ankyrin repea	O	ankyrin repea	20210325
	ENSG00000287387		51.20	-2.95	-5.91	3.34747E-09	1.44824E-06	8.8325319	26.8024228	0	0	11.4355418	10.6949281	2.62745522	6.7397577	184.692397	420.425914	4.26674124	2.06792357	32.7067727	53.29238501	19.4980216	35.1586658											
RPL3	ENSG00000100316	6122	9022.52	-0.81	-5.87	4.43201E-09	1.87484E-06	6448.85364	6827.42599	6528.76545	6690.55867	4445.9957	7644.42642	6005.32406	7240.95946	14297.8823	8473.88004	9107.725	15764.8302	7887.34071	11253.14464	10575.6438	15167.59823	ASC-1|L3|TA	MIM:604163	22	22q13.1	ribosomal pro	protein-codir	RPL3	ribosomal pro	O	60S ribosoma	20210418
	ENSG00000287510		231.14	-3.36	-5.86	4.63587E-09	1.91844E-06	50.1939121	35.5331342	0	11.7205019	46.9099279	1.65894133	16.5995785	19.932378	896.698294	2047.89553	5.64930139	10.7138991	138.908236	105.2295405	96.1504479	214.435604											
ZNF814	ENSG00000204514	730051	305.02	-1.71	-5.86	4.75166E-09	1.92452E-06	274.694239	170.924029	67.1570461	111.583983	33.4102259	82.2174848	253.232544	29.645972	2083.65422	275.686839	178.707902	287.380884	171.561084	277.0729019	405.098413	178.262779	-	HGNC:HGNC:	19	19q13.43	zinc finger pr	protein-codir	ZNF814	zinc finger pr	O	zinc finger pr	20210302
	ENSG00000269888		103.69	-3.32	-5.84	5.36471E-09	2.08413E-06	29.7173328	35.8923366	0	0	0	11.9343262	5.30523726	6.00232336	627.379633	817.308378	11.0479596	8.68839583	12.4747038	29.22264059	16.8294441	47.17181763											
GAB3	ENSG00000160219	139716	871.50	1.21	5.84	5.33888E-09	2.08413E-06	690.444741	980.785079	985.823394	1257.13013	1890.28459	1012.68341	1173.80329	1438.78703	117.619602	540.001231	594.87326	516.815559	1159.52466	361.6751713	513.469105	710.3379678	-	MIM:300482	X	Xq28	GRB2 associa	protein-codir	GAB3	GRB2 associa	O	GRB2-associa	20210302
MAL	ENSG00000172005	4118	140.78	-3.68	-5.82	5.7849E-09	2.20243E-06	159.319883	156.406515	0	7.70391817	3.46978697	53.3257826	3.83646813	1.11487101	1019.59532	129.803823	83.2778526	209.416451	68.7865022	173.0026651	120.032881	63.38381165	MVP17|VIP1	MIM:188860	2	2q11.1	mal, T cell dif	protein-codir	MAL	mal, T cell dif	O	myelin and ly	20210302
	ENSG00000271581		212.78	-2.12	-5.80	6.47193E-09	2.36922E-06	49.5335478	158.302151	0	4.9792792	111.496749	31.977171	138.520199	260.073942	210.603472	602.959959	25.2346372	23.2436432	271.864777	497.0118249	443.725461	574.9018859											
SEMA3E	ENSG00000170381	9723	22.64	4.76	5.80	6.46288E-09	2.36922E-06	0	7.60479648	48.8614161	14.3412896	157.764629	66.5393526	29.5645411	23.8952377	0	0.7339674	0.73048839	0	8.35931785	0.680003762	0	3.239785124	M-SEMAH|M	MIM:608166	7	7q21.11	semaphorin 3	protein-codir	SEMA3E	semaphorin 3	O	semaphorin-3	20210410
RPS14	ENSG00000164587	6208	3879.68	-0.85	-5.79	6.91407E-09	2.48332E-06	3135.97969	3242.08634	2803.38754	2236.20276	1635.15028	3236.511	2726.95499	3151.62454	4631.68392	3895.16952	4080.19537	5633.09625	3725.16058	5812.135709	5534.58526	6594.96321	EMTB|S14	MIM:130620	5	5q33.1	ribosomal pro	protein-codir	RPS14	ribosomal pro	O	40S ribosoma	20210404
	ENSG00000270141		44.32	-4.54	-5.77	7.99316E-09	2.81774E-06	18.889349	28.8401959	0	3.2988828	0	10.3278074	0	0	162.604186	101.495322	34.3661935	66.3990519	48.579831	84.83486481	141.853579	7.648781383											
KLRD1	ENSG00000134539	3824	6508.63	1.31	5.74	9.69529E-09	3.29725E-06	3740.09898	5959.69114	15096.136	7637.06688	8422.77093	9079.39943	10259.5211	12303.3299	842.053225	4737.89184	3834.52182	3600.06068	4942.92713	2766.12618	2815.01397	8101.436264	CD94	MIM:602894	12	12p13.2	killer cell lect	protein-codir	KLRD1	killer cell lect	O	natural killer	20210302
SCARNA12	ENSG00000238795	677777	116.64	-1.71	-5.72	1.05758E-08	3.53193E-06	94.6134656	169.001934	40.4076263	48.2326679	31.638122	18.5000134	25.6976559	54.1996596	172.69158	294.619134	144.167337	282.452224	67.832067	120.0856566	114.198806	187.9795882	U89	MIM:615642	12	12p13.31	small Cajal bo	ncRNA	SCARNA12	small Cajal bo	O	U89 small nu	20210302
	ENSG00000259001		3553.53	-1.03	-5.69	1.28379E-08	4.14206E-06	1732.46316	1864.29218	389.273266	2125.86102	2144.09644	3488.67643	3295.41911	4856.91428	3555.12723	5414.83126	1381.47057	3406.47111	5240.87095	4951.702528	6615.67187	6393.319487											
NCK2	ENSG00000071051	8440	446.57	-0.88	-5.69	1.26358E-08	4.14206E-06	563.99653	419.02789	242.858998	245.200217	189.133279	393.199572	289.617534	240.635891	747.515893	583.955175	459.694001	511.40604	494.017289	666.087322	583.427744	515.3918823	GRB4|NCKbe	MIM:604930	2	2q12.2	NCK adaptor	protein-codir	NCK2	NCK adaptor	O	cytoplasmic p	20210320
	ENSG00000264577		58.49	-3.46	-5.67	1.4578E-08	4.62511E-06	3.88971609	15.7601319	0	0	9.002987	3.1910473	3.21719196	7.60991129	269.519274	475.82971	0.76695094	2.15664471	28.7077537	33.72398097	28.3060864	54.16934485											
UTRN	ENSG00000152818	7402	6361.77	0.64	5.65	1.64191E-08	5.12384E-06	8448.06891	6854.65879	15005.7008	8018.31273	6531.75837	5530.63579	5848.39758	6591.04357	5744.39882	4951.39023	6522.53293	4900.85809	4451.32482	3798.37359	4281.90062	4308.976999	DMDL|DRP|I	MIM:128240	6	6q24.2	utrophin	protein-codir	UTRN	utrophin	O	utrophin|dys	20210305
LOC1001299	ENSG00000245748	100129931	27.65	-3.75	-5.62	1.89882E-08	5.82998E-06	20.554442	8.66266451	0	0	0	1.69978552	0	1.93767639	188.835491	128.260543	2.58530198	0	23.4971321	22.02003525	18.9712086	25.33347533	-	Ensembl:ENS	4	4p16.1	uncharacteriz	ncRNA	-	-	-	-	20210302
EPPK1	ENSG00000261150	83481	40.17	-4.59	-5.58	2.41829E-08	7.30491E-06	0	37.0727895	0	3.00817891	0	22.6677239	0	6.72341417	18.0805855	114.068245	42.7355054	76.2661352	114.132236	85.38941653	65.84935	56.7340016	EPIPL|EPIPL1	MIM:607553	8	8q24.3	epiplakin 1	protein-codir	EPPK1	epiplakin 1	O	epiplakin|450	20210302
CXCR5	ENSG00000160683	643	79.02	-2.88	-5.58	2.45595E-08	7.30491E-06	18.9347936	16.4849141	0	0	41.7062107	14.5012309	20.2885023	29.2331735	301.057809	431.588977	12.2841243	9.29345068	126.452725	91.32046197	71.3448388	79.81711415	BLR1|CD185	MIM:601613	11	11q23.3	C-X-C motif c	protein-codir	CXCR5	C-X-C motif c	O	C-X-C chemol	20210307
SLAMF7	ENSG00000026751	57823	1492.28	0.91	5.54	3.08184E-08	9.02554E-06	1131.61511	1542.9729	3040.23831	2069.92993	1259.72858	1931.66182	2173.39512	2352.92684	565.594339	1080.38408	1721.99378	1051.28075	956.839518	747.0182569	712.986008	1537.983776	19A|CD319|	MIM:606625	1	1q23.3	SLAM family	protein-codir	SLAMF7	SLAM family	O	SLAM family	20210302
RPS2	ENSG00000140988	6187	3914.88	-0.92	-5.53	3.1618E-08	9.11939E-06	3341.84802	2927.22724	3197.89158	2762.00345	1257.56759	3696.84432	2180.25604	2425.47048	8320.75942	4683.91639	3535.0975	5771.17638	3385.9217	5211.734239	4422.08181	5518.218938	LLREP3|S2	MIM:603624	16	16p13.3	ribosomal pro	protein-codir	RPS2	ribosomal pro	O	40S ribosoma	20210404
THOC2	ENSG00000125676	57187	1212.32	0.73	5.52	3.4547E-08	9.81547E-06	1082.23091	755.136706	2183.12801	2003.79984	2002.35594	1619.44097	1367.44238	1128.41913	674.042344	668.180943	1233.54717	1430.42155	977.951659	853.0500792	854.220785	563.6958349	CXorf3|MRX	MIM:300395	X	Xq25	THO complex	protein-codir	THOC2	THO complex	O	THO complex	20210307
FCGBP	ENSG00000275395	8857	565.40	-1.70	-5.51	3.52282E-08	9.86182E-06	413.896012	532.497596	114.132671	235.991973	212.581998	314.54205	222.414246	176.736853	836.41746	373.516455	500.407646	1361.54428	948.065102	986.8090629	1123.09761	693.6765554	FC(GAMMA)B	MIM:617553	19	19q13.2	Fc fragment o	protein-codir	FCGBP	Fc fragment o	O	IgGFc-binding	20210302
PRPF38B	ENSG00000134186	55119	2125.39	0.71	5.51	3.67378E-08	1.01354E-05	1574.78755	2175.55407	2862.74823	3286.37331	3424.12342	2761.49745	2488.5368	2470.74355	1069.4176	1456.25061	2360.1481	1540.36699	2130.28672	1728.771504	1585.94845	1090.627411	NET1	HGNC:HGNC:	1	1p13.3	pre-mRNA pr	protein-codir	PRPF38B	pre-mRNA pr	O	pre-mRNA-sp	20210302
CMTM7	ENSG00000153551	112616	160.77	-1.25	-5.50	3.85873E-08	1.03457E-05	262.235231	163.883431	33.6939503	71.2059778	44.5162708	68.515126	36.2877454	73.1293229	757.470599	225.534794	143.914547	167.775917	111.097789	183.4093864	105.583478	124.0385346	CKLFSF7	MIM:607890	3	3p22.3	CKLF like MA	protein-codir	CMTM7	CKLF like MA	O	CKLF-like MA	20210302
CEP78	ENSG00000148019	84131	1376.32	0.94	5.50	3.82809E-08	1.03457E-05	878.963357	1120.10914	2344.76497	2848.52582	1939.20403	1617.35469	2482.40524	1347.49439	500.035627	950.336039	1464.71927	1218.15431	1130.80409	743.1127528	733.122697	702.0112873	C9orf81|CRD	MIM:617110	9	9q21.2	centrosomal	protein-codir	CEP78	centrosomal	O	centrosomal	20210302
SNORD58A	ENSG00000206602	26791	40.65	-3.23	-5.46	4.63261E-08	1.22481E-05	8.26017691	3.60259862	0	0	0	18.4467427	8.06192332	34.1412575	21.8207672	72.1225471	4.93783999	3.85088233	67.4228736	98.30188912	101.478239	207.996992	RNU58A|U58	HGNC:HGNC:	18	18q21.1	small nucleol	snoRNA	SNORD58A	small nucleol	O	RNA, U58A sr	20210302
C14orf132	ENSG00000227051	56967	63.73	-3.38	-5.45	5.02041E-08	1.30829E-05	64.4377064	80.3875079	0	1.33688711	0	30.9496171	2.20092524	1.02156838	423.435917	98.7631354	12.4212465	56.6208029	39.3699143	142.5491141	19.0271653	47.09008061	C14orf88	HGNC:HGNC:	14	14q32.2	chromosome	protein-codir	C14orf132	chromosome	O	uncharacteriz	20210302
RACK1	ENSG00000204628	10399	4990.82	-0.76	-5.45	5.0858E-08	1.30829E-05	4590.57751	3679.06655	3359.6074	3692.51587	2408.3331	4254.47954	3264.47387	4171.72332	5685.50221	5143.81912	5533.16215	10043.4809	4315.55535	6465.678652	5599.88041	7645.201792	GNB2L1|Gnb	MIM:176981	5	5q35.3	receptor for a	protein-codir	RACK1	receptor for a	O	receptor of a	20210404
FGF23	ENSG00000118972	8074	19.47	5.12	5.42	5.86365E-08	1.48827E-05	2.50868034	0	93.4203179	10.0773946	152.804919	28.0090742	9.41896559	7.4896202	2.16049317	0	1.36435965	0	4.31017854	0	0	0	ADHR|FGFN|	MIM:605380	12	12p13.32	fibroblast gro	protein-codir	FGF23	fibroblast gro	O	fibroblast gro	20210418
	ENSG00000256128		339.21	1.39	5.41	6.21655E-08	1.55708E-05	150.367021	187.085833	1210.82839	605.660187	392.355174	489.175038	503.698472	453.670475	44.1641394	131.082346	226.67596	197.163655	203.137595	231.5960528	107.624861	293.1461883											
GPM6A	ENSG00000150625	2823	19.40	3.92	5.40	6.73654E-08	1.66541E-05	21.8250922	4.1710278	136.424346	67.3451556	34.1075891	15.4630659	3.82487084	10.2240006	0	1.57300189	7.20872953	2.03485419	3.8551459	0	0	2.419682096	GPM6|M6A	MIM:601275	4	4q34.2	glycoprotein	protein-codir	GPM6A	glycoprotein	O	neuronal mei	20210406
LOC1001284	ENSG00000251143	100128494	92.52	-1.83	-5.39	7.23993E-08	1.76691E-05	91.0280781	75.8088743	0	8.30005868	17.4623388	56.8122746	41.4463375	46.6115562	222.477948	317.423021	33.6983677	14.3882137	115.441575	152.0337166	162.263665	125.1712997	-	Ensembl:ENS	11	11q13.4	uncharacteriz	ncRNA	-	-	-	-	20210302
RPS16	ENSG00000105193	6217	4451.65	-0.77	-5.38	7.53322E-08	1.81522E-05	3525.65723	2748.2027	5378.13716	4210.52307	1796.0504	2918.38997	2765.48609	2962.23046	7120.44679	3842.32928	5569.52071	9211.27431	3343.4691	4834.875383	4898.52052	6101.272188	S16	MIM:603675	19	19q13.2	ribosomal pro	protein-codir	RPS16	ribosomal pro	O	40S ribosoma	20210404
CLN5	ENSG00000102805	1203	214.13	-1.71	-5.37	7.88082E-08	1.87524E-05	355.527421	256.126682	38.084932	68.4354641	39.5284427	105.53643	47.3474027	50.4796094	401.032131	533.323999	212.716969	269.468414	214.329107	403.9559765	335.340323	94.85303192	-	MIM:608102	13	13q22.3	CLN5 intracel	protein-codir	CLN5	CLN5 intracel	O	ceroid-lipofu	20210410
USP6NL	ENSG00000148429	9712	89.16	-2.64	-5.32	1.03782E-07	2.439E-05	91.8308945	46.5501884	15.2375191	3.09893633	0	19.0838764	14.0235691	4.83367439	816.228481	50.5626253	72.029757	60.7518009	55.4971838	68.42508298	80.3813988	28.04806178	RNTRE|TRE2	MIM:605405	10	10p14	USP6 N-term	protein-codir	USP6NL	USP6 N-term	O	USP6 N-term	20210305
RPL18	ENSG00000063177	6141	2092.49	-0.80	-5.32	1.05741E-07	2.45474E-05	1758.74694	1769.21651	1655.34418	1257.18336	916.70063	1701.81769	1459.28233	1594.38896	4084.3384	2495.30165	1714.4235	2127.00978	1975.94816	3036.967326	2826.53261	3106.597889	DBA18|L18	MIM:604179	19	19q13.33	ribosomal pro	protein-codir	RPL18	ribosomal pro	O	60S ribosoma	20210410
VIPR1	ENSG00000114812	7433	144.34	-3.35	-5.30	1.17439E-07	2.69345E-05	346.947675	122.822706	0	5.02012331	16.88306	84.4960565	15.5411506	3.53087734	235.201528	175.947633	178.320152	144.56826	228.016375	330.469473	311.717849	109.9285218	HVR1|II|PAC	MIM:192321	3	3p22.1	vasoactive in	protein-codir	VIPR1	vasoactive in	O	vasoactive in	20210302
TRGC1	ENSG00000211689	6966	624.96	0.91	5.29	1.21217E-07	2.74701E-05	117.892586	230.481555	2635.46506	553.369752	1041.16385	347.174315	667.061288	1237.20407	85.3464099	181.024007	909.829743	227.866396	646.308008	214.2511205	340.206895	564.6958564	C1|TCRG|TC	MIM:186970	7	7p14.1	T cell recepto	other	TRGC1	T cell recepto	O	T-cell recepto	20210302
SYPL1	ENSG00000008282	6856	256.13	-1.22	-5.28	1.29532E-07	2.9009E-05	230.558116	293.471963	173.784949	124.188041	53.2714689	185.086506	100.367541	110.517227	615.945577	315.539269	315.288715	438.055687	157.708387	359.3093471	364.239632	260.6817275	H-SP1|SYPL	MIM:616665	7	7q22.3	synaptophysi	protein-codir	SYPL1	synaptophysi	O	synaptophysi	20210417
EMP2	ENSG00000213853	2013	25.34	4.23	5.27	1.38648E-07	3.03368E-05	1.85676092	5.55536883	58.1874977	16.2046016	158.447141	85.210129	28.3209547	28.104819	6.18119992	0	1.96599491	0.63326927	11.0251277	0.737292084	2.20668313	0.834474288	XMP	MIM:602334	16	16p13.13	epithelial me	protein-codir	EMP2	epithelial me	O	epithelial me	20210302
VPS13C	ENSG00000129003	54832	5029.02	0.65	5.25	1.53809E-07	3.32716E-05	5766.43592	5155.44483	6016.42917	7612.25616	6421.64654	7312.5425	5688.68648	5054.34356	2536.28662	3667.70612	4748.64642	4523.2687	4609.09563	3894.456128	4258.21361	3198.871603	PARK23	MIM:608879	15	15q22.2	vacuolar prot	protein-codir	VPS13C	vacuolar prot	O	vacuolar prot	20210418
SH3D19	ENSG00000109686	152503	252.48	-3.64	-5.24	1.58845E-07	3.39749E-05	48.2600083	31.0067241	61.3204358	0.32357063	0	2.7018449	6.71457561	10.3357918	1706.39483	1973.80796	10.0359112	3.31988512	42.3833801	50.85502007	42.027211	50.17236357	EBP|EVE1|Ev	MIM:608674	4	4q31.3	SH3 domain c	protein-codir	SH3D19	SH3 domain c	O	SH3 domain-	20210327
ARID5B	ENSG00000150347	84159	897.62	0.72	5.21	1.84056E-07	3.89299E-05	1105.57107	973.756327	1690.59135	878.101282	1007.38429	1335.57685	775.767954	1204.63388	575.199155	607.430479	732.242566	791.242194	589.43771	808.2173844	465.302044	821.4713278	DESRT|MRF-	MIM:608538	10	10q21.2	AT-rich intera	protein-codir	ARID5B	AT-rich intera	O	AT-rich intera	20210302
	ENSG00000279605		32.69	-2.49	-5.21	1.9038E-07	3.92909E-05	15.1598942	13.6965367	0	1.55975261	9.41711158	5.25799675	11.0031839	12.6899272	111.878812	159.540631	2.09712207	2.69401988	37.6419638	45.00477249	37.2744694	58.18063259											
RPL27A	ENSG00000166441	6157	3315.45	-0.69	-5.21	1.91955E-07	3.92909E-05	2603.70385	2728.30381	2923.16201	3247.78596	1135.85426	2799.8195	2557.77411	2287.48371	4897.53516	4587.98147	4651.37679	6045.12748	2274.03906	3054.965521	3203.51266	4048.710996	L27A	MIM:603637	11	11p15.4	ribosomal pro	protein-codir	RPL27A	ribosomal pro	O	60S ribosoma	20210404

RBM39	ENSG00000131051	9584	3728.80	0.64	5.21	1.90754E-07	3.92909E-05	4398.81284	3667.83495	9200.88316	3933.38612	4084.41491	3999.05557	3948.15589	3637.70825	2375.04882	2805.37525	4019.96239	2863.26473	2826.94991	2760.332684	2917.65536	2222.004058	CAPER|CAPE	MIM:604739	20	20q11.22	RNA binding	protein-codir	RBM39	RNA binding	O	RNA-binding	20210404
SLC7A11	ENSG00000151012	23657	37.39	-4.58	-5.20	1.96693E-07	3.98324E-05	0	23.4481929	3.19461615	0	16.2216326	0	0	1.18659117	31.6284416	27.5904423	103.361458	52.545192	317.105178	9.038338625	5.20195451	7.773286022	CCBR1|xCT	MIM:607933	4	4q28.3	solute carrier	protein-codir	SLC7A11	solute carrier	O	cystine/gluta	20210418
RPS5	ENSG00000083845	6193	1818.52	-1.14	-5.20	2.02712E-07	4.06192E-05	1860.51322	1984.51737	1650.35559	1005.12294	253.203106	1267.30914	748.716588	880.571505	3795.11059	2491.83234	2132.78617	3515.29812	1108.62472	2162.133797	1770.43963	2469.78479	S5	MIM:603630	19	19q13.43	ribosomal pr	protein-codir	RPS5	ribosomal pr	O	40S ribosoma	20210404
IL24	ENSG00000162892	11009	30.60	-3.98	-5.19	2.1099E-07	4.09192E-05	3.05774022	12.9725238	0	0	0	0	3.43014165	1.86824459	169.647154	204.918969	1.52313127	0	21.0125687	23.04514008	19.4580019	28.62109881	C49A|FISP|IL	MIM:604136	1	1q32.1	interleukin 2	protein-codir	IL24	interleukin 2	O	interleukin-2	20210417
IFNGR2	ENSG00000159128	3460	77.75	-3.19	-5.19	2.08465E-07	4.09192E-05	104.201183	16.0432887	0	3.30199605	11.4077501	18.5940858	1.32483754	0	790.77753	46.006019	32.9179118	49.1934322	54.8581922	54.75081046	44.3942125	16.26992294	AF-1|IFGR2|I	MIM:147569	21	21q22.11	interferon ga	protein-codir	IFNGR2	interferon ga	O	interferon ga	20210302
RPS2P5	ENSG00000240342	100130562	136.06	-1.90	-5.19	2.12504E-07	4.09192E-05	85.0711655	98.0568704	59.8661484	9.8570238	9.12167613	72.9095927	34.1251272	97.7298629	236.689253	466.808939	46.6681625	64.3245138	120.245838	223.8705251	156.486281	395.1918541	RPS2_20_12	HGNC:HGNC:	12	12q24.23	ribosomal pr	pseudo	RPS2P5	ribosomal pr	O	-	20210302
ZCCHC8	ENSG00000033030	55596	350.71	0.77	5.19	2.12807E-07	4.09192E-05	352.16851	300.429916	586.749596	497.728494	408.430408	527.134149	398.877919	450.355364	139.71826	203.951822	375.343777	266.239616	317.662408	283.7869712	266.695676	236.1473477	PFBMFT5	MIM:616381	12	12q24.31	zinc finger CC	protein-codir	ZCCHC8	zinc finger CC	O	zinc finger CC	20210302
RPL15	ENSG00000174748	6138	5933.50	-0.83	-5.18	2.22219E-07	4.22236E-05	7086.62729	5704.40345	4227.1541	3230.94553	2309.99527	4190.02068	3836.51367	3303.46376	15204.9374	7589.54252	4640.14689	7292.0387	4706.77954	6863.671438	6140.08444	8609.608723	DBA12|EC45	MIM:604174	3	3p24.2	ribosomal pr	protein-codir	RPL15	ribosomal pr	O	60S ribosoma	20210410
GREM1	ENSG00000166923	26585	25.61	3.84	5.18	2.24028E-07	4.22236E-05	5.8923321	7.88261708	65.0390463	14.3890367	192.080174	63.3413451	22.9001808	16.2043931	1.90613941	1.30592793	3.22138557	3.58355017	6.82755892	0	0.59605608	4.654292304	C15DUPq|CK	MIM:603054	15	15q13.3	gremlin 1, DA	protein-codir	GREM1	gremlin 1, DA	O	gremlin-1|DA	20210417
IQGAP1	ENSG00000140575	8826	5053.63	0.63	5.18	2.27352E-07	4.24301E-05	4778.52965	6762.20797	9870.23133	4966.72028	6510.80671	4564.1052	5163.1284	6899.93187	3622.26956	4203.56051	4091.18305	3364.82747	4771.8424	3206.974538	3332.99228	4748.715654	HUMORFA01	MIM:603379	15	15q26.1	IQ motif cont	protein-codir	IQGAP1	IQ motif cont	O	ras GTPase-a	20210418
ITGAM	ENSG00000169896	3684	210.26	1.77	5.16	2.47853E-07	4.58071E-05	418.170582	247.71669	509.387445	141.253204	172.036941	267.567569	432.609023	431.449422	37.4780811	84.0028744	119.755289	117.528018	124.892997	77.971439	68.0797137	114.2233962	CD11B|CR3A	MIM:120980	16	16p11.2	integrin subu	protein-codir	ITGAM	integrin subu	O	integrin alpha	20210404
	ENSG00000258017		139.31	-2.82	-5.14	2.69839E-07	4.93908E-05	67.5786008	20.5733563	0	1.97991885	50.8418969	5.63256681	11.094809	11.0103231	1254.19827	585.795087	11.0781729	6.93363805	41.302947	54.07083012	38.7042841	68.18042925											
H4C2	ENSG00000278705	8366	52.40	-2.06	-5.14	2.75422E-07	4.94617E-05	28.3311881	19.1108099	0	8.72508085	25.661858	33.0673146	29.5686015	28.5371579	72.6035036	109.977597	54.8174511	91.7566109	67.8639721	78.06546716	75.3583928	114.9276098	H4-16|H4/I|I	MIM:602829	6	6p22.2	H4 clustered	protein-codir	H4C2	H4 clustered	O	histone H4|H	20210302
MFNG	ENSG00000100060	4242	352.57	-1.26	-5.14	2.75078E-07	4.94617E-05	174.312886	125.205627	297.886301	142.125367	141.482638	267.17926	216.436855	288.440796	221.696895	412.618392	433.694222	592.837965	428.613248	603.5235477	845.587332	449.5061189	-	MIM:602577	22	22q13.1	MFNG O-fucc	protein-codir	MFNG	MFNG O-fucc	O	beta-1,3-N-ac	20210302
	ENSG00000273329		219.11	0.84	5.07	3.88444E-07	6.84669E-05	174.468539	170.261581	368.71081	403.080444	470.003489	227.483434	270.654509	184.600895	90.4408927	93.7027165	173.447707	170.920248	246.923428	180.54549	149.398054	131.1162302											
RPS12	ENSG00000112306	6206	5409.89	-0.90	-5.06	4.08937E-07	7.14177E-05	4737.49464	4987.27893	3947.961	3700.95199	2024.92756	3802.43014	3025.12795	3637.2379	10058.1326	4174.75007	7629.17873	9599.55312	3955.48697	6710.354956	6357.58749	8209.764091	S12	MIM:603660	6	6q23.2	ribosomal pr	protein-codir	RPS12	ribosomal pr	O	40S ribosoma	20210404
RPS3A	ENSG00000145425	6189	9056.77	-0.68	-5.06	4.28783E-07	7.42028E-05	7825.58279	8646.60939	6295.99012	6834.91652	5069.52803	7978.81417	6258.15856	6593.19641	11659.6384	9407.99635	9082.37722	15269.5027	7983.74314	11452.73341	12103.9276	12445.6299	FTE1|MFTL|S	MIM:180478	4	4q31.3	ribosomal pr	protein-codir	RPS3A	ribosomal pr	O	40S ribosoma	20210404
PRRG3	ENSG00000130032	79057	195.69	5.08	5.04	4.54332E-07	7.79159E-05	0.72280549	4.31406947	43.3176438	7.41461244	2728.95486	192.800512	69.6801396	43.2860978	7.40150569	2.08226178	0.58878209	0	26.6379418	0.725504525	0.72244023	2.445198946	PRGP3|TMG	MIM:300685	X	Xq28	proline rich a	protein-codir	PRRG3	proline rich a	O	transmembra	20210302
CEP350	ENSG00000135837	9857	3524.99	0.62	5.03	4.82148E-07	8.1948E-05	4712.41215	5150.89558	3857.58751	4268.09938	4567.93411	4043.74121	3706.22615	3796.32647	2042.91504	3243.32195	2373.86795	2538.01939	3658.72218	2922.181892	2801.25623	2716.294981	CAP350|GM	MIM:617870	1	1q25.2	centrosomal	protein-codir	CEP350	centrosomal	O	centrosome-a	20210302
	ENSG00000245970		57.72	-3.95	-5.03	5.02525E-07	8.46554E-05	11.749066	12.3152379	0	0	2.14562046	0	0	3.09427827	401.955803	423.122625	0	0	12.3234698	21.75688981	13.0699675	21.98905522											
	ENSG00000262160		67.40	-4.61	-5.02	5.11907E-07	8.54794E-05	6.26455018	20.1737075	6.41202639	0	0	0	0	1.79273577	467.27546	489.30971	1.45118679	1.34444554	30.301554	16.82079184	16.8468419	20.35199531											
RPS18	ENSG00000231500	6222	5702.83	-0.79	-5.02	5.29561E-07	8.76585E-05	5589.86021	5093.5758	3448.97138	3812.80804	2259.04206	5053.40962	3587.45514	4291.38778	10972.9457	5811.33416	3869.02415	8880.26612	4810.95984	7895.815936	7249.39204	8619.023588	D6S218E|HK	MIM:180473	6	6p21.32	ribosomal pr	protein-codir	RPS18	ribosomal pr	O	40S ribosoma	20210404
CKAP5	ENSG00000175216	9793	835.63	1.33	5.01	5.44319E-07	8.87338E-05	830.958721	511.368513	3570.43681	925.775995	1060.35334	646.793254	1435.21162	983.707711	231.96536	473.136293	542.211164	543.425888	416.214828	346.1683617	360.9733	491.3853214	CHTOG|MSP	MIM:611142	11	11p11.2	cytoskeleton	protein-codir	CKAP5	cytoskeleton	O	cytoskeleton-	20210404
NOP53	ENSG00000105373	29997	2476.45	-0.62	-5.00	5.60875E-07	9.04816E-05	3278.78002	2762.73218	1793.23897	2161.81984	1154.62239	1443.91723	1621.1033	1711.51552	3472.19611	4002.35156	2444.72239	3315.44289	2073.48074	2744.07756	2566.94385	3076.226512	GLTSCR2|PIC	MIM:605691	19	19q13.33	NOP53 ribosc	protein-codir	NOP53	NOP53 ribosc	O	ribosome bio	20210302
MAN1C1	ENSG00000117643	57134	228.78	-2.22	-4.99	5.97658E-07	9.56052E-05	236.464576	264.055135	1.70563623	83.082714	32.2684734	180.743735	87.5858025	31.1982396	511.283683	281.630351	147.127254	349.854403	250.46969	442.7387782	492.031228	268.2213528	HMIC|MAN1	MIM:616772	1	1p36.11	mannosidase	protein-codir	MAN1C1	mannosidase	O	mannosyl-oli	20210302
KLRF1	ENSG00000150045	51348	239.01	1.25	4.98	6.22688E-07	9.87791E-05	239.90282	99.9357155	840.697372	57.2345215	363.360303	264.324489	508.199718	270.811359	76.2745751	71.8487582	463.452072	25.3896225	181.417893	53.79396742	132.470159	175.1167176	CLEC5C|NKp	MIM:605029	12	12p13.31	killer cell lect	protein-codir	KLRF1	killer cell lect	O	killer cell lect	20210302
ELAVL1	ENSG00000066044	1994	324.36	-0.67	-4.98	6.42929E-07	0.000101147	243.997674	242.98114	213.693241	253.842182	177.492647	224.996834	290.985348	365.217549	334.843234	441.473997	371.396103	401.593839	292.738236	417.0118424	484.024242	433.5165101	ELAV1|HUR|	MIM:603466	19	19p13.2	ELAV like RN	protein-codir	ELAVL1	ELAV like RN	O	ELAV-like pro	20210418
SLC25A6	ENSG00000169100	293	802.86	-0.78	-4.96	7.01975E-07	0.000109531	640.206988	562.251904	444.223748	702.909347	535.32317	579.809022	525.149344	646.581591	1708.27948	1271.37693	769.595701	1179.72686	674.449959	938.0653398	826.069862	841.6904908	AAC3|ANT|A	MIM:300151	X|Y	X;Y	solute carrier	protein-codir	SLC25A6	solute carrier	O	ADP/ATP trar	20210404
LYZ	ENSG00000090382	4069	413.81	-2.61	-4.95	7.4702E-07	0.00011468	237.583913	80.6536679	48.5991972	18.3137365	126.837087	6.45089499	4.47139598	0	4820.15931	62.1398595	69.0992761	95.8702712	944.611399	28.14599206	43.5747894	34.464922	LYZF1|LZM	MIM:153450	12	12q15	lysozyme	protein-codir	LYZ	lysozyme	O	lysozyme C|1	20210302
TKT	ENSG00000163931	7086	297.86	-1.20	-4.94	7.76511E-07	0.000117315	256.876252	259.056676	139.701346	84.7944805	120.182299	159.340862	251.936529	207.634619	444.366901	470.316465	262.6398	505.075739	373.288405	353.549718	341.646292	535.2942596	HEL-S-48|HE	MIM:606781	3	3p21.1	transketolase	protein-codir	TKT	transketolase	O	transketolase	20210404
RAP1GAP2	ENSG00000132359	23108	135.23	1.44	4.92	8.68251E-07	0.000130142	62.4891616	128.738729	161.880831	195.482864	211.222752	211.266614	385.847524	222.525977	28.010391	126.94564	71.4331524	41.504995	111.347345	63.54719803	81.2849824	60.19764533	GARNL4|RAP	MIM:618714	17	17p13.3	RAP1 GTPase	protein-codir	RAP1GAP2	RAP1 GTPase	O	rap1 GTPase-	20210302
RN7SL1	ENSG00000276168	6029	39689.48	-1.27	-4.91	9.10663E-07	0.000135433	30176.6538	24520.376	58482.0533	30155.0432	4055.55012	9013.62193	11375.8479	10731.9234	82103.9036	137133.975	44786.5658	114174.065	9827.86411	15745.02588	24480.4297	28268.84193	7L1a|7SL|RN	MIM:612177	14	14q21.3	RNA compon	scRNA	RN7SL1	RNA compon	O	RNA, 7SL, cyt	20210302
JAKMIP2	ENSG00000176049	9832	295.27	1.19	4.91	9.20164E-07	0.000135785	125.495067	167.066888	1223.4153	404.87064	379.065012	250.351386	395.932137	447.833259	73.4541026	139.618104	271.662209	144.773457	226.149442	77.47643679	128.438844	268.7313833	JAMIP2|NEC	MIM:611197	5	5q32	janus kinase	protein-codir	JAKMIP2	janus kinase	O	janus kinase	20210417
ZACN	ENSG00000186919	353174	20.61	-4.04	-4.91	9.33496E-07	0.000136693	0.93459523	15.3484498	0	0	0	0	4.58554895	5.34227895	75.6166357	109.491924	6.81431363	4.01237985	25.9018183	33.1740179	20.220758	28.32574381	L2|LGICZ|LG	MIM:610935	17	17q25.1	zinc activatec	protein-codir	ZACN	zinc activatec	O	zinc-activatec	20210302
KLRC4	ENSG00000183542	8302	347.01	1.10	4.90	9.81146E-07	0.000142573	43.5344339	223.897785	1181.02054	864.668555	254.813456	788.963004	374.741248	184.560093	61.0188526	137.82093	474.161676	361.676559	107.134077	287.7871253	142.377003	63.95415525	NKG2-F|NKG	MIM:602893	12	12p13.2	killer cell lect	protein-codir	KLRC4	killer cell lect	O	NKG2-F type	20210302
CA6	ENSG00000131686	765	60.57	-4.13	-4.87	1.13759E-06	0.000162821	35.259841	15.7221621	0	0	0	0	0	1.06128096	838.330294	15.446232	5.07755537	15.1144888	8.79719086	9.200637289	11.9778583	13.08896881	CA-VI|GUSTI	MIM:114780	1	1p36.23	carbonic anh	protein-codir	CA6	carbonic anh	O	carbonic anh	20210302
DENND5A	ENSG00000184014	23258	326.22	-1.71	-4.85	1.22219E-06	0.000172337	308.580978	415.371576	123.687438	47.5943653	28.1196191	391.083315	127.687084	76.4783214	864.35285	346.88595	345.136788	361.418172	269.135854	605.9401498	560.905424	347.0950934	DEE49|EIEE4	MIM:617278	11	11p15.4	DENN domai	protein-codir	DENND5A	DENN domai	O	DENN domai	20210302
RPL30	ENSG00000156482	6156	4757.95	-0.62	-4.85	1.26517E-06	0.000177086	6764.265	5808.55461	3376.22013	3623.37647	1805.06981	3243.15776	2837.84619	2708.90237	9685.17317	6883.09412	5396.98721	8195.44651	2768.99038	4121.755854	4064.83462	4843.55468	L30	MIM:180467	8	8q22.2	ribosomal pr	protein-codir	RPL30	ribosomal pr	O	60S ribosoma	20210404
LPAR6	ENSG00000139679	10161	717.55	0.83	4.84	1.27921E-06	0.000177744	549.871919	955.536529	1549.55194	1452.76533	1085.97792	383.485198	642.914353	735.560704	207.531103	660.35308	635.668348	732.520196	841.458631	332.7063868	339.517453	375.4142124	ARWH1|HYP	MIM:609239	13	13q14.2	lysophosphat	protein-codir	LPAR6	lysophosphat	O	lysophosphat	20210302
	ENSG00000283907		989.42	-1.23	-4.83	1.34641E-06	0.000184413	530.766529	1393.14917	83.0497659	85.5338121	288.213876	589.538504	418.546667	910.650934	2458.22905	3733.31944	204.151072	66.3527345	1215.71415	1226.052354	820.158763	1807.341116											
C1QBP	ENSG00000108561	708	167.75	-1.18	-4.83	1.34658E-06	0.000184413	113.841188	113.91206	165.496525	134.655547	35.7054619	135.148427	72.2808031	62.1344056	400.03673	180.126267	188.233312	356.296966	110.997108	219.9525357	209.983333	185.175091	COXPD33|GC	MIM:601269	17	17p13.2	complement	protein-codir	C1QBP	complement	O	complement	20210404
	ENSG00000281383		1660.46	-3.38	-4.83	1.37337E-06	0.000185929	130.454109	754.924632	0	17.2897648	839.6373	65.8232468	208.716592	343.985324	6401.36403	7975.46092	50.1746832	4.1336055	2384.51825	1314.036025	2324.00375	3752.898695											
ERMARD	ENSG00000130023	55780	186.23	0.94	4.83	1.37718E-06	0.000185929	187.042548	221.145735	485.89261	175.439653	245.970696	212.77349	279.942208	155.604334	62.4959418	122.042999	170.512545	95.7652101	157.172605	165.1932112	163.924189	78.82310366	C6orf70|PVN	MIM:615532	6	6q27	ER membran	protein-codir	ERMARD	ER membran	O	endoplasmic	20210302
MYC	ENSG00000136997	4609	418.56	-2.58	-4.82	1.40114E-06	0.000187831	777.489285	440.572487	69.8150617	48.575394	2.89124327	290.696465	85.5027951	38.402281	678.981464	553.308912	622.241103	925.903027	470.953007	707.7480187	660.833747	323.0576052	MRTL|MYCC	MIM:190080	8	8q24.21	MYC proto-o	protein-codir	MYC	MYC proto-o	O	myc proto-or	20210418
MIF	ENSG00000240972	4282	210.77	-0.93	-4.82	1.45567E-06	0.000193777	116.424553	159.361384	197.27498	109.233851	142.248692	116.981074	132.799553	196.276672	263.803222	372.096054	238.919528	291.447812	197.220308	271.9213973	273.425045	292.8195861	GIF|GLIF|MN	MIM:153620	22	22q11.23	macrophage	protein-codir	MIF	macrophage	O	macrophage	20210418
AQP3	ENSG00000165272	360	128.56	-1.36	-4.81	1.49214E-06	0.000197252	155.340576	131.935823	56.6089251	22.7159339	35.0447808	90.6158589	107.444898	25.8704067	351.419682	177.378494	77.837457	105.657561	110.542394	234.3746501	253.563589	120.5319409	AQP-3|GIL	MIM:600170	9	9p13.3	aquaporin 3 (	protein-codir	AQP3	aquaporin 3 (	O	aquaporin-3|	20210418
CXCL8	ENSG00000169429	3576	953.32	-3.51	-4.81	1.50534E-06	0.000197626	813.851679	224.385222	5.9171161	10.9931222	30.757569	0	8.84830646	1.4985298	13138.4785	84.3822132	166.523964	155.749044	545.248473	23.02083508	15.5530777	27.90891919	GCP-1|GCP1	MIM:146930	4	4q13.3	C-X-C motif c	protein-codir	CXCL8	C-X-C motif c	O	interleukin-8	20210418
TPTEP1	ENSG00000100181	387590	39.44	-4.05	-4.81	1.53247E-06	0.000198153	1.08528494	47.2266004	3.32543755	0	0	1.02481347	3.82967133	1.27682291	13.9141708	10.3642522	28.0547656	371.071414	3.25938594	41.28752905	64.2070806	41.07687811	psiTPTE22	HGNC:HGNC:	22	22q11.1	TPTE pseudo	pseudo	TPTEP1	TPTE pseudo	O	endogenous	20210302
	ENSG00000258377		37.83	-3.38	-4.81	1.53405E-06	0.000198153	3.43732069	17.7562039	0	3.22573769	0	8.12345306	5.82093609	2.03535501	179.114195	244.075505	6.39570394	2.86183541	26.4920198	27.22457645	33.1560592	45.48816968											
RBM45	ENSG00000155636	129831	169.79	1.10	4.81	1.54059E-06	0.000198153	198.717865	133.454659	479.242137	182.246459	236.927142	223.340197	240.010412	180.73305	97.8815976	91.6005614	112.053964	69.870046	174.126321	93.89465899	96.0378526	106.5274391	DRB1|RB-1	MIM:608888	2	2q31.2	RNA binding	protein-codir	RBM45	RNA binding	O	RNA-binding	20210417
MIAT	ENSG00000225783	440823	2722.75	0.96	4.79	1.62894E-06	0.000208111	2396.17914	1505.5835	4651.07633	1908.82656	5715.12833	3375.32683	4546.68285	4212.22731	720.847801	1498.48663	2459.92497	884.964404	4164.44611	2103.326432	2018.04883	1402.93858	C22orf35|GC	MIM:611082	22	22q12.1	myocardial ir	ncRNA	MIAT	myocardial ir	O	MI related nc	20210418
RPL41	ENSG00000229117	6171	4890.91	-0.78	-4.79	1.66202E-06	0.000210922	3501.76528	3646.28574	4295.0766	4066.12023	1799.67687	4304.1534	3066.95595	3486.77	10958.6998	5719.89404	6497.05795	8075.38004	3173.20864	4695.137642	4663.22592	6305.135111	L41	MIM:613315	12	12q13.2	ribosomal pr	protein-codir	RPL41	ribosomal pr	O	60S ribosoma	20210302
TSPAN6	ENSG00000000003	7105	20.41	-4.09	-4.78	1.78564E-06	0.000225109	8.78504297	1.02110225	0	0	0	0	0	0.4421871	273.962948	14.1064856	6.6047344	3.46898438	9.33556164	3.496582641	3.01554701	2.358215443	T245|TM4SF	MIM:300191	X	Xq22.1	tetraspanin 6	protein-codir	TSPAN6	tetraspanin 6	O	tetraspanin-6	20210302
ARAP2	ENSG00000047365	116984	3071.74	0.74	4.77	1.81416E-06	0.0002272	2112.74782	3047.07948	3298.90874	4893.70043	5225.80501	2909.6942	4557.64423	4576.9397	1106.61122	2676.52545	2893.59061	2145.8248	3027.92331	1574.33907	2017.39039	3083.174617	CENTD1|PAR	MIM:606645	4	4p14	ArfGAP with	protein-codir	ARAP2	ArfGAP with	O	arf-GAP with	20210302
LOC1053702	ENSG00000286330	105370259	69.35	-3.34	-4.77	1.84856E-06	0.000229995	160.354	88.2405425	0	0	0	22.7712485	4.49309502	3.59944123	355.705864	102.69128	51.4652912	91.9523229	34.566567	86.21644512	69.3387006	38.1483577	-	Ensembl:ENS	13	13q22.1	uncharacteriz	ncRNA	-	-	-	-	20210302
SNN	ENSG00000184602	8303	73.67	-1.72	-4.76	1.95832E-06	0.000240507	62.6324613	76.495198	55.614408	16.8249847	26.6336809	17.1450977	19.3585549	24.2953113	212.258	108.97721	68.8406718	111.542616	64.2441029	98.16379143	107.768123	107.8700106	-	MIM:603032	16	16p13.13	stannin	protein-codir	SNN	stannin	O	stannin|AG8_	20210302
AOAH	ENSG00000136250	313	1044.90	0.75	4.76	1.94921E-06	0.000240507	661.964643	1026.50208	2141.90092	1833.47087	934.583923	1368.7303	1038.86676	1428.82011	318.910627	810.749132	1329.63415	1051.10669	847.589532	527.8010145	607.844813	789.9513948	-	MIM:102593	7	7p14.2	acyloxyacyl h	protein-codir	AOAH	acyloxyacyl h	O	acyloxyacyl h	20210302
TTC9	ENSG00000133985	23508	187.92	-1.76	-4.74	2.12604E-06	0.000259431	301.139462	243.137273	22.1274804	59.298792	37.6172805	112.429684	37.9022132	19.8381201	841.005617	149.786802	157.294332	279.566036	114.080927	263.4847041	247.868148	120.078006	TTC9A	MIM:610488	14	14q24.2	tetratricopep	protein-codir	TTC9	tetratricopep	O	tetratricopep	20210302
ZBED2	ENSG00000177494	79413	20.24	-3.39	-4.73	2.23027E-06	0.000268705	5.53068377	5.68369758	0	1.65963937	4.87559001	0	1.33451445	1.23010009	119.775897	127.085693	3.40131969	2.54487381	21.2613665	7.499539403	13.5122833	8.488295778	-	MIM:615246	3	3q13.13	zinc finger BE	protein-codir	ZBED2	zinc finger BE	O	zinc finger BE	20210302
MACF1	ENSG00000127603	23499	8938.65	0.76	4.73	2.22034E-06	0.000268705	5927.40931	7575.77158	7708.65984	12280.3665	16867.3887	10366.9993	13038.4661	17622.9536	5439.18864	4897.80805	6056.81583	4905.84794	9176.42622	7120.540396	6617.45336	7416.26751	ABP620|ACF	MIM:608271	1	1p34.3	microtubule	protein-codir	MACF1	microtubule	O	microtubule-	20210401
GNLY	ENSG00000115523	10578	2829.07	0.93	4.73	2.27657E-06	0.000272558	2402.52884	1813.98133	3085.86335	3065.05742	3969.02372	2383.34238	3359.79911	9336.14576	1078.15412	1580.09055	2241.82753	1310.91111	3170.06773	1087.341055	934.714434	4446.195375	D2S69E|LAG	MIM:188855	2	2p11.2	granulysin	protein-codir	GNLY	granulysin	O	granulysin|T-	20210406
NELL2	ENSG00000184613	4753	1389.04	-2.23	-4.72	2.30911E-06	0.000274726	2573.68396	1526.14898	700.949721	181.784013	99.5955022	1647.98729	152.913281	40.5665669	1837.03323	1765.24397	2151.78149	2223.49996	1118.17167	3336.228067	1996.47699	872.520915	NRP2	MIM:602320	12	12q12	neural EGFL	protein-codir	NELL2	neural EGFL	O	protein kinas	20210302
	ENSG00000285608		50.09	-4.22	-4.72	2.40222E-06	0.000284029	14.1022048	6.11141198	0	0	0	0	0	0	470.247816	289.986481	0	0	4.97744518	4.762009922	6.13498885	5.093534191											
TNFAIP8L1	ENSG00000185361	126282	122.42	-1.16	-4.71	2.4661E-06	0.000289782	115.673355	108.833403	80.9688615	42.0348825	36.7935051	112.540898	71.8814518	46.105961	247.844625	168.492454	102.504319	85.7202195	143.673821	216.1697664	229.953676	149.4927873	TIPE1	MIM:615869	19	19p13.3	TNF alpha inc	protein-codir	TNFAIP8L1	TNF alpha inc	O	tumor necros	20210406
HEATR9	ENSG00000270379	256957	73.20	1.35	4.70	2.56041E-06	0.000299018	69.1639642	46.6478991	133.844743	72.7474904	107.867608	190.471529	95.3741043	120.56839	10.1589048	28.4757945	40.5062686	32.6615767	73.1229297	60.32190725	30.0804412	59.14437378	C17orf66	HGNC:HGNC:	17	17q12	HEAT repeat	protein-codir	HEATR9	HEAT repeat	O	protein HEAT	20210302
PPA1	ENSG00000180817	5464	319.17	-0.99	-4.70	2.64354E-06	0.000306844	416.432366	302.805836	115.1963	241.338745	54.8720364	212.444807	180.381503	171.826664	1057.32379	274.465585	325.552334	556.34187	140.615572	373.6420909	345.855693	337.5856916	HEL-S-66p|IC	MIM:179030	10	10q22.1	inorganic pyr	protein-codir	PPA1	inorganic pyr	O	inorganic pyr	20210307
CERKL	ENSG00000188452	375298	86.94	-3.69	-4.70	2.6627E-06	0.000307195	4.51827682	29.7636329	3.44357308	0	2.092943	0	1.26685508	19.2971555	480.799227	763.516791	2.18672149	2.92725423	19.7864438	20.30172275	15.8343518	25.38442849	RP26	MIM:608381	2	2q31.3	ceramide kin	protein-codir	CERKL	ceramide kin	O	ceramide kin	20210410
COTL1	ENSG00000103187	23406	572.30	-1.25	-4.68	2.81415E-06	0.000322712	630.706185	874.05216	211.522596	623.275174	135.465708	181.206016	269.087308	146.443359	1313.80067	997.934607	503.071638	741.255067	560.421421	747.0824194	527.687825	693.843809	CLP	MIM:606748	16	16q24.1	coactosin like	protein-codir	COTL1	coactosin like	O	coactosin-like	20210302
RPS4X	ENSG00000198034	6191	6316.65	-0.59	-4.68	2.92632E-06	0.000329617	5805.81976	6768.13621	3615.59344	4599.17445	4323.41256	4842.96519	5841.61603	4790.51495	8394.13001	6492.61058	5820.51178	8306.79131	7149.81074	6874.087833	9010.66044	8430.5276	CCG2|DXS30	MIM:312760	X	Xq13.1	ribosomal pr	protein-codir	RPS4X	ribosomal pr	O	40S ribosoma	20210404
	ENSG00000286065		452.80	0.77	4.68	2.91262E-06	0.000329617	339.465085	324.397067	606.8066	529.303637	996.445793	551.129427	580.844433	620.110309	136.740507	304.371304	399.150367	364.76542	553.549752	256.5053643	296.459765	384.783687											
WDR76	ENSG00000092470	79968	223.49	1.09	4.68	2.90821E-06	0.000329617	104.026493	291.450683	425.490358	318.667211	325.862286	365.600576	382.596044	239.26539	74.8789294	123.255682	139.871623	161.446728	170.443556	121.5710497	110.616678	220.8021809	CDW14	HGNC:HGNC:	15	15q15.3	WD repeat d	protein-codir	WDR76	WD repeat d	O	WD repeat-c	20210302
TAF1	ENSG00000147133	6872	1293.04	0.74	4.67	2.95125E-06	0.00033047	720.805276	926.949899	2852.24956	1540.01835	1698.98777	1778.69275	1976.95306	1553.78314	400.702512	741.423272	961.783795	862.384983	1334.26656	1240.3108	1133.87209	965.5002024	BA2R|CCG1|	MIM:313650	X	Xq13.1	TATA-box bin	protein-codir	TAF1	TATA-box bin	O	transcription	20210410
R3HDM4	ENSG00000198858	91300	220.35	-0.87	-4.67	3.0811E-06	0.000337079	300.381321	236.482159	85.8782668	92.6227821	86.4996687	159.650912	176.946306	148.23824	396.785681	344.08456	190.236939	228.814004	236.994635	303.6848775	327.933926	210.4005185	C19orf22	HGNC:HGNC:	19	19p13.3	R3H domain	protein-codir	R3HDM4	R3H domain	O	R3H domain-	20210302
IL18R1	ENSG00000115604	8809	196.58	0.85	4.67	3.06188E-06	0.000337079	167.087376	509.223457	235.115778	130.029956	106.644273	231.017782	287.247564	368.847879	82.3432178	232.278989	175.145206	95.8332222	68.0445284	82.13730586	156.56187	217.7208649	CD218a|CDw	MIM:604494	2	2q12.1	interleukin 1	protein-codir	IL18R1	interleukin 1	O	interleukin-1	20210302
PTPRM	ENSG00000173482	5797	575.97	1.27	4.67	3.07463E-06	0.000337079	598.59473	331.810237	1739.18535	1121.30241	645.945501	647.662339	1069.517	515.287278	361.423782	255.369545	330.12244	373.859918	419.771552	159.6747925	257.977178	387.9423228	PTPRL1|R-PT	MIM:176888	18	18p11.23	protein tyros	protein-codir	PTPRM	protein tyros	O	receptor-type	20210327
TSPOAP1	ENSG00000005379	9256	375.04	1.47	4.67	3.03752E-06	0.000337079	277.989072	54.0775223	1995.59183	248.488761	744.55787	621.238691	359.403546	262.055697	59.8930694	68.0055329	329.409869	63.3011557	485.019447	145.4920966	199.35326	86.81279586	BZRAP1|PBR	MIM:610764	17	17q22	TSPO associa	protein-codir	TSPOAP1	TSPO associa	O	peripheral-ty	20210302
APEX1	ENSG00000100823	328	441.09	-0.68	-4.66	3.10755E-06	0.00033803	530.921001	461.320172	301.732391	448.585529	127.769841	343.109458	231.503913	284.888876	1002.10576	522.845265	456.813826	690.392617	290.435202	511.8671905	428.897568	424.1956101	APE|APE1|A	MIM:107748	14	14q11.2	apurinic/apyr	protein-codir	APEX1	apurinic/apyr	O	DNA-(apurini	20210418
PITPNM2	ENSG00000090975	57605	107.24	-1.90	-4.66	3.15155E-06	0.000340578	147.063723	53.3297528	26.3463259	23.4390684	16.5752863	122.293514	35.7485023	11.7425798	166.630613	96.5640014	126.95311	188.216379	161.104215	185.8140702	304.844359	49.23724481	NIR-3|NIR3|	MIM:608920	12	12q24.31	phosphatidyl	protein-codir	PITPNM2	phosphatidyl	O	membrane-a	20210417
DEGS2	ENSG00000168350	123099	19.15	-3.83	-4.65	3.24022E-06	0.000346521	0.391238	3.49110511	0	0	3.01952377	0.77571499	2.23285903	4.49100397	115.471942	94.6978233	2.3289482	5.96414673	19.0180233	21.76790097	7.84519665	24.92108295	C14orf66|DE	MIM:610862	14	14q32.2	delta 4-desat	protein-codir	DEGS2	delta 4-desat	O	sphingolipid	20210302
CACNA1I	ENSG00000100346	8911	127.04	-2.99	-4.64	3.52765E-06	0.000375153	213.371966	156.295438	0	10.1368514	0	93.4904586	20.9544471	10.8638234	134.809704	279.274657	147.486003	83.4145342	140.145757	434.7202085	166.377194	141.3760913	Cav3.3|ca(v)	MIM:608230	22	22q13.1	calcium volta	protein-codir	CACNA1I	calcium volta	O	voltage-depe	20210302
NLRP6	ENSG00000174885	171389	39.82	-2.64	-4.63	3.59373E-06	0.000380056	47.2043065	33.8120659	2.67183023	3.21280205	3.90768895	24.2732443	0.90447187	7.05604066	98.8875325	94.8253532	39.4807683	90.4831769	38.7842751	82.64311048	48.0427344	20.93343486	AVR|CLR11.4	MIM:609650	11	11p15.5	NLR family p	protein-codir	NLRP6	NLR family p	O	NACHT, LRR a	20210307
GTF3C5	ENSG00000148308	9328	542.61	1.64	4.60	4.32093E-06	0.000454438	175.170191	179.558499	422.067145	174.776025	2936.17713	1588.85117	823.474915	843.308998	120.6241	173.080086	183.223981	124.240656	447.431976	176.6386745	130.923388	182.2899262	TFIIIC63|TFIII	MIM:604890	9	9q34.13	general trans	protein-codir	GTF3C5	general trans	O	general trans	20210307
IRS2	ENSG00000185950	8660	203.48	-2.02	-4.59	4.37274E-06	0.00045736	239.715947	297.87394	100.456769	70.749577	28.9212773	87.1500692	47.5857636	2.11914877	514.642947	461.364764	254.579141	296.018548	148.304467	233.7483956	317.811635	154.710991	IRS-2	MIM:600797	13	13q34	insulin recep	protein-codir	IRS2	insulin recep	O	insulin recep	20210302
KBTBD11	ENSG00000176595	9920	53.55	-2.98	-4.59	4.51541E-06	0.000467149	82.2467759	53.7056925	0	1.54128334	5.37832338	22.7089511	2.13927267	14.1689939	119.914704	98.6412287	65.4317111	96.8117876	63.6814281	46.69509925	80.5309188	103.2408626	KLHDC7C	MIM:618794	8	8p23.3	kelch repeat	protein-codir	KBTBD11	kelch repeat	O	kelch repeat	20210302
	ENSG00000265735		148.01	-1.82	-4.59	4.49364E-06	0.000467149	97.1999508	76.2774143	56.2333245	34.5806443	8.41253822	24.3670809	11.7813708	19.2443124	695.448977	943.787413	73.5587592	172.271875	16.3050945	31.98625685	46.3272491	60.3330364											
	ENSG00000228830		24.07	-4.42	-4.58	4.63999E-06	0.000477443	1.38132982	1.05755004	0	0	0	0	2.28159152	0.68106559	197.036322	122.633026	0	0.50924581	8.12013025	21.72547043	11.3906515	18.37713611											
PYGB	ENSG00000100994	5834	264.28	-0.84	-4.58	4.67539E-06	0.000478499	362.804615	177.831516	86.1858773	266.854636	179.851398	196.544665	164.312138	124.84276	545.473731	347.108476	288.685848	330.545688	316.272067	308.7670151	269.103071	263.2170018	GPBB	MIM:138550	20	20p11.21	glycogen pho	protein-codir	PYGB	glycogen pho	O	glycogen pho	20210417
FOXO1	ENSG00000150907	2308	897.76	-0.84	-4.57	4.83165E-06	0.000489568	1222.43185	860.709128	559.56596	794.345891	446.383496	536.967335	459.682755	383.478162	1157.09692	1203.82868	1079.1958	1908.74943	702.26367	973.4836698	1141.29422	934.6748604	FKH1|FKHR|	MIM:136533	13	13q14.11	forkhead box	protein-codir	FOXO1	forkhead box	O	forkhead box	20210406
DLG1	ENSG00000075711	1739	785.68	0.90	4.57	4.83499E-06	0.000489568	567.13686	693.770461	2819.29191	1077.17854	1161.81455	685.978398	769.921135	770.215158	381.754674	457.521666	691.549802	486.475814	512.911954	503.3568874	472.947696	519.058728	DLGH1|SAP-	MIM:601014	3	3q29	discs large M	protein-codir	DLG1	discs large M	O	disks large hc	20210316
NKTR	ENSG00000114857	4820	3694.62	0.77	4.57	4.87993E-06	0.000491505	4234.38035	3150.31756	5952.20385	4542.31925	5130.47068	4760.98918	4455.75261	4366.50514	1655.39039	2045.89533	3977.59622	2447.22305	3527.93503	3738.279196	3591.49637	1537.099254	p104	MIM:161565	3	3p22.1	natural killer	protein-codir	NKTR	natural killer	O	NK-tumor rec	20210404
PASK	ENSG00000115687	23178	854.08	-2.23	-4.56	5.0841E-06	0.000509373	1359.23819	553.662534	25.0583131	148.663107	124.043343	796.749978	231.676364	167.791082	738.279546	807.045974	1273.30644	2293.7516	675.904504	1758.000219	1822.1981	889.9275722	PASKIN|STK3	MIM:607505	2	2q37.3	PAS domain	protein-codir	PASK	PAS domain	O	PAS domain-	20210302

C1orf50	ENSG00000164008	79078	162.21	1.28	4.55	5.31766E-06	0.000529984	91.6091735	152.371833	817.560857	216.942602	235.358366	266.473948	100.686072	78.005011	36.900126	92.4381192	128.267467	65.1591246	111.863334	77.27904277	71.4498944	52.91927625	-	HGNC:HGNC:	1	1p34.2	chromosome	protein-codir	C1orf50	chromosome	O	uncharacteri	20210302
PTPN22	ENSG00000134242	26191	1833.30	0.85	4.55	5.38879E-06	0.000534276	1048.02452	1162.21834	6728.17392	1971.42743	2034.73392	1533.0513	2402.71623	2756.32849	593.508563	1163.78097	1887.62235	1286.49557	1200.72231	798.6530077	1112.54861	1652.745311	LYP|LYP1|LYI	MIM:600716	1	1p13.2	protein tyros	protein-codir	PTPN22	protein tyros	O	tyrosine-prot	20210329
HAPLN3	ENSG00000140511	145864	79.11	-3.35	-4.55	5.43382E-06	0.000535949	155.226244	45.1063167	8.17490524	10.5319603	0	61.0726746	1.63098761	0.48985305	110.240725	72.4508089	58.09669	68.2013249	110.061743	346.0716394	163.122339	55.25445188	EXLD1|HsT19	HGNC:HGNC:	15	15q26.1	hyaluronan a	protein-codir	HAPLN3	hyaluronan a	O	hyaluronan a	20210302
PTP4A1	ENSG00000285976	7803	367.66	-5.81	-4.54	5.53994E-06	0.00054248	826.395594	193.722266	0	0	0	61.6655398	23.0240466	0	1836.6586	586.16436	1176.64616	363.802309	148.216644	179.6473911	274.055194	212.530891	HH72|PRL-1|	MIM:601585	6	6q12	protein tyros	protein-codir	PTP4A1	protein tyros	O	protein tyros	20210302
SNORA81	ENSG00000221420	677847	126.82	2.18	4.54	5.55703E-06	0.00054248	351.901122	314.760502	193.212375	312.471943	69.7378903	164.081773	59.7492242	158.083907	9.30581253	40.0331232	94.1176598	67.2080236	51.8166757	11.80060538	15.2903091	115.4744789	HBI-61	MIM:611334	3	3q27.3	small nucleol	snoRNA	SNORA81	small nucleol	O	HBI-61 snoRN	20210302
ADRB2	ENSG00000169252	154	443.06	0.86	4.54	5.6404E-06	0.000547809	236.114307	409.658749	609.619902	775.328923	722.224641	529.460506	652.00169	584.368161	152.706067	286.47277	258.694175	391.636104	555.752501	195.5857183	251.100592	478.2749254	ADRB2R|ADF	MIM:109690	5	5q32	adrenocepto	protein-codir	ADRB2	adrenocepto	O	beta-2 adren	20210406
	ENSG00000270066		2128.49	-6.30	-4.53	5.82678E-06	0.000563038	77.1441185	15.5934963	0	277.024715	0	2867.83045	0	493.279028	5481.44742	11488.3367	74.5937619	59.6348698	924.232916	4903.784693	5808.1507	1584.830682											
P2RX7	ENSG00000089041	5027	94.16	1.04	4.53	5.86338E-06	0.000563714	32.6622211	148.144792	196.979032	70.7091801	264.967742	121.227744	143.137133	50.3511334	23.7975175	45.1348739	63.4264377	42.1427729	134.040724	64.95316766	77.0279034	27.85567987	P2X7	MIM:602566	12	12q24.31	purinergic re	protein-codir	P2RX7	purinergic re	O	P2X purinoce	20210406
ISG20	ENSG00000172183	3669	266.62	-0.66	-4.53	5.99695E-06	0.000573658	313.745254	233.540885	74.4285761	199.175774	136.728497	189.895813	238.521865	279.118032	471.004485	378.763423	155.060845	335.117801	206.025341	366.2237982	350.315963	338.2607895	CD25|HEM45	MIM:604533	15	15q26.1	interferon sti	protein-codir	ISG20	interferon sti	O	interferon-sti	20210417
	ENSG00000287286		31.63	-3.16	-4.51	6.42072E-06	0.000611124	4.9105736	8.60656201	0	0	6.90137305	0.82013688	1.31396439	11.6490493	157.278649	225.876458	1.34907598	0.64348881	18.8240371	22.7770685	15.3927229	29.73455535											
TMEM204	ENSG00000131634	79652	236.86	-2.16	-4.51	6.50363E-06	0.000615936	337.096888	142.056223	0	131.469015	16.2215705	162.402287	100.903269	65.2713733	324.129171	314.854389	201.45136	536.428025	239.576919	345.506095	561.038598	311.2835342	C16orf30|CLI	MIM:611002	16	16p13.3	transmembra	protein-codir	TMEM204	transmembra	O	transmembra	20210302
EIF3H	ENSG00000147677	8667	1273.70	-0.64	-4.50	6.72687E-06	0.000633924	1340.67209	1382.20929	914.866737	955.214484	560.496867	1118.52079	770.973854	974.486481	2261.36808	1319.79749	1442.02413	1675.91368	912.33804	1534.819101	1571.34849	1644.163316	EIF3S3|eIF3-	MIM:603912	8	8q23.3-q24.1	eukaryotic tra	protein-codir	EIF3H	eukaryotic tra	O	eukaryotic tra	20210404
SGPP2	ENSG00000163082	130367	40.46	-2.92	-4.50	6.85539E-06	0.000642854	9.25908667	37.6749991	0	1.6238358	9.42990558	5.79334081	6.02549232	0.62097173	373.853052	63.8093564	25.3421294	16.2656783	18.0510521	34.6294143	28.556837	16.35609373	SPP2|SPPase	MIM:612827	2	2q36.1	sphingosine-	protein-codir	SGPP2	sphingosine-	O	sphingosine-	20210408
PAX5	ENSG00000196092	5079	1072.32	3.75	4.50	6.89432E-06	0.000643335	0.90160625	0.96932199	171.820465	79.1622835	9885.20408	3461.77489	1509.66462	1315.01451	2.85622749	9.08398298	8.67602746	1.86306081	486.566606	79.1824308	67.7150785	76.73956454	ALL3|BSAP	MIM:167414	9	9p13.2	paired box 5	protein-codir	PAX5	paired box 5	O	paired box pr	20210322
SPECC1L-ADC	ENSG00000258555	101730217	64.98	8.21	4.49	7.03995E-06	0.00065372	154.360046	144.187262	0	121.747828	114.114478	170.630711	42.5706363	106.860148	0	0	46.6393166	0	0	72.1696413	0	66.47388892	-	HGNC:HGNC:	22	22q11.23	SPECC1L-ADC	ncRNA	SPECC1L-ADC	SPECC1L-ADC	O	-	20210302
SH3BGRL2	ENSG00000198478	83699	98.85	-2.69	-4.48	7.34451E-06	0.00067869	54.6497329	248.382621	3.31327841	9.58686526	24.3135641	67.1219384	3.93472304	1.85644829	471.479506	182.55444	64.7393694	141.747285	37.582404	105.4230664	100.981199	63.93478126	-	MIM:615678	6	6q14.1	SH3 domain I	protein-codir	SH3BGRL2	SH3 domain I	O	SH3 domain-	20210302
RPS6	ENSG00000137154	6194	11690.56	-0.72	-4.48	7.52835E-06	0.000692317	12006.5477	12305.5291	10098.3343	8083.7683	5303.91038	9828.91463	6310.63257	7278.05438	18345.5959	13129.773	13090.623	21284.705	8889.60458	13508.0131	13466.1406	14118.75785	S6	MIM:180460	9	9p22.1	ribosomal pro	protein-codir	RPS6	ribosomal pro	O	40S ribosoma	20210404
LDLRAP1	ENSG00000157978	26119	522.88	-1.39	-4.48	7.57053E-06	0.000692849	817.867846	529.216807	102.161869	158.425207	116.077852	361.981031	299.949821	275.264666	605.700155	705.352855	487.529031	630.853293	691.71987	932.663487	984.946417	666.2981864	ARH|ARH1|A	MIM:605747	1	1p36.11	low density li	protein-codir	LDLRAP1	low density li	O	low density li	20210302
EEIG1	ENSG00000167106	399665	1831.14	-1.24	-4.47	7.91906E-06	0.000721278	1932.68609	890.280646	435.921234	522.086213	2002.52799	1471.33454	1264.59722	849.670682	1394.85967	2047.9094	1965.40364	2694.43336	3145.64641	3319.366102	2582.08378	2779.356424											
RSRC2	ENSG00000111011	65117	1122.74	0.60	4.46	8.06069E-06	0.000727219	1097.82691	1272.61196	2309.12175	1733.46832	1273.81366	1016.16482	1027.16658	1068.60698	1064.99309	851.345541	1311.69832	1238.74751	762.478562	649.8618131	769.197701	516.7108166	-	HGNC:HGNC:	12	12q24.31	arginine and	protein-codir	RSRC2	arginine and	O	arginine/seri	20210417
KLRC3	ENSG00000205810	3823	353.99	1.23	4.46	8.03053E-06	0.000727219	180.031301	112.628732	2422.96724	219.509505	307.048137	536.451738	241.297649	314.748506	95.609791	96.3804351	485.5116	129.722795	164.616591	90.32309179	99.8888965	167.053142	NKG2-E|NKG	MIM:602892	12	12p13.2	killer cell lect	protein-codir	KLRC3	killer cell lect	O	NKG2-E type	20210302
SULT1B1	ENSG00000173597	27284	142.67	-1.90	-4.46	8.13987E-06	0.000730899	215.346944	45.2686605	0	50.9992116	21.9469606	65.2412788	56.0969227	18.2354661	673.422952	58.5648714	114.927016	479.840974	45.5742073	163.4871822	218.214679	55.53324542	ST1B1|ST1B2	MIM:608436	4	4q13.3	sulfotransfera	protein-codir	SULT1B1	sulfotransfera	O	sulfotransfera	20210302
SNED1	ENSG00000162804	25992	255.99	-2.27	-4.46	8.30124E-06	0.000737674	279.21791	124.995786	191.597821	11.1038658	9.43141237	442.407722	32.1174399	7.16644674	205.09525	259.45505	243.955863	182.972142	262.786949	1445.011661	284.267794	114.2878415	IRE-BP1|SST3	MIM:616634	2	2q37.3	sushi, nidoge	protein-codir	SNED1	sushi, nidoge	O	sushi, nidoge	20210302
CEP68	ENSG00000011523	23177	541.53	-1.07	-4.46	8.28065E-06	0.000737674	730.710165	670.65689	198.602842	206.0924	195.738819	489.396552	233.713798	276.33986	918.013359	722.649874	502.682594	733.690336	469.44575	852.8955524	957.564239	506.2075705	KIAA0582	MIM:616889	2	2p14	centrosomal	protein-codir	CEP68	centrosomal	O	centrosomal	20210302
SUMO3	ENSG00000184900	6612	212.29	-0.73	-4.46	8.33158E-06	0.000737674	109.701697	193.836485	201.75024	242.292075	97.6345784	116.412319	124.39121	210.637576	206.337901	276.321165	226.346237	398.881395	184.849816	213.690838	265.34579	328.2007136	SMT3A|SMT3	MIM:602231	21	21q22.3	small ubiquit	protein-codir	SUMO3	small ubiquit	O	small ubiquit	20210329
LOC1249049	ENSG00000273828	124904917	31.56	-2.23	-4.45	8.55064E-06	0.000750092	28.7164254	11.6529422	0	0	11.7066846	31.9248701	9.50564439	13.3926387	103.392148	42.0520418	14.3691611	28.5836491	57.7424378	41.1276784	46.9045499	63.84651083											
XRN1	ENSG00000114127	54464	2562.60	0.59	4.45	8.52093E-06	0.000750092	1313.37913	2396.75163	2286.01017	4684.06412	3991.28387	3150.67431	3333.7894	3753.80708	1046.44306	1533.89438	2023.30532	2062.04228	2687.9111	2181.571335	2399.44157	2157.292069	SEP1	MIM:607994	3	3q23	5'-3' exoribor	protein-codir	XRN1	5'-3' exoribor	O	5'-3' exoribor	20210306
CCR4	ENSG00000183813	1233	163.25	-2.96	-4.44	8.91092E-06	0.000778111	92.3098391	235.952065	0	13.0830614	0	205.613932	29.197115	7.98131871	57.0603796	332.427134	170.584411	203.784556	142.187397	893.1294031	120.605778	108.0843372	CC-CKR-4|CD	MIM:604836	3	3p22.3	C-C motif che	protein-codir	CCR4	C-C motif che	O	C-C chemokir	20210302
SNHG7	ENSG00000233016	84973	159.92	-1.11	-4.44	8.98106E-06	0.00077988	137.565331	158.972819	21.0715588	74.2502832	110.226343	141.278846	116.051071	74.0003298	349.936513	284.520988	143.080399	215.800877	153.907103	254.0137873	164.993627	159.0459884	NCRNA00061	HGNC:HGNC:	9	9q34.3	small nucleol	ncRNA	SNHG7	small nucleol	O	small nucleol	20210418
TRGC2	ENSG00000227191	6967	976.57	0.94	4.44	9.01311E-06	0.00077988	562.944383	735.104925	995.153943	1616.55761	1330.34518	1400.01162	3016.11994	862.438641	265.359256	504.537934	907.914835	745.041366	744.119435	483.0039085	796.314773	660.169237	TCRGC2|TRG	MIM:615450	7	7p14.1	T cell recepto	other	TRGC2	T cell recepto	O	T-cell recepto	20210302
COX4I1	ENSG00000131143	1327	1290.57	-0.63	-4.41	1.02829E-05	0.000885725	1118.93463	1078.84107	1279.70841	1244.70899	657.464003	901.697511	884.037723	935.511832	1596.50253	1233.37104	1454.33815	1939.3305	1056.1304	1661.937805	1513.5834	2092.986168	COX IV-1|CO	MIM:123864	16	16q24.1	cytochrome c	protein-codir	COX4I1	cytochrome c	O	cytochrome c	20210404
FBXL16	ENSG00000127585	146330	38.24	-3.85	-4.41	1.03983E-05	0.000891631	135.105167	40.0088787	4.07943587	0	0	4.98598535	0	2.78851752	46.7050081	88.5118528	26.4401537	34.3036144	54.8235925	66.67499596	58.2924647	49.17209499	C16orf22|Fbl	MIM:609082	16	16p13.3	F-box and leu	protein-codir	FBXL16	F-box and leu	O	F-box/LRR-re	20210302
	ENSG00000256667		326.73	1.07	4.40	1.06195E-05	0.000906514	228.700026	178.810994	1256.74683	428.564095	173.375939	343.039062	466.561382	499.451378	57.3451583	166.534938	502.412404	159.03706	142.881841	141.8896838	147.998048	334.3453682											
GZMB	ENSG00000100453	3002	378.88	1.31	4.40	1.09993E-05	0.000934745	139.260077	296.491422	685.730675	568.913979	675.66868	341.717515	456.72163	926.25448	56.4392102	296.953396	141.790811	272.621027	421.753027	71.29103017	90.8967207	619.6549383	C11|CCPI|CG	MIM:123910	14	14q12	granzyme B	protein-codir	GZMB	granzyme B	O	granzyme B|	20210406
GIMAP6	ENSG00000133561	474344	808.20	-0.90	-4.38	1.19568E-05	0.001011599	525.48995	482.301264	339.179861	417.500595	617.048765	795.130287	677.98541	640.431248	519.839593	911.611867	1073.64354	1366.49183	909.728996	1266.070818	1137.8828	1250.935059	IAN-2|IAN-6|	MIM:616960	7	7q36.1	GTPase, IMAI	protein-codir	GIMAP6	GTPase, IMAI	O	GTPase IMAP	20210302
APOBEC3G	ENSG00000239713	60489	1760.40	0.75	4.38	1.21129E-05	0.001020274	758.428645	1745.65889	2103.5947	1772.19349	3515.15832	1405.51711	2437.41934	3394.20924	411.632089	1715.48899	1154.58625	674.661449	2698.42799	752.5196489	1079.80224	2547.055615	A3G|ARCD|A	MIM:607113	22	22q13.1	apolipoprote	protein-codir	APOBEC3G	apolipoprote	O	DNA dC->dU-	20210329
	ENSG00000228903		44.07	-1.58	-4.37	1.23296E-05	0.001033947	30.2654694	40.4985499	30.080236	10.9066102	13.3891629	34.9724114	6.86796139	22.8510684	71.076404	109.480756	39.2177525	45.2216267	57.831328	77.00291636	54.0961158	61.40639099											
HINT1	ENSG00000169567	3094	750.27	-0.73	-4.37	1.24289E-05	0.001037705	782.582453	642.781246	672.295085	546.517297	421.434608	504.731224	388.269668	401.279716	1913.27877	838.055909	1011.81368	1230.63339	425.199843	859.3072639	688.679877	677.5222884	HINT|NMAN	MIM:601314	5	5q23.3	histidine triac	protein-codir	HINT1	histidine triac	O	histidine triac	20210418
KDSR	ENSG00000119537	2531	261.01	-1.38	-4.37	1.26977E-05	0.001051198	362.632385	303.282949	19.9360379	249.557716	30.2722083	164.568587	112.451763	96.5856157	684.411779	425.731424	235.49706	398.559807	91.7542119	276.4420306	377.318936	347.1940398	DHSR|EKVP4	MIM:136440	18	18q21.33	3-ketodihydr	protein-codir	KDSR	3-ketodihydr	O	3-ketodihydr	20210302
RPL10	ENSG00000147403	6134	7670.83	-0.68	-4.37	1.2701E-05	0.001051198	7970.86244	7311.06268	6075.21036	5124.68116	3213.5952	6989.33825	4841.81171	5941.85368	9260.95183	8495.34153	7224.69888	10965.4292	6427.02849	10611.27361	9762.86577	12517.21857	AUTSX5|DXS	MIM:312173	X	Xq28	ribosomal pro	protein-codir	RPL10	ribosomal pro	O	60S ribosoma	20210404
MMP28	ENSG00000271447	79148	11.52	-4.50	-4.36	1.27627E-05	0.001051739	1.42956805	4.18311349	0	0	0	9.31373098	0	0	30.0400496	9.54711968	3.99318508	29.7069705	27.0892291	38.6151475	18.1214354	12.31806061	EPILYSIN|MN	MIM:608417	17	17q12	matrix metall	protein-codir	MMP28	matrix metall	O	matrix metall	20210302
LRRN3	ENSG00000173114	54674	739.06	-4.13	-4.36	1.29002E-05	0.001058481	2420.59318	809.327359	2.92832025	8.72128583	32.6649458	741.740656	14.9680493	2.95263888	1310.8483	638.229848	719.422726	1515.86466	279.751174	1302.918403	1735.8876	288.1993704	FIGLER5|NLR	HGNC:HGNC:	7	7q31.1	leucine rich r	protein-codir	LRRN3	leucine rich r	O	leucine-rich r	20210302
PABPC1	ENSG00000070756	26986	16356.26	-0.74	-4.35	1.34396E-05	0.001098013	18457.9823	20847.2969	13165.9866	9826.68934	7823.55637	12901.0048	8980.75045	8500.93332	30073.3714	17509.2267	19889.6628	22243.621	13965.89	19566.26814	19704.6402	18243.27805	PAB1|PABP|	MIM:604679	8	8q22.3	poly(A) bindi	protein-codir	PABPC1	poly(A) bindi	O	polyadenylat	20210418
HIF1AN	ENSG00000166135	55662	819.65	0.69	4.35	1.35375E-05	0.00110128	1733.8767	788.212749	1177.86495	669.603004	1093.04643	891.115146	990.830807	866.81381	664.849171	649.738647	593.927038	525.441623	558.276788	599.6709464	714.397408	596.7593367	FIH1	MIM:606615	10	10q24.31	hypoxia indu	protein-codir	HIF1AN	hypoxia indu	O	hypoxia-indu	20210417
	ENSG00000219410		18.70	-4.03	-4.34	1.42138E-05	0.001146499	12.3599192	5.78366542	0	0	0	0	0.91542336	0	72.3199729	138.264744	0	1.08890549	9.53466121	10.56746382	23.5241765	24.77039818											
VCL	ENSG00000035403	7414	451.00	0.81	4.34	1.43879E-05	0.001155642	619.492498	573.14014	441.278379	792.227267	429.85559	484.093148	691.570136	569.569949	270.655635	421.502477	284.777457	255.066388	386.044183	262.3297685	366.817924	367.6331085	CMD1W|CM	MIM:193065	10	10q22.2	vinculin	protein-codir	VCL	vinculin	O	vinculin|epid	20210404
CD55	ENSG00000196352	1604	656.99	-1.64	-4.33	1.47004E-05	0.001175784	1306.73165	961.829233	153.45659	77.1838015	12.3363673	365.087835	131.410014	111.593993	4195.38605	607.191347	405.948084	447.639391	219.681159	504.0820178	646.734679	365.4750207	CHAPLE|CR|(	MIM:125240	1	1q32.2	CD55 molecu	protein-codir	CD55	CD55 molecu	O	complement	20210307
ANKRD36	ENSG00000135976	375248	1226.73	0.66	4.33	1.50322E-05	0.001197293	727.272836	1192.71444	1687.34966	1907.51395	2068.90921	1574.59945	1249.89648	1483.45173	407.148625	596.210965	1076.16359	1050.73	1407.47705	1203.518069	1288.87089	705.8344244	UNQ2430	HGNC:HGNC:	2	2q11.2	ankyrin repea	protein-codir	ANKRD36	ankyrin repea	O	ankyrin repea	20210302
SLFN12L	ENSG00000205045	100506736	468.06	0.86	4.33	1.512E-05	0.001199265	354.425867	457.746676	460.567501	522.335392	1044.68758	720.44598	678.983608	507.944071	104.623776	222.155068	494.010928	300.121704	539.950079	314.5731956	453.70447	312.6936025	-	MIM:614956	17	17q12	schlafen fami	protein-codir	SLFN12L	schlafen fami	O	schlafen fami	20210302
SNORD1C	ENSG00000274091	677850	24.03	-4.10	-4.31	1.62283E-05	0.001276537	34.3822227	36.9297145	0	4.08407642	0	1.84094772	0	0	46.9581387	39.3893916	95.7051062	28.5720886	11.0988318	31.79273703	30.8283541	22.8524921	R38C|snR38(	HGNC:HGNC:	17	17q25.1	small nucleol	snoRNA	SNORD1C	small nucleol	O	R38c snoRNA	20210302
CD27	ENSG00000139193	939	94.17	-2.55	-4.31	1.62239E-05	0.001276537	232.760923	151.594308	16.2710029	3.16045376	3.05509102	32.3306879	10.6319935	16.2668231	134.643	146.736814	88.0176564	155.626789	92.2163297	175.1125088	141.125153	107.1283412	S152|S152. L	MIM:186711	12	12p13.31	CD27 molecu	protein-codir	CD27	CD27 molecu	O	CD27 antigen	20210329
	ENSG00000233264		78.47	1.52	4.31	1.64648E-05	0.001289809	19.4018537	22.6889841	309.640208	127.875717	303.549624	73.6680684	68.8346939	62.0774432	11.5965958	27.0286147	47.9172663	30.4823393	75.0793993	21.01673871	26.4863305	28.20823873											
HSP90AB1	ENSG00000096384	3326	2585.07	-0.83	-4.31	1.66725E-05	0.001290157	4454.31174	3792.86774	1265.34283	1196.69378	895.860797	1530.12467	1121.7201	1265.04146	7422.88067	3232.46837	2277.21339	3603.02392	1432.22374	2663.984969	2983.33463	2224.061352	D6S182|HSP8	MIM:140572	6	6p21.1	heat shock pr	protein-codir	HSP90AB1	heat shock pr	O	heat shock pr	20210404
DAPK2	ENSG00000035664	23604	144.21	1.15	4.31	1.66487E-05	0.001290157	30.4641871	224.356941	174.56684	88.9929695	370.436401	172.960186	282.764554	191.66132	17.6967881	90.6530504	55.7741486	30.7590063	285.843287	105.048655	58.8517085	126.5939197	DRP-1|DRP1	MIM:616567	15	15q22.31	death associa	protein-codir	DAPK2	death associa	O	death-associa	20210417
B3GAT1	ENSG00000109956	27087	252.67	1.37	4.31	1.65728E-05	0.001290157	64.1635602	157.225662	513.131511	335.519443	593.470778	313.823749	556.355501	350.681252	38.3511663	156.434355	100.331451	126.851823	361.723086	40.09004011	165.318043	169.298289	CD57|GLCAT	MIM:151290	11	11q25	beta-1,3-gluc	protein-codir	B3GAT1	beta-1,3-gluc	O	galactosylgala	20210302
MTSS1	ENSG00000170873	9788	470.59	1.05	4.30	1.72161E-05	0.001326824	415.942954	405.305581	290.184446	748.826348	850.951245	584.814437	1283.40902	644.573031	239.723158	328.10238	276.330161	332.005583	312.569121	231.1044932	259.877948	325.6792169	MIM|MIMA|	MIM:608486	8	8q24.13	MTSS I-BAR d	protein-codir	MTSS1	MTSS I-BAR d	O	protein MTSS	20210406
TUT7	ENSG00000083223	79670	1162.01	0.73	4.30	1.73185E-05	0.001329334	1411.50404	1419.62309	1425.07027	1536.04867	1387.48104	1436.40046	1320.72592	1515.86036	1129.33963	567.373444	1146.40329	580.19938	955.03899	763.2808958	888.46129	1109.344894	PAPD6|TENT	MIM:613467	9	9q21.33	terminal urid	protein-codir	TUT7	terminal urid	O	terminal urid	20210302
CCR7	ENSG00000126353	1236	636.19	-3.70	-4.29	1.7538E-05	0.001340773	654.52454	606.272644	0	23.1466467	65.6273792	389.519603	87.4468809	14.0685361	567.53986	770.986587	941.739337	1901.8183	760.567886	1367.542581	1317.72956	710.4511583	BLR2|CC-CKR	MIM:600242	17	17q21.2	C-C motif che	protein-codir	CCR7	C-C motif che	O	C-C chemokir	20210417
	ENSG00000285830		31.60	-2.69	-4.29	1.81439E-05	0.00138155	24.8315458	10.5877546	0	1.65016173	2.12021115	1.69604791	2.59615207	4.31722722	161.915955	223.427517	3.04939756	0	17.5552869	16.21301276	16.2377696	19.4671501											
IGHMBP2	ENSG00000132740	3508	192.34	1.28	4.28	1.86352E-05	0.001413305	615.965608	221.486397	84.230176	494.124915	331.449809	220.305973	137.768949	165.234495	90.2656024	114.783106	98.2796349	151.173116	139.169859	65.40717439	77.4216742	70.30896737	CATF1|CMT2	MIM:600502	11	11q13.3	immunoglob	protein-codir	IGHMBP2	immunoglob	O	DNA-binding	20210410
CST7	ENSG00000077984	8530	1538.69	0.76	4.28	1.88141E-05	0.001421209	1089.44268	1581.54171	2451.55638	2826.12914	1467.96398	1748.02058	2152.28899	2081.84735	708.173709	1305.65071	1366.39055	1391.66358	1091.05751	678.991759	815.650308	1862.744923	CMAP	MIM:603253	20	20p11.21	cystatin F	protein-codir	CST7	cystatin F	O	cystatin-F|cy	20210302
PHF11	ENSG00000136147	51131	491.47	0.61	4.27	1.91675E-05	0.001442187	550.117958	513.273613	813.012048	502.941979	623.353458	543.678387	622.927262	576.033574	295.6179	421.514745	469.851155	418.123234	384.644165	444.6359909	390.240097	293.5019786	APY|BCAP|IC	MIM:607796	13	13q14.2	PHD finger pr	protein-codir	PHF11	PHD finger pr	O	PHD finger pr	20210302
STAG2	ENSG00000101972	10735	20021.84	2.50	4.27	1.93671E-05	0.001451462	1755.63319	1957.10648	5697.17815	4327.36116	192403.249	54896.4758	21937.0796	18418.7657	1616.45972	1482.28935	1616.94628	2038.51964	6678.42724	1740.4353	1941.8126	1841.6811	HPE13|MKM	MIM:300826	X	Xq25	stromal antig	protein-codir	STAG2	stromal antig	O	cohesin subu	20210313
C1orf21	ENSG00000116667	81563	1376.25	0.89	4.27	1.97295E-05	0.001472824	1142.45479	1427.03948	1488.06027	1909.5542	2592.44184	1452.58352	1992.4264	1914.10533	541.441366	915.785679	1304.60219	865.759791	1742.94447	531.9825877	547.262284	1651.604282	PIG13	HGNC:HGNC:	1	1q25.3	chromosome	protein-codir	C1orf21	chromosome	O	uncharacteri	20210302
ND5	ENSG00000198786	4540	52133.92	0.93	4.26	2.01512E-05	0.001495937	135041.766	124395.367	100133.443	46069.0281	26750.0601	47464.2422	35425.1247	29911.9094	108667.521	26850.923	46587.4294	21575.1083	20961.2134	19478.36964	19052.8905	25778.27781	MTND5	MIM:516005	MT	-	NADH dehyd	protein-codir	MT-ND5	mitochondria	O	NADH dehyd	20210407
THUMPD3-AS	ENSG00000206573	440944	621.37	0.79	4.26	2.08181E-05	0.001536021	739.717083	925.155712	1658.2952	631.822758	637.433283	623.789164	808.186741	416.626687	376.587054	434.827509	489.866357	377.950107	429.105551	551.4529917	550.823171	290.2268018	SETD5-AS1	HGNC:HGNC:	3	3p25.3	THUMPD3 an	ncRNA	THUMPD3-AS	THUMPD3 an	O	SETD5 antise	20210302
CCDC82	ENSG00000149231	79780	666.84	0.72	4.25	2.12125E-05	0.001559077	777.680618	594.473554	1201.63977	900.547142	1100.35764	748.878276	542.658365	753.628524	825.491783	333.570342	624.042613	462.916438	526.615835	388.8890186	468.99151	419.1340545	HSPC048	HGNC:HGNC:	11	11q21	coiled-coil do	protein-codir	CCDC82	coiled-coil do	O	coiled-coil do	20210302
C5AR1	ENSG00000197405	728	46.07	-4.17	-4.25	2.15369E-05	0.001570789	20.7929944	20.7920995	11.8889895	1.8687747	0	0	0	0	520.972851	18.9080709	12.5397263	6.15744196	24.4627519	10.16435706	19.3219725	69.25686204	C5A|C5AR|C	MIM:113995	19	19q13.32	complement	protein-codir	C5AR1	complement	O	C5a anaphyla	20210406
ABLIM1	ENSG00000099204	3983	2388.94	-0.94	-4.25	2.14857E-05	0.001570789	2623.63604	2189.80964	926.735215	799.822227	1414.07145	2264.04467	1544.91761	1482.60375	5797.83112	2028.3009	2080.98111	3318.96567	2644.9847	2840.896793	3841.51908	2423.959372	ABLIM|LIMAI	MIM:602330	10	10q25.3	actin binding	protein-codir	ABLIM1	actin binding	O	actin-binding	20210302
	ENSG00000260565		635.42	0.63	4.24	2.19193E-05	0.001592576	591.03084	592.706592	998.993875	928.412279	911.770908	788.177959	715.735671	578.560801	251.428675	358.924798	560.360056	571.353249	707.639465	704.439742	557.530884	349.6074452											
SH2D1B	ENSG00000198574	117157	223.63	1.21	4.24	2.20516E-05	0.001596101	63.9065013	44.3601553	1631.83413	73.986559	427.127299	39.0215931	251.503877	18.1176135	24.4981844	17.3933909	443.947304	19.0255951	400.900866	13.60497617	90.9122434	17.95069479	EAT2	MIM:608510	1	1q23.3	SH2 domain c	protein-codir	SH2D1B	SH2 domain c	O	SH2 domain-	20210302
	ENSG00000267469		28.10	-3.62	-4.24	2.24659E-05	0.001613811	5.75423737	7.96826672	0	0	5.33722377	5.43151928	0	0	230.973526	141.277809	4.05864896	1.13225839	11.6643275	10.23225992	6.0731248	19.69074429											
APPL2	ENSG00000136044	55198	512.61	0.69	4.24	2.24044E-05	0.001613811	396.84135	381.967815	703.089398	527.457731	1049.20636	673.576863	728.303828	578.920644	193.670698	422.46759	394.206317	312.603089	712.570379	435.4152676	386.51955	304.9584505	DIP13B	MIM:606231	12	12q23.3	adaptor prot	protein-codir	APPL2	adaptor prot	O	DCC-interacti	20210302
SNORA63	ENSG00000200320	6043	1070.96	-2.78	-4.23	2.29362E-05	0.0016387	4099.29447	2028.03028	19.310575	77.174385	65.1969698	129.560063	62.0495644	214.761493	1718.638	2035.95048	1576.01836	1512.54617	459.503384	878.1525475	980.542721	1278.670342	E3|E3-2|RNE	MIM:180647	3	3q27.3	small nucleol	snoRNA	SNORA63	small nucleol	O	RNA, U107 sr	20210302
IL6R	ENSG00000160712	3570	194.66	-2.49	-4.23	2.29845E-05	0.0016387	155.109979	363.054584	52.9724836	9.23356797	4.87332937	139.670089	54.3266113	8.54178431	209.191281	177.027852	134.479624	200.922422	401.42358	585.6024433	480.55546	137.6128818	CD126|HIES5	MIM:147880	1	1q21.3	interleukin 6	protein-codir	IL6R	interleukin 6	O	interleukin-6	20210418
PCDH9	ENSG00000184226	5101	20.75	2.10	4.23	2.32042E-05	0.001648191	69.4084215	9.28676667	21.2559559	7.13810671	60.1324438	12.4860422	21.3891491	66.7707223	24.7591991	4.85361514	3.35348011	1.57467909	10.08067	2.306461625	13.412121	3.869544703	-	MIM:603581	13	13q21.32	protocadheri	protein-codir	PCDH9	protocadheri	O	protocadheri	20210302
RNF157-AS1	ENSG00000267128	100507218	52.11	-4.29	-4.23	2.35421E-05	0.001664816	102.646559	89.0829432	0	0	4.57291815	54.5922256	0	0.23128685	97.9394321	30.1855551	46.2789535	55.0047673	69.0523278	84.02048686	146.687562	53.42954096	-	HGNC:HGNC:	17	17q25.1	RNF157 antis	ncRNA	RNF157-AS1	RNF157 antis	O	RNF157 antis	20210302
SDK2	ENSG00000069188	54549	78.82	-3.53	-4.23	2.37006E-05	0.001664816	148.831503	38.9174338	0	0	0	96.4071659	13.1085907	4.88759703	127.573409	56.5318682	94.905352	232.862062	89.6878476	145.7774104	111.090856	100.6087037	-	MIM:607217	17	17q25.1	sidekick cell a	protein-codir	SDK2	sidekick cell a	O	protein sidek	20210302
SCRN1	ENSG00000136193	9805	95.11	-1.95	-4.23	2.36415E-05	0.001664816	136.794132	53.8932923	38.296586	37.2302516	0	25.591122	51.9325113	9.04422863	396.495991	66.1379658	67.2960991	87.9803185	57.9882626	81.54028559	297.808293	113.7477307	SES1	MIM:614965	7	7p14.3	secernin 1	protein-codir	SCRN1	secernin 1	O	secernin-1	20210302
MEGF6	ENSG00000162591	1953	172.03	-2.19	-4.23	2.37948E-05	0.001665288	218.851569	108.315483	0	15.1329998	9.07193024	198.808986	99.7125842	50.6428013	234.991511	228.011219	115.541181	107.124782	295.883877	417.927851	530.757946	121.7348043	EGFL3	MIM:604266	1	1p36.32	multiple EGF	protein-codir	MEGF6	multiple EGF	O	multiple epid	20210302
PBXIP1	ENSG00000163346	57326	1056.73	-0.94	-4.22	2.44178E-05	0.00168412	933.838612	989.553008	331.841442	814.538916	342.952788	746.093178	673.79703	1134.32822	855.844699	1276.14427	1118.68047	1241.62274	1131.05103	1532.362815	1624.86726	2160.241353	HPIP	MIM:618819	1	1q21.3	PBX homeob	protein-codir	PBXIP1	PBX homeob	O	pre-B-cell leu	20210302
BCAP31	ENSG00000185825	10134	198.83	-0.69	-4.22	2.4384E-05	0.00168412	116.846537	208.131191	158.05605	99.3045919	149.341266	188.876318	128.343155	182.243202	178.100292	264.258091	211.107224	212.333886	230.086248	270.9945578	243.987547	339.1913206	6C6-AG|BAP3	MIM:300398	X	Xq28	B cell recepto	protein-codir	BCAP31	B cell recepto	O	B-cell recepto	20210418
CCNH	ENSG00000134480	902	733.89	0.72	4.22	2.42448E-05	0.00168412	625.854054	677.467363	836.230693	1368.85733	1326.32075	906.83921	855.587534	673.730174	273.51907	469.126929	766.379891	680.572298	787.941393	358.9317507	601.094958	533.7566614	CAK|CycH|p3	MIM:601953	5	5q14.3	cyclin H	protein-codir	CCNH	cyclin H	O	cyclin-H|CAK	20210302
NEUROD2	ENSG00000171532	4761	89.74	3.78	4.22	2.4228E-05	0.00168412	1.55805134	0	21.4741292	3.04445117	790.855216	282.354715	110.850401	98.3348392	60.4931462	0.76519673	0.49030291	0	52.1713659	4.911768239	1.91364543	6.633274361	DEE72|EIEE7	MIM:601725	17	17q12	neuronal diff	protein-codir	NEUROD2	neuronal diff	O	neurogenic d	20210417
	ENSG00000263934		10480.63	-1.00	-4.22	2.49086E-05	0.001711769	7140.58674	6230.34833	2816.86396	6962.71303	2894.94388	8253.87759	6583.02664	11877.5828	8629.54607	11138.2016	8524.22302	34326.7517	5891.25599	7885.15861	11967.2941	26567.73447											
NUTM2B-AS1	ENSG00000225484	101060691	445.16	0.77	4.21	2.58161E-05	0.001767755	351.071007	265.956728	845.310367	965.571801	409.259517	620.235901	526.128198	524.179229	195.07428	250.246007	411.556053	473.290726	171.81083	373.6751867	487.026478	252.1180565	OPML1	MIM:618639	10	10q22.3	NUTM2B ant	ncRNA	NUTM2B-AS1	NUTM2B ant	O	-	20210302
TPRG1L	ENSG00000158109	127262	538.83	-0.62	-4.20	2.62948E-05	0.001794075	519.327785	570.593636	306.784683	436.37325	395.711339	399.852276	403.676416	368.273154	605.00994	735.539902	580.248468	815.672974	515.031344	591.2742235	582.156935	795.7978931	FAM79A|h-m	MIM:611460	1	1p36.32	tumor protei	protein-codir	TPRG1L	tumor protei	O	tumor protei	20210302
	ENSG00000175841		14.44	-3.11	-4.20	2.64436E-05	0.001797789	2.35906134	41.1238324	0	1.78054063	0	1.54922132	3.75625309	1.66594824	13.7157577	35.4735223	21.3135702	24.1584769	18.4874128	13.29633655	30.676169	21.75617127											
MYH10	ENSG00000133026	4628	23.59	-3.56	-4.19	2.78169E-05	0.001881567	1.38900181	9.10597959	42.5718661	6.88277307	0	1.09275827	0.4382302	2.40598049	111.717596	79.0910053	3.16537175	35.6063133	28.8062494	25.70123574	10.9248815	18.47521552	NMMHC-IIB|	MIM:160776	17	17p13.1	myosin heavy	protein-codir	MYH10	myosin heavy	O	myosin-10|m	20210404
RPS28	ENSG00000233927	6234	1889.34	-0.94	-4.19	2.78736E-05	0.001881567	4187.2973	1728.10622	2137.73096	741.997047	310.735944	897.969331	876.199403	971.448722	3272.10265	3406.28487	2824.14745	1647.89058	1198.78407	2089.874534	1692.08872	2246.710055	DBA15|S28|	MIM:603685	19	19p13.2	ribosomal pro	protein-codir	RPS28	ribosomal pro	O	40S ribosoma	20210404
DSC1	ENSG00000134765	1823	54.77	-4.34	-4.19	2.83173E-05	0.001902596	71.6739206	121.910529	0	0	0	130.093182	0	0.60274784	45.8385022	77.3752796	83.999631	127.152705	20.7975835	131.0155219	38.7492543	27.08552222	CDHF1|DG2/	MIM:125643	18	18q12.1	desmocollin	protein-codir	DSC1	desmocollin	O	desmocollin-	20210302

SPON1	ENSG00000262655	10418	146.68	-2.30	-4.19	2.84472E-05	0.001902596	277.565438	223.278047	2.90170719	18.4752986	4.43471446	95.6493817	84.1112397	18.2588939	217.758207	219.42658	164.820798	218.384594	114.702302	249.6887538	288.711315	148.7703172	VSGP/F-spon	MIM:604989	11	11p15.2	spondin 1	protein-codir	SPON1	spondin 1	O	spondin-1|sp	20210329
	ENSG00000274712		162.09	0.79	4.19	2.8485E-05	0.001902596	211.66349	211.291843	201.161368	160.259106	203.444892	225.312955	250.443778	166.982453	68.6890316	122.821588	119.382026	84.1601888	167.885803	152.8949772	142.787379	104.2156584											
APP	ENSG00000142192	351	96.46	-1.52	-4.18	2.91393E-05	0.001932738	124.482606	56.3622895	57.9792309	27.6788162	21.8587049	24.0625698	37.3910666	18.2910717	542.594175	77.4144052	55.1365446	139.70556	66.5306744	104.2677383	103.292787	86.31027773	AAA|ABETA|	MIM:104760	21	21q21.3	amyloid beta	protein-codir	APP	amyloid beta	O	amyloid-beta	20210418
RPL9	ENSG00000163682	6133	2950.92	-0.66	-4.18	2.9644E-05	0.001952607	2579.23002	2638.48706	3666.77784	3034.49838	857.731353	2212.49898	1913.09701	1505.40104	4636.07788	3181.31405	3922.42052	6289.16143	1601.68571	2486.403984	3778.95606	2910.98471	L9|NPC-A-16	MIM:603686	4	4p14	ribosomal pr	protein-codir	RPL9	ribosomal pr	O	60S ribosoma	20210404
SNORD116-3	ENSG00000207014	100033415	146.53	-2.76	-4.17	3.02464E-05	0.001978593	504.17419	325.597645	0	6.24498148	0	21.7609729	13.0457711	25.3793131	402.213117	286.914989	146.703603	290.154793	33.4058913	75.40431003	100.874022	112.5535453	HBII-85-3	HGNC:HGNC:	15	15q11.2	small nucleol	snoRNA	SNORD116-3	small nucleol	O	-	20210302
	ENSG00000235852		8.74	-4.14	-4.16	3.12078E-05	0.00203449	1.78740162	0	0	0	0	0.95324979	0	2.31487201	39.3671344	43.5373458	1.3848019	2.43049461	8.47779545	16.34425457	14.1642755	9.094019579											
	ENSG00000257386		20.37	-4.16	-4.16	3.16354E-05	0.002041394	5.23347268	1.37123626	0	0	0	0	0	0	228.880495	71.8020206	6.35301679	0	1.68714824	5.529947721	2.64125077	2.422985142											
RNF157	ENSG00000141576	114804	187.19	-1.74	-4.16	3.15209E-05	0.002041394	605.641093	108.666525	44.497846	29.3677124	66.6076154	76.1260875	25.8311863	52.5559906	342.161968	262.657254	163.588563	247.446332	139.646552	306.1061821	374.629666	149.5749666	-	HGNC:HGNC:	17	17q25.1	ring finger pr	protein-codir	RNF157	ring finger pr	O	E3 ubiquitin l	20210302
MALAT1	ENSG00000251562	378938	273815.19	0.73	4.16	3.1863E-05	0.002049134	309604.75	286128.667	211879.325	347143.724	456748.675	370112.323	382726.23	336040.917	95090.1365	156814.667	191443.182	163505.092	306666.713	257433.9054	235803.368	273901.324	HCN|LINC00(	MIM:607924	11	11q13.1	metastasis as	ncRNA	MALAT1	metastasis as	O	hepcarcin|lo	20210418
LDHB	ENSG00000111716	3945	1528.79	-1.10	-4.16	3.21781E-05	0.002059424	2008.8443	1659.25844	1273.31676	630.252723	321.006125	1297.18056	572.397796	609.057071	3075.60057	1755.46029	1202.34019	2660.94351	1072.50971	2462.144331	2104.87074	1755.532864	HEL-S-281|LD	MIM:150100	12	12p12.1	lactate dehyc	protein-codir	LDHB	lactate dehyc	O	L-lactate deh	20210404
TRPS1	ENSG00000104447	7227	369.63	0.97	4.16	3.22394E-05	0.002059424	464.569461	330.356953	658.061692	740.919614	438.001319	623.491899	426.001577	274.425017	295.924385	243.012161	259.056275	246.94218	223.973374	149.9390631	321.350263	218.0130108	GC79|LGCR	MIM:604386	8	8q23.3	transcription	protein-codir	TRPS1	transcription	O	zinc finger tra	20210410
PPAN	ENSG00000130810	56342	139.81	0.91	4.16	3.24458E-05	0.00206568	154.418158	215.656369	279.755607	155.004439	200.085774	137.544474	167.385434	157.574088	82.7906742	78.9094637	90.9598546	103.939281	110.818768	113.521723	118.465881	70.10760945	BXDC3|SSF|S	MIM:607793	19	19p13.2	peter pan ho	protein-codir	PPAN	peter pan ho	O	suppressor of	20210302
TSHZ2	ENSG00000182463	128553	89.52	-3.00	-4.15	3.26832E-05	0.00207386	125.470043	181.627191	0	6.93718283	0	108.652734	10.1215977	2.00912426	272.109207	103.979626	89.2956556	239.00176	74.0891966	124.8377181	52.155804	42.05456606	C20orf17|OV	MIM:614118	20	20q13.2	teashirt zinc f	protein-codir	TSHZ2	teashirt zinc f	O	teashirt hom	20210302
SIAH3	ENSG00000215475	283514	13.23	-3.26	-4.14	3.4033E-05	0.002152333	7.52483312	2.75086525	0	0	0	0.8315716	6.05261862	4.98539139	10.8423149	17.1374938	11.4185677	15.1106161	46.4967308	8.240439211	50.9393784	29.27183367	-	MIM:615609	13	13q14.13	siah E3 ubiqu	protein-codir	SIAH3	siah E3 ubiqu	O	seven in abse	20210302
	ENSG00000265907		24.48	-3.40	-4.14	3.42213E-05	0.002153419	7.27417046	30.7175782	0	0	0	0	0	1.48599433	191.807993	119.25337	1.29037911	0	8.41549044	7.262635169	2.81709824	21.32582173											
ITGB1	ENSG00000150093	3688	4050.18	0.77	4.14	3.42764E-05	0.002153419	2138.94993	3190.88806	3163.32999	7873.32951	6571.81609	4431.3456	5227.2155	8022.7777	1208.47713	3214.2957	1923.57543	3532.69618	4328.34587	2976.783931	1521.17611	5477.835162	CD29|FNRB|	MIM:135630	10	10p11.22	integrin subu	protein-codir	ITGB1	integrin subu	O	integrin beta-	20210418
CNTRL	ENSG00000119397	11064	1412.45	0.76	4.14	3.45084E-05	0.002160862	2812.39005	1547.67729	2731.27849	1231.307	1299.23962	1887.78438	1378.74864	1514.58516	1008.24252	1029.01965	1026.50662	890.413564	1073.811	1276.132722	1168.94349	723.189001	CEP1|CEP11(	MIM:605496	9	9q33.2	centriolin	protein-codir	CNTRL	centriolin	O	centriolin|11	20210404
SH3RF3	ENSG00000172985	344558	51.88	-2.82	-4.14	3.53262E-05	0.002204821	85.4172561	38.7160462	11.2965378	1.59402832	0	44.9790473	5.38656074	2.11679343	131.028949	73.3381256	38.0980823	74.7411389	89.3978939	56.53189752	142.806826	34.55574805	POSH2|SH3M	MIM:618933	2	2q13	SH3 domain c	protein-codir	SH3RF3	SH3 domain c	O	E3 ubiquitin-p	20210302
BICDL1	ENSG00000135127	92558	254.38	-1.29	-4.13	3.59535E-05	0.002236638	355.634307	310.933214	40.1263681	99.197369	70.6028635	183.482343	118.967279	108.646963	679.16398	372.260509	403.97908	211.936562	256.336978	270.9702845	374.729151	213.1497926	BICDR-1|BICI	MIM:617002	12	12q24.23	BICD family li	protein-codir	BICDL1	BICD family li	O	BICD family-li	20210302
MX1	ENSG00000157601	4599	273.42	-1.00	-4.13	3.62246E-05	0.002246163	187.031373	195.691933	115.769122	164.16022	128.262492	252.07945	96.1487064	235.232866	983.320129	317.775742	259.956647	311.210798	195.164002	350.3684628	237.004151	345.5365124	IFI-78K|IFI78	MIM:147150	21	21q22.3	MX dynamin	protein-codir	MX1	MX dynamin	O	interferon-in	20210329
GNB5	ENSG00000069966	10681	227.94	-0.73	-4.13	3.70481E-05	0.002289764	156.744704	184.444215	108.621239	300.163616	79.2892464	176.70146	200.173064	198.148235	300.144596	292.810477	204.597317	346.60384	186.058987	351.0404486	316.332096	245.1132048	GB5|IDDCA|I	MIM:604447	15	15q21.2	G protein sub	protein-codir	GNB5	G protein sub	O	guanine nucle	20210307
ZEB2	ENSG00000169554	9839	3453.21	1.06	4.12	3.83262E-05	0.002361092	1323.75569	1785.7745	3701.40882	9450.90837	8100.16546	3828.58201	5933.31384	3973.12286	933.954911	1678.77668	2866.46118	2420.54848	4187.40705	963.9550692	1440.85151	2662.394899	HSPC082|SIP	MIM:605802	2	2q22.3	zinc finger E-l	protein-codir	ZEB2	zinc finger E-l	O	zinc finger E-l	20210418
EMC1	ENSG00000127463	23065	261.99	-0.81	-4.11	3.91083E-05	0.0024015	176.848739	165.344245	98.3315846	320.478894	200.375688	163.374235	155.7403	286.316603	424.963876	319.215735	281.795445	363.306028	320.664174	262.2132982	309.453181	343.4962085	CAVIPMR|KIA	MIM:616846	1	1p36.13	ER membran	protein-codir	EMC1	ER membran	O	ER membran	20210307
TCF7	ENSG00000081059	6932	3140.68	-1.58	-4.10	4.07684E-05	0.002479451	7958.99342	4165.62635	1329.99464	967.781427	199.339965	2405.93875	697.32559	574.991456	5176.11575	3677.32017	3530.12115	5606.93634	2325.31522	5053.115932	3944.27712	2637.736314	TCF-1	MIM:189908	5	5q31.1	transcription	protein-codir	TCF7	transcription	O	transcription	20210302
	ENSG00000244720		117.81	0.99	4.10	4.06661E-05	0.002479451	59.5769617	114.409583	291.005012	80.3530737	205.780477	256.744896	110.066968	142.014991	26.2275456	53.3229004	94.6457284	37.0844078	130.23612	101.3040794	111.49717	70.61502376											
NOG	ENSG00000183691	9241	70.73	-3.39	-4.10	4.15268E-05	0.002509539	63.3149451	72.7514008	14.5263085	0	0	50.8121022	12.5344091	0	137.675105	105.960636	29.9767054	149.597858	99.313353	175.7846853	148.303257	71.14473228	SYM1|SYNS1	MIM:602991	17	17q22	noggin	protein-codir	NOG	noggin	O	noggin|symp	20210302
GANAB	ENSG00000089597	23193	838.79	-0.80	-4.10	4.14992E-05	0.002509539	755.669755	783.187757	330.413222	545.887996	706.147607	555.987264	587.778236	674.945379	723.413425	1022.23087	1020.33022	841.964759	1070.86294	1292.899113	1211.54862	1297.295485	G2AN|GIIA|(	MIM:104160	11	11q12.3	glucosidase II	protein-codir	GANAB	glucosidase II	O	neutral alpha	20210410
AFF2	ENSG00000155966	2334	70.29	-2.44	-4.09	4.23138E-05	0.002549006	95.2819226	3.162021	11.8266049	6.76487123	0.87006855	7.72606592	13.533009	0.6844323	794.601253	14.8238694	2.65445145	57.0369002	22.4669518	32.16495998	52.1916895	8.907987328	FMR2|FMR2I	MIM:300806	X	Xq28	AF4/FMR2 fa	protein-codir	AFF2	AF4/FMR2 fa	O	AF4/FMR2 fa	20210416
	ENSG00000261553		46.17	-2.65	-4.09	4.27764E-05	0.002568741	1.73151301	78.6021883	8.88427282	10.6752028	12.0220396	4.32645647	0	13.0665318	14.5779211	174.676947	12.4605475	176.40527	27.2852321	34.76894747	98.9328416	70.32621843											
CX3CR1	ENSG00000168329	1524	1175.75	1.21	4.09	4.33141E-05	0.002592853	334.220156	457.570716	2609.32956	2797.30666	1095.40703	1713.54635	2271.29633	1976.84367	139.194166	523.432366	615.615751	1071.69457	1043.31201	432.6042559	435.096111	1295.52767	CCRL1|CMKB	MIM:601470	3	3p22.2	C-X3-C motif	protein-codir	CX3CR1	C-X3-C motif	O	CX3C chemok	20210418
	ENSG00000245904		80.61	-1.81	-4.09	4.37938E-05	0.002598619	77.6160205	173.055179	19.7205316	23.7987784	5.95414815	24.5157497	9.11060569	32.9318577	200.211746	282.513153	33.0527021	59.4196508	97.8837106	105.2013338	92.3828781	52.38853033											
ST3GAL5	ENSG00000115525	8869	398.36	0.70	4.09	4.382E-05	0.002598619	228.070842	356.344297	714.594459	544.311679	762.150787	309.133345	566.17599	480.548109	149.809111	310.470644	332.360838	365.059442	486.043205	211.0817626	211.199188	346.4228297	SATI|SIAT9|S	MIM:604402	2	2p11.2	ST3 beta-gala	protein-codir	ST3GAL5	ST3 beta-gala	O	lactosylceram	20210327
TCP11L2	ENSG00000166046	255394	789.67	-0.80	-4.08	4.55168E-05	0.002690864	1112.4158	1110.29921	507.245266	414.592682	389.18324	532.374789	428.79943	335.560373	1448.99716	951.563637	920.082601	965.94208	619.685647	1002.782951	1139.67631	755.5910062	-	HGNC:HGNC:	12	12q23.3	t-complex 11	protein-codir	TCP11L2	t-complex 11	O	T-complex pr	20210302
MORC4	ENSG00000133131	79710	85.86	-1.55	-4.08	4.568E-05	0.002692149	53.3604084	70.6760391	10.8056293	19.4987922	33.1060439	50.3188491	31.2057157	53.9011531	423.677927	84.0147078	91.7278448	69.5639053	58.3126073	117.1674475	126.022815	80.33880987	ZCW4|ZCWC	MIM:300970	X	Xq22.3	MORC family	protein-codir	MORC4	MORC family	O	MORC family	20210302
ATP8A1	ENSG00000124406	10396	2475.66	0.63	4.07	4.65033E-05	0.002723804	2239.56776	2175.37206	2969.80933	4268.0694	3382.90877	2820.44146	3116.67751	2875.82182	1072.07428	2046.18565	3056.06969	2507.73081	2108.10591	1507.519974	1747.19659	1716.996374	ATPASEII|ATI	MIM:609542	4	4p13	ATPase phos	protein-codir	ATP8A1	ATPase phos	O	phospholipid	20210302
IGKC	ENSG00000211592	3514	21.84	-3.56	-4.07	4.76967E-05	0.002785138	7.58448662	2.69359333	0	1.7116996	9.42750488	0	0	0	206.34767	6.48236275	0	8.74418919	76.8526946	2.863773796	17.5730943	9.114273125	HCAK1|IGKCI	MIM:147200	2	2p11.2	immunoglob	other	IGKC	immunoglob	O	immunoglob	20210322
TOMM22	ENSG00000100216	56993	135.15	-1.25	-4.07	4.78698E-05	0.002786697	161.605815	180.659684	63.4947372	57.3340696	37.8942361	57.7924842	34.4350999	114.009806	194.170842	196.899677	108.597828	206.096526	144.143479	173.1774725	177.297874	254.7381011	1C9-2|MST0(	MIM:607046	22	22q13.1	translocase o	protein-codir	TOMM22	translocase o	O	mitochondria	20210404
ADD2	ENSG00000075340	119	27.13	-4.12	-4.06	4.88242E-05	0.002833592	130.238576	17.3497453	0	0	0	2.38135124	0	3.15950903	23.4527425	33.277336	20.9070095	59.8186498	28.0231078	30.09395253	56.0555876	29.33856299	ADDB	MIM:102681	2	2p13.3	adducin 2	protein-codir	ADD2	adducin 2	O	beta-adducin	20210302
LAMP3	ENSG00000078081	27074	10.13	-4.22	-4.06	4.96616E-05	0.002873426	0.81158539	0	0	0	0	8.8666333	5.54893591	0.46558646	37.5207642	22.4943274	7.77569382	18.5764111	26.2156588	6.507996934	14.7380853	12.5694376	CD208|DC LA	MIM:605883	3	3q27.1	lysosomal ass	protein-codir	LAMP3	lysosomal ass	O	lysosome-ass	20210329
NBEA	ENSG00000172915	26960	149.87	-2.35	-4.06	5.00207E-05	0.002885438	165.266047	276.993793	91.8227612	12.0741459	1.79080053	82.6141969	11.46703	4.24561504	920.091963	122.920333	97.8245727	160.177055	90.4111636	145.6897649	155.826134	58.69681747	BCL8B|LYST2	MIM:604889	13	13q13.3	neurobeachir	protein-codir	NBEA	neurobeachir	O	neurobeachir	20210417
SSR2	ENSG00000163479	6746	666.34	-0.70	-4.05	5.11366E-05	0.002940898	575.199085	597.566797	224.708907	569.31181	418.037401	538.071718	576.137898	595.37374	851.337394	689.809547	656.386561	1194.11116	640.756034	727.851368	743.872905	1062.924213	HSD25|TLAP	MIM:600867	1	1q22	signal sequer	protein-codir	SSR2	signal sequer	O	translocon-as	20210307
	ENSG00000176868		45.73	-3.35	-4.05	5.20587E-05	0.002975942	0	4.2025799	0	0	0	29.9694943	4.8177666	10.9100727	10.8139116	220.913619	279.663332	5.27806898	8.08040793	49.2290725	50.97880241	20.4332974	36.33657109										
SNORD116-1	ENSG00000207263	100033428	34.42	-3.08	-4.05	5.20038E-05	0.002975942	65.1397515	64.0617279	1.45193462	5.28216641	0	7.51716197	0	5.04771847	71.6017292	66.3396642	72.849107	63.7390147	17.9946986	21.75129178	37.5936466	50.31628148	HBII-85-16	HGNC:HGNC:	15	15q11.2	small nucleol	snoRNA	SNORD116-1	small nucleol	O	-	20210302
EYA3	ENSG00000158161	2140	307.33	0.62	4.04	5.26671E-05	0.003001707	295.623274	415.723563	368.133676	343.269879	419.509644	395.62783	385.4061	349.550807	250.398934	192.146839	258.030761	264.464164	209.64144	277.6615723	271.042533	221.1032635	-	MIM:601655	1	1p35.3	EYA transcrip	protein-codir	EYA3	EYA transcrip	O	eyes absent h	20210302
CYP1B1	ENSG00000138061	1545	40.22	-4.29	-4.04	5.43181E-05	0.003077382	8.25397253	0	0	0	27.0864304	0	9.02813449	0	14.6342928	15.7515499	37.0483237	74.3398914	445.9141	6.840389726	1.49307095	3.163089997	ASGD6|CP1B	MIM:601771	2	2p22.2	cytochrome P	protein-codir	CYP1B1	cytochrome P	O	cytochrome P	20210410
	ENSG00000235944		61.22	-1.97	-4.04	5.45781E-05	0.003082324	85.9930371	14.6736584	0	13.9154221	4.48635503	78.1649284	27.0407554	34.1632373	75.1767694	58.5064872	31.1621624	109.772086	50.120545	170.7769229	163.383107	62.18636871											
RPS3	ENSG00000149273	6188	5720.08	-0.67	-4.03	5.47292E-05	0.003082324	9067.09677	9001.48207	3224.27178	2966.9551	2811.80851	3712.4933	2874.18179	3477.13796	8633.12926	10334.6703	4913.25423	6819.89973	4301.63276	5931.027112	5995.67894	7456.640247	S3	MIM:600454	11	11q13.4	ribosomal pr	protein-codir	RPS3	ribosomal pr	O	40S ribosoma	20210417
SSTR3	ENSG00000278195	6753	16.23	-3.16	-4.03	5.51961E-05	0.00309945	26.6941465	6.20355634	0	0	0	6.56794606	1.63481588	3.64079691	27.6047315	20.7730939	9.71928079	15.2683171	26.0549787	22.25265359	54.2862087	39.00838491	SS-3-R|SS3-R	MIM:182453	22	22q13.1	somatostatin	protein-codir	SSTR3	somatostatin	O	somatostatin	20210302
CNTNAP1	ENSG00000108797	8506	13.46	-3.78	-4.03	5.56193E-05	0.003114027	10.6613006	1.50490149	3.88680214	0	0	0.48917458	0	2.28298041	69.7347558	42.6628297	1.18015787	1.82804046	19.1714503	31.94413228	10.0343966	19.99114592	CASPR|CHN3	MIM:602346	17	17q21.2	contactin ass	protein-codir	CNTNAP1	contactin ass	O	contactin-ass	20210410
KLRC2	ENSG00000205809	3822	314.25	1.31	4.03	5.60151E-05	0.00312699	82.8437177	85.06379	2639.34188	179.978575	96.4909097	297.99057	335.560253	169.634854	72.018293	79.1610268	571.14723	126.773234	11.1129325	100.8598026	107.549897	72.48086352	CD159c|NKG	MIM:602891	12	12p13.2	killer cell lect	protein-codir	KLRC2	killer cell lect	O	NKG2-C type	20210316
	ENSG00000272912		56.38	3.45	4.03	5.68898E-05	0.00316653	15.9723961	0	7.88447253	0	481.084872	186.258797	73.4567904	84.5916555	1.90797475	2.7673637	2.62488602	0	36.2066965	4.693608434	0	4.690713975											
BTF3L4	ENSG00000134717	91408	352.44	-0.83	-4.02	5.75382E-05	0.003193288	366.684669	332.054561	143.582125	270.209482	215.090413	140.774029	330.03577	228.313911	858.648312	354.71274	398.837592	542.470065	315.336405	379.9840116	406.644524	355.7275797	-	HGNC:HGNC:	1	1p32.3	basic transcri	protein-codir	BTF3L4	basic transcri	O	transcription	20210302
MAGI2-AS3	ENSG00000234456	100505881	64.31	-2.13	-4.02	5.78E-05	0.003198489	15.2352381	44.8931514	48.6930844	0.66365967	59.7391662	61.8263917	16.7145045	3.38861925	138.156956	113.662776	53.8356079	47.4106681	235.690785	99.44642742	51.4612551	38.15633613	-	HGNC:HGNC:	7	7q21.11	MAGI2 antise	ncRNA	MAGI2-AS3	MAGI2 antise	O	MAGI2 antise	20210316
	ENSG00000277947		593.05	-1.45	-4.02	5.88611E-05	0.003243595	78.3528238	66.2746154	10.0154714	29.336427	181.443922	446.446654	352.433176	874.579328	67.4115409	46.7862814	30.6189315	187.80645	858.824793	1114.365572	1602.17262	3541.851973											
PLOD1	ENSG00000083444	5351	55.33	1.24	4.02	5.89559E-05	0.003243595	25.234296	52.9015359	92.2179091	55.1130008	152.245202	43.6804327	88.8250429	113.038577	19.3062845	28.8485955	27.0225484	36.7467688	66.7566936	14.06879059	18.4352728	50.91312172	EDS6|EDSKCI	MIM:153454	1	1p36.22	procollagen-l	protein-codir	PLOD1	procollagen-l	O	procollagen-l	20210410
	ENSG00000279364		19.30	-3.95	-4.01	5.94552E-05	0.003252265	1.26616657	6.00533532	0	0	0	0	0	0.62514201	125.256871	151.23702	0.80947016	0	1.62354859	5.988085279	5.33372731	10.69652347											
BTN3A1	ENSG00000026950	11119	2197.79	0.79	4.02	5.9333E-05	0.003252265	3352.90664	2162.5296	2602.62575	2103.22665	3302.705	2370.892	2960.84726	3130.25024	943.348644	1409.10142	2288.49331	1008.99148	2456.96064	1976.371695	1802.22201	1293.216142	BT3.1|BTF5|I	MIM:613593	6	6p22.2	butyrophilin s	protein-codir	BTN3A1	butyrophilin s	O	butyrophilin s	20210302
SCARNA6	ENSG00000251791	677772	1317.55	-1.20	-4.01	5.99143E-05	0.003267988	1526.36409	1149.34075	517.403194	658.752773	210.102923	1159.03419	652.268712	721.475466	1139.49643	1808.4292	2316.75976	3540.55554	678.468535	1313.455716	1654.92409	2034.041615	U88	MIM:615641	2	2q37.1	small Cajal bo	ncRNA	SCARNA6	small Cajal bo	O	-	20210302
ATP6V0E2-AS	ENSG00000204934	401431	16.36	-2.56	-4.01	6.15024E-05	0.003341694	18.2457275	6.39604095	0	0	2.08395291	9.02359002	3.76848763	7.54964107	27.9403787	32.3034455	8.43187277	21.5784779	14.1260701	27.64107093	52.628788	30.00948045	-	HGNC:HGNC:	7	7q36.1	ATP6V0E2 an	ncRNA	ATP6V0E2-AS	ATP6V0E2 an	O	ATP6V0E2 an	20210302
MID1IP1	ENSG00000165175	58526	175.71	-1.42	-4.01	6.16167E-05	0.003341694	128.221663	130.727367	8.31604767	19.3618497	166.073073	222.167711	114.42528	111.506139	375.983425	299.197829	96.6385024	156.551826	191.300145	323.9117511	267.24672	199.7151369	G12-like|MIG	MIM:300961	X	Xp11.4	MID1 interac	protein-codir	MID1IP1	MID1 interac	O	mid1-interact	20210307
KLF7	ENSG00000118263	8609	335.09	-1.59	-4.01	6.19285E-05	0.003349063	759.955564	232.912945	43.1512802	89.2640682	53.406528	691.33554	40.6011464	35.1170455	950.916467	322.395152	327.850013	423.853711	227.878406	589.8315781	475.701543	97.31709362	UKLF	MIM:604865	2	2q33.3	Kruppel like f	protein-codir	KLF7	Kruppel like f	O	Krueppel-like	20210302
LOC647132	ENSG00000230987	647132	8.81	4.58	4.00	6.26904E-05	0.003380663	0	1.56469563	38.3988034	5.05694046	76.2804754	7.11209831	3.08914592	6.8896109	0	0.72815098	0.76088526	0	1.05576181	0	0	0	-	Ensembl:ENS	1	1q31.1	DExH-box hel	pseudo	-	-	-	DEAH (Asp-G	20210302
PAWR	ENSG00000177425	5074	73.20	-3.03	-4.00	6.4084E-05	0.003436346	11.4192636	14.831755	0	0	0	14.8793734	3.72236352	5.18114743	969.859457	14.6161541	5.43963569	16.8511018	23.5942854	50.59519788	30.2117126	9.987119608	PAR4|Par-4	MIM:601936	12	12q21.2	pro-apoptotic	protein-codir	PAWR	pro-apoptotic	O	PRKC apopto	20210329
INPP5D	ENSG00000168918	3635	1706.49	0.73	4.00	6.43236E-05	0.003439505	1918.00156	1388.98286	3961.13773	1673.7756	2078.15002	1769.51674	1954.73706	2406.90424	813.290531	1100.12314	1343.9019	1265.41797	2029.08574	1121.907496	1160.63259	1318.240059	SHIP|SHIP-1|	MIM:601582	2	2q37.1	inositol polyp	protein-codir	INPP5D	inositol polyp	O	phosphatidyl	20210302
ARRDC2	ENSG00000105643	27106	441.62	-0.82	-3.99	6.50822E-05	0.00346114	301.1454	438.708358	137.761069	163.219826	327.616041	450.067622	417.004858	350.664527	721.268968	931.851774	342.415536	417.931048	647.360901	513.3025929	503.220633	402.3074703	CLONE24945	HGNC:HGNC:	19	19p13.11	arrestin dom	protein-codir	ARRDC2	arrestin dom	O	arrestin dom	20210302
CD8B	ENSG00000172116	926	1080.73	-0.67	-3.99	6.50918E-05	0.00346114	849.199893	1193.78236	540.893448	833.704382	585.927099	1262.91639	548.289481	870.326794	956.59247	1414.9779	784.296091	1905.26096	1057.00951	1501.550599	1109.999	1876.970476	CD8B1|LEU2	MIM:186730	2	2p11.2	CD8b molecu	protein-codir	CD8B	CD8b molecu	O	T-cell surface	20210302
FLNB	ENSG00000136068	2317	448.95	-1.05	-3.99	6.58794E-05	0.003493259	953.03284	375.057468	204.525935	210.248234	110.670572	468.185583	111.486888	175.838652	1101.28564	518.350047	404.808493	754.854616	288.694521	611.5132788	573.211619	321.3753204	ABP-278|ABF	MIM:603381	3	3p14.3	filamin B	protein-codir	FLNB	filamin B	O	filamin-B|AB	20210410
ZNF835	ENSG00000127903	90485	8.46	-5.03	-3.99	6.63404E-05	0.003507933	4.69785862	4.88831419	0	0	0	0	0	0	15.9362901	2.95579659	34.9662611	14.0703248	20.1231255	8.845135345	16.595287	12.24178862	BC37295_3	HGNC:HGNC:	19	19q13.43	zinc finger pr	protein-codir	ZNF835	zinc finger pr	O	zinc finger pr	20210302
UBAC2	ENSG00000134882	337867	501.10	-0.89	-3.98	6.78878E-05	0.00354498	580.375153	423.368334	159.479818	257.691761	375.396267	503.823091	221.913599	402.949871	539.918895	863.84378	476.508925	753.507468	522.645105	693.5964741	601.675627	640.8744202	PHGDHL1	HGNC:HGNC:	13	13q32.3	UBA domain	protein-codir	UBAC2	UBA domain	O	ubiquitin-ass	20210307
SMC1A	ENSG00000072501	8243	1045.54	0.65	3.98	6.79721E-05	0.00354498	951.616094	864.714342	1330.56863	851.805124	1568.46696	1612.38534	1387.25436	1485.09581	705.512108	436.268418	494.227196	526.510662	1211.70152	920.7190077	1262.34408	1119.415224	CDLS2|DEE8	MIM:300040	X	Xp11.22	structural ma	protein-codir	SMC1A	structural ma	O	structural ma	20210410
FAM76B	ENSG00000077458	143684	442.41	0.67	3.98	6.78078E-05	0.00354498	655.708111	469.883261	867.361889	697.658004	403.608359	405.769249	419.75061	456.560819	260.23778	420.916368	461.729026	388.099251	243.108826	342.8243443	315.747829	269.6002816	-	HGNC:HGNC:	11	11q21	family with se	protein-codir	FAM76B	family with se	O	protein FAM7	20210302
ACAD11	ENSG00000240303	84129	159.69	0.86	3.98	6.75771E-05	0.00354498	234.33445	124.097288	264.125308	223.315461	225.068195	181.983985	198.052326	185.819936	70.5878543	104.78778	190.622068	102.787276	108.711668	117.0031781	135.681294	88.02611544	ACAD-11	MIM:614288	3	3q22.1	acyl-CoA deh	protein-codir	ACAD11	acyl-CoA deh	O	acyl-CoA deh	20210302
TBC1D19	ENSG00000109680	55296	183.69	1.14	3.98	6.77846E-05	0.00354498	202.950635	177.221822	531.437204	187.916296	417.151186	215.354998	156.661322	172.977734	49.9993654	148.385181	129.961578	112.686502	103.728331	86.08838369	121.09063	125.508618	-	HGNC:HGNC:	4	4p15.2	TBC1 domain	protein-codir	TBC1D19	TBC1 domain	O	TBC1 domain	20210302
ZBTB37	ENSG00000185278	84614	1759.38	0.64	3.98	6.83662E-05	0.003555788	1811.04596	1336.58441	2191.11999	2423.89184	3516.46099	1529.22844	2319.39679	2081.60387	879.706798	1244.25605	1637.043	1431.70631	1911.28652	1445.326314	1468.65377	922.7087765	D430004I08R	HGNC:HGNC:	1	1q25.1	zinc finger an	protein-codir	ZBTB37	zinc finger an	O	zinc finger an	20210417
USP28	ENSG00000048028	57646	534.56	0.92	3.98	6.86283E-05	0.003559696	200.139409	407.450962	1552.49846	586.375515	942.896804	633.001018	818.444561	618.343425	199.733295	319.905867	512.094246	407.717716	436.044604	169.7640367	334.725558	413.8411889	-	MIM:610748	11	11q23.2	ubiquitin spe	protein-codir	USP28	ubiquitin spe	O	ubiquitin carl	20210418
LOC728554	ENSG00000170089	728554	87.79	-1.46	-3.98	6.93056E-05	0.003585061	76.6429167	66.3509672	28.2983224	11.8323458	29.0698959	89.3906358	26.1341439	84.1519289	197.857466	113.869024	82.0325994	85.8933852	152.925035	138.8915242	139.969174	81.28136938	-	Ensembl:ENS	5	5q35.3	THO complex	pseudo	-	-	-	-	20210302
LINGO3	ENSG00000220008	645191	24.46	-2.75	-3.97	7.06165E-05	0.003642971	10.024291	11.0694514	0	0	0	42.785423	2.63792719	14.4167922	75.285123	100.483634	8.15164314	10.2044128	25.6401754	35.71836666	24.3676494	30.61518714	LERN2|LRRN	MIM:609792	19	19p13.3	leucine rich r	protein-codir	LINGO3	leucine rich r	O	leucine-rich r	20210302
SNORD116-1	ENSG00000206656	100033429	36.35	-2.99	-3.97	7.24732E-05	0.00372865	111.267735	44.8565286	0	4.16094155	0	4.73721865	0	8.3501438	65.2664551	120.59539	65.2700089	67.9429092	7.56001817	25.13020385	20.9988691	35.45606107	HBII-85-17	HGNC:HGNC:	15	15q11.2	small nucleol	snoRNA	SNORD116-1	small nucleol	O	-	20210302
CSF1R	ENSG00000182578	1436	17.64	-3.70	-3.97	7.30106E-05	0.003731756	4.04854667	0	0	0	46.2430488	0	0	0.27112284	128.093503	6.99368082	1.11015912	5.41503733	76.0255676	3.291644101	2.53353828	8.145577812	BANDDOS|C-	MIM:164770	5	5q32	colony stimul	protein-codir	CSF1R	colony stimul	O	macrophage	20210410
SREK1	ENSG00000153914	140890	1354.28	0.69	3.97	7.29085E-05	0.003731756	1957.31062	1949.02998	1354.32386	2140.00807	1359.31332	1671.56309	1517.87742	1364.86106	712.875825	1123.63779	1347.23796	1135.52737	972.108379	1175.387417	1222.24763	665.2240801	SFRS12|SRrp	MIM:609268	5	5q12.3	splicing regul	protein-codir	SREK1	splicing regul	O	splicing regul	20210307
	ENSG00000205176		45.57	4.48	3.97	7.31217E-05	0.003731756	12.1526489	6.17708862	139.801104	9.70327261	12.585576	4.13855552	2.76327122	52.3975417	481.059026	6.43302219	1.20696732	0	0.6323304	0	0	0											
ANKK1	ENSG00000170209	255239	5.85	-4.24	-3.96	7.36927E-05	0.003740837	0	0.75375817	0	0	0	0	2.026004	0.93502357	5.0682312	22.4014226	11.43447	10.9197211	9.33454853	13.32497264	13.7505187	3.634766186	PKK2|sgK288	MIM:608774	11	11q23.2	ankyrin repea	protein-codir	ANKK1	ankyrin repea	O	ankyrin repea	20210329
CEP128	ENSG00000100629	145508	149.63	0.79	3.96	7.35526E-05	0.003740837	221.80247	192.146921	213.90332	241.54614	104.587875	85.7375815	243.750262	201.45779	80.7479427	142.615655	180.128801	124.101985	57.758076	53.82442886	108.646043	141.3328662	C14orf145|C	HGNC:HGNC:	14	14q31.1	centrosomal	protein-codir	CEP128	centrosomal	O	centrosomal	20210302
BEST1	ENSG00000167995	7439	410.74	-1.37	-3.96	7.42111E-05	0.003757133	962.379903	352.816233	3.10946799	1.65808391	43.7706029	12.0480803	16.7777627	10.8314758	4574.97764	356.276878	5.48274818	6.40391167	96.1066348	34.56586421	26.9933706	67.58883315	ARB|BEST|BI	MIM:607854	11	11q12.3	bestrophin 1	protein-codir	BEST1	bestrophin 1	O	bestrophin-1	20210418
SNORD110	ENSG00000221116	692213	177.49	-2.95	-3.96	7.59708E-05	0.003836022	363.738668	682.764423	8.10756493	60.9368404	7.98878914	5.10120574	0	0	318.608302	494.78929	466.84762	294.968954	17.6838369	38.10421333	26.3125295	53.82498007	HBII-55	HGNC:HGNC:	20	20p13	small nucleol	snoRNA	SNORD110	small nucleol	O	-	20210302
	ENSG00000279833		28.28	-2.87	-3.95	7.78263E-05	0.003908975	4.63730118	28.0311295	0	3.26876255	0	0.85006007	6.5137344	11.368213	89.6272635	156.49373	2.46766469	2.85133589	46.1778138	47.89242599	26.4798039	25.84492833											
RGL4	ENSG00000159496	266747	42.21	-2.22	-3.95	7.86858E-05	0.003941747	36.8411454	18.3897286	19.8019956	0	15.8583245	7.4498827	1.05858011	11.6035994	354.667644	29.5470313	35.7591295	23.3555197	28.0091585	24.68325656	23.2420189	45.03189715	Rgr	MIM:612214	22	22q11.23	ral guanine n	protein-codir	RGL4	ral guanine n	O	ral-GDS-relat	20210316
GPLD1	ENSG00000112293	2822	12.27	-2.52	-3.95	7.89006E-05	0.003942131	3.57289525	7.1842715	2.68346788	0.6916243	0	8.67789585	5.58693826	1.29987326	22.9336281	45.8510251	11.3079637	20.4712332	16.6016904	26.28903995	20.0869095	3.060436421	GPIPLD|GPIP	MIM:602515	6	6p22.3	glycosylphos	protein-codir	GPLD1	glycosylphos	O	phosphatidyl	20210302
MMP9	ENSG00000100985	4318	19.43	-5.02	-3.94	7.9961E-05	0.003953605	1.19574511	0	11.3856366	0	0	0	0	0	30.0869273	5.4771563	0.49345302	28.8667551	220.752766	5.889992611	4.11421643	2.668646627	CLG4B|GELB	MIM:120361	20	20q13.12	matrix metall	protein-codir	MMP9	matrix metall	O	matrix metall	20210418
YTHDC2	ENSG00000047188	64848	820.90	0.71	3.95	7.96065E-05	0.003953605	788.689881	742.140267	2271.54536	1007.42719	1155.66611	807.833934	821.583113	682.438274	337.794517	645.199643	826.808411	623.146699	699.7963	720.4079054	629.666145	374.197001	CAHL|hYTHD	MIM:616530	5	5q22.2	YTH domain c	protein-codir	YTHDC2	YTH domain c	O	3'-5' RNA heli	20210418
HSBP1L1	ENSG00000226742	440498	48.47	-1.46	-3.94	8.04493E-05	0.003967442	57.9175204	43.0545521	28.5810201	28.8700734	4.62909528	15.784577	12.2302732	9.40201902	241.018505	97.3641983	30.6545639	38.9107869	17.1623228	70.94264336	46.0970922	32.95654864	-	HGNC:HGNC:	18	18q23	heat shock fa	protein-codir	HSBP1L1	heat shock fa	O	heat shock fa	20210302

CKAP4	ENSG00000136026	10970	79.48	-1.36	-3.94	8.11827E-05	0.003972671	102.257728	61.9873202	32.6549646	18.903146	28.6139435	71.416582	21.0616898	10.4816483	376.89061	135.965302	31.0217137	85.6325611	41.6265014	116.3520183	83.3047735	53.44458158	CLIMP-63|CL	MIM:618595	12	12q23.3	cytoskeleton	protein-codir	CKAP4	cytoskeleton	O	cytoskeleton-	20210322
KLRG1	ENSG00000139187	10219	631.04	0.64	3.94	8.0882E-05	0.003972671	565.219721	412.912223	983.308938	500.786332	1161.04936	394.48093	700.341867	1541.84077	282.213549	316.980361	545.056287	360.792899	623.522249	434.5828089	394.390051	879.1541585	2F1|CLEC15A	MIM:604874	12	12p13.31	killer cell lect	protein-codir	KLRG1	killer cell lect	O	killer cell lect	20210418
NABP1	ENSG00000173559	64859	949.05	0.85	3.94	8.13901E-05	0.003972671	954.331708	743.870714	1790.92269	934.450944	1668.46283	944.788342	986.407957	1762.46471	754.979727	401.270912	515.734927	398.38254	719.687516	1016.11365	766.804028	826.2046916	OBFC2A|SOS	MIM:612103	2	2q32.3	nucleic acid b	protein-codir	NABP1	nucleic acid b	O	SOSS comple	20210302
FASLG	ENSG00000117560	356	256.68	1.13	3.94	8.1124E-05	0.003972671	16.1166855	144.001034	397.121943	335.564549	603.124114	310.209809	447.048757	567.369683	20.5218797	110.605027	149.972426	127.890394	358.739478	59.80292724	116.291609	342.4426882	ALPS1B|APT1	MIM:134638	1	1q24.3	Fas ligand	protein-codir	FASLG	Fas ligand	O	tumor necros	20210410
ABR	ENSG00000159842	29	662.77	1.05	3.94	8.31857E-05	0.00404993	781.211716	432.269856	2372.38558	493.705433	945.548426	759.192539	896.323578	655.46667	210.841237	539.877454	476.144648	279.865355	674.527897	330.6917504	378.642642	377.5818847	MDB	MIM:600365	17	17p13.3	ABR activator	protein-codir	ABR	ABR activator	O	active breakp	20210417
SLC22A23	ENSG00000137266	63027	44.60	-3.58	-3.93	8.38511E-05	0.004061552	75.6388664	0.54716922	19.1109687	16.3433418	0	0	0.34022627	11.4086113	303.705762	48.4668717	23.6040484	32.1853123	28.4387183	60.90112365	84.3081393	8.680533573	C6orf85	MIM:611697	6	6p25.2	solute carrier	protein-codir	SLC22A23	solute carrier	O	solute carrier	20210327
ATP6V1C2	ENSG00000143882	245973	47.78	-3.25	-3.93	8.43855E-05	0.004062523	19.7413481	9.75203957	0	0	0	0	5.89873538	0.60162937	546.556192	136.377617	0	0	9.67895349	8.233447008	11.1549139	16.49551132	ATP6C2|VMA	MIM:618070	2	2p25.1	ATPase H+ tra	protein-codir	ATP6V1C2	ATPase H+ tra	O	V-type proto	20210417
TNFRSF13C	ENSG00000159958	115650	62.65	-2.36	-3.93	8.51397E-05	0.004062523	139.213965	94.7420035	0	12.783403	15.8752601	9.95587659	22.5651736	2.6184042	76.7145787	110.626201	122.499842	98.8367389	99.8837586	78.5711303	83.1003217	34.40684631	BAFF-R|BAFF	MIM:606269	22	22q13.2	TNF receptor	protein-codir	TNFRSF13C	TNF receptor	O	tumor necros	20210406
PFKFB3	ENSG00000170525	5209	349.14	-0.83	-3.93	8.53651E-05	0.004062523	243.175811	377.449652	149.264323	226.939719	275.419628	177.654121	230.7742	250.998301	918.949877	483.518396	313.838082	341.126978	648.832762	237.1399986	366.501146	344.7325076	IPFK2|PFK2|i	MIM:605319	10	10p15.1	6-phosphofru	protein-codir	PFKFB3	6-phosphofru	O	6-phosphofru	20210418
EIF3E	ENSG00000104408	3646	2461.55	-0.62	-3.93	8.48997E-05	0.004062523	3484.54644	2171.0235	2308.9383	2544.58149	926.046646	1829.35371	1062.08974	1321.16098	5242.50507	2360.15238	2574.3305	4743.37846	1466.52151	2295.212882	2498.4319	2556.48606	EIF3-P48|EIF	MIM:602210	8	8q23.1	eukaryotic tra	protein-codir	EIF3E	eukaryotic tra	O	eukaryotic tra	20210404
PPIB	ENSG00000166794	5479	546.25	-0.61	-3.93	8.49288E-05	0.004062523	422.080487	385.798702	521.563408	501.826272	218.678883	471.299454	417.658174	548.410127	795.29325	541.826295	546.255353	769.036522	532.707147	620.3328488	598.209763	849.1030538	B|CYP-S1|CY	MIM:123841	15	15q22.31	peptidylproly	protein-codir	PPIB	peptidylproly	O	peptidyl-prol	20210404
PTGDR	ENSG00000168229	5729	288.70	1.10	3.93	8.5262E-05	0.004062523	86.6100951	275.422526	260.472835	203.673958	475.265161	500.174869	892.285689	483.662465	75.1951284	169.486049	232.864812	50.4918241	325.150443	112.2758182	260.251478	215.9951415	AS1|ASRT1|D	MIM:604687	14	14q22.1	prostaglandin	protein-codir	PTGDR	prostaglandin	O	prostaglandin	20210316
LOC12490228	ENSG00000227218	124902280	13.44	-3.22	-3.93	8.56887E-05	0.004067755	13.4290086	5.19051335	0	0	0	0	3.54157922	0.62930172	46.3789462	92.4303634	1.67336391	1.02717649	8.18541417	16.87078402	17.4825615	8.195845482											
	ENSG00000234389		21.44	1.89	3.93	8.64765E-05	0.004094941	32.7634523	16.8766197	89.1928824	20.1432302	24.1444531	29.9786784	34.7373793	21.2942668	4.47864329	5.12636007	9.27915364	3.93096339	20.4110096	7.108287677	18.4649566	5.188956395											
	ENSG00000260465		12.27	-4.65	-3.92	8.68019E-05	0.004100151	0	2.90027235	0	0	6.55910283	0	0	0	52.2034632	93.9620115	3.79869438	0.69459847	8.09060319	14.03751334	4.92710604	9.17279962											
SLC46A1	ENSG00000076351	113235	30.35	-2.70	-3.92	8.7305E-05	0.004113706	6.3082282	91.4021957	0	0.94343127	0	11.0785954	0.8055684	2.64893215	162.246496	111.709326	2.94254955	4.65677833	15.9983338	45.27046539	23.6497968	6.005194164	G21|HCP1|P	MIM:611672	17	17q11.2	solute carrier	protein-codir	SLC46A1	solute carrier	O	proton-coupl	20210410
VPS51	ENSG00000149823	738	497.88	-0.75	-3.92	8.79714E-05	0.004134871	608.574896	497.470348	136.135622	329.57099	313.188072	443.619508	410.66364	339.748961	655.905944	790.528937	466.633412	605.812066	577.18973	573.1999307	592.934991	624.94335	ANG2|ANG3	MIM:615738	11	11q13.1	VPS51 subun	protein-codir	VPS51	VPS51 subun	O	vacuolar prot	20210302
AIF1	ENSG00000204472	199	76.50	-3.19	-3.92	8.9476E-05	0.004195236	90.9031147	16.9729526	137.184347	1.6531748	2.29483478	1.94180932	0	0	759.201101	10.4658151	52.1841848	65.8608188	27.2999888	10.79214694	17.2928894	30.02580618	AIF-1|IBA1|II	MIM:601833	6	6p21.33	allograft infla	protein-codir	AIF1	allograft infla	O	allograft infla	20210302
CD101	ENSG00000134256	9398	97.75	-2.16	-3.91	9.07173E-05	0.004232988	34.9112819	118.38568	0	2.97289091	361.900146	5.56454762	29.8581469	8.23006557	74.7503776	107.492471	31.4804675	77.3563092	478.827117	49.26584114	81.8137418	101.14989	EWI-101|IGS	MIM:604516	1	1p13.1	CD101 molec	protein-codir	CD101	CD101 molec	O	immunoglobu	20210302
SNORA22	ENSG00000206634	677807	40.84	-1.91	-3.91	9.07259E-05	0.004232988	52.143542	34.0815496	8.68121807	9.85384672	0	17.203329	14.9545015	25.8345839	33.3931388	86.5803468	41.2011228	98.9537765	16.2250127	54.10252244	74.9060887	85.27268916	ACA22|SNOR	HGNC:HGNC:	7	7q11.21	small nucleol	snoRNA	SNORA22	small nucleol	O	ACA22 snoRN	20210302
PPFIA3	ENSG00000177380	8541	30.65	1.95	3.91	9.24004E-05	0.004300332	10.0907594	40.1351636	16.204586	33.2488388	95.3395205	51.2979261	76.5001986	71.7264756	12.9278796	21.0941913	8.20505065	7.47695989	22.4266698	9.083641091	6.63613262	8.064359435	LPNA3	MIM:603144	19	19q13.33	PTPRF interac	protein-codir	PPFIA3	PTPRF interac	O	liprin-alpha-3	20210302
PDLIM5	ENSG00000163110	10611	477.26	0.71	3.91	9.30233E-05	0.004308493	688.702225	582.566017	1083.77311	576.168993	476.082148	580.811854	373.844427	438.171462	375.668941	369.804574	367.577124	417.103837	366.799824	293.4260052	235.93991	409.7078509	ENH|ENH1|L	MIM:605904	4	4q22.3	PDZ and LIM	protein-codir	PDLIM5	PDZ and LIM	O	PDZ and LIM	20210329
ICOSLG	ENSG00000160223	23308	14.15	-3.24	-3.91	9.3358E-05	0.004313501	4.28500566	5.51290958	0	0	0	10.6915041	5.26695611	0.9513435	40.5775365	11.4635209	10.2556377	8.74652774	36.6004588	14.77708206	24.1276938	53.16011199	B7-H2|B7H2	MIM:605717	21	21q22.3	inducible T ce	protein-codir	ICOSLG	inducible T ce	O	ICOS ligand|E	20210302
VSIG1	ENSG00000101842	340547	122.96	-3.05	-3.91	9.40382E-05	0.004313522	581.991593	188.457737	0	4.0316555	0	129.456587	6.76374848	9.7850659	119.86494	199.007362	92.0069893	133.475472	89.9304991	167.0250822	171.521436	74.05960825	1700062D20I	MIM:300620	X	Xq22.3	V-set and imr	protein-codir	VSIG1	V-set and imr	O	V-set and imr	20210302
GCNT4	ENSG00000176928	51301	177.54	-2.21	-3.91	9.40137E-05	0.004313522	386.528861	210.209216	0	24.5916711	10.6145676	200.003281	46.7535914	23.2213587	209.299042	184.699972	179.339469	227.602989	67.9326146	509.5070263	397.303692	163.0173002	C2GNT3|LIN(	MIM:616782	5	5q13.3	glucosaminyl	protein-codir	GCNT4	glucosaminyl	O	beta-1,3-gala	20210302
TIAL1	ENSG00000151923	7073	1173.82	0.78	3.91	9.40321E-05	0.004313522	1679.30285	907.798826	3030.89599	1341.33518	1516.11315	1286.60639	1168.53358	1142.61768	543.994323	926.715188	1038.01501	878.349825	928.819861	871.4413247	856.025366	664.6248116	TCBP|TIAR	MIM:603413	10	10q26.11	TIA1 cytotoxi	protein-codir	TIAL1	TIA1 cytotoxi	O	nucleolysin T	20210404
THOC1	ENSG00000079134	9984	379.62	0.60	3.90	9.57909E-05	0.004383356	290.068195	388.941088	971.349582	586.745986	337.971111	472.664354	343.943043	297.755041	156.906778	258.83603	478.570206	426.59894	310.745745	293.2519449	292.757132	166.7991721	HPR1|P84|P8	MIM:606930	18	18p11.32	THO complex	protein-codir	THOC1	THO complex	O	THO complex	20210406
FCRL6	ENSG00000181036	343413	607.90	0.95	3.90	9.72392E-05	0.004438959	265.462581	464.47354	954.603264	921.707128	1104.95901	818.58759	684.000404	945.821786	114.417492	471.365513	431.664179	377.358382	821.451223	243.013419	205.891519	901.6099907	FcRH6	MIM:613562	1	1q23.2	Fc receptor li	protein-codir	FCRL6	Fc receptor li	O	Fc receptor-li	20210302
	ENSG00000228863		14.33	-3.42	-3.90	9.81696E-05	0.00447071	0	4.41321808	0	0	7.8382457	0.72188903	0.59193645	6.298648	66.5741043	80.538951	5.85443809	2.41432743	13.3282867	18.88364535	6.85215091	15.04604774											
CNN2	ENSG00000064666	1265	1011.69	-0.74	-3.89	0.000100142	0.004549654	1209.71736	935.472367	375.387768	367.275935	450.491397	950.573609	649.712073	1401.41079	1459.69344	1188.74691	694.112173	1198.44335	891.355388	1154.143598	1391.1602	1869.274347	-	MIM:602373	19	19p13.3	calponin 2	protein-codir	CNN2	calponin 2	O	calponin-2|ca	20210302
	ENSG00000279088		106.53	1.04	3.88	0.000104861	0.004741406	123.171484	98.4255184	359.328355	133.061232	74.2472606	95.3219983	180.888453	100.305517	47.121684	97.3499996	95.3792772	92.5646425	49.9782539	37.15281236	80.6934773	39.44030388											
CHAC1	ENSG00000128965	79094	12.31	-3.21	-3.87	0.000108664	0.004901728	16.7536164	0.84210389	0	0	0	0	4.87005861	2.52291085	92.7141589	18.6086103	3.89076113	3.39625889	6.27776543	19.25980424	20.5204735	7.30082541	-	MIM:614587	15	15q15.1	ChaC glutathi	protein-codir	CHAC1	ChaC glutathi	O	glutathione-s	20210302
EML6	ENSG00000214595	400954	72.05	1.83	3.87	0.000109654	0.004934712	88.7291207	251.37189	53.6412218	82.8399058	250.099884	21.1808607	32.3004887	111.777764	6.41365105	105.967923	12.360704	26.5917084	45.1359479	26.7772126	28.285439	9.250996347	-	HGNC:HGNC:	2	2p16.1	EMAP like 6	protein-codir	EML6	EMAP like 6	O	echinoderm r	20210410
SCOC-AS1	ENSG00000196951	100129858	11.90	-3.30	-3.87	0.00011037	0.004946861	1.00505253	1.60013945	0	0	0	1.45782645	6.85119402	9.30621723	38.6118418	2.96737398	26.1169793	36.0893658	14.2562036	8.469397724	16.8973431	26.74282078	-	HGNC:HGNC:	4	4q31.1	SCOC antisen	ncRNA	SCOC-AS1	SCOC antisen	O	-	20210302
FAAH2	ENSG00000165591	158584	32.15	-3.14	-3.86	0.000111477	0.004981415	115.473603	20.4387804	10.2330187	1.3238907	0	12.4433777	0.50471286	1.38321872	25.1490359	64.6454616	35.4716527	31.7938472	27.7767165	44.89296401	75.3703261	47.53933938	AMDD	MIM:300654	X	Xp11.21	fatty acid am	protein-codir	FAAH2	fatty acid am	O	fatty-acid am	20210302
SNORD21	ENSG00000206680	6083	713.81	-1.62	-3.86	0.000111888	0.00498804	2078.69342	3085.90717	28.5761457	62.7587823	24.0919063	15.9886119	8.85371882	24.6918517	2367.70042	2444.575	433.59859	645.028474	40.5866273	44.64057097	41.3189024	73.99237231	RNU21|U21	MIM:603635	1	1p22.1	small nucleol	snoRNA	SNORD21	small nucleol	O	RNA, U21 sm	20210302
LILRB1	ENSG00000104972	10859	315.45	1.29	3.86	0.000112212	0.004990833	205.500627	264.058309	338.45886	148.865255	477.18431	625.406198	889.225723	642.689206	283.572587	132.731129	143.060088	65.4443157	407.101425	155.6056598	154.999037	113.2658777	CD85J|ILT-2|	MIM:604811	19	19q13.42	leukocyte imr	protein-codir	LILRB1	leukocyte imr	O	leukocyte imr	20210302
GLIS3	ENSG00000107249	169792	68.34	-2.81	-3.86	0.000113994	0.00505823	47.7869581	7.51827565	0	0	0	33.6129954	24.5883956	3.15521398	760.224183	18.3138975	13.4303286	48.1682935	26.4595596	20.58642291	74.9415108	14.64034138	NDH|ZNF515	MIM:610192	9	9p24.2	GLIS family zi	protein-codir	GLIS3	GLIS family zi	O	zinc finger pr	20210418
A2M-AS1	ENSG00000245105	144571	425.15	1.00	3.85	0.000116527	0.005158627	389.532455	158.977006	227.086338	554.815058	1671.97011	471.228223	500.257984	705.398458	143.311814	142.856377	327.614688	184.238337	608.595194	175.410358	278.92135	262.237699	-	HGNC:HGNC:	12	12p13.31	A2M antisens	ncRNA	A2M-AS1	A2M antisens	O	A2M antisens	20210302
SNORD116-1	ENSG00000207174	100033427	38.92	-2.32	-3.85	0.000117587	0.005193479	53.3511349	50.9524576	0	2.46595132	2.4807632	21.0560828	6.01457744	25.921506	64.4661389	58.7641965	37.2622742	44.317775	36.7142989	58.6703	90.9429515	69.26034267	HBII-85-15	HGNC:HGNC:	15	15q11.2	small nucleol	snoRNA	SNORD116-1	small nucleol	O	-	20210302
SLF1	ENSG00000133302	84250	378.65	0.82	3.85	0.000119407	0.005261652	461.014362	611.201032	461.120333	236.597314	910.078489	283.865445	289.707118	696.04931	211.224143	327.882027	242.667111	232.871305	276.967294	241.205357	152.594944	423.3870911	ANKRD32|BR	MIM:618467	5	5q15	SMC5-SMC6	protein-codir	SLF1	SMC5-SMC6	O	SMC5-SMC6	20210302
XAF1	ENSG00000132530	54739	431.83	1.05	3.85	0.000120344	0.005290705	618.569987	495.727375	1683.2078	251.565266	307.274982	567.396643	377.518212	539.734159	408.199893	154.979301	260.674351	153.516884	313.700744	359.4325049	188.2078	229.6032058	BIRC4BP|HSX	MIM:606717	17	17p13.1	XIAP associat	protein-codir	XAF1	XIAP associat	O	XIAP-associat	20210302
	ENSG00000238241		28.65	-4.03	-3.84	0.000121568	0.005332178	95.5461964	22.0985133	0	0	0	3.35184347	10.4106513	0	17.2693817	14.9954504	13.8097657	57.5165256	65.204107	79.91424967	43.8090076	34.47714905											
PLEKHA7	ENSG00000166689	144100	25.02	-2.94	-3.84	0.000122212	0.005335853	18.8809451	3.25338755	17.8650895	0	0	3.99052998	1.38818504	1.48765678	225.042347	5.18390808	16.8965063	5.81156288	10.1647699	14.34572583	63.2558792	12.79780382	-	MIM:612686	11	11p15.2-p15.	pleckstrin ho	protein-codir	PLEKHA7	pleckstrin ho	O	pleckstrin ho	20210302
INF2	ENSG00000203485	64423	84.92	-2.91	-3.84	0.000122178	0.005335853	192.705988	20.9273969	88.2724	1.17194749	0	61.5219264	3.50719976	4.13408887	208.572328	24.3948196	83.2991196	191.825058	101.41674	176.6380653	119.485426	80.81914919	C14orf151|C	MIM:610982	14	14q32.33	inverted form	protein-codir	INF2	inverted form	O	inverted form	20210302
INSYN2B	ENSG00000204767	100131897	17.05	-4.32	-3.84	0.000122982	0.005352374	0	15.6244332	2.41933248	1.22855131	0	0	0	0	137.311784	68.472057	1.04926347	0.50374295	7.32056136	21.3991008	8.90134302	8.62109518	C5orf57|FAM	HGNC:HGNC:	5	5q35.1	inhibitory syr	protein-codir	INSYN2B	inhibitory syr	O	protein INSYN	20210302
EEF2	ENSG00000167658	1938	7722.27	-0.81	-3.84	0.000123153	0.005352374	5488.0427	4657.13026	10315.2089	6991.30855	3793.00722	4532.03498	4170.82883	5476.36227	13047.9174	8902.74694	6167.59948	13614.5727	6423.8252	10011.48441	8208.17899	11756.05138	EEF-2|EF-2|E	MIM:130610	19	19p13.3	eukaryotic tra	protein-codir	EEF2	eukaryotic tra	O	elongation fa	20210418
SRGAP2B	ENSG00000196369	647135	185.45	0.96	3.84	0.000125381	0.005436799	158.817481	330.961197	170.165151	90.1089478	609.153525	202.094414	366.829881	116.069602	80.2698319	173.640263	77.7368446	81.0782314	170.70579	82.56829193	134.700742	122.3587194	SRGAP2L|SR(	MIM:614703	1	1q21.1	SLIT-ROBO RI	protein-codir	SRGAP2B	SLIT-ROBO RI	O	SLIT-ROBO RI	20210302
DNPH1	ENSG00000112667	10591	24.43	-1.92	-3.83	0.000126593	0.005476867	1.51780032	11.6891985	9.63052679	2.59082101	7.58803846	25.2530839	31.8031153	8.12572528	19.9807759	30.1321953	20.9571324	31.3512624	43.8186073	43.08430014	60.1505683	43.25420781	C6orf108|RC	MIM:618762	6	6p21.1	2'-deoxynucl	protein-codir	DNPH1	2'-deoxynucl	O	2'-deoxynucl	20210417
RNF43	ENSG00000108375	54894	19.01	-4.09	-3.83	0.000129054	0.005558082	0.97477159	19.7461452	0	2.62057011	0	26.3592486	0	0	129.954407	44.1153853	10.4177535	10.7308505	12.8299762	9.6321195	22.1664295	14.69068838	RNF124|SSP(	MIM:612482	17	17q22	ring finger pr	protein-codir	RNF43	ring finger pr	O	E3 ubiquitin-|	20210418
KCNQ1	ENSG00000053918	3784	46.39	-2.89	-3.83	0.000129685	0.005572669	96.1895376	33.964404	0	0	27.0549046	11.5243775	11.6565055	0	163.594025	66.3831748	21.9443576	58.3216335	53.2469123	38.53476344	112.393597	47.47048495	ATFB1|ATFB3	MIM:607542	11	11p15.5-p15.	potassium vo	protein-codir	KCNQ1	potassium vo	O	potassium vo	20210418
RAB15	ENSG00000139998	376267	24.15	-2.75	-3.82	0.000132742	0.00569116	15.9483417	11.6940911	0	0	0	24.9083747	2.02044757	3.57134909	162.706792	91.7207659	7.15140527	9.60891344	9.28809211	18.52940305	16.853707	12.33910972	-	HGNC:HGNC:	14	14q23.3	RAB15, mem	protein-codir	RAB15	RAB15, mem	O	ras-related pr	20210302
MFGE8	ENSG00000140545	4240	92.89	-1.46	-3.82	0.000133074	0.005692561	127.762982	24.6852093	15.0834221	20.2973793	41.740891	220.771253	15.0476272	15.2403133	116.394519	39.8950016	96.712161	76.9723256	123.715929	383.3228605	130.811389	37.75348504	BA46|EDIL1|	MIM:602281	15	15q26.1	milk fat globu	protein-codir	MFGE8	milk fat globu	O	lactadherin|(	20210329
RPS8	ENSG00000142937	6202	4349.94	-0.66	-3.82	0.000135529	0.005784618	3814.92871	5762.58823	3566.76952	3343.53111	2256.04135	3165.81684	2425.0477	2609.78567	8729.99674	5270.20096	4082.12922	7049.25117	3336.31883	4317.635229	4960.69786	4908.291041	S8	MIM:600357	1	1p34.1	ribosomal pro	protein-codir	RPS8	ribosomal pro	O	40S ribosoma	20210404
SNORA54	ENSG00000207008	677833	72.09	-1.92	-3.81	0.000136233	0.005801635	118.821652	44.6689386	2.81319933	2.50577745	17.3627121	60.1222052	21.8663593	55.1905604	63.0810974	65.9331113	52.9703109	43.0845953	77.9531825	165.2484491	100.899481	260.857802	ACA54	HGNC:HGNC:	11	11p15.4	small nucleol	snoRNA	SNORA54	small nucleol	O	ACA54 snoRN	20210302
SNORA24	ENSG00000275994	677809	216.27	-2.17	-3.81	0.000137349	0.005836125	1189.57834	246.221288	5.72722755	21.0471427	14.323293	34.0113381	17.6134057	43.36859	367.306527	307.71217	381.288367	351.703777	77.4794493	108.6934967	118.338176	175.9156955	ACA24|SNOR	HGNC:HGNC:	4	4q26	small nucleol	snoRNA	SNORA24	small nucleol	O	ACA24 snoRN	20210302
RPL12	ENSG00000197958	6136	3600.85	-0.85	-3.81	0.000138022	0.005838637	3262.8009	2649.89522	2847.55337	3120.05307	1098.80388	2503.8619	1405.99871	1615.87807	14410.6979	2428.9746	4072.25251	7156.61228	1824.94388	3269.177749	2826.23674	3119.926537	L12	MIM:180475	9	9q33.3	ribosomal pro	protein-codir	RPL12	ribosomal pro	O	60S ribosoma	20210404
TAGLN2	ENSG00000158710	8407	503.52	-0.67	-3.81	0.000141356	0.005966434	479.821443	551.219509	145.94249	377.669718	278.857557	404.867667	491.805062	428.971748	549.266569	669.628995	392.224077	768.219499	436.495009	652.6858493	583.643747	844.9702052	HA1756	MIM:604634	1	1q23.2	transgelin 2	protein-codir	TAGLN2	transgelin 2	O	transgelin-2|	20210404
SNORD46	ENSG00000200913	94161	358.37	-2.34	-3.80	0.000144177	0.006072011	1265.27332	535.95944	22.7728291	59.0412038	7.22328766	32.8443299	11.1643877	61.5777911	322.549575	310.771344	1155.49631	1393.64225	66.4172456	94.94288119	115.162503	279.055586	RNU40|RNU4	HGNC:HGNC:	1	1p34.1	small nucleol	snoRNA	SNORD46	small nucleol	O	RNA, U40 sm	20210302
TRAPPC5	ENSG00000181029	126003	104.24	-0.78	-3.80	0.000145708	0.006096025	128.497745	123.494885	35.3559591	80.8938359	52.1933975	81.4206271	40.7128558	80.2419149	216.238513	176.319226	90.2976861	107.604819	95.3075384	121.552115	90.6336921	147.0941666	TRS31	HGNC:HGNC:	19	19p13.2	trafficking pro	protein-codir	TRAPPC5	trafficking pro	O	trafficking pro	20210302
ARMCX2	ENSG00000184867	9823	55.21	-3.01	-3.80	0.000146296	0.00610722	11.4114143	4.94448048	0	0	0	5.72460631	91.1191839	3.85770601	599.83844	22.2205448	20.5277743	23.1809157	11.7626607	29.31519972	52.9964004	6.504575801	ALEX2|GASP	MIM:300363	X	Xq22.1	armadillo rep	protein-codir	ARMCX2	armadillo rep	O	armadillo rep	20210418
	ENSG00000273275		20.56	-3.47	-3.79	0.000148452	0.006183674	1.34892484	11.1594369	0	0	4.01623033	0	3.14104594	0	93.3988088	180.477807	0.88710547	2.43736087	5.61701397	10.5374876	8.33914678	7.54486237											
CLDN7	ENSG00000181885	1366	10.78	-3.74	-3.79	0.000150018	0.006235251	2.07635024	1.34379428	0	0	0	2.48214741	0	0	121.92224	11.474089	2.12108464	0.80901557	8.15791821	8.148305208	11.4048612	2.584078818	CEPTRL2|CLD	MIM:609131	17	17p13.1	claudin 7	protein-codir	CLDN7	claudin 7	O	claudin-7|clo	20210302
PREB	ENSG00000138073	10113	183.21	-0.84	-3.79	0.00015076	0.006252442	159.117845	89.7048791	75.6582461	85.8559652	111.783919	107.878223	237.237124	224.794623	266.941062	199.442655	174.498871	155.978745	273.544318	265.9057997	226.177313	276.7805997	SEC12	MIM:606395	2	2p23.3	prolactin regu	protein-codir	PREB	prolactin regu	O	prolactin regu	20210302
RN7SL4P	ENSG00000263740	6030	292.71	-2.18	-3.79	0.000151308	0.006261033	76.4549422	86.9620574	200.875186	50.8030886	2.10297598	28.7575625	33.3667461	56.695127	1834.5367	1706.6074	51.4600553	140.077834	92.804864	82.95464923	58.3483156	180.5496247	7L28|RN7SL4	HGNC:HGNC:	3	3p25.1	RNA, 7SL, cyt	pseudo	RN7SL4P	RNA, 7SL, cyt	O	RNA, 7SL, cyt	20210302
FCGR2A	ENSG00000143226	2212	480.50	2.35	3.79	0.000151625	0.006261033	284.251493	152.31853	36.8874456	11.5291341	174.437675	44.8540841	25.9290304	33.2663002	6808.91782	20.7598211	2.77988112	1.56250194	78.1629996	2.025149153	5.59869001	4.655929674	CD32|CD32A	MIM:146790	1	1q23.3	Fc fragment c	protein-codir	FCGR2A	Fc fragment c	O	low affinity ir	20210418
	ENSG00000272211		30.63	-3.51	-3.79	0.000152733	0.006293136	3.65284871	6.02091869	0	1.62942728	0	0.8034618	0	10.0437768	181.921104	247.275123	1.49979214	1.42448683	10.8208485	8.609445275	9.99251994	6.374334219											
SLC5A2	ENSG00000140675	6524	13.41	-4.01	-3.78	0.000153978	0.006330719	6.80743138	1.40747516	0	0	0	0	0	2.21587784	64.3227752	54.6436794	5.360468	0.32995146	6.92892755	44.77311694	25.5304453	2.315791002	SGLT2	MIM:182381	16	16p11.2	solute carrier	protein-codir	SLC5A2	solute carrier	O	sodium/gluco	20210410
SPAG9	ENSG00000008294	9043	884.61	0.69	3.78	0.000154539	0.00634008	1229.11615	591.079071	1849.70665	1193.97567	968.46833	1132.44567	830.382667	1065.72958	579.364453	609.904474	788.619989	517.979706	772.910689	756.1118256	684.381633	583.5720904	CT89|HLC-6|	MIM:605430	17	17q21.33	sperm associ	protein-codir	SPAG9	sperm associ	O	C-Jun-amino-	20210302
	ENSG00000265401		48.22	-2.63	-3.78	0.000155947	0.006384118	23.5644469	7.83859793	0	4.65302436	4.47170599	0	11.1836809	13.8330226	242.853325	347.759973	2.37507803	3.56629989	16.0186961	22.82903129	34.4515469	36.04594986											
NOSIP	ENSG00000142546	51070	146.67	-0.76	-3.78	0.000157178	0.006420682	132.957708	118.288987	60.6098692	68.168247	65.9831118	169.189802	167.470241	105.396538	165.240879	182.650314	111.281555	142.455982	200.577402	244.5471695	250.536775	161.4344586	CGI-25	MIM:616759	19	19q13.33	nitric oxide sy	protein-codir	NOSIP	nitric oxide sy	O	nitric oxide sy	20210302
PRPF4B	ENSG00000112739	8899	2181.65	0.60	3.78	0.000158853	0.006475232	3258.62245	2423.09113	2116.72119	2835.47493	2253.73822	2366.01551	3118.08983	2619.79372	1497.01192	1875.12056	2166.02014	1495.62714	1663.32228	1918.715786	2036.72683	1262.232339	PR4H|PRP4|I	MIM:602338	6	6p25.2	pre-mRNA pr	protein-codir	PRPF4B	pre-mRNA pr	O	serine/threor	20210302
CDK5RAP3	ENSG00000108465	80279	446.06	0.72	3.77	0.00016233	0.006602792	611.875231	302.216266	694.814628	267.060607	883.380008	653.88505	509.497045	504.854478	229.659912	229.97435	398.079249	219.133181	504.547015	560.388549	374.596243	193.0329225	C53|HSF-27|	MIM:608202	17	17q21.32	CDK5 regulat	protein-codir	CDK5RAP3	CDK5 regulat	O	CDK5 regulat	20210329
	ENSG00000218226		21.07	-3.27	-3.76	0.000167704	0.006792356	0.93386284	0	0	0	2.38149287	2.39935233	8.46811139	18.4784339	68.5293173	119.391745	3.57533127	0.69085958	33.1976889	19.44125228	29.7048824	29.85469717											
JCHAIN	ENSG00000132465	3512	10.18	-4.66	-3.76	0.000170229	0.006865431	3.13096129	0	19.0221032	0	0	0	0	0	52.2035955	1.90111463	4.57972101	8.30895485	49.866746	10.82050574	4.1549074	8.8165019	IGCJ|IGJ|JCH	MIM:147790	4	4q13.3	joining chain	protein-codir	JCHAIN	joining chain	O	immunoglobu	20210417
	ENSG00000280143		34.78	-2.96	-3.76	0.000170858	0.006876237	0	5.08977032	0	1.61582452	14.3058267	12.0489467	4.59617464	24.3766166	139.072453	186.717329	12.6555599	5.05062601	32.6957353	59.98816943	35.3953023	22.84767416											
LUC7L3	ENSG00000108848	51747	2881.80	0.61	3.75	0.000174558	0.007010323	2056.26026	2071.9298	4045.26173	3032.28187	4485.62007	4691.43539	3841.59034	3483.73735	1723.28562	1889.41744	2053.07202	1507.43343	3000.2902	3347.200429	3387.24743	1492.782306	CRA|CREAP-1	MIM:609434	17	17q21.33	LUC7 like 3 pr	protein-codir	LUC7L3	LUC7 like 3 pr	O	luc7-like prot	20210404
TMEM272	ENSG00000281106	283521	44.15	-3.30	-3.74	0.000180454	0.007221874	94.2866019	35.1837249	0	8.35388569	0	48.0403518	4.69016461	0	38.2898569	21.9910218	43.6575089	68.2734593	44.4481462	149.1541304	97.9923698	52.01660144	LINC00282|N	HGNC:HGNC:	13	13q14.3	transmembra	protein-codir	TMEM272	transmembra	O	transmembra	20210302
SNX9	ENSG00000130340	51429	378.41	-1.34	-3.74	0.000180585	0.007221874	515.600184	482.324535	510.429559	105.38889	35.1982095	262.572656	119.198876	88.6862262	845.429083	588.060928	443.818895	458.650365	367.58181	397.1866507	470.535029	363.9455755	SDP1|SH3PX1	MIM:605952	6	6q25.3	sorting nexin	protein-codir	SNX9	sorting nexin	O	sorting nexin-	20210302
RAI2	ENSG00000131831	10742	7.46	-3.37	-3.74	0.000182245	0.007272985	4.99995161	6.2305033	0	1.80753148	0	0.8655686	0	0	35.8620209	24.8372698	8.58750798	11.5594404	3.52755278	10.96345768	2.26438602	7.893363503	-	MIM:300217	X	Xp22.13	retinoic acid i	protein-codir	RAI2	retinoic acid i	O	retinoic acid-	20210302
CRACDL	ENSG00000196872	343990	18.39	-3.07	-3.73	0.0001892	0.007460676	1.61376099	2.76697393	0	4.4228403	0	0.82301333	1.38503964	3.2192863	207.963299	8.86380261	3.95697085	16.7130252	13.06685	9.159940283	15.8225797	4.428413186	C2orf55|KIA	HGNC:HGNC:	2	2q11.2	CRACD like	protein-codir	CRACDL	CRACD like	O	CRACD-like p	20210302
GIPC3	ENSG00000179855	126326	22.86	-3.03	-3.73	0.0001893	0.007460676	48.9404604	20.9393765	0	0	0	2.50174494	9.82890652	2.75841815	42.0023603	26.0205338	8.40710308	52.6513021	48.2441942	37.16215845	37.8205645	28.47807447	C19orf64|DF	MIM:608792	19	19p13.3	GIPC PDZ dor	protein-codir	GIPC3	GIPC PDZ dor	O	PDZ domain-	20210302
RPLP2	ENSG00000177600	6181	7036.47	-0.66	-3.73	0.000189163	0.007460676	10419.9393	10092.3547	6064.36055	4131.04946	1983.52714	5085.26942	3874.91041	3741.08661	13041.7365	8757.57843	10209.9031	11155.9737	3754.43957	6694.074146	7130.71734	6446.594595	D11S2243E|L	MIM:180530	11	11p15.5	ribosomal pro	protein-codir	RPLP2	ribosomal pro	O	60S acidic rib	20210404
RPL11	ENSG00000142676	6135	5806.82	-0.63	-3.73	0.000193109	0.007595073	5583.50201	5776.90718	6697.97362	5001.90619	2001.21202	4234.67783	3210.65484	3172.45997	14590.844	6219.54722	7454.18037	9792.07407	3212.58792	4853.658757	5019.46642	6087.481305	DBA7|GIG34	MIM:604175	1	1p36.11	ribosomal pro	protein-codir	RPL11	ribosomal pro	O	60S ribosoma	20210410
SLC16A10	ENSG00000112394	117247	176.75	-2.80	-3.72	0.000198929	0.007772909	370.699427	89.7545537	166.650677	8.4442931	0	310.726419	1.78256207	7.34077154	194.593228	218.778565	153.07528	502.399602	43.911917	331.2601899	265.655303	162.9119117	MCT10|PRO(	MIM:607550	6	6q21	solute carrier	protein-codir	SLC16A10	solute carrier	O	monocarboxy	20210302
	ENSG00000255776		41.88	1.38	3.72	0.000199054	0.007772909	20.1395217	15.6816495	127.487246	36.5251199	99.3492443	22.3204671	43.2897861	132.266466	9.87985086	13.7941525	31.9591616	9.12145888	46.5496736	7.430160824	29.7195088	24.53742904											
KCNN4	ENSG00000104783	3783	36.19	-1.83	-3.72	0.000201393	0.007839905	84.7239683	20.6374141	0	8.13539957	9.48979723	31.3201604	7.6206059	3.67882315	79.64593	55.5002249	30.8143473	22.8505123	64.336869	104.506531	43.8000411	12.04658726	DHS2|IK|IK1	MIM:602754	19	19q13.31	potassium ca	protein-codir	KCNN4	potassium ca	O	intermediate	20210302
WDR5	ENSG00000196363	11091	140.04	-1.27	-3.71	0.000204454	0.007926648	84.3814376	176.848186	10.2384929	65.1578709	120.188453	49.2181499	123.702205	87.7589259	278.24527	274.127975	118.29209	201.815457	108.120696	187.390235	187.450671	167.6452792	BIG-3|CFAP8	MIM:609012	9	9q34.2	WD repeat do	protein-codir	WDR5	WD repeat do	O	WD repeat-co	20210418
PSTPIP2	ENSG00000152229	9050	135.87	1.03	3.71	0.000204122	0.007926648	56.2535217	144.595142	105.26749	217.613324	326.580437	250.023548	146.540665	230.299988	57.4162239	59.8463334	113.599449	57.5332678	153.844539	79.03058293	66.6845646	108.7239651	MAYP	MIM:616046	18	18q21.1|tdb	proline-serin	protein-codir	PSTPIP2	proline-serin	O	proline-serin	20210418
REG4	ENSG00000134193	83998	20.20	-3.53	-3.71	0.000204923	0.007928689	45.1833174	14.6593923	0	0	0	13.3255983	1.27302054	0	18.4898026	47.4664129	22.9686631	77.8488026	6.4912424	40.43463787	24.329576	10.73006678	GISP|REG-IV|	MIM:609846	1	1p12	regenerating	protein-codir	REG4	regenerating	O	regenerating	20210302
KDM6B	ENSG00000132510	23135	662.75	-0.98	-3.71	0.000205887	0.007949829	785.320068	523.941335	160.003944	286.90487	174.603729	609.921765	484.295831	632.181563	1473.94763	672.113526	708.338992	326.88999	723.164939	1270.969866	1142.10592	629.3519006	JMJD3|NEDC	MIM:611577	17	17p13.1	lysine demet	protein-codir	KDM6B	lysine demet	O	lysine-specifi	20210418

ITGA6	ENSG00000091409	3655	606.61	-1.81	-3.71	0.000207309	0.007983573	1162.71881	1172.00604	101.306892	243.631597	26.1072054	578.019003	96.2620901	83.1993247	680.708817	577.590596	854.13392	1476.77785	316.111821	842.3038376	1091.68194	403.1855326	CD49f|ITGA6	MIM:147556	2	2q31.1	integrin subu	protein-codir	ITGA6	integrin subu	O	integrin alpha	20210410
ARHGAP12	ENSG00000165322	94134	468.93	0.70	3.71	0.0002076	0.007983573	833.694912	532.114331	855.637139	842.103448	331.466535	568.009653	411.753737	348.176185	306.127666	308.200167	519.178284	423.819114	271.558515	264.8538624	387.165891	299.0745578	-	MIM:610577	10	10p11.22	Rho GTPase a	protein-codir	ARHGAP12	Rho GTPase a	O	rho GTPase-a	20210302
SNX32	ENSG00000172803	254122	5.96	-4.02	-3.71	0.000210116	0.008064057	0	0.92240496	0	0	0	0.59799369	0	0.91301114	26.3910617	24.110599	5.99885368	0.62140247	6.73326609	16.72433896	0.56523371	11.72186196	SNX6B	HGNC:HGNC:	11	11q13.1	sorting nexin	protein-codir	SNX32	sorting nexin	O	sorting nexin	20210302
CDH1	ENSG00000039068	999	18.81	-2.97	-3.70	0.000211515	0.008101391	5.59637972	5.72839804	4.45686956	1.93041634	0	10.8878716	0	1.12992122	130.645164	18.8006363	8.26811306	1.386797	3.14192647	51.7230497	12.8369553	44.4819912	Arc-1|BCDS1	MIM:192090	16	16q22.1	cadherin 1	protein-codir	CDH1	cadherin 1	O	cadherin-1|C	20210418
LAG3	ENSG00000089692	3902	93.49	0.90	3.70	0.000213515	0.008161601	56.0682727	104.409642	246.824484	186.549487	92.627393	55.7647147	72.3097497	179.384145	22.5462669	83.8606967	80.0786278	80.2879201	57.9436551	45.98874815	38.169035	92.95482956	CD223	MIM:153337	12	12p13.31	lymphocyte a	protein-codir	LAG3	lymphocyte a	O	lymphocyte a	20210418
STAT4	ENSG00000138378	6775	1164.77	0.65	3.70	0.000217459	0.00829568	834.034289	941.783967	1831.52993	1302.97104	1872.42292	1463.06781	1302.06995	1666.15611	259.692602	867.302724	1352.96208	849.540956	957.124719	979.5633095	988.631794	1167.545579	SLEB11	MIM:600558	2	2q32.2-q32.3	signal transdu	protein-codir	STAT4	signal transdu	O	signal transdu	20210418
SNORD10	ENSG00000238917	652966	1708.34	-2.73	-3.70	0.000218109	0.008303857	10145.4418	4482.9961	87.413148	291.933854	0	11.8877245	9.20201548	26.7185776	2732.6341	2805.48563	2093.55422	4268.40347	49.6971598	83.78162344	90.6135092	153.6150601	mgU6-77	HGNC:HGNC:	17	17p13.1	small nucleol	snoRNA	SNORD10	small nucleol	O	mgU6-77 sno	20210302
LINC00402	ENSG00000235532	100507612	153.95	-2.42	-3.70	0.000219747	0.008320248	370.226122	246.478208	0	18.2824153	15.8489737	137.961764	32.4178299	7.94353441	135.897153	195.748183	298.891364	241.827881	139.702784	256.7918122	269.167906	95.97866086	-	HGNC:HGNC:	13	13q22.1	long intergen	ncRNA	LINC00402	long intergen	O	-	20210302
PRAG1	ENSG00000275342	157285	149.88	-0.77	-3.69	0.000220288	0.008320248	167.439955	131.761742	47.4454879	53.5103467	220.218068	84.71011	166.415929	59.3297681	233.204618	173.620227	84.9684637	130.846301	276.186495	165.6628146	243.039127	159.7860726	NACK|PEAK2	MIM:617344	8	8p23.1	PEAK1 relate	protein-codir	PRAG1	PEAK1 relate	O	inactive tyros	20210302
FER	ENSG00000151422	2241	235.90	0.88	3.69	0.000220123	0.008320248	517.300386	241.410051	127.722457	328.141459	324.487594	241.275051	267.842697	301.827556	516.784369	153.363888	121.718504	123.829712	140.965475	151.5067995	82.1156925	134.1225623	PPP1R74|TYK	MIM:176942	5	5q21.3	FER tyrosine	protein-codir	FER	FER tyrosine	O	tyrosine-prot	20210302
PRPF38A	ENSG00000134748	84950	775.07	0.60	3.69	0.000221773	0.00835976	1072.23573	1078.75359	1498.76991	966.16421	628.444525	706.789937	1001.86763	605.73738	832.706508	682.92154	616.383886	548.802287	522.931607	513.5598491	544.150869	580.9729542	PRP38A|Prp3	MIM:617031	1	1p32.3	pre-mRNA pr	protein-codir	PRPF38A	pre-mRNA pr	O	pre-mRNA-sp	20210404
NAP1L1	ENSG00000187109	4673	4484.60	-0.59	-3.68	0.000229962	0.008651296	4480.73101	4855.67845	2587.46959	3341.87466	2791.15184	4619.70787	3182.04462	2915.79793	4446.06136	5213.44689	3764.05261	6346.67917	4091.40303	6276.687493	6844.32321	5996.473913	NAP1|NAP1L	MIM:164060	12	12q21.2	nucleosome a	protein-codir	NAP1L1	nucleosome a	O	nucleosome a	20210404
	ENSG00000244513		22.08	-3.20	-3.68	0.000231143	0.008667975	12.0249588	0	0	0	23.2876569	0.36050188	1.87174997	0.70286749	164.746503	104.296657	1.05495848	2.44475606	14.4424593	9.762901038	11.6887027	6.6493857											
PTPRC	ENSG00000081237	5788	19953.04	0.60	3.68	0.000232121	0.008681068	28606.149	30222.8342	28507.3676	26466.3083	19320.0693	18946.8231	19137.5281	20813.6796	9473.48708	17152.2044	20096.9662	20273.6107	15244.9499	13414.32648	12907.3933	18664.94022	B220|CD45|(	MIM:151460	1	1q31.3-q32.1	protein tyros	protein-codir	PTPRC	protein tyros	O	receptor-type	20210329
	ENSG00000260853		29.85	-2.74	-3.68	0.000233909	0.008713698	9.84528108	9.63124583	1.47763963	0	3.25924172	0	1.83913709	0.85084175	272.489688	141.558186	0	0	15.0507097	7.650656272	6.97242428	6.979466133											
SELL	ENSG00000188404	6402	1072.38	-2.65	-3.68	0.000233871	0.008713698	2186.7487	1537.62716	33.0240288	25.2328015	67.4414772	744.576277	100.790535	307.621023	1586.30147	1416.07434	1278.27478	2576.09783	746.566499	1958.535592	1713.49108	879.7249885	CD62L|LAM1	MIM:153240	1	1q24.2	selectin L	protein-codir	SELL	selectin L	O	L-selectin|CD	20210302
VASN	ENSG00000168140	114990	6.32	-4.44	-3.68	0.000234759	0.008728284	0.96654236	5.95994578	0	0	0	0	0	0	37.4700208	13.8130799	2.75506568	1.88038487	9.5727005	12.85422029	6.55627964	9.254666962	SLITL2	MIM:608843	16	16p13.3	vasorin	protein-codir	VASN	vasorin	O	vasorin|prot	20210418
CACHD1	ENSG00000158966	57685	35.90	-2.99	-3.68	0.000237773	0.008823084	132.721759	10.5755338	15.212538	1.30280405	0	10.0446057	1.02645392	0	174.160041	31.104482	38.4817398	61.156074	6.45998502	8.986506549	57.8866696	25.2824462	-	HGNC:HGNC:	1	1p31.3	cache domai	protein-codir	CACHD1	cache domai	O	VWFA and ca	20210302
PLLP	ENSG00000102934	51090	28.06	-2.81	-3.67	0.000238676	0.008826045	28.0617563	8.10187191	0	0	0	18.1866233	4.77341723	4.52843295	134.329003	131.62575	1.87628135	8.94790899	21.3030008	13.55975931	9.58290089	64.04025073	PMLP|TM4SF	MIM:600340	16	16q13	plasmolipin	protein-codir	PLLP	plasmolipin	O	plasmolipin|p	20210302
SCML1	ENSG00000047634	6322	166.25	-1.92	-3.67	0.00023878	0.008826045	373.73747	149.553777	15.0087399	66.9236211	6.22873043	209.767021	43.7063428	14.9940909	170.229582	205.861344	185.897173	245.969626	215.734195	305.4782262	360.776954	90.07243157	-	MIM:300227	X	Xp22.13	Scm polycom	protein-codir	SCML1	Scm polycom	O	sex comb on	20210302
	ENSG00000225339		249.23	-2.43	-3.67	0.000241401	0.008871236	19.7343031	10.9806288	11.4749207	5.88959659	40.3835324	4.39289663	13.6158983	25.2491122	1378.50879	2242.63752	27.6715615	7.003051	47.2381425	30.35312323	57.2030345	65.38320712											
RRS1	ENSG00000179041	23212	87.96	-0.86	-3.67	0.000241014	0.008871236	89.0525497	99.8203118	44.0007522	75.2596635	33.8161793	80.9060837	41.3036687	38.6519844	210.587246	144.678014	72.2851545	97.9156037	65.3991749	119.5553032	119.219042	74.97333677	-	MIM:618311	8	8q13.1	ribosome bio	protein-codir	RRS1	ribosome bio	O	ribosome bio	20210418
HECTD2	ENSG00000165338	143279	103.94	0.89	3.67	0.000240835	0.008871236	52.4979309	76.5477298	171.142667	189.766686	235.988747	124.909646	144.866552	108.046378	41.7745699	74.0118759	82.4176129	60.5317263	94.0474055	57.02661166	83.6183493	65.83923031	-	HGNC:HGNC:	10	10q23.32	HECT domain	protein-codir	HECTD2	HECT domain	O	probable E3 u	20210417
ARHGEF18	ENSG00000104880	23370	701.39	-0.98	-3.67	0.00024377	0.008906723	1159.468	702.292632	103.991721	272.183793	526.865408	538.96972	404.216868	401.5147	800.999202	850.396753	427.503938	619.084691	839.325367	1425.67793	1285.06612	864.7438026	P114-RhoGEF	MIM:616432	19	19p13.2	Rho/Rac guar	protein-codir	ARHGEF18	Rho/Rac guar	O	rho guanine r	20210302
GRK6	ENSG00000198055	2870	383.87	-0.81	-3.67	0.000243349	0.008906723	318.110141	396.17671	84.8419654	357.953383	185.200955	286.482748	327.542648	356.040843	498.940355	513.500433	355.927308	403.732474	448.886069	613.1048548	550.518794	444.9394518	GPRK6	MIM:600869	5	5q35.3	G protein-cou	protein-codir	GRK6	G protein-cou	O	G protein-cou	20210302
	ENSG00000225864		26.75	-2.65	-3.67	0.00024495	0.008926287	3.12270064	21.8350806	0	1.72270272	10.2211911	0	23.3528669	11.11078	70.0941597	82.4580565	7.85867079	1.426421	44.4870655	59.93204213	39.6220749	50.72077843											
SNORD116-2	ENSG00000207279	100033435	35.34	-2.63	-3.67	0.000246789	0.00894832	47.9977116	52.1984449	1.45193462	8.80361068	0	5.01144131	0	3.78578885	94.6165707	102.061022	95.0205743	113.916537	5.33176254	4.028016996	11.9616148	19.21167111	HBII-85-24	HGNC:HGNC:	15	15q11.2	small nucleol	snoRNA	SNORD116-2	small nucleol	O	-	20210302
IMPDH2	ENSG00000178035	3615	329.36	-1.21	-3.67	0.000246536	0.00894832	235.114229	262.381157	391.542587	103.437594	116.177636	100.985104	140.074942	105.445316	1701.02247	333.893432	258.991363	395.403419	210.627101	269.2805728	306.901255	338.4905042	IMPD2|IMPD	MIM:146691	3	3p21.31	inosine monc	protein-codir	IMPDH2	inosine monc	O	inosine-5'-mc	20210404
SDC2	ENSG00000169439	6383	21.17	-3.97	-3.66	0.000248225	0.008983309	4.33388994	0	72.2270381	6.58195694	0	0	0	0.49580126	117.897489	5.21507194	2.53265453	22.6410398	82.6598397	16.04538858	4.86441326	3.214161551	CD362|HSPG	MIM:142460	8	8q22.1	syndecan 2	protein-codir	SDC2	syndecan 2	O	syndecan-2|(	20210316
SNORD24	ENSG00000206611	26820	18.27	-1.85	-3.66	0.000250279	0.009040455	15.3221673	26.7304777	0	6.96732742	2.43576832	5.59925647	3.77636816	7.04973592	39.8535932	65.8008211	17.930254	13.7835985	12.658943	16.15172898	14.8110385	43.44126766	RNU24|U24	HGNC:HGNC:	9	9q34.2	small nucleol	snoRNA	SNORD24	small nucleol	O	RNA, U24 sm	20210302
LOC1224553	ENSG00000284526	122455342	51.16	-2.96	-3.66	0.000253534	0.009115188	3.26664894	22.298236	6.09318551	3.29210203	0	0	8.30959096	8.3211918	301.68759	375.495883	2.61620969	5.40264281	13.2034616	17.50263817	22.1759737	28.92006399											
LOC1053714	ENSG00000260279	105371414	9.75	-2.83	-3.66	0.000254743	0.009115188	5.77755604	1.08635908	0	0	5.50303847	0.92205318	2.28164434	1.61214134	36.7217412	50.3490925	1.62173344	5.08681674	14.1687451	11.79820032	14.5225297	4.516596094	-	Ensembl:ENS	16	16q24.3	uncharacteriz	ncRNA	-	-	-	-	20210302
DPP4	ENSG00000197635	1803	245.85	-1.32	-3.66	0.000254708	0.009115188	484.195452	526.117174	66.2502169	106.184702	55.8056366	92.3739655	83.7726086	58.8770396	315.707377	428.214248	203.844055	328.109071	175.316391	290.3387441	404.324523	314.2122821	ADABP|ADCF	MIM:102720	2	2q24.2	dipeptidyl pe	protein-codir	DPP4	dipeptidyl pe	O	dipeptidyl pe	20210322
SNHG29	ENSG00000175061	125144	1300.91	-0.66	-3.66	0.000253919	0.009115188	1535.16929	1406.49941	1039.52406	881.851591	582.65789	1140.68891	701.163261	803.342917	2472.19404	1191.12344	1404.40184	2280.84551	1008.87872	1475.693024	1391.29986	1499.242059	C17orf45|C1	HGNC:HGNC:	17	17p11.2	small nucleol	ncRNA	SNHG29	small nucleol	O	C17orf76 ant	20210418
XRCC6	ENSG00000196419	2547	835.67	-0.65	-3.66	0.000253233	0.009115188	727.465083	805.228014	427.2322	539.68871	440.771799	938.318119	541.069312	719.18772	2004.92281	867.05353	758.197239	981.577817	679.921223	956.6722975	941.620782	1041.804022	CTC75|CTCBI	MIM:152690	22	22q13.2	X-ray repair c	protein-codir	XRCC6	X-ray repair c	O	X-ray repair c	20210404
WDR86	ENSG00000187260	349136	8.94	-3.27	-3.66	0.000255235	0.00911569	3.67442066	0.80682686	0	1.56855935	0	0	5.34573046	0.62446829	48.4471321	20.3363771	1.79200353	4.97548366	10.1750092	25.29043455	18.4942342	1.521480712	-	HGNC:HGNC:	7	7q36.1	WD repeat dc	protein-codir	WDR86	WD repeat dc	O	WD repeat-cc	20210302
LEF1-AS1	ENSG00000232021	641518	72.75	-2.89	-3.65	0.000264545	0.009412856	181.372372	63.870985	0	0	0	42.1493693	27.6544571	9.10193166	110.599186	78.1897714	90.1741749	148.833433	69.1815594	178.4488392	128.206584	36.15122385	LEF1NAT	HGNC:HGNC:	4	4q25	LEF1 antisens	ncRNA	LEF1-AS1	LEF1 antisens	O	LEF1 antisens	20210302
IL7R	ENSG00000168685	3575	12185.02	-1.29	-3.65	0.000264474	0.009412856	24384.5871	19210.7753	1901.32672	3520.78835	1320.88447	15961.6877	4531.50149	4755.31763	14012.3473	15529.4515	12416.5203	12402.9352	6324.76686	24679.53331	19667.5857	14340.30417	CD127|CDW:	MIM:146661	5	5p13.2	interleukin 7	protein-codir	IL7R	interleukin 7	O	interleukin-7	20210329
RPL37A	ENSG00000197756	6168	3046.61	-0.76	-3.65	0.000265353	0.009423998	2635.41824	2625.69895	3407.24104	2212.34079	920.851058	2200.20571	1472.03302	2059.16055	9254.43985	3513.43814	2939.96419	4718.76395	1922.87752	2753.850486	2467.72809	3641.80408	L37A	MIM:613314	2	2q35	ribosomal prc	protein-codir	RPL37A	ribosomal prc	O	60S ribosoma	20210404
CLEC2B	ENSG00000110852	9976	954.15	0.61	3.65	0.000266262	0.009434513	464.704512	951.997503	2057.98688	1654.22759	1000.66472	1196.31309	977.622549	891.251847	379.517213	517.442783	1370.99485	1131.25175	755.47593	423.8626724	726.157898	766.9298393	AICL|CLECSF	MIM:603242	12	12p13.31	C-type lectin	protein-codir	CLEC2B	C-type lectin	O	C-type lectin	20210302
	ENSG00000211829		253.94	1.25	3.65	0.00026664	0.009434513	38.1399087	66.0893379	2319.17588	138.629277	337.519348	129.539506	244.060241	9.84829941	21.7727305	23.3926081	345.112424	43.5221888	194.961683	48.97259681	82.1409157	20.18981368											
GIMAP8	ENSG00000171115	155038	415.93	-0.85	-3.64	0.000268364	0.009477871	467.644422	402.475641	213.643626	157.248153	268.263624	490.533804	293.076968	176.675663	363.023746	485.407202	462.176744	487.027345	582.830503	747.6428645	625.552485	431.728058	IAN-9|IAN6|I	MIM:616962	7	7q36.1	GTPase, IMAI	protein-codir	GIMAP8	GTPase, IMAI	O	GTPase IMAP	20210302
RANGAP1	ENSG00000100401	5905	200.89	-0.97	-3.64	0.000269489	0.009500001	155.527604	120.094756	46.4993547	70.4247759	148.122579	193.37391	195.781321	155.636738	604.847203	214.440123	207.407766	165.48104	204.406134	224.1817837	274.811602	233.2213634	Fug1|RANGA	MIM:602362	22	22q13.2	Ran GTPase a	protein-codir	RANGAP1	Ran GTPase a	O	ran GTPase-a	20210302
HOOK1	ENSG00000134709	51361	252.29	-1.80	-3.64	0.000271127	0.009540056	882.990586	252.159158	0	22.0226879	24.7710616	175.377148	46.0963819	29.6341103	1392.94921	199.599012	171.222767	176.280706	117.557531	207.6776106	236.780347	101.5995666	HK1	MIM:607820	1	1p32.1	hook microtu	protein-codir	HOOK1	hook microtu	O	protein Hook	20210302
RPS15A	ENSG00000134419	6210	4455.30	-0.85	-3.64	0.000274886	0.009654469	3276.1189	3215.62541	3699.69348	2655.85748	1712.5036	3179.27867	2312.91234	3013.35762	17077.423	3557.62096	4424.80829	6762.48899	2980.66917	4094.326456	4245.97245	5076.153155	DBA20|S15a	MIM:603674	16	16p12.3	ribosomal prc	protein-codir	RPS15A	ribosomal prc	O	40S ribosoma	20210410
RPL7A	ENSG00000148303	6130	4231.75	-0.64	-3.64	0.000276888	0.009697557	3645.09792	3346.07053	3477.10934	3150.02012	2207.98751	3482.05888	2874.83861	3169.50082	11324.9444	3825.31639	4297.84251	5730.9094	3086.32095	4341.060516	3864.52768	5884.327741	L7A|SURF3|1	MIM:185640	9	9q34.2	ribosomal prc	protein-codir	RPL7A	ribosomal prc	O	60S ribosoma	20210404
RNPC3	ENSG00000185946	55599	847.32	0.73	3.64	0.000277131	0.009697557	1270.22459	1546.86898	619.327967	1013.39865	1383.75766	888.264207	898.206259	873.612263	546.836328	569.59222	649.053888	632.82986	822.245732	765.2913739	713.635049	363.9309713	IGHD5|RBM4	MIM:618016	1	1p21.1	RNA binding	protein-codir	RNPC3	RNA binding	O	RNA-binding	20210417
NR2C1	ENSG00000120798	7181	294.50	0.76	3.63	0.000278084	0.009713045	452.912353	188.703592	589.406502	487.723947	402.495314	341.450697	326.456155	214.535023	164.232542	234.388826	294.432245	260.414018	181.411475	205.5552799	235.03763	132.862868	TR2	MIM:601529	12	12q22	nuclear recep	protein-codir	NR2C1	nuclear recep	O	nuclear recep	20210302
	ENSG00000287086		19.05	-4.16	-3.63	0.000288861	0.010020821	2.50996596	1.55885246	0	0	0	0	0	4.09690443	93.2151661	148.69859	0	0	13.4755158	15.74649966	9.35365624	16.16017788											
GBP2	ENSG00000162645	2634	1447.58	0.66	3.62	0.000289001	0.010020821	598.938005	1748.43699	2819.22824	1574.74186	2076.29802	1659.16729	1646.73027	2383.6738	791.941056	1061.6126	1355.90616	1183.924	937.435613	1093.093363	998.839828	1231.304124	-	MIM:600412	1	1p22.2	guanylate bir	protein-codir	GBP2	guanylate bir	O	guanylate-bir	20210302
HABP4	ENSG00000130956	22927	136.91	-0.90	-3.62	0.000291242	0.010080153	191.601396	119.002331	74.268168	59.535395	15.6983448	152.103653	107.348938	71.4203586	359.266584	168.994869	93.725941	131.721448	109.42514	212.9952316	186.46326	137.0550049	IHABP-4|IHA	MIM:617369	9	9q22.32	hyaluronan b	protein-codir	HABP4	hyaluronan b	O	intracellular h	20210302
NUAK2	ENSG00000163545	81788	203.14	-0.70	-3.62	0.000293375	0.010117177	115.988575	173.657562	99.1862142	248.384199	109.645172	192.574603	193.857777	127.237202	255.82292	209.124495	225.590135	283.908578	239.340449	273.7019229	257.556318	244.6102821	SNARK	MIM:608131	1	1q32.1	NUAK family	protein-codir	NUAK2	NUAK family	O	NUAK family	20210316
SH3TC1	ENSG00000125089	54436	41.32	-1.45	-3.62	0.000298046	0.010167759	10.9050487	11.7186659	7.58560404	22.8236979	88.6957791	17.2578809	8.00290625	32.2297811	22.2511666	31.2931854	22.5936873	28.8782564	112.983699	94.07316307	49.9813058	99.78938412	-	HGNC:HGNC:	4	4p16.1	SH3 domain a	protein-codir	SH3TC1	SH3 domain a	O	SH3 domain a	20210302
PTRH2	ENSG00000141378	51651	86.27	-1.41	-3.62	0.000296055	0.010167759	76.8412175	61.3937767	21.9027486	99.8393754	8.77782406	59.9861847	21.0476751	63.940679	98.8672948	217.065705	98.1756184	207.581037	82.7501798	92.85068714	105.090937	64.21892509	BIT1|CFAP37	MIM:608625	17	17q23.1	peptidyl-tRNA	protein-codir	PTRH2	peptidyl-tRNA	O	peptidyl-tRNA	20210404
AKR1A1	ENSG00000117448	10327	95.28	-0.99	-3.62	0.000297029	0.010167759	144.388075	76.6055939	85.0253504	33.3906646	17.1790617	48.738243	49.9887713	41.9243493	396.532996	128.05188	109.58947	82.5940356	73.4450649	88.06959126	100.708885	48.19223472	ALDR1|ALR|/	MIM:103830	1	1p34.1	aldo-keto rec	protein-codir	AKR1A1	aldo-keto rec	O	aldo-keto rec	20210406
LOC1053709	ENSG00000261136	105370941	67.29	1.09	3.62	0.000295715	0.010167759	30.3418339	98.8097886	213.40906	83.5767867	117.559458	81.6084825	67.4425138	58.8533758	30.6216832	30.3672472	46.6828237	37.8307984	59.7369708	53.28032947	41.1321167	25.37658039	-	Ensembl:ENS	15	15q14	uncharacteriz	ncRNA	-	-	-	-	20210302
SNX14	ENSG00000135317	57231	1010.07	0.64	3.62	0.000298757	0.010173764	1002.75845	643.772153	2262.78122	1391.36433	1310.97424	991.630775	1105.08015	1325.9827	608.327745	692.979468	824.192103	793.817318	945.144844	775.0255804	785.876744	701.4592922	RGS-PX2|SCA	MIM:616105	6	6q14.3	sorting nexin	protein-codir	SNX14	sorting nexin	O	sorting nexin	20210302
CARMIL1	ENSG00000079691	55604	73.55	-1.88	-3.61	0.000305326	0.010378915	227.5516	41.0064356	22.9693221	34.3923373	0	34.9408839	14.6687058	2.68942616	302.30082	60.02867	42.13339	80.5253981	65.4960316	111.1142325	107.34468	29.56879336	CARMIL|CAR	MIM:609593	6	6p22.2	capping prote	protein-codir	CARMIL1	capping prote	O	F-actin-uncap	20210302
KIFC1	ENSG00000237649	3833	60.83	-3.49	-3.61	0.000308179	0.010438596	6.08388303	2.85889762	0	0	0	0	6.31515678	3.95085186	601.232287	330.128974	2.70110138	4.67398895	4.70819442	4.135249289	1.47583034	4.963782333	HSET|KNSL2	MIM:603763	6	6p21.32	kinesin family	protein-codir	KIFC1	kinesin family	O	kinesin-like p	20210404
TMED2	ENSG00000086598	10959	977.81	-0.62	-3.61	0.000309649	0.01046978	728.516757	937.83144	560.951817	942.009494	707.566258	746.055136	691.548898	773.056044	1942.5046	983.644178	1058.59484	1310.84388	824.649086	977.888121	1045.08881	1414.28414	P24A|RNP24	HGNC:HGNC:	12	12q24.31	transmembra	protein-codir	TMED2	transmembra	O	transmembra	20210302
TGFA	ENSG00000163235	7039	32.78	-2.17	-3.60	0.000312596	0.010550674	22.8289437	28.1990654	0	3.60074255	5.08268884	0.60627219	48.2383071	15.4001726	125.531001	31.0412237	18.5767902	7.09642032	44.0268032	23.21770573	63.7289603	87.31604347	TFGA	MIM:190170	2	2p13.3	transforming	protein-codir	TGFA	transforming	O	protransform	20210302
LCMT2	ENSG00000168806	9836	203.37	1.07	3.60	0.000313523	0.010563228	127.857738	88.8828564	437.080155	283.729325	578.442602	382.640777	199.28776	175.595199	71.6745064	152.453874	132.279543	85.5682048	207.509396	113.2811617	126.894234	90.74562656	PPM2|TYW4	MIM:611246	15	15q15.3	leucine carbc	protein-codir	LCMT2	leucine carbc	O	tRNA wybuto	20210302
	ENSG00000259375		18.66	-3.57	-3.60	0.000317222	0.010642655	10.2498671	2.74024975	0	1.84799342	0	0	0	0.63093223	195.843829	47.0773155	0	0	4.35818812	8.18435501	17.438366	10.22295189											
MAGED4B	ENSG00000187243	81557	15.83	-3.41	-3.60	0.00031719	0.010642655	2.18955995	2.47707601	0	0	0	56.7668915	0	0	13.162946	4.78661089	15.9212401	6.82518395	2.30831466	120.4455107	7.57038589	20.76494403	-	MIM:300765	X	Xp11.22	MAGE family	protein-codir	MAGED4B	MAGE family	O	melanoma-as	20210302
HPCAL4	ENSG00000116983	51440	72.65	-2.69	-3.60	0.000317558	0.010642655	135.122597	146.635148	22.304443	0	2.44929159	40.319917	2.05746221	5.7473074	66.4384276	135.539828	124.78313	161.828287	50.3811653	92.88687065	132.315	43.5894155	HLP4	MIM:619211	1	1p34.2	hippocalcin li	protein-codir	HPCAL4	hippocalcin li	O	hippocalcin-li	20210302
	ENSG00000273489		20.17	-4.58	-3.59	0.000325919	0.010884535	1.19764991	0	0	0	0	0	0	0.6179862	137.799318	152.29341	0	0	7.47427687	4.871559248	7.6867398	10.8461373											
ATP6	ENSG00000198899	4508	34245.78	0.80	3.59	0.000329095	0.010971359	79468.922	94226.1111	73368.1534	25115.4242	14483.2279	23974.0981	20487.2515	21672.4899	64937.9243	22176.4532	38743.2692	11449.4913	13381.5439	10528.20924	13204.6954	20715.27139	ATPase6|MT.	MIM:516060	MT	-	ATP synthase	protein-codir	MT-ATP6	mitochondria	O	ATP synthase	20210407
EHBP1	ENSG00000115504	23301	371.27	0.74	3.59	0.000334445	0.011130252	851.880797	473.510049	333.983891	520.807648	222.329246	580.200419	327.647352	441.26297	437.861938	263.653435	299.246592	351.089522	189.649474	164.8815163	227.729642	254.6616815	HPC12|NACS	MIM:609922	2	2p15	EH domain bi	protein-codir	EHBP1	EH domain bi	O	EH domain-b	20210302
SNORD116-1	ENSG00000207063	100033413	31.47	-2.72	-3.59	0.000337076	0.011198202	82.5733046	64.9157168	0	2.3475081	0	4.039515	0	6.36010886	79.1522528	100.036863	39.0266433	65.4688888	3.04421313	21.81861323	15.5564557	19.22197124	HBII-85-1|PW	MIM:605436	15	15q11.2	small nucleol	snoRNA	SNORD116-1	small nucleol	O	-	20210418
EDAR	ENSG00000135960	10913	16.90	-3.65	-3.58	0.000343791	0.011401411	23.0926167	24.79732	0	0	0	13.2520739	0	0.62128977	27.4394858	29.7494994	14.1500674	62.1308357	24.2971359	15.84289304	18.0534372	16.97539404	DL|ECTD10A	MIM:604095	2	2q13	ectodysplasir	protein-codir	EDAR	ectodysplasir	O	tumor necros	20210408
GPR183	ENSG00000169508	1880	320.21	-1.52	-3.58	0.00034502	0.011422262	338.101549	356.701322	62.3345965	86.2356273	60.627169	316.070919	149.657078	92.6850806	141.26746	314.35774	687.835967	472.394101	227.710345	919.2834226	416.184484	481.8723944	EBI2|hEBI2	MIM:605741	13	13q32.3	G protein-cou	protein-codir	GPR183	G protein-cou	O	G-protein cou	20210302
WDR86-AS1	ENSG00000243836	100131176	7.33	-3.91	-3.58	0.000345782	0.011427607	2.07557615	0	0	2.07332833	0	0	0	6.55805847	46.502971	6.86445667	5.2689299	4.66897731	7.25154634	9.999161829	16.1819375	9.789569984	-	HGNC:HGNC:	7	7q36.1	WDR86 antis	ncRNA	WDR86-AS1	WDR86 antis	O	WDR86 antis	20210302
	ENSG00000258430		14.13	-2.10	-3.58	0.000348451	0.01149587	20.4418242	7.30178889	0	0	8.18737537	5.99133935	2.22499275	5.96407166	48.2137931	23.068612	8.27576726	3.82714449	20.9656224	16.19350736	29.3454849	26.1243116											
GPR141	ENSG00000187037	353345	286.02	1.15	3.57	0.000350683	0.011549473	49.567144	113.95699	203.813885	540.329625	1043.94185	380.408305	538.13887	313.477027	25.3618131	143.46986	191.979441	184.818706	526.467518	79.39253262	89.7527262	151.3772858	PGR13	MIM:609045	7	7p14.1	G protein-cou	protein-codir	GPR141	G protein-cou	O	probable G-p	20210302
SNHG8	ENSG00000269893	100093630	329.08	-0.90	-3.57	0.000351413	0.011553539	494.410323	282.047072	111.997978	277.910772	57.673896	201.938457	265.18405	86.9244869	716.76335	324.121655	483.88034	1025.87226	91.0960307	250.6334428	420.917664	173.9141922	LINC00060|N	HGNC:HGNC:	4	4q26	small nucleol	ncRNA	SNHG8	small nucleol	O	long intergen	20210418
ADGRG1	ENSG00000205336	9289	964.17	0.97	3.57	0.000352752	0.011577578	362.087347	770.180142	1682.9448	1676.59209	1381.51987	1099.69959	1693.34917	1456.11901	416.816072	452.539858	553.341368	741.052799	1447.03349	303.6194835	402.460003	987.2888907	BFPP|BPPR|(	MIM:604110	16	16q21	adhesion G p	protein-codir	ADGRG1	adhesion G p	O	adhesion G-p	20210409
	ENSG00000243004		123.30	-2.27	-3.57	0.000355077	0.011633819	56.8807676	22.4536684	0	3.92641651	0	13.662022	5.7910246	8.01255347	900.231122	892.609972	1.63722075	7.00667556	14.5156738	15.74006641	11.0718539	19.20332481											
RAD54B	ENSG00000197275	25788	13.81	-3.90	-3.56	0.000365118	0.011942254	15.3399867	0	0	21.4303943	0	0	14.2161181	0	12.0629858	13.0263414	15.7800989	41.8691803	20.7460852	22.65888293	26.6253028	17.17068497	RDH54	MIM:604289	8	8q22.1	RAD54 homo	protein-codir	RAD54B	RAD54 homo	O	DNA repair ar	20210417
ANKRD18EP	ENSG00000217165	100131031	17.36	-4.12	-3.56	0.000367453	0.011977469	0	1.61253668	3.8081373	0	0	0	0.60520583	0	100.178109	153.289831	0.83644868	0.81516774	3.48149765	4.899808198	3.72073686	4.477716536	-	HGNC:HGNC:	6	6p21.2	ankyrin repea	pseudo	ANKRD18EP	ankyrin repea	O	uncharacteriz	20210302
	ENSG00000231329		20.21	-2.86	-3.56	0.000367247	0.011977469	3.65929027	11.2807839	0	0	10.5985062	0	3.53397379	2.44117509	82.8169123	149.595526	2.20517806	0.67601192	5.90299273	12.54367591	13.7876537	24.36250268											
SNORA73B	ENSG00000200087	26768	868.00	-1.57	-3.56	0.000368489	0.01199071	3520.68219	1442.9111	66.8187501	260.836044	60.3697879	204.046734	146.333646	277.117233	1596.98909	1199.15026	1296.73739	1665.75942	308.802216	454.7182303	509.946471	876.8458319	105A|E1c|RN	MIM:603239	1	1p35.3	small nucleol	snoRNA	SNORA73B	small nucleol	O	RNA, U105A s	20210302
ZNF285	ENSG00000267508	26974	9.16	-4.95	-3.56	0.000377551	0.012233268	15.0494909	17.9858254	0	0	0	0	0	0	6.22359537	3.9402172	12.2952181	28.3232686	15.7935365	10.99436662	25.1689431	10.84680086	ZNF285A	HGNC:HGNC:	19	19q13.31	zinc finger pr	protein-codir	ZNF285	zinc finger pr	O	zinc finger pr	20210302
OSM	ENSG00000099985	5008	15.40	-3.41	-3.55	0.000378514	0.012233268	3.07723977	1.78669233	0	0	0	0	0	0.69558121	213.49928	10.7669472	2.06081366	3.87168807	3.84930649	0.871996276	0.90330107	5.059240393	-	MIM:165095	22	22q12.2	oncostatin M	protein-codir	OSM	oncostatin M	O	oncostatin-M	20210329
SNORD12	ENSG00000212304	692057	46.91	-2.33	-3.56	0.000377952	0.012233268	19.6779386	37.4502467	1.32279826	7.36291972	0	43.992048	8.72983901	39.623813	33.7888666	36.6123019	141.010196	102.009434	44.0310419	81.06273975	36.7798663	117.160492	HBII-99|MIR:	HGNC:HGNC:	20	20q13.13	small nucleol	snoRNA	SNORD12	small nucleol	O	hsa-mir-1259	20210302
FAM241A	ENSG00000174749	132720	102.11	-1.20	-3.55	0.000386324	0.012464507	90.2355238	124.535119	20.0882328	20.8184711	79.5484477	55.0391556	61.2183302	73.5308929	211.48404	139.592494	100.775191	130.304774	103.262699	167.8822849	170.132772	85.29750454	C4orf32	HGNC:HGNC:	4	4q25	family with se	protein-codir	FAM241A	family with se	O	uncharacteriz	20210302
NPC2	ENSG00000119655	10577	130.49	-1.09	-3.55	0.000387878	0.012472381	195.852059	95.9933526	21.5481557	139.711354	44.9897676	102.942894	32.3791717	85.524736	302.501531	136.179499	177.105004	234.333598	140.682501	119.4382037	104.544553	154.041046	EDDM1|HE1	MIM:601015	14	14q24.3	NPC intracell	protein-codir	NPC2	NPC intracell	O	NPC intracell	20210410
MRE11	ENSG00000020922	4361	441.89	0.67	3.55	0.000387272	0.012472381	405.019013	228.479348	772.878772	596.596069	913.387819	629.384853	478.478117	385.989503	233.132396	254.966575	360.368454	404.721002	453.576152	308.5647872	442.205173	202.5382246	ATLD|HNGS1	MIM:600814	11	11q21	MRE11 homc	protein-codir	MRE11	MRE11 homc	O	double-stran	20210418
RNY4	ENSG00000252316	6086	29.06	2.35	3.55	0.000388544	0.01247273	72.5043705	58.5303059	11.7533712	23.3799173	17.3187907	97.8601512	28.0899825	78.0662898	7.59018394	13.5899156	18.0503009	13.5852641	8.71868185	1.505543362	0.79560665	13.67753434	HY4|Y4	MIM:601823	7	7q36.1	RNA, Ro60-as	ncRNA	RNY4	RNA, Ro60-as	O	RNA, Ro-asso	20210302
ZNF552	ENSG00000178935	79818	67.30	-0.90	-3.55	0.000389633	0.012486627	93.6601164	33.8317342	34.7982259	57.5306301	58.1998833	44.2902899	25.5389065	35.0819199	120.505295	97.9925595	53.2290963	141.220544	82.6511554	74.7981396	65.3308343	58.09928117	-	HGNC:HGNC:	19	19q13.43	zinc finger pr	protein-codir	ZNF552	zinc finger pr	O	zinc finger pr	20210302
MTFMT	ENSG00000103707	123263	81.27	-1.58	-3.54	0.000393462	0.012545967	46.6059488	43.9625418	3.2123856	18.5887239	43.1275458	43.2958793	121.509921	37.7781643	285.551032	102.236319	107.582144	65.0267462	121.376674	72.37531688	96.6688474	91.37774962	COXPD15|FN	MIM:611766	15	15q22.31	mitochondria	protein-codir	MTFMT	mitochondria	O	methionyl-tR	20210302
BEX4	ENSG00000102409	56271	133.63	-0.93	-3.54	0.000393071	0.012545967	128.56039	154.381146	115.861631	39.6251895	39.5572326	139.667531	94.8849706	47.7880368	315.509811	208.397009	137.738968	169.401829	87.467907	155.9705111	194.438556	108.7884386	BEXL1	MIM:300692	X	Xq22.1	brain express	protein-codir	BEX4	brain express	O	protein BEX4	20210417

ANKRD36B	ENSG00000196912	57730	550.88	0.61	3.54	0.000393406	0.012545967	628.845996	487.552608	501.796265	774.777686	812.947493	814.874279	750.212388	467.698937	283.780866	258.892176	584.65034	560.141732	539.584912	454.6329066	630.870023	262.7526978	KIAA1641	HGNC:HGNC:	2	2q11.2	ankyrin repea	protein-codir	ANKRD36B	ankyrin repea	O	ankyrin repea	20210324
WDR31	ENSG00000148225	114987	8.58	-3.78	-3.54	0.000399211	0.01270338	1.0419697	3.13530466	0	0	0	0	0	2.00573378	75.5797986	11.7188286	6.3141567	6.2459225	3.54665027	21.30338212	1.45708379	4.929900633	-	HGNC:HGNC:	9	9q32	WD repeat d	protein-codir	WDR31	WD repeat d	O	WD repeat-c	20210302
KCTD7	ENSG00000243335	154881	367.98	0.78	3.54	0.000399733	0.01270338	417.843526	229.744049	666.924848	449.054582	383.693698	825.151488	412.627236	369.299919	165.5882	189.364678	178.096954	254.955948	349.73336	433.6262937	282.487806	279.4386001	CLN14|EPM3	MIM:611725	7	7q11.21	potassium ch	protein-codir	KCTD7	potassium ch	O	BTB/POZ don	20210410
NUTM2D	ENSG00000214562	728130	28.34	-1.66	-3.54	0.000400571	0.012708793	20.2687738	6.77495423	0	5.84022663	19.2625571	24.8111742	10.2277964	39.8533679	32.9650765	35.0525053	18.9940721	39.2646382	59.8219009	30.89809532	44.5238098	64.88842478	FAM22D	HGNC:HGNC:	10	10q23.2	NUT family m	protein-codir	NUTM2D	NUT family m	O	NUT family m	20210403
SNORD116-2	ENSG00000207001	100033414	19.12	-3.24	-3.54	0.000402414	0.012722079	54.1558354	27.2664464	0	2.36644832	0	0	0	6.99428036	69.8170122	33.8772755	22.2365108	35.7033527	17.1851374	8.115178501	17.2501656	10.95225043	HBII-85-2	HGNC:HGNC:	15	15q11.2	small nucleol	snoRNA	SNORD116-2	small nucleol	O	-	20210302
HK2	ENSG00000159399	3099	88.73	-2.71	-3.54	0.000403663	0.012722079	75.0009851	99.8441392	94.981683	8.40312671	4.79907547	26.6495715	0	0.64661937	679.803727	64.296607	34.5488771	61.9828705	86.4843292	57.20992641	89.9320251	35.02424717	HKII|HXK2	MIM:601125	2	2p12	hexokinase 2	protein-codir	HK2	hexokinase 2	O	hexokinase-2	20210418
RCOR3	ENSG00000117625	55758	870.89	0.63	3.54	0.000401731	0.012722079	1202.55883	816.352028	1173.45762	993.820368	1716.03405	969.482004	850.311552	819.486172	473.96665	719.378912	860.229895	671.924845	654.120397	737.6133141	684.948303	590.5205804	-	HGNC:HGNC:	1	1q32.2-q32.3	REST corepre	protein-codir	RCOR3	REST corepre	O	REST corepre	20210417
ANKRD28	ENSG00000206560	23243	618.83	0.75	3.54	0.000403383	0.012722079	540.851066	506.643244	470.105522	1187.43464	1690.86992	451.072831	661.305902	917.855137	311.612444	475.069416	431.920264	565.90767	520.508674	409.7552592	329.396555	430.9760938	CFAP79|FAP	MIM:611122	3	3p25.1	ankyrin repea	protein-codir	ANKRD28	ankyrin repea	O	serine/threor	20210302
ADHFE1	ENSG00000147576	137872	169.64	0.87	3.54	0.000405103	0.012746352	92.2150737	153.37998	335.053053	140.032977	347.577292	245.780104	223.150599	213.496257	78.7146794	118.988913	154.099563	72.5130915	183.218044	183.5773899	125.780192	46.71568876	ADH8|HMFT	MIM:611083	8	8q13.1	alcohol dehy	protein-codir	ADHFE1	alcohol dehy	O	hydroxyacid-	20210302
GRAP	ENSG00000154016	10750	83.59	-1.91	-3.53	0.000409106	0.012824472	77.3738735	93.2276832	2.37041023	26.331843	2.66593338	39.0677567	64.624221	45.7003699	62.8177983	66.2538811	60.6065495	153.242817	122.189052	137.3441254	232.025385	151.5437446	DFNB114	MIM:604330	17	17p11.2	GRB2 related	protein-codir	GRAP	GRB2 related	O	GRB2-related	20210302
XBP1	ENSG00000100219	7494	576.22	-0.69	-3.53	0.000409607	0.012824472	527.933963	515.098431	217.818719	325.559172	233.206856	392.16905	520.541051	890.837194	800.502468	561.758507	727.906021	712.086947	388.095339	639.6971153	624.814467	1141.471768	TREB-5|TREB	MIM:194355	22	22q12.1|22q	X-box binding	protein-codir	XBP1	X-box binding	O	X-box-binding	20210418
PPP3CC	ENSG00000120910	5533	785.63	0.61	3.53	0.000409311	0.012824472	623.202083	682.762117	981.538854	1297.79553	851.135922	1274.28687	1110.44579	787.800856	328.43442	722.953701	847.376241	664.765357	605.302313	571.6115712	600.19331	620.4168811	CALNA3|CNA	MIM:114107	8	8p21.3	protein phos	protein-codir	PPP3CC	protein phos	O	serine/threor	20210302
LINC02273	ENSG00000245954	100996286	48.83	-2.58	-3.53	0.000413231	0.012916693	130.298453	10.5706874	0	23.0307999	0	38.6209279	0.72548925	17.849375	87.8728025	31.2357144	84.5151448	135.481481	25.9353537	79.54971191	77.9348603	37.70012673	-	HGNC:HGNC:	4	4q31.3	long intergen	ncRNA	LINC02273	long intergen	O	-	20210302
EIF4G3	ENSG00000075151	8672	597.98	0.78	3.53	0.00041753	0.013008333	374.317847	479.09355	938.694896	841.933613	1197.11518	751.977695	709.731825	765.549747	539.04183	355.597864	454.454039	377.795509	707.624226	266.9411796	336.004983	471.7425721	eIF-4G 3|eIF	MIM:603929	1	1p36.12	eukaryotic tr	protein-codir	EIF4G3	eukaryotic tr	O	eukaryotic tr	20210417
SYNJ1	ENSG00000159082	8867	599.33	1.20	3.53	0.000417231	0.013008333	154.023589	232.017046	3552.01677	706.834156	1184.17093	636.791856	591.73876	416.205887	169.782864	241.495072	359.062031	235.470437	310.810538	251.367972	236.476949	311.0793016	DEE53|EIEE5	MIM:604297	21	21q22.11	synaptojanin	protein-codir	SYNJ1	synaptojanin	O	synaptojanin-	20210406
ALS2CL	ENSG00000178038	259173	24.34	-2.81	-3.52	0.000425969	0.013212716	9.61592362	6.1544357	3.15262899	0	14.8471284	0	0.46819031	2.98213666	243.128058	13.6069236	3.78505068	3.33290456	38.3848967	30.9135539	12.7457148	6.33389304	RN49018	MIM:612402	3	3p21.31	ALS2 C-termi	protein-codir	ALS2CL	ALS2 C-termi	O	ALS2 C-termi	20210302
IP6K1	ENSG00000176095	9807	307.20	-0.76	-3.52	0.000426172	0.013212716	99.1929765	87.0724028	151.052288	370.407385	386.025988	305.150872	260.306407	263.900198	242.499221	207.951585	361.005769	409.120411	401.978328	350.5960993	466.866208	552.1406422	IHPK1|PiUS	MIM:606991	3	3p21.31	inositol hexal	protein-codir	IP6K1	inositol hexal	O	inositol hexal	20210302
	ENSG00000264443		23.51	-4.21	-3.52	0.000431283	0.01334945	10.5581152	0	0	0	0	0.83132898	0	0	143.749594	183.528607	0	0	10.3931939	8.80939988	7.37616561	10.93910419											
NPAS2	ENSG00000170485	4862	233.08	-3.68	-3.52	0.000435536	0.013458838	179.063137	271.526456	0	1.96776539	0	133.107066	1.27892657	0.3857046	2883.91223	65.3315569	17.2629862	115.902374	13.4465925	7.328169758	24.091417	14.75102731	MOP4|PASD	MIM:603347	2	2q11.2	neuronal PAS	protein-codir	NPAS2	neuronal PAS	O	neuronal PAS	20210418
RBM3	ENSG00000102317	5935	1385.71	-0.68	-3.52	0.000436232	0.013458838	1162.68291	1463.23763	668.009857	1290.95001	733.899802	945.61851	1043.75137	928.80562	3382.65571	1468.23111	1555.11147	2639.26512	976.08225	1287.498789	1534.79572	1090.719803	IS1-RNPL|RN	MIM:300027	X	Xp11.23	RNA binding	protein-codir	RBM3	RNA binding	O	RNA-binding	20210417
	ENSG00000277801		19.64	-3.07	-3.52	0.000437318	0.013470511	6.16781526	6.45790487	0	0	0	3.25193256	1.2982032	0	104.183584	164.741923	0.77894011	1.49800439	2.08820068	2.524814827	11.0907968	10.23784192											
CCL4	ENSG00000275302	6351	911.18	0.84	3.51	0.000446836	0.013741467	381.637169	605.91023	863.515629	876.495914	705.535316	1384.61726	1661.45057	2803.87198	458.101449	270.167491	360.697585	411.22873	769.385702	537.6993844	525.50944	1963.085055	ACT2|AT744.	MIM:182284	17	17q12	C-C motif che	protein-codir	CCL4	C-C motif che	O	C-C motif che	20210307
ID3	ENSG00000117318	3399	26.50	-2.94	-3.51	0.000449074	0.013788019	10.0803609	23.964937	41.0882395	1.9061671	0	13.6689679	0	2.58284736	37.2536825	28.0969799	16.4263328	41.2198905	48.6432097	71.26392473	55.0061675	32.72237569	HEIR-1|bHLH	MIM:600277	1	1p36.12	inhibitor of D	protein-codir	ID3	inhibitor of D	O	DNA-binding	20210322
AXIN2	ENSG00000168646	8313	65.88	-1.73	-3.51	0.000451397	0.013828662	50.3849487	49.6686168	89.1251148	7.37389554	1.94101642	34.0607073	26.8623369	14.7509478	277.54422	71.8681023	35.3069516	33.8582462	45.003843	170.8214097	89.1876366	56.35510844	AXIL|ODCRC	MIM:604025	17	17q24.1	axin 2	protein-codir	AXIN2	axin 2	O	axin-2|axin-li	20210410
URB2	ENSG00000135763	9816	141.42	-0.92	-3.51	0.000451851	0.013828662	157.325218	127.851224	40.6608376	83.7532818	62.0998122	136.5358	107.860723	78.6859176	201.494421	209.048376	202.872109	268.797698	113.264309	186.2833701	157.055903	129.0891501	KIAA0133|NE	HGNC:HGNC:	1	1q42.13	URB2 ribosor	protein-codir	URB2	URB2 ribosor	O	unhealthy rib	20210404
PLXNB2	ENSG00000196576	23654	40.63	-2.59	-3.51	0.000453955	0.013848531	34.2488395	7.49930088	127.73811	2.1204539	3.44656526	9.76592066	0	8.14034873	203.231949	15.8797657	8.29461189	18.0604504	49.4253324	89.67552652	12.2435593	60.31437407	MM1|Nbla0(	MIM:604293	22	22q13.33	plexin B2	protein-codir	PLXNB2	plexin B2	O	plexin-B2	20210418
	ENSG00000225345		6.43	-3.97	-3.51	0.000455104	0.01385993	0	0	0	0	0	4.94674151	0	2.06406655	3.7039444	4.72804438	14.8526701	15.7834307	18.7492451	31.39905004	2.44456929	4.217944873											
KMO	ENSG00000117009	8564	8.29	-3.69	-3.50	0.000458697	0.01385993	4.49562984	0	0	0	14.5818332	0	0	0.33644195	10.9544543	20.7688208	9.26660231	4.65567152	49.6505153	0.75946755	3.47037476	13.64902135	dJ317G22.1	MIM:603538	1	1q43	kynurenine 3	protein-codir	KMO	kynurenine 3	O	kynurenine 3	20210302
UXS1	ENSG00000115652	80146	270.05	-1.20	-3.50	0.00045775	0.01385993	299.719323	172.828844	50.2935423	122.149632	56.3252508	457.863324	210.547303	109.87159	188.375548	290.368202	378.362699	381.144589	223.98	549.9580663	472.857799	356.118237	SDR6E1|UGD	MIM:609749	2	2q12.2	UDP-glucuror	protein-codir	UXS1	UDP-glucuror	O	UDP-glucuror	20210327
RPS23	ENSG00000186468	6228	4342.51	-0.60	-3.50	0.00045838	0.01385993	5332.43707	5328.22654	3745.1949	3170.00363	1507.59615	3704.9602	2432.83177	2636.0927	9375.37473	4508.41124	4763.67342	7207.77819	2742.36272	4268.451581	4241.95547	4514.855136	BTDD|MABA	MIM:603683	5	5q14.2	ribosomal pr	protein-codir	RPS23	ribosomal pr	O	40S ribosoma	20210404
ARGLU1	ENSG00000134884	55082	1739.36	0.76	3.50	0.000458072	0.01385993	2546.47645	1825.07029	2426.98684	1993.71711	2705.94203	1981.28079	1878.41418	1756.91838	896.260617	1751.28463	1914.23817	1147.36473	1448.40119	1566.726024	1504.5066	486.1792156	-	MIM:614046	13	13q33.3	arginine and	protein-codir	ARGLU1	arginine and	O	arginine and	20210302
AGAP2-AS1	ENSG00000255737	100130776	7.62	-4.13	-3.50	0.000462551	0.013954242	0	1.69300321	0	0	0	0	11.1731727	0	21.4919889	32.3400576	1.46513987	0.82742341	18.0934298	7.350679543	17.2747818	10.14769491	PUNISHER	HGNC:HGNC:	12	12q14.1	AGAP2 antise	ncRNA	AGAP2-AS1	AGAP2 antise	O	-	20210316
TRAV12-3	ENSG00000211794	28672	20.44	-3.08	-3.50	0.000466924	0.01404164	24.9710935	31.3560163	0	0	0	3.92662461	3.09808444	2.96764814	19.0023079	13.1657883	105.116978	25.3515938	10.9404226	36.47726495	25.6821463	24.92342805	TCRAV12S3|	HGNC:HGNC:	14	14q11.2	T cell recepto	other	TRAV12-3	T cell recepto	O	-	20210302
	ENSG00000233175		24.80	-3.07	-3.50	0.00046629	0.01404164	1.1715809	3.79762965	0	9.06571411	13.4888171	0	6.55131154	6.50219971	129.140277	113.591548	8.56384442	2.39091001	29.2332986	24.68573581	15.4027108	33.15747259											
AFG3L2	ENSG00000141385	10939	313.36	-0.70	-3.50	0.000472313	0.014181301	212.21973	125.233073	295.261083	264.922923	317.478749	196.99884	289.59045	204.982451	650.144339	249.983782	301.223236	366.34721	407.255657	275.4227702	461.843813	394.9235723	OPA12|SCA2	MIM:604581	18	18p11.21	AFG3 like ma	protein-codir	AFG3L2	AFG3 like ma	O	AFG3-like prc	20210410
ADCK2	ENSG00000133597	90956	60.97	-1.46	-3.49	0.000477223	0.014306172	43.9622071	42.357571	10.4290115	54.0368013	5.11214883	32.8946276	21.4431625	49.244577	229.216013	66.5433623	45.093872	52.3925694	88.4388763	56.37184247	63.0721878	114.9164307	AARF	HGNC:HGNC:	7	7q34	aarF domain	protein-codir	ADCK2	aarF domain	O	uncharacteriz	20210302
	ENSG00000268509		4.17	-3.79	-3.49	0.000479816	0.014339776	0	0.5968381	0	1.40367861	0	0	0.73457925	0.69823919	15.6892661	15.8838378	3.85333154	6.09369201	5.5717821	6.188370796	4.08887621	5.912812123											
NFATC1	ENSG00000131196	4772	324.35	-0.61	-3.49	0.000479851	0.014339776	410.49537	364.230877	237.882463	141.179096	216.387063	292.703715	217.842789	219.378976	427.372852	395.054709	355.721331	325.635451	408.917346	455.689092	382.611165	338.5137772	NF-ATC|NF-A	MIM:600489	18	18q23	nuclear facto	protein-codir	NFATC1	nuclear facto	O	nuclear facto	20210329
RHPN1	ENSG00000158106	114822	31.05	-2.14	-3.49	0.000489416	0.014579852	45.2868992	14.1490072	0	3.52592948	0	30.0690154	10.7468817	14.3455607	70.2360266	59.9671216	28.9835082	20.4917284	41.913224	65.72635665	78.7122856	12.61170158	ODF5|RHOPI	MIM:601031	8	8q24.3	rhophilin Rhc	protein-codir	RHPN1	rhophilin Rhc	O	rhophilin-1|(	20210302
PITX1-AS1	ENSG00000224186	100996485	15.59	-3.69	-3.48	0.000495213	0.014660783	4.10401004	1.2157635	0	0	0	0	0.79954562	5.62057658	80.4226769	87.4199126	1.87327459	0	24.5090559	31.10156005	7.2595242	5.176894905	C5orf66	HGNC:HGNC:	5	5q31.1	PITX1 antiser	ncRNA	PITX1-AS1	PITX1 antiser	O	-	20210302
FOSB	ENSG00000125740	2354	45.30	-2.31	-3.48	0.000494437	0.014660783	67.9961245	29.3375183	0	6.52886329	0	0	25.6627011	7.82086038	383.173136	48.2428818	3.64950968	15.6198461	47.0842405	14.18926148	52.7337458	22.80754383	AP-1|G0S3|(	MIM:164772	19	19q13.32	FosB proto-o	protein-codir	FOSB	FosB proto-o	O	protein fosB|	20210302
TRAV19	ENSG00000211799	28664	55.96	-2.08	-3.48	0.000493711	0.014660783	43.5036334	113.547784	0	1.76681891	0	25.8821392	21.7529696	67.1338622	57.4926101	119.501943	44.1843995	76.3087473	15.7848241	86.72511799	71.0659694	150.6749509	TCRAV12S1|	HGNC:HGNC:	14	14q11.2	T cell recepto	other	TRAV19	T cell recepto	O	-	20210302
DPH5	ENSG00000117543	51611	120.45	-1.63	-3.48	0.000494672	0.014660783	112.283915	106.697701	0	33.240496	93.1725943	108.285159	42.7428343	51.3266839	150.158238	213.052036	106.347826	257.993366	88.9602625	173.1108329	200.855623	188.9918555	AD-018|CGI-	MIM:611075	1	1p21.2	diphthamide	protein-codir	DPH5	diphthamide	O	diphthine me	20210302
MPPED2	ENSG00000066382	744	8.54	-4.52	-3.48	0.000500197	0.014762412	0	0	20.1624431	0	0	2.10765726	0	0	47.9904029	1.59423701	7.09479751	5.89963807	11.0447571	9.142400843	17.5462012	14.11873449	239FB|C11or	MIM:600911	11	11p14.1	metallophos	protein-codir	MPPED2	metallophos	O	metallophos	20210302
ADGRE1	ENSG00000174837	2015	11.96	-3.70	-3.48	0.000506862	0.014903179	0	25.5469958	0	5.13509972	0	0	0.61864308	4.93298143	6.06435158	14.9785262	30.1741809	31.4977695	12.9243386	37.95651036	17.5093686	3.98458863	EMR1|TM7LI	MIM:600493	19	19p13.3-p13.	adhesion G p	protein-codir	ADGRE1	adhesion G p	O	adhesion G p	20210302
ERBB3	ENSG00000065361	2065	69.94	-1.43	-3.48	0.000507913	0.014903179	95.7342372	67.8250091	26.0179864	9.00951773	18.3240947	44.0367471	20.0727009	23.3102	343.494917	50.1598283	62.3063437	57.1752671	65.8920836	68.13253354	115.682303	51.86972454	ErbB-3|FERLI	MIM:190151	12	12q13.2	erb-b2 recep	protein-codir	ERBB3	erb-b2 recep	O	receptor tyro	20210302
TTLL3	ENSG00000214021	26140	307.58	0.72	3.48	0.00050724	0.014903179	277.945231	255.855886	408.477699	355.174703	595.976033	311.674773	461.074443	357.319048	106.145028	204.196825	259.128843	195.31011	356.363479	371.9192382	247.609139	157.1059094	HOTTL	MIM:619195	3	3p25.3	tubulin tyrosi	protein-codir	TTLL3	tubulin tyrosi	O	tubulin monc	20210306
SNORA53	ENSG00000212443	677832	361.75	1.58	3.48	0.000508099	0.014903179	2026.58527	1131.40855	282.708299	532.283774	17.1273841	155.051554	99.379918	170.041044	358.758873	369.094256	259.275071	238.645872	43.0078721	12.28881156	7.58553407	84.71306783	ACA53	HGNC:HGNC:	12	12q23.1	small nucleol	snoRNA	SNORA53	small nucleol	O	-	20210302
SMDT1	ENSG00000183172	91689	213.47	-0.87	-3.47	0.000511553	0.014976252	174.95688	140.791248	283.36778	103.865717	38.9821058	91.5589761	143.885366	215.507702	652.893569	246.034788	267.523145	229.467309	123.0604	115.3560087	209.532066	378.732854	C22orf32|DD	MIM:615588	22	22q13.2	single-pass m	protein-codir	SMDT1	single-pass m	O	essential MC	20210302
FGL2	ENSG00000127951	10875	203.84	1.29	3.47	0.000512163	0.014976252	93.9962814	146.57336	454.088693	266.69257	525.741635	119.515277	634.147295	112.509251	157.767306	107.378327	84.4525952	114.898571	259.438528	23.30143248	93.7585438	67.13559264	T49|pT49	MIM:605351	7	7q11.23	fibrinogen lik	protein-codir	FGL2	fibrinogen lik	O	fibroleukin|fi	20210406
MYB	ENSG00000118513	4602	56.81	-2.64	-3.47	0.000517154	0.015075883	112.272322	92.1448857	0	0	0	40.617179	26.7041913	4.2897359	156.365427	94.0951415	74.6597555	94.7726117	36.2894216	78.48005826	60.0170551	38.31626343	Cmyb|c-myb	MIM:189990	6	6q23.3	MYB proto-o	protein-codir	MYB	MYB proto-o	O	transcription	20210418
MATCAP2	ENSG00000164542	23366	29.94	-2.00	-3.47	0.000516756	0.015075883	61.1155519	6.4042157	17.6713186	3.69965517	0	6.20607623	3.91248939	3.86228304	226.570306	14.8431399	11.758386	43.0508523	8.7614904	13.67970891	18.8761361	38.58701923											
DUBR	ENSG00000243701	344595	90.56	1.83	3.47	0.000518269	0.015085259	128.907074	118.832569	912.998332	26.3024891	30.7560742	63.9217521	17.5655265	10.6933573	24.3009339	14.0214654	27.5798204	24.390369	15.1905148	8.293563868	17.5203476	7.729156764	DUM|LINC00	MIM:616619	3	3q13.12	DPPA2 upstre	ncRNA	DUBR	DPPA2 upstre	O	long intergen	20210302
	ENSG00000262714		18.21	-3.29	-3.47	0.00052057	0.015095676	25.6559756	16.0194915	0	0	0	17.154834	3.35467928	0	20.6506506	27.8729968	24.278874	52.6806272	26.1399278	43.53065774	14.1526553	19.88386351											
MICU3	ENSG00000155970	286097	37.30	-2.41	-3.47	0.000521005	0.015095676	96.7204576	11.9289099	14.5364207	0	0	45.0680199	11.7188047	2.64607375	29.6729722	71.2397953	38.4609775	53.5012225	14.0147041	120.9350811	50.5623405	35.76811626	EFHA2	MIM:610633	8	8p22	mitochondria	protein-codir	MICU3	mitochondria	O	calcium uptal	20210302
TPT1	ENSG00000133112	7178	16456.31	-0.68	-3.47	0.000519781	0.015095676	16271.1705	16674.1924	17498.0701	10632.2126	5983.41721	13489.0787	9131.36599	10830.4511	39213.2304	18289.9204	13985.9414	25858.2218	11513.731	17379.20133	17550.6204	19000.20171	HRF|TCTP|p(	MIM:600763	13	13q14.13	tumor protei	protein-codir	TPT1	tumor protei	O	translationall	20210302
TRIQK	ENSG00000205133	286144	34.13	-2.85	-3.47	0.000525911	0.015191556	20.8826215	13.511331	123.104067	3.60089679	0	0	0.36140732	1.39236884	265.572616	24.0628585	22.7861052	7.7771897	17.6227155	4.411802297	22.6999334	18.33310304	C8orf83|PRO	HGNC:HGNC:	8	8q22.1	triple QxxK/R	protein-codir	TRIQK	triple QxxK/R	O	triple QxxK/R	20210417
COX2	ENSG00000198712	4513	38210.95	0.72	3.47	0.000525442	0.015191556	86361.0464	85557.0097	73680.395	28615.5872	17046.0483	29990.0566	30651.3698	28488.7942	73788.2113	27579.2009	36084.1207	12515.0792	16365.7877	14176.12826	20372.1534	30104.18125	COII|MTCO2	MIM:516040	MT	-	cytochrome c	protein-codir	MT-CO2	mitochondria	O	cytochrome c	20210407
RASGEF1B	ENSG00000138670	153020	307.28	0.81	3.46	0.000530965	0.015314318	237.60632	137.509027	512.014213	318.99185	788.749284	310.574667	373.969739	408.653763	92.7676857	138.224779	139.649927	135.24844	535.760494	179.8319049	249.669169	357.3257902	GPIG4	MIM:614532	4	4q21.21	RasGEF doma	protein-codir	RASGEF1B	RasGEF doma	O	ras-GEF doma	20210417
LGALS9	ENSG00000168961	3965	81.71	-2.37	-3.46	0.000531779	0.015314604	54.4906479	7.8043241	0	25.9869141	0	1.30582398	5.35145253	19.358487	1027.26553	17.3412807	28.7657528	34.8781065	38.3862735	23.20427118	7.39347764	15.84957904	HUAT|LGALS	MIM:601879	17	17q11.2	galectin 9	protein-codir	LGALS9	galectin 9	O	galectin-9|ec	20210418
TFAP4	ENSG00000090447	7023	53.37	-1.25	-3.46	0.000532704	0.01531807	66.7625509	27.7141312	9.26509518	12.3734107	14.2779416	75.8929318	47.9378403	20.5763049	91.1267769	64.0110479	36.9360145	98.2273905	40.4577102	113.1462866	77.7805483	57.39818905	AP-4|bHLHc4	MIM:600743	16	16p13.3	transcription	protein-codir	TFAP4	transcription	O	transcription	20210302
FXYD7	ENSG00000221946	53822	18.94	-2.71	-3.46	0.000534527	0.015347307	18.4890156	43.2867977	0	13.1939988	0	1.98237164	1.72346134	1.72436971	16.5501903	40.2351794	39.1039398	22.0798969	16.2927282	35.30884541	34.097913	18.98289897	-	MIM:606684	19	19q13.12	FXYD domain	protein-codir	FXYD7	FXYD domain	O	FXYD domain	20210302
PIM2	ENSG00000102096	11040	359.52	-1.48	-3.46	0.000535567	0.015354004	485.285468	380.803922	15.8835829	170.752498	95.5474652	310.316068	201.888796	112.05691	578.745707	321.804314	608.962471	658.755214	310.291051	651.9199001	442.606421	406.6523409	-	MIM:300295	X	Xp11.23	Pim-2 proto-(	protein-codir	PIM2	Pim-2 proto-(	O	serine/threor	20210329
GIMAP5	ENSG00000196329	55340	1291.14	-0.81	-3.46	0.000539665	0.01542501	1398.60156	978.512246	1005.68386	797.795099	661.877344	1088.67519	784.310396	707.057348	964.85603	1129.02664	2237.40284	2684.11354	1566.64266	1844.495056	1333.64963	1475.524952	HIMAP3|IAN	MIM:608086	7	7q36.1	GTPase, IMAI	protein-codir	GIMAP5	GTPase, IMAI	O	GTPase IMAP	20210302
SLC40A1	ENSG00000138449	30061	143.29	-2.23	-3.46	0.000543596	0.015514075	103.099413	22.945761	123.370842	4.36151648	11.3009938	49.3653498	15.4622587	0	1586.93864	47.1177027	35.1175017	53.2276264	71.6734495	66.27988947	59.455091	42.93455302	FPN1|HFE4|I	MIM:604653	2	2q32.2	solute carrier	protein-codir	SLC40A1	solute carrier	O	solute carrier	20210418
SLC5A10	ENSG00000154025	125206	14.31	-4.08	-3.46	0.00054494	0.015514137	6.2321905	0	0	0	0	0	0	6.11874185	59.9358584	82.27342	0.49282099	0.42478356	8.73982364	22.92723911	26.4527831	15.40811726	SGLT-5|SGLT	MIM:618636	17	17p11.2	solute carrier	protein-codir	SLC5A10	solute carrier	O	sodium/gluc	20210302
	ENSG00000267598		17.94	-2.89	-3.46	0.000546043	0.015514137	13.4953994	3.71559687	0	0	0	0	8.19760426	3.59679294	63.5735357	138.728765	0.63629379	1.02351839	9.51987244	8.318070376	13.6999161	22.5847211											
ZNF662	ENSG00000182983	389114	29.09	-2.62	-3.46	0.000545335	0.015514137	70.592336	41.0959776	0	1.13233358	0	17.0808653	9.94003738	2.83325902	28.6435368	31.7163279	43.2295888	38.6725025	19.6097289	70.80041337	63.8177668	26.26515096	-	HGNC:HGNC:	3	3p22.1	zinc finger pr	protein-codir	ZNF662	zinc finger pr	O	zinc finger pr	20210302
LINC01215	ENSG00000271856	101929623	146.97	-1.92	-3.46	0.000547137	0.015522064	331.937238	250.588349	0	14.9908132	23.0103217	160.704038	49.2728943	16.0699608	202.101903	248.506559	151.430567	203.412654	97.0803858	267.9283508	246.937173	87.51919002	-	HGNC:HGNC:	3	3q13.12	long intergen	ncRNA	LINC01215	long intergen	O	-	20210302
KLHL21	ENSG00000162413	9903	459.95	-0.62	-3.45	0.00055379	0.015687424	277.867145	366.336346	324.756266	503.693278	245.512952	479.353565	323.760712	371.66733	320.727085	712.47981	543.399749	508.078574	647.225272	611.9924147	491.955673	630.4586573	-	MIM:616262	1	1p36.31	kelch like fan	protein-codir	KLHL21	kelch like fan	O	kelch-like prc	20210302
EIF4A1	ENSG00000161960	1973	4077.50	-1.08	-3.45	0.000555703	0.015718227	4823.29725	3071.5907	2028.30441	1950.49338	1122.18697	2436.28518	2132.88768	3141.13271	2260.55718	2871.04437	4485.55358	7803.74009	3644.50526	5473.605622	6990.1452	11004.65193	DDX2A|EIF-4	MIM:602641	17	17p13.1	eukaryotic tr	protein-codir	EIF4A1	eukaryotic tr	O	eukaryotic in	20210404
CENPE	ENSG00000138778	1062	90.81	1.43	3.45	0.000556672	0.015722271	302.985442	35.7131666	141.841872	291.058076	87.9303154	58.2741225	32.7233145	172.008614	32.335704	29.0282371	87.7039114	34.9090509	64.4550058	28.69017207	23.4565795	29.78190673	CENP-E|KIF1(	MIM:117143	4	4q24	centromere p	protein-codir	CENPE	centromere p	O	centromere-a	20210302
IRF2BP1	ENSG00000170604	26145	90.02	-0.78	-3.45	0.000561893	0.015804469	41.3282877	89.2910081	29.5766396	45.2593477	81.1150997	77.4754051	63.3513263	111.831637	123.00531	142.293874	66.6723313	64.180008	116.293315	139.92704	93.4716646	155.2946858	-	MIM:615331	19	19q13.32	interferon re	protein-codir	IRF2BP1	interferon re	O	interferon re	20210417
CDC27	ENSG00000004897	996	721.61	0.69	3.45	0.00056103	0.015804469	1033.32677	763.871011	1948.62576	887.537381	409.11867	827.92374	614.07002	835.991081	661.518674	612.29219	652.026492	447.149103	498.701514	423.9128927	352.963993	576.7709394	ANAPC3|APC	MIM:116946	17	17q21.32	cell division c	protein-codir	CDC27	cell division c	O	cell division c	20210329
PTPN7	ENSG00000143851	5778	384.12	0.71	3.45	0.000564002	0.015835321	309.346378	296.135446	484.18309	336.416199	375.594301	386.787577	761.761783	895.829375	128.550257	253.963695	291.87231	213.749779	419.12133	261.6559232	272.084148	458.9366478	BPTP-4|HEPT	MIM:176889	1	1q32.1	protein tyros	protein-codir	PTPN7	protein tyros	O	tyrosine-prot	20210417
ZNF772	ENSG00000197128	400720	66.31	-1.35	-3.45	0.00056537	0.015850357	50.8126529	125.533218	33.1005325	23.6618932	11.5852629	37.6190225	44.2598153	17.5923399	73.3575526	70.430273	114.355797	93.2950463	69.9926533	124.2583227	101.83106	69.34723468	-	HGNC:HGNC:	19	19q13.43	zinc finger pr	protein-codir	ZNF772	zinc finger pr	O	zinc finger pr	20210302
FAM186B	ENSG00000135436	84070	5.44	-5.05	-3.45	0.000568035	0.015876615	0	0	0	0	0	0	0	0	34.7024371	20.4403897	4.27132323	4.21047658	2.14263418	1.986410033	14.7575289	4.486450794	C12orf25	HGNC:HGNC:	12	12q13.12	family with s	protein-codir	FAM186B	family with s	O	protein FAM1	20210302
	ENSG00000272540		27.21	-2.52	-3.45	0.000569643	0.015876615	9.49743147	9.11193763	0	1.80258431	0	16.225838	6.02228394	5.41264149	143.452724	127.123559	3.61383416	0	20.9836724	31.24888192	15.0125523	45.8387724											
TBK1	ENSG00000183735	29110	639.44	0.63	3.45	0.000569423	0.015876615	350.72938	667.191752	1692.7604	608.612196	824.394193	744.026657	747.173064	742.530501	320.915986	557.422033	542.282392	378.837051	581.21758	481.9604087	417.793754	573.133979	FTDALS4|IIAE	MIM:604834	12	12q14.2	TANK binding	protein-codir	TBK1	TANK binding	O	serine/threor	20210418
	ENSG00000224614		5.74	-2.82	-3.44	0.000575574	0.016018473	0.51853314	1.62690764	0	1.38460479	0	2.23872769	2.54275414	1.76849388	10.5300907	9.36363999	4.33781097	1.27830068	9.27457034	7.686038818	19.3895023	19.85091246											
PTCSC1	ENSG00000287736	100302522	11.59	-2.98	-3.44	0.000577486	0.016048207	0	4.17858919	0	0	2.31357886	1.59480298	1.93853924	8.41264596	28.2767028	73.0590967	2.754004	1.93499847	20.969447	25.20784779	4.43304795	10.28920933	NCRNA00197	MIM:617440	8	8q24.22	papillary thyr	ncRNA	PTCSC1	papillary thyr	O	papillary thyr	20210302
PCID2	ENSG00000126226	55795	576.03	0.78	3.44	0.000584086	0.016207973	623.178784	339.62447	1018.74169	697.546112	1454.43429	339.981436	959.273079	500.321492	200.262059	416.789633	414.724006	361.768633	759.983007	332.574427	476.284304	320.9319257	F10	MIM:613713	13	13q34	PCI domain c	protein-codir	PCID2	PCI domain c	O	PCI domain-c	20210302
LZTS3	ENSG00000088899	9762	32.37	-1.81	-3.44	0.000586846	0.016237205	21.6410536	11.8200772	0	28.2070645	3.29325599	14.6658742	14.7619213	31.3249571	93.5180569	63.5126111	23.1264213	38.9479297	55.677031	37.20270525	54.6025733	25.64704791	PROSAPIP1	MIM:610484	20	20p13	leucine zippe	protein-codir	LZTS3	leucine zippe	O	leucine zippe	20210417
SYTL2	ENSG00000137501	54843	1546.21	0.95	3.44	0.00058609	0.016237205	1237.9332	1104.10631	1986.59734	4141.76527	2208.98475	2038.56664	1479.33572	1963.15445	2388.94523	864.659647	790.962973	1255.27626	884.692206	566.9671357	838.337146	989.1506135	CHR11SYT|E	MIM:612880	11	11q14.1	synaptotagm	protein-codir	SYTL2	synaptotagm	O	synaptotagm	20210417
SLC35A4	ENSG00000176087	113829	346.64	-0.69	-3.44	0.000589654	0.016291227	360.509442	322.417886	123.88004	280.730016	157.138743	350.01139	256.62908	329.505778	450.461368	419.319233	386.599623	444.70428	383.376841	376.0240249	402.363133	502.6444716	-	HGNC:HGNC:	5	5q31.3	solute carrier	protein-codir	SLC35A4	solute carrier	O	probable UDI	20210330
IFI16	ENSG00000163565	3428	2118.70	0.70	3.43	0.000593217	0.016365921	2222.3919	2975.22644	2809.61131	2104.01657	2889.67495	2333.52203	2581.61516	2482.44315	3671.78775	1517.08777	1685.78559	1175.62061	1439.95519	1358.887235	1149.22343	1502.382506	IFNGIP1|PYH	MIM:147586	1	1q23.1	interferon ga	protein-codir	IFI16	interferon ga	O	gamma-inter	20210418
SLC3A1	ENSG00000138079	6519	18.60	-3.21	-3.43	0.00059646	0.016406809	5.27980916	14.2556283	0	0	0	26.0179627	0	2.73813429	88.9153705	111.385175	8.53435962	9.72857916	11.181689	6.771121322	5.66685295	7.084879417	ATR1|CSNU1	MIM:104614	2	2p21	solute carrier	protein-codir	SLC3A1	solute carrier	O	neutral and b	20210302
PCYT1A	ENSG00000161217	5130	512.32	0.92	3.43	0.00059626	0.016406809	208.872772	246.190993	1845.32998	870.470711	647.743846	523.932643	657.42123	609.949605	112.404331	349.719574	385.520483	336.670069	438.832937	297.439938	242.773378	423.9091639	CCTA|CCTalp	MIM:123695	3	3q29	phosphate cy	protein-codir	PCYT1A	phosphate cy	O	choline-phos	20210307
GBF1	ENSG00000107862	8729	1229.85	1.11	3.43	0.000608743	0.016649474	4915.01218	1144.81369	4591.97818	650.121792	678.827842	847.52393	867.551045	1068.16322	1099.47989	493.096073	427.225302	478.011353	622.903821	550.8147498	654.468219	587.6174854	ARF1GEF	MIM:603698	10	10q24.32	golgi brefeldi	protein-codir	GBF1	golgi brefeldi	O	Golgi-specific	20210417
CEBPB	ENSG00000172216	1051	133.42	-1.06	-3.43	0.000613053	0.016719303	94.9943288	81.8751635	45.6257528	52.13584	44.2690648	140.092518	97.3694663	95.334017	453.616807	272.512826	85.1839824	95.9496773	176.08956	152.2650917	125.363117	122.0251684	C/EBP-beta|I	MIM:189965	20	20q13.13	CCAAT enhar	protein-codir	CEBPB	CCAAT enhar	O	CCAAT/enhar	20210302
	ENSG00000280027		14.22	-3.67	-3.43	0.000614631	0.01673835	21.1460247	28.7114049	0	0	0	10.7358648	0	0	36.8937138	21.8621791	19.5964683	36.375006	19.2809937	19.12301213	4.97595612	8.779653398											

ANPEP	ENSG00000166825	290	21.67	-2.69	-3.42	0.000617901	0.016803386	7.81989457	3.39975127	18.9026434	6.78045069	8.82527994	0	0	0.54342363	91.9095802	4.02224648	14.1819406	14.4933263	150.927566	11.79215605	2.04589077	11.05489504	APN|CD13|G	MIM:151530	15	15q26.1	alanyl amino	protein-codir	ANPEP	alanyl amino	O		aminopeptid	20210316
SIRPB1	ENSG00000101307	10326	104.59	-2.79	-3.42	0.000621648	0.016881145	74.5034069	20.9777534	0	0	0	2.18415829	1.09807426	4.75141176	1477.45551	2.53421096	17.8245183	37.4396184	10.708141	6.274952304	6.02934031	11.59893957	CD172b|SIRP	MIM:603889	20	20p13	signal regulat	protein-codir	SIRPB1	signal regulat	O		signal-regulat	20210302
CSGALNACT1	ENSG00000147408	55790	181.07	-1.57	-3.42	0.000624804	0.016942701	89.3047227	176.046068	3.97413195	10.6182013	143.449602	190.738247	160.717063	106.018703	244.629831	237.169758	166.272141	214.727928	316.08474	438.0837739	269.538943	129.6804052	CSGalNAcT-1	MIM:616615	8	8p21.3	chondroitin s	protein-codir	CSGALNACT1	chondroitin s	O		chondroitin s	20210302
SCARNA13	ENSG00000252481	677768	608.31	-1.17	-3.42	0.000627364	0.016987917	690.705189	458.273825	343.743916	975.690963	54.3535643	90.4115399	128.84099	149.797231	343.114639	423.787929	1782.3404	3110.50081	264.656032	233.7009236	310.435584	372.6000603	U93	HGNC:HGNC:	14	14q32.13	small Cajal bo	ncRNA	SCARNA13	small Cajal bo	O		-	20210302
	ENSG00000268107		9.99	-3.87	-3.42	0.000629576	0.016999454	0	17.4001864	0	0	10.6593928	0	0	4.87923827	14.0743797	23.8398469	19.2637092	7.30472492	11.6592898	15.40635833	24.1912287	11.08662836												
KANK1	ENSG00000107104	23189	76.56	-1.51	-3.42	0.000632257	0.017047666	143.123822	49.9985557	67.3599102	8.38883255	17.2885212	87.5310144	12.9992034	19.8996786	138.389683	114.665924	73.0571795	86.950844	79.3050343	135.3729067	112.963894	77.69273949	ANKRD15|CP	MIM:607704	9	9p24.3	KN motif and	protein-codir	KANK1	KN motif and	O		KN motif and	20210302
ICAM3	ENSG00000076662	3385	300.81	-0.74	-3.42	0.000636014	0.017124702	227.880293	241.293563	160.984832	225.70718	261.368398	134.097746	219.120199	343.81623	504.823495	360.471972	233.112877	438.014996	306.856858	462.5354554	300.748873	392.147459	CD50|CDW5(	MIM:146631	19	19p13.2	intercellular a	protein-codir	ICAM3	intercellular a	O		intercellular a	20210302
ARHGEF11	ENSG00000132694	9826	122.81	-2.50	-3.41	0.000640983	0.017234109	101.14368	110.674862	12.5419054	2.53427166	0	94.5597626	0	15.741121	1294.81791	61.9657592	48.305281	39.2380184	28.3826868	66.09149469	63.0279145	25.87356932	GTRAP48|PD	MIM:605708	1	1q23.1	Rho guanine	protein-codir	ARHGEF11	Rho guanine	O		rho guanine r	20210418
FRY	ENSG00000073910	10129	296.48	1.21	3.41	0.000648431	0.017385249	541.87889	115.317809	378.443974	149.680874	576.793669	379.096392	636.314835	464.342211	123.612753	92.9082813	112.157081	111.585044	567.633798	207.9006738	55.7941007	230.2673444	13CDNA73|2	MIM:614818	13	13q13.1	FRY microtub	protein-codir	FRY	FRY microtub	O		protein furry	20210302
CD7	ENSG00000173762	924	145.62	-0.92	-3.41	0.000649356	0.01738556	170.073627	217.291604	50.3609543	73.4508337	39.4085424	133.594189	77.6354175	83.5328672	206.67009	275.323258	102.783763	109.40319	187.421415	167.9285746	233.880732	201.1858738	GP40|LEU-9|	MIM:186820	17	17q25.3	CD7 molecule	protein-codir	CD7	CD7 molecule	O		T-cell antigen	20210302
SNORD104	ENSG00000199753	692227	145.57	-1.60	-3.41	0.000653162	0.017462914	475.01041	448.239935	0	8.48183781	5.37602755	10.4569541	2.30812318	9.33575845	564.420098	632.015295	37.2624456	21.2232213	9.31325935	19.05116348	26.6232511	59.98645764	U104	HGNC:HGNC:	17	17q23.3	small nucleol	snoRNA	SNORD104	small nucleol	O		-	20210302
UBASH3B	ENSG00000154127	84959	228.11	-1.89	-3.41	0.00065456	0.017475732	350.765165	298.6093	9.76634322	48.1928839	6.73498655	515.476728	105.671811	30.4699482	382.999354	200.969058	226.420027	421.341801	193.747171	362.2491416	275.523054	220.8103831	STS-1|STS1|T	MIM:609201	11	11q24.1	ubiquitin asso	protein-codir	UBASH3B	ubiquitin asso	O		ubiquitin-asso	20210404
IGHM	ENSG00000211899	3507	20.97	-2.83	-3.40	0.000667483	0.017696664	0	7.36763057	2.60916469	1.72302577	0	2.69009235	3.66957039	2.13168727	7.57207929	8.21754838	4.9564475	14.4259529	228.970098	8.799455998	21.5296913	20.78925623	AGM1|MU|\	MIM:147020	14	14q32.33	immunoglobu	other	IGHM	immunoglobu	O		constant regi	20210302
SNORD116-8	ENSG00000207093	100033420	65.54	-2.11	-3.40	0.000666321	0.017696664	161.63868	169.316921	6.89158987	13.2089662	0	5.96650847	5.51870808	6.71007771	122.651866	98.1205686	146.19792	224.198822	14.1315804	33.70667938	25.4393117	14.9893227	HBII-85-8	HGNC:HGNC:	15	15q11.2	small nucleol	snoRNA	SNORD116-8	small nucleol	O		-	20210302
PDE4B	ENSG00000184588	5142	470.23	-1.24	-3.40	0.000665455	0.017696664	296.494376	282.949834	123.128723	124.943452	792.634236	684.165429	88.69084	143.997144	1682.0508	363.074492	474.591669	527.070967	673.72454	441.7623189	442.98175	381.3845313	DPDE4|PDEI\	MIM:600127	1	1p31.3	phosphodiest	protein-codir	PDE4B	phosphodiest	O		cAMP-specifi	20210302
ZCCHC14	ENSG00000140948	23174	271.04	-0.69	-3.40	0.000665579	0.017696664	154.877252	213.599599	82.7156957	180.805483	208.879421	247.35331	402.400991	188.760873	177.840749	222.664327	259.637343	378.095749	347.680737	420.9110272	520.804195	329.6611196	BDG-29|BDG	HGNC:HGNC:	16	16q24.2	zinc finger CC	protein-codir	ZCCHC14	zinc finger CC	O		zinc finger CC	20210302
ZFP90	ENSG00000184939	146198	677.79	0.90	3.40	0.000674015	0.017845005	2023.06405	595.820922	1506.19164	978.419153	578.370633	418.77221	585.908345	704.357089	388.063711	353.934701	464.247206	399.687743	487.186535	397.8880979	401.160039	561.5688815	FIK|NK10|ZN	MIM:609451	16	16q22.1	ZFP90 zinc fir	protein-codir	ZFP90	ZFP90 zinc fir	O		zinc finger pr	20210302
TRIM35	ENSG00000104228	23087	238.44	-0.76	-3.40	0.000680669	0.017996124	264.516648	128.894739	167.86197	223.61261	158.754375	126.055212	105.377165	205.656478	441.534584	516.445187	209.252722	338.606634	270.081733	231.1744395	122.57844	304.5635059	HLS5|MAIR	MIM:617007	8	8p21.2	tripartite mot	protein-codir	TRIM35	tripartite mot	O		E3 ubiquitin-	20210406
CCR9	ENSG00000173585	10803	10.85	-3.88	-3.40	0.000682713	0.018025148	31.82818	12.5157967	2.69986084	0	0	0	0	0	14.0711285	43.171793	13.641307	12.2926156	4.5727758	5.419958947	19.9889328	13.43203304	CC-CKR-9|CD	MIM:604738	3	3p21.31	C-C motif che	protein-codir	CCR9	C-C motif che	O		C-C chemokir	20210316
GZMA	ENSG00000145649	3001	523.43	0.62	3.39	0.000688178	0.018144265	458.035737	535.469236	439.535745	520.214276	414.939954	523.142138	840.343528	1287.17881	317.567083	351.495912	451.443537	295.06406	358.497334	193.7296295	438.849526	949.3670621	CTLA3|HFSP	MIM:140050	5	5q11.2	granzyme A	protein-codir	GZMA	granzyme A	O		granzyme A|	20210302
EMILIN2	ENSG00000132205	84034	25.16	-2.34	-3.39	0.000692886	0.018243128	11.7427992	21.8202011	1.65877145	0	10.4201077	0	21.3166063	19.7097461	69.7653245	84.5608962	8.16304205	10.850266	58.9227316	24.64861479	47.5601018	11.36756809	EMILIN-2|FO	MIM:608928	18	18p11.32-p11	elastin micro	protein-codir	EMILIN2	elastin micro	O		EMILIN-2|ela	20210302
TOX	ENSG00000198846	9760	394.24	0.67	3.39	0.000696496	0.018312846	180.446159	265.039053	763.633609	784.613441	634.433662	286.770718	559.541043	395.286695	108.566104	220.476129	430.025327	444.469835	418.226514	158.1093239	203.62604	454.6295893	TOX1	MIM:606863	8	8q12.1	thymocyte se	protein-codir	TOX	thymocyte se	O		thymocyte se	20210302
MTCO1P12	ENSG00000237973	107075141	688.61	0.75	3.39	0.000702521	0.018445772	1477.95278	1795.4569	686.405266	571.801596	666.155984	549.323197	636.674328	707.913664	726.142208	414.07393	417.254395	335.160008	480.296576	365.9120528	523.250331	663.9739018	-	HGNC:HGNC:	1	1p36.33	MT-CO1 pseu	pseudo	MTCO1P12	MT-CO1 pseu	O		mitochondria	20210302
ND3	ENSG00000198840	4537	10187.61	0.80	3.39	0.000704566	0.018474001	24919.2175	23156.6022	28823.1549	9067.5832	3317.52181	7845.10862	5512.98441	6104.7335	9817.39121	7761.34071	14026.5804	3475.88139	3542.16637	3463.56789	4933.97794	7233.934491	MTND3	MIM:516002	MT	-	NADH dehyd	protein-codir	MT-ND3	mitochondria	O		NADH dehyd	20210302
SNORD105	ENSG00000209645	692229	85.42	-2.39	-3.38	0.000712681	0.018661078	154.391126	171.146278	0	9.19351852	0	32.7549003	26.5094975	40.3718001	82.2502302	104.2302	151.869747	98.5933799	93.5407843	124.8464269	120.925045	156.0420331	U105	HGNC:HGNC:	19	19p13.2	small nucleol	snoRNA	SNORD105	small nucleol	O		-	20210302
NHSL2	ENSG00000204131	340527	1573.43	0.80	3.38	0.00071763	0.018764846	809.864286	1789.45468	787.179624	1964.92284	2698.01943	1948.61826	3611.39687	2551.7101	734.404405	1288.26862	600.682083	993.149767	1993.14682	790.9937141	721.639	1891.507178	-	HGNC:HGNC:	X	Xq13.1	NHS like 2	protein-codir	NHSL2	NHS like 2	O		NHS-like prot	20210302
ST8SIA4	ENSG00000113532	7903	872.08	0.71	3.38	0.000727065	0.018959462	350.954859	552.97604	2584.22226	1430.05125	1233.2237	922.61067	956.128657	1024.95285	355.538244	581.218473	793.162259	641.051506	728.758705	513.3815287	527.450905	757.5794293	PST|PST1|SI/	MIM:602547	5	5q21.1	ST8 alpha-N-a	protein-codir	ST8SIA4	ST8 alpha-N-a	O		CMP-N-acety	20210302
MYSM1	ENSG00000162601	114803	1142.80	0.72	3.38	0.000731729	0.019028949	1674.86474	945.522475	1660.66502	1808.67902	2414.47826	1010.62822	775.548885	1081.62911	1734.6927	728.686889	1180.32806	802.296351	616.594036	625.1737336	584.432829	640.604492	2A-DUB|2AD	MIM:612176	1	1p32.1	Myb like, SW	protein-codir	MYSM1	Myb like, SW	O		deubiquitinas	20210302
	ENSG00000259661		8.89	-2.91	-3.37	0.000747669	0.019416951	9.14764492	1.44860663	0	0	0	0	1.93610492	1.18964383	71.7000702	33.5259926	3.767742	1.37845476	2.34353111	4.321093109	6.41997785	4.982136312												
ACER3	ENSG00000078124	55331	242.40	0.71	3.37	0.000749542	0.019439068	308.455893	338.384793	127.842674	212.543631	335.540109	380.588762	407.081593	323.649762	112.786444	223.661163	147.378084	169.31108	216.103534	151.6613745	236.005543	187.4412768	APHC|PHCA|	MIM:617036	11	11q13.5	alkaline cerar	protein-codir	ACER3	alkaline cerar	O		alkaline cerar	20210403
PVT1	ENSG00000249859	5820	204.93	-2.11	-3.36	0.000769326	0.01984402	549.165564	196.900544	0	10.7109523	61.0124107	263.611814	64.8840719	23.1613879	142.662704	180.674506	197.75135	257.605734	287.715388	525.8856311	359.140061	157.9562969	LINC00079|N	MIM:165140	8	8q24.21	Pvt1 oncoger	ncRNA	PVT1	Pvt1 oncoger	O		CXCR4/PVT1	20210418
LINC01128	ENSG00000228794	643837	223.33	-0.96	-3.36	0.000772599	0.019874597	219.260569	177.467201	17.5949591	238.801808	74.9361855	263.811135	181.360533	117.275132	375.153093	210.323313	90.5704831	191.012788	271.934136	422.5348744	437.714814	283.5793592	-	HGNC:HGNC:	1	1p36.33	long intergen	ncRNA	LINC01128	long intergen	O		-	20210302
TRABD2A	ENSG00000186854	129293	211.16	-1.95	-3.36	0.000777999	0.019986494	405.876296	216.29646	0	56.6515041	43.973611	163.559871	17.4120241	135.255757	263.896969	258.672795	226.469126	594.561083	203.699185	305.285875	262.684724	224.2994453	C2orf89|TIKI	MIM:614912	2	2p11.2	TraB domain	protein-codir	TRABD2A	TraB domain	O		metalloprote	20210302
RGL1	ENSG00000143344	23179	60.81	-1.84	-3.36	0.000783227	0.020066626	45.8874047	63.42107	3.24384797	16.9385582	23.4726353	4.14186215	3.32427237	7.97142488	582.454117	19.0922481	27.9948862	48.095964	51.6580086	35.56321372	14.204734	25.49024212	RGL	MIM:605667	1	1q25.3	ral guanine n	protein-codir	RGL1	ral guanine n	O		ral guanine n	20210302
SNORD116-2	ENSG00000207375	100033434	14.35	-3.23	-3.36	0.000789538	0.020088103	26.7025492	31.9841106	0	0	0	0	0	2.83519724	41.3662469	51.9751456	14.8015731	21.9375502	7.18737315	1.447969523	17.6602867	11.63040391	HBII-85-23	HGNC:HGNC:	15	15q11.2	small nucleol	snoRNA	SNORD116-2	small nucleol	O		-	20210302
ZDHHC11B	ENSG00000206077	653082	27.76	-1.70	-3.36	0.00078871	0.020088103	6.63177079	36.0916255	0	24.6438496	0	19.9236278	13.5961001	11.5492173	18.6345027	93.5322503	15.2818536	43.135503	14.8507704	74.29614325	59.502984	12.42997135	DHHC-11B	HGNC:HGNC:	5	5p15.33	zinc finger DH	protein-codir	ZDHHC11B	zinc finger DH	O		probable pal	20210302
PRKCA	ENSG00000154229	5578	838.60	-1.19	-3.36	0.000790508	0.020088103	1968.24089	808.00174	633.299834	201.192336	112.840491	972.774223	405.975355	137.253651	1037.99847	960.048801	1011.09695	1190.90612	598.271403	1542.463042	1145.61631	691.6668467	AAG6|PKC-al	MIM:176960	17	17q24.2	protein kinas	protein-codir	PRKCA	protein kinas	O		protein kinas	20210418
NDUFB11	ENSG00000147123	54539	177.83	-0.71	-3.36	0.000790045	0.020088103	194.515638	195.373906	83.9817889	138.356474	93.6968329	143.459294	114.690737	121.514854	248.179082	182.835858	199.913042	339.881502	160.284286	199.3798225	205.998639	223.1874486	CI-ESSS|ESSS	MIM:300403	X	Xp11.3	NADH:ubiqui	protein-codir	NDUFB11	NADH:ubiqui	O		NADH dehyd	20210410
MAP3K1	ENSG00000095015	4214	1338.76	-0.58	-3.36	0.0007914	0.020088103	1672.92888	1595.30156	515.707962	1179.99934	836.624013	1091.52135	1040.75915	824.780232	2184.18778	1485.67289	1406.86858	1555.02587	1204.81244	2129.680802	1388.94749	1307.404178	MAPKKK1|M	MIM:600982	5	5q11.2	mitogen-activ	protein-codir	MAP3K1	mitogen-activ	O		mitogen-activ	20210410
RELCH	ENSG00000134444	57614	1002.01	0.66	3.36	0.000791452	0.020088103	521.670471	909.259697	2823.11393	1069.28533	1795.09072	950.564964	1041.23096	998.644203	308.531517	692.07889	848.487457	910.442523	834.881641	929.3172974	775.727205	623.8852284	HsT3308|HsT	MIM:618001	18	18q21.33	RAB11 bindin	protein-codir	RELCH	RAB11 bindin	O		RAB11-bindir	20210302
RBM5-AS1	ENSG00000281691	100775107	10.79	-4.25	-3.35	0.00079429	0.02010567	0	1.64256376	0	0	0	0	0	0	53.9981813	97.849637	0.73902363	0.6757007	1.71225332	11.03316647	4.19126222	0.805851196	LUST	HGNC:HGNC:	3	3p21.31	RBM5 antiser	ncRNA	RBM5-AS1	RBM5 antiser	O		LUCA-15-spe	20210302
SMAD5	ENSG00000113658	4090	638.16	0.61	3.35	0.000793688	0.02010567	948.618519	730.47944	671.797749	606.669346	844.364765	729.253686	730.232429	859.185644	311.482371	495.65929	510.548156	498.357417	593.374339	522.0626578	379.322608	779.1737481	DWFC|JV5-1	MIM:603110	5	5q31.1	SMAD family	protein-codir	SMAD5	SMAD family	O		mothers agai	20210322
PIGG	ENSG00000174227	54872	366.89	0.69	3.35	0.000795313	0.02010567	261.195174	341.527834	1169.55565	384.983992	303.075246	524.195998	522.996186	254.043206	178.261565	237.603495	326.472801	236.939911	322.785169	316.1126416	331.296918	159.1367921	GPI7|LAS21|	MIM:616918	4	4p16.3	phosphatidyl	protein-codir	PIGG	phosphatidyl	O		GPI ethanola	20210302
	ENSG00000281490		20.50	-2.88	-3.35	0.000800447	0.020181864	0.66407622	14.5886588	0	0	0	0.85260216	19.3531221	20.405387	66.3229268	102.169261	2.49227915	1.0761642	34.5301128	16.8429908	25.7535593	22.90184976												
ZSCAN18	ENSG00000121413	65982	83.32	-1.30	-3.35	0.000805455	0.020254488	71.8530876	44.5662992	12.0253142	27.7339089	15.2356521	149.566969	90.5206226	24.5974089	129.958666	77.98483	83.2839237	68.3203215	148.65104	110.4853323	201.58842	76.70721885	ZNF447	HGNC:HGNC:	19	19q13.43	zinc finger an	protein-codir	ZSCAN18	zinc finger an	O		zinc finger an	20210302
BCAS4	ENSG00000124243	55653	60.83	-1.75	-3.35	0.000811799	0.020306703	130.542594	53.7797111	0	11.595857	1.90928227	67.210034	26.5366708	19.2252692	149.296865	176.930063	31.3565709	45.5893836	68.5929438	52.27387907	77.0525672	61.31091675	CNOL	MIM:607471	20	20q13.13	breast carcin	protein-codir	BCAS4	breast carcin	O		breast carcin	20210302
ACSL6	ENSG00000164398	23305	211.73	-1.34	-3.35	0.000811395	0.020306703	184.021453	120.128752	318.855985	32.7887879	81.5114475	151.198464	39.3811976	160.864333	277.109583	260.248248	209.724572	277.800587	182.695751	328.3801194	435.824504	327.1276394	ACS2|FACL6|	MIM:604443	5	5q31.1	acyl-CoA synt	protein-codir	ACSL6	acyl-CoA synt	O		long-chain-fa	20210302
PLEKHG3	ENSG00000126822	26030	335.60	0.80	3.35	0.000813619	0.020325523	197.071055	504.930307	122.525875	435.217464	1074.13351	403.458412	233.744226	510.616481	281.329744	246.805391	56.8835733	231.68309	393.438693	152.5594126	196.132005	329.1221383	ARHGEF43|K	MIM:617940	14	14q23.3	pleckstrin ho	protein-codir	PLEKHG3	pleckstrin ho	O		pleckstrin ho	20210302
BACH2	ENSG00000112182	60468	421.21	-1.69	-3.35	0.000815163	0.020337399	399.540674	250.93216	0	168.962846	97.7244804	991.56177	161.87499	138.339353	392.004976	497.125235	324.116728	697.513429	457.226699	825.3997323	1000.31522	336.7303554	BTBD25|IMD	MIM:605394	6	6q15	BTB domain a	protein-codir	BACH2	BTB domain a	O		transcription	20210417
KCTD12	ENSG00000178695	115207	66.61	-2.53	-3.35	0.000817118	0.020359489	67.6474896	34.2511455	85.2756214	11.4330475	21.7145805	33.4478775	0	0	307.912091	34.105666	38.6654821	144.811237	98.1994951	102.3758543	56.0988079	29.741622	C13orf2|PFE	MIM:610521	13	13q22.3	potassium ch	protein-codir	KCTD12	potassium ch	O		BTB/POZ don	20210302
RGS10	ENSG00000148908	6001	368.51	-0.87	-3.35	0.000819195	0.020384579	513.183284	382.888369	147.345874	197.189086	119.04597	388.242266	267.884633	187.419322	383.162593	437.058863	349.772855	654.840058	287.25488	533.9188721	507.09963	539.8436108	-	MIM:602856	10	10q26.11	regulator of (	protein-codir	RGS10	regulator of (	O		regulator of (	20210302
SERPINB2	ENSG00000197632	5055	37.81	-3.59	-3.34	0.000835362	0.020732649	44.7761035	44.5762629	0	0	0	0	0	0	439.598595	11.8967568	11.9802565	29.6310953	14.9451056	1.583632303	0	5.936062417	HsT1201|PAI	MIM:173390	18	18q21.33-q2	serpin family	protein-codir	SERPINB2	serpin family	O		plasminogen	20210302
ZNF629	ENSG00000102870	23361	26.35	-1.66	-3.34	0.000837552	0.020756683	15.2814009	48.7142408	14.2963477	4.85340114	13.8487263	8.42232007	5.03372373	7.85334091	68.2009536	40.9285707	37.0587325	26.6023659	13.9133925	37.84626451	43.9941274	34.80268764	ZNF|ZNF65	HGNC:HGNC:	16	16p11.2	zinc finger pr	protein-codir	ZNF629	zinc finger pr	O		zinc finger pr	20210302
G2E3	ENSG00000092140	55632	555.11	0.61	3.34	0.000838511	0.020756683	498.154285	735.644362	1179.41442	674.138405	983.874421	437.796536	379.320877	596.361307	405.043708	509.152992	491.053135	511.21091	411.005481	368.2280646	357.642757	343.6717569	KIAA1333|PH	MIM:611299	14	14q12	G2/M-phase	protein-codir	G2E3	G2/M-phase	O		G2/M phase-	20210302
CYBB	ENSG00000165168	1536	110.55	-2.02	-3.33	0.000853638	0.021103715	80.7080055	26.3647628	135.263643	29.9156412	72.0362176	1.63610104	0	1.80709851	361.341809	16.2240075	59.1478917	67.7817323	872.397395	17.34452754	9.15338019	17.67844716	AMCBX2|CGI	MIM:300481	X	Xp21.1-p11.4	cytochrome b	protein-codir	CYBB	cytochrome b	O		cytochrome b	20210415
SERHL	ENSG00000172250	94009	15.93	-3.42	-3.33	0.000857245	0.021153157	1.48751202	1.09608802	0	0	0	18.3060511	0	6.47253385	90.9798847	54.9761544	0.63645539	0.62926713	10.471379	19.062208	31.8311287	18.91590739	BK126B4.1|B	MIM:607979	22	22q13.2	serine hydrol	pseudo	SERHL	serine hydrol	O		kraken-like|s	20210302
INTS5	ENSG00000185085	80789	125.85	-0.79	-3.33	0.000857861	0.021153157	90.9142272	113.635868	59.0985442	44.2356279	93.5252216	78.8011505	131.81515	146.200581	138.001409	150.885947	121.822273	169.528254	145.442017	161.6669188	170.017238	197.9854269	INT5|KIAA16	MIM:611349	11	11q12.3	integrator co	protein-codir	INTS5	integrator co	O		integrator co	20210302
FBLN7	ENSG00000144152	129804	8.75	-2.96	-3.33	0.000864283	0.021283942	9.12266114	12.4538802	0	3.64319342	1.96902884	0	0	0.25635883	10.8268105	28.0854073	7.80096751	10.6668275	39.794551	5.757922669	3.9943799	5.590789731	TM14	MIM:611551	2	2q13-q14.1	fibulin 7	protein-codir	FBLN7	fibulin 7	O		fibulin-7|FIBL	20210302
FHIP1A	ENSG00000164142	729830	51.50	-2.86	-3.33	0.000873752	0.021489331	22.9369038	28.4505054	109.46168	10.8511767	0	3.46439618	0	1.09783755	445.284847	80.644363	6.62083616	16.3215286	13.8568736	22.00625423	38.9466273	24.07227849	FAM160A1|F	HGNC:HGNC:	4	4q31.3	FHF complex	protein-codir	FHIP1A	FHF complex	O		protein FAM1	20210302
SDR42E1	ENSG00000184860	93517	105.19	-1.20	-3.33	0.000875816	0.021512294	64.0826894	119.305647	63.837177	63.739763	20.0378389	143.14849	33.2996509	24.8588957	294.766467	106.013872	87.1680906	235.622474	52.9061458	137.4857355	167.654029	69.07661691	HSPC105	MIM:616164	16	16q23.3	short chain d	protein-codir	SDR42E1	short chain d	O		short-chain d	20210302
TNFRSF10D	ENSG00000173530	8793	25.15	-2.33	-3.32	0.000885259	0.021716232	41.3899491	30.2439045	0	0	21.5632109	1.64928457	10.1427476	4.33154178	55.7410038	39.0360327	28.5376609	37.6786879	22.4513381	31.45532083	53.3752885	24.76197505	CD264|DCR2	MIM:603614	8	8p21.3	TNF receptor	protein-codir	TNFRSF10D	TNF receptor	O		tumor necros	20210302
S1PR4	ENSG00000125910	8698	281.34	-0.81	-3.32	0.000889271	0.021786578	181.952789	160.161336	340.787568	121.665431	182.979926	165.957274	263.158958	236.080691	338.339335	469.13675	220.228462	213.04572	404.927794	398.4315609	390.26191	414.2694413	EDG6|LPC1|S	MIM:603751	19	19p13.3	sphingosine-1	protein-codir	S1PR4	sphingosine-1	O		sphingosine 1	20210302
FAM222A	ENSG00000139438	84915	8.95	-3.84	-3.32	0.000903372	0.022086204	5.43953058	9.92044777	0	9.18178202	0	0	0	0	44.1112114	6.5665484	9.7005106	7.11225529	18.2454313	6.956493016	5.9428311	20.09270079	C12orf34	HGNC:HGNC:	12	12q24.11	family with se	protein-codir	FAM222A	family with se	O		protein FAM2	20210302
	ENSG00000262292		8.17	-3.29	-3.32	0.000904984	0.022086204	3.91834155	7.35875432	0	0	0	0	2.0941985	3.20033952	4.3481666	7.94019433	10.1928479	25.5671686	18.4700976	7.168113443	23.0227923	17.47209146												
ANKRD16	ENSG00000134461	54522	46.80	-1.65	-3.32	0.000906142	0.022086204	31.4804043	15.4336728	0	6.50549077	23.968364	14.1493345	8.483261	19.577305	432.797538	31.1540787	17.1259013	22.4423483	34.6406682	41.89874295	16.0771029	33.0280406	-	MIM:618017	10	10p15.1	ankyrin repea	protein-codir	ANKRD16	ankyrin repea	O		ankyrin repea	20210302
RPS15	ENSG00000115268	6209	1636.32	-0.74	-3.32	0.000906126	0.022086204	959.444619	1533.87066	1296.68657	1210.16289	685.922049	1324.76	1117.38625	1301.0037	4538.51026	2261.00891	1422.42278	1547.48056	1452.17125	1761.004873	1588.54141	2180.818755	RIG|S15	MIM:180535	19	19p13.3	ribosomal pro	protein-codir	RPS15	ribosomal pro	O		40S ribosoma	20210404
CHMP7	ENSG00000147457	91782	625.08	-1.28	-3.32	0.000907639	0.022094393	908.976116	760.928543	34.9424848	160.892836	351.345295	646.960228	389.392535	238.887441	737.500138	699.826878	637.327719	691.342445	686.715855	1156.667436	1258.09663	641.4172975	-	MIM:611130	8	8p21.3	charged mult	protein-codir	CHMP7	charged mult	O		charged mult	20210302
	ENSG00000280195		13.64	-3.66	-3.31	0.00094422	0.022920169	0	0	0	1.43543703	2.79831577	0	7.1218183	9.80351096	60.0371467	69.6257907	5.85059952	7.11000278	21.1531603	19.06077586	9.15103606	5.059960876												
GS1-24F4.2	ENSG00000245857	100652791	4.05	4.29	3.31	0.000943087	0.022920169	3.58815933	1.34084126	9.12546935	4.95109612	21.0566489	12.0361523	2.58906271	6.459776	0	0	0	0	2.97546511	0	0.71394229	0	-	Ensembl:ENS	8	8p23.1	uncharacteriz	ncRNA	-	-	-		-	20210302
PLD6	ENSG00000179598	201164	29.30	-2.02	-3.31	0.000947134	0.022938471	11.4368457	20.1457383	0	34.9613865	10.3490311	20.5427675	0	15.6809075	46.0684445	40.1915413	54.7181116	86.829258	25.6250265	60.37505321	18.7625607	23.19223721	ZUC	MIM:614960	17	17p11.2	phospholipas	protein-codir	PLD6	phospholipas	O		mitochondria	20210302
	ENSG00000253133		38.36	-2.31	-3.30	0.000956613	0.023138604	6.1118106	14.0974952	0	1.61108824	13.5114031	3.24493568	21.5415724	4.26800232	200.984194	269.36254	2.15365717	13.1260215	12.4027112	16.49266294	10.7945001	24.0410711												
ATG5	ENSG00000057663	9474	285.56	0.70	3.30	0.000959567	0.023180596	384.927645	335.473989	603.560525	282.290557	400.933346	276.034371	239.138972	331.162806	184.347629	157.562104	226.898168	278.61638	200.035644	184.3095025	235.634647	248.1131172	APG5|APG5-	MIM:604261	6	6q21	autophagy re	protein-codir	ATG5	autophagy re	O		autophagy pr	20210417
AHCY	ENSG00000101444	191	118.91	-1.76	-3.30	0.000972868	0.023472143	111.558075	46.6160007	151.033136	105.239861	0	40.9653731	12.4190757	6.24411826	867.173717	97.6956409	70.621249	115.268057	62.7339137	98.47846617	80.0636532	36.49052065	SAHH|adoHc	MIM:180960	20	20q11.22	adenosylhom	protein-codir	AHCY	adenosylhom	O		adenosylhom	20210404
SNORD116-2	ENSG00000278715	100033431	17.54	-3.10	-3.30	0.000977679	0.023473533	13.3304469	48.965798	0	0	0	5.24613612	0	6.79385854	36.7126368	24.4207367	17.0520958	20.0780508	6.57814759	24.57708228	49.831648	27.0888086	HBII-85-20	HGNC:HGNC:	15	15q11.2	small nucleol	snoRNA	SNORD116-2	small nucleol	O		-	20210302
IGSF9B	ENSG00000080854	22997	99.68	-2.13	-3.30	0.0009758	0.023473533	42.0978125	76.0004109	0	9.85447103	9.6001226	99.1880015	114.081919	16.1914431	69.8624259	147.577731	208.211181	244.754504	171.988933	289.0983584	52.6812137	43.63184981	LINC00947|N	MIM:613773	11	11q25	immunoglobu	protein-codir	IGSF9B	immunoglobu	O		protein turtle	20210404
CERS6	ENSG00000172292	253782	386.93	-1.58	-3.30	0.000975197	0.023473533	545.88042	243.742793	682.847555	63.3511265	46.6203633	389.50559	77.0973418	47.992227	504.387756	280.941371	368.323796	759.549549	407.038131	658.0721568	837.573292	277.8959633	CERS5|LASS6	MIM:615336	2	2q24.3	ceramide syn	protein-codir	CERS6	ceramide syn	O		ceramide syn	20210302
CTSB	ENSG00000164733	1508	310.26	-0.99	-3.30	0.000977858	0.023473533	208.088761	178.32859	292.150582	114.756996	251.434391	118.439562	163.157398	163.331489	1165.18631	270.029733	191.244823	216.249904	745.619776	281.9517756	216.454154	387.732692	APPS|CPSB|F	MIM:116810	8	8p23.1	cathepsin B	protein-codir	CTSB	cathepsin B	O		cathepsin B|/	20210417
VAMP3	ENSG00000049245	9341	526.52	-1.12	-3.29	0.000986016	0.023609816	311.361427	409.924505	97.5468633	413.034655	136.080196	605.295784	347.686126	319.252793	1095.91919	1168.5346	902.196575	1039.69923	280.334655	439.7741429	328.698734	529.0242787	CEB	MIM:603657	1	1p36.23	vesicle associ	protein-codir	VAMP3	vesicle associ	O		vesicle-associ	20210302
EPHB4	ENSG00000196411	2050	10.18	-2.84	-3.29	0.000991303	0.023706591	9.73431797	5.67880736	4.52611481	0	0	2.55541541	0	4.92337938	20.2038635	33.3363505	13.6191875	8.82851755	14.944212	25.89117786	15.5660581	3.12598285	CMAVM2|HF	MIM:600011	7	7q22.1	EPH receptor	protein-codir	EPHB4	EPH receptor	O		ephrin type-E	20210408
CC2D2A	ENSG00000048342	57545	31.31	-3.13	-3.29	0.000997133	0.023816094	5.90031126	6.30897177	12.8227543	0	0.72272052	0.92238368	0.20826296	1.03940816	323.017174	82.062578	6.52643839	0	11.6112997	24.56926496	25.2608726	0	COACH2|JBT	MIM:612013	4	4p15.32	coiled-coil an	protein-codir	CC2D2A	coiled-coil an	O		coiled-coil an	20210410
FXYD2	ENSG00000137731	486	30.06	-3.43	-3.29	0.001001429	0.023888722	12.6456873	16.6707168	0	0	0	0	0	0	416.426501	13.442897	2.22333315	3.47524098	0.35706347	5.82713568	6.52709641	3.334042364	ATP1G1|HON	MIM:601814	11	11q23.3	FXYD domain	protein-codir	FXYD2	FXYD domain	O		sodium/pota	20210302
B3GNT4	ENSG00000176383	79369	9.93	-3.24	-3.29	0.001002883	0.023893469	7.33055553	10.4145896	0	0	0	0	0	1.32792065	41.7458947	48.8678664	0	2.31059944	5.14297312	5.053658896	3.91400678	32.73508955	B3GN-T4|bet	MIM:605864	12	12q24.31	UDP-GlcNAc:	protein-codir	B3GNT4	UDP-GlcNAc:	O		N-acetyllacto	20210302
TRBV6-1	ENSG00000211706	28606	18.71	-2.38	-3.29	0.001005892	0.023925391	25.7595546	20.371882	0	1.98883312	0	1.55506202	15.8704051	5.67795285	32.9603361	16.246552	26.4396235	51.4019316	6.89385556	16.96211448	34.0970458	43.08799622	TCRBV13S3|T	HGNC:HGNC:	7	7q34	T cell recepto	other	TRBV6-1	T cell recepto	O		T-cell recepto	20210302
	ENSG00000261093		8.28	-2.93	-3.29	0.001014843	0.024087958	7.29044581	2.04792933	0	0	2.92244171	0	0.73640304	1.31613132	34.6111285	43.3225106	3.59969708	0	9.91981308	9.982777547	7.65933006	9.011096808												
LARP7	ENSG00000174720	51574	435.38	0.59	3.28	0.00102464	0.024290212	504.041567	726.333416	898.903052	544.832384	369.435004	456.190692	311.966031	438.527171	279.940608	314.815904	462.097592	409.612905	300.161171	331.1630982	348.296429	269.7603176	ALAZS|HDCN	MIM:612026	4	4q25	La ribonuclec	protein-codir	LARP7	La ribonuclec	O		la-related pro	20210307
DNAI7	ENSG00000118307	55259	36.77	-3.48	-3.28	0.001037329	0.024560448	15.0450739	0	0	0	0	12.7057868	0	0	534.207346	3.21970349	0.75913472	7.12388462	3.46166964	3.274501398	4.68846505	3.890272717	CASC1|CFAP	MIM:616906	12	12p12.1	dynein axone	protein-codir	DNAI7	dynein axone	O		dynein intern	20210302
RHOH	ENSG00000168421	399	708.19	-0.63	-3.28	0.001045539	0.024724085	399.252662	824.830375	558.681485	359.830932	455.149479	721.144603	482.424175	700.082403	565.434397	651.540104	752.181092	957.136998	806.483182	1077.942291	946.674987	1072.188588	ARHH|TTF	MIM:602037	4	4p14	ras homolog	protein-codir	RHOH	ras homolog	O		rho-related G	20210302
	ENSG00000273319		36.19	-2.51	-3.28	0.001048645	0.024736058	8.11875522	31.2219251	0	0	17.3180096	0	18.0937593	5.42941499	190.624655	228.8295	3.79871856	3.6259002	20.705018	21.79199359	10.2681054	19.28780889												
TMEM154	ENSG00000170006	201799	445.36	-1.08	-3.28	0.001047959	0.024736058	471.5109	267.588364	193.909053	98.043297	27.2138897	179.923313	250.952112	186.532377	3427.26816	246.189251	168.953154	167.001532	130.353751	344.4541036	432.499489	533.2942074	-	HGNC:HGNC:	4	4q31.3	transmembra	protein-codir	TMEM154	transmembra	O		transmembra	20210302
TBC1D4	ENSG00000136111	9882	258.06	-1.62	-3.28	0.001050235	0.024742908	552.134219	580.55103	145.857847	14.8079145	14.280021	166.419096	96.8911519	27.1207814	384.068029	362.122344	374.531609	355.824268	222.315422	359.2477501	376.16716	96.6729991	AS160|NIDDI	MIM:612465	13	13q22.2	TBC1 domain	protein-codir	TBC1D4	TBC1 domain	O		TBC1 domain	20210302
FAM184B	ENSG00000047662	27146	143.82	2.53	3.27	0.00105755	0.024853732	8.21309202	10.8369046	15.4512183	4.89966467	1057.32201	488.019595	201.78673	177.354393	76.3179442	129.048934	0.64356268	2.34075361	84.8366445	18.32293146	11.8290146	13.8667624	-	HGNC:HGNC:	4	4p15.32-p15.	family with se	protein-codir	FAM184B	family with se	O	-	protein FAM1	20210302

MAPKAPK5-A	ENSG00000234608	51275	162.94	-1.28	-3.27	0.001064039	0.024913958	137.257971	138.490326	8.282287	177.662178	32.2904413	134.034724	125.624219	74.8245191	431.675869	132.16994	190.341355	335.503753	125.807669	226.0115272	198.553823	138.4490419	C12orf47	HGNC:HGNC:	12	12q24.12	MAPKAPK5 a	ncRNA	MAPKAPK5-A	MAPKAPK5 a	O	MAPKAPK5 a	20210406
FTL	ENSG00000087086	2512	4164.94	-0.85	-3.27	0.001063554	0.024913958	3969.22522	3080.38617	2585.04762	2598.82923	1086.41437	1898.15673	1885.3646	1662.72498	25615.6566	3235.23698	2957.9329	5332.58189	2663.38269	2754.346999	2540.41453	2773.38052	LFTD|NBIA3	MIM:134790	19	19q13.33	ferritin light c	protein-codir	FTL	ferritin light c	O	ferritin light c	20210408
ND4	ENSG00000198886	4538	78718.31	0.73	3.27	0.001061807	0.024913958	199639.476	194821.948	138642.886	56061.387	33736.1077	70095.5618	57185.128	48854.2536	160598.171	45369.6794	70502.8184	34525.4062	33231.4767	30866.89073	37960.5267	47401.24582	MTND4	MIM:516003	MT	-	NADH dehydı	protein-codir	MT-ND4	mitochondria	O	NADH dehydı	20210407
SNPH	ENSG00000101298	9751	83.27	-1.58	-3.27	0.001065619	0.024920303	109.65573	119.324837	0	11.1337512	33.496073	87.8255662	20.9155934	51.7651762	171.214213	111.817035	80.3585063	95.5209057	101.593678	119.6626736	111.669673	106.2940065	-	MIM:604942	20	20p13	syntaphilin	protein-codir	SNPH	syntaphilin	O	syntaphilin	20210302
RPL9P8	ENSG00000237550	254948	1746.91	-0.58	-3.27	0.001072109	0.025041316	1776.69019	1740.63052	2446.61537	1772.41765	506.415986	1456.53504	585.566166	1186.21427	2514.59601	1630.49566	2384.12164	3758.70975	1003.22373	1845.308789	1159.42568	2183.636758	RPL9P9|RPL9	HGNC:HGNC:	15	15q25.2	ribosomal pro	pseudo	RPL9P8	ribosomal pro	O	ribosomal pro	20210302
SCNN1A	ENSG00000111319	6337	54.80	-3.04	-3.27	0.001081072	0.025219714	14.5410069	57.4807359	0	0	0	0	1.75938644	0.48246249	764.003382	6.78450204	4.12337712	4.07919625	2.59587875	1.855775969	6.29119274	12.82335629	BESC2|ENaCa	MIM:600228	12	12p13.31	sodium chanı	protein-codir	SCNN1A	sodium chanı	O	amiloride-ser	20210302
SLC4A11	ENSG00000088836	83959	7.00	-3.51	-3.27	0.001082994	0.025233625	1.66487419	4.41705612	0	0	0	0	0	2.93036515	61.7776726	7.35352706	4.10159089	4.29562215	5.43895821	4.138603819	3.41560733	12.40333097	BTR1|CDPD1	MIM:610206	20	20p13	solute carrier	protein-codir	SLC4A11	solute carrier	O	sodium bicarl	20210410
BOD1L1	ENSG00000038219	259282	2227.72	0.59	3.27	0.001086256	0.025278698	2574.49714	2132.51442	3536.21282	2587.62695	4468.97216	2131.409	1941.59882	2093.94879	3091.49478	1354.87626	1570.22191	1835.03546	1820.65184	1650.475018	1554.52504	1299.501543	BOD1L|FAM4	MIM:616746	4	4p15.33	biorientation	protein-codir	BOD1L1	biorientation	O	biorientation	20210307
PATZ1	ENSG00000100105	23598	212.06	-1.08	-3.27	0.001091227	0.025363373	238.59882	203.926233	15.3931763	151.333175	122.401015	157.790448	129.527571	159.352165	264.810317	292.330017	235.588723	276.468799	271.842519	283.4310025	325.762417	264.3514347	MAZR|PATZ|	MIM:605165	22	22q12.2	POZ/BTB and	protein-codir	PATZ1	POZ/BTB and	O	POZ-, AT hoo	20210302
C1RL-AS1	ENSG00000205885	283314	83.63	1.31	3.27	0.001093987	0.025396507	37.5189076	92.2493505	58.64914	118.106779	393.555745	73.0688486	83.0071153	123.072282	36.2687882	40.66295	34.7878072	13.2817295	74.7052313	76.6254991	22.3005738	60.22608598	MATL2963	HGNC:HGNC:	12	12p13.31	C1RL antisens	ncRNA	C1RL-AS1	C1RL antisens	O	ABC12-49244	20210302
	ENSG00000227518		19.06	-3.47	-3.26	0.001104542	0.025579151	5.11544303	0	0	1.58569451	15.3427826	0	0	1.34263382	109.851591	134.279792	1.86081997	3.4716541	5.31286069	3.975866136	17.5214096	5.324657937											
GZF1	ENSG00000125812	64412	139.29	-0.72	-3.26	0.001114669	0.025782312	109.264125	164.839489	18.926234	115.197572	153.772328	83.7088881	82.5001105	104.567942	203.966561	227.720662	134.109517	151.880868	191.381192	114.1781661	213.712494	158.8767558	JLSM|ZBTB23	MIM:613842	20	20p11.21	GDNF inducib	protein-codir	GZF1	GDNF inducib	O	GDNF-inducib	20210302
CARMIL2	ENSG00000159753	146206	231.16	-0.75	-3.26	0.001116865	0.025801758	340.657506	146.72134	75.7757674	71.7711847	177.342779	212.874243	226.42142	181.197047	284.294027	369.907381	211.278636	153.406602	307.451049	373.9864745	351.622174	213.8282998	CARMIL2b|IN	MIM:610859	16	16q22.1	capping prote	protein-codir	CARMIL2	capping prote	O	capping prote	20210302
ST6GALNAC3	ENSG00000184005	256435	18.92	-3.64	-3.26	0.001133239	0.026148298	0	53.2663867	0	35.2522189	0	3.38282999	0	0.35387873	1.17893986	25.9586379	40.9166132	6.76559445	4.02149885	40.79738435	56.3808365	34.50059662	PRO7177|SIA	MIM:610133	1	1p31.1	ST6 N-acetylg	protein-codir	ST6GALNAC3	ST6 N-acetylg	O	alpha-N-acet	20210417
ZDHHC9	ENSG00000188706	51114	30.13	-2.58	-3.25	0.001137941	0.026224991	28.8872828	31.5052751	5.89442766	4.14005824	4.63072381	66.0739925	0	0.39971876	32.4634094	27.0107787	21.8222208	62.6623292	66.7126233	63.0648173	23.2030467	43.6634255	CGI89|CXorf1	MIM:300646	X	Xq26.1	zinc finger DH	protein-codir	ZDHHC9	zinc finger DH	O	palmitoyltrar	20210302
ANAPC16	ENSG00000166295	119504	704.19	-0.64	-3.25	0.001140421	0.026250365	992.077573	906.070506	512.421875	359.229759	446.196294	475.872229	326.60322	340.524608	2099.8823	757.171859	716.497864	948.434036	578.940779	577.0472078	613.647253	616.417906	APC16|C10oı	MIM:613427	10	10q22.1	anaphase prc	protein-codir	ANAPC16	anaphase prc	O	anaphase-prc	20210307
TPGS1	ENSG00000141933	91978	54.27	-1.67	-3.25	0.001152204	0.02648955	31.3597147	43.8525939	10.049813	16.7618704	2.64490502	9.63861343	63.2529482	79.8029096	61.0682242	87.4828072	54.66185	46.626817	84.2060089	106.8489164	74.5268907	95.49599955	C19orf20|GT	HGNC:HGNC:	19	19p13.3	tubulin polyg	protein-codir	TPGS1	tubulin polyg	O	tubulin polyg	20210302
ZNF37BP	ENSG00000234420	100129482	422.32	0.67	3.25	0.001158354	0.026598818	451.077845	329.817039	457.720455	595.684747	755.204611	451.815602	495.679578	608.380485	300.59919	281.73542	349.614937	325.192047	410.676147	392.0473229	376.732979	175.0653271	KOX21|ZNF3	HGNC:HGNC:	10	10q11.21	zinc finger pr	pseudo	ZNF37BP	zinc finger pr	O	zinc finger pr	20210302
ZNF581	ENSG00000171425	51545	54.44	-1.52	-3.24	0.001189912	0.027192281	111.482675	102.496812	0	11.8518224	32.5341075	14.2234436	6.35225308	29.8667059	125.854958	112.446034	41.3822124	46.7352084	40.6297559	67.42164316	39.7380112	87.97351779	HSPC189	HGNC:HGNC:	19	19q13.42	zinc finger pr	protein-codir	ZNF581	zinc finger pr	O	zinc finger pr	20210417
PRF1	ENSG00000180644	5551	1888.31	0.71	3.24	0.001197197	0.027325952	1245.77036	1066.58861	2490.31226	2862.28585	2741.55791	2148.47329	2967.21859	2905.31454	667.859235	1366.30328	1072.22189	1407.10034	2186.2203	861.8809078	1244.4114	2979.442159	HPLH2|P1|PI	MIM:170280	10	10q22.1	perforin 1	protein-codir	PRF1	perforin 1	O	perforin-1|cy	20210410
KLF5	ENSG00000102554	688	272.00	-1.88	-3.23	0.001218682	0.027749808	312.206225	77.6477026	0	5.80125599	0	32.2847963	10.657688	15.7702115	3676.45458	43.7209964	28.1807239	21.6858304	23.6683693	25.28871687	42.0413404	36.61018478	BTEB2|CKLF|	MIM:602903	13	13q22.1	Kruppel like f	protein-codir	KLF5	Kruppel like f	O	Krueppel-like	20210418
NBAS	ENSG00000151779	51594	675.84	0.66	3.23	0.001224222	0.027842636	1007.83247	621.510434	1282.71941	707.580174	538.490721	924.56463	802.554161	793.707947	321.524243	511.567218	512.614851	585.343558	514.229759	540.9186859	608.84514	539.4907203	ILFS2|NAG|S	MIM:608025	2	2p24.3	NBAS subunit	protein-codir	NBAS	NBAS subunit	O	neuroblaston	20210307
SELENON	ENSG00000162430	57190	129.52	-0.97	-3.23	0.00123355	0.028014613	145.108415	194.200248	32.3889062	59.7156813	112.593504	72.6559643	101.357189	42.0014965	192.50086	149.352096	110.982865	148.12806	154.076387	199.0625508	195.024565	163.1029457	CFTD|MDRS1	MIM:606210	1	1p36.11	selenoproteir	protein-codir	SELENON	selenoproteir	O	selenoproteir	20210410
	ENSG00000268947		7.08	-4.06	-3.23	0.001241211	0.028061398	0	12.5109059	0	0	0	9.87484744	0	0	6.20347625	13.4096587	15.2488423	3.2625565	14.1611268	18.70029784	12.8561193	6.972917654											
CD40LG	ENSG00000102245	959	32.24	-2.21	-3.23	0.001239927	0.028061398	61.2731263	32.3328988	0	8.28360006	2.37830531	10.0816663	26.3748661	2.47301781	31.9178533	41.5078167	35.2008106	37.1515939	44.7732493	99.15229235	30.7183228	52.18682525	CD154|CD40	MIM:300386	X	Xq26.3	CD40 ligand	protein-codir	CD40LG	CD40 ligand	O	CD40 ligand|	20210410
KLHL29	ENSG00000119771	114818	26.31	-2.08	-3.23	0.001238265	0.028061398	15.4382037	6.41901757	0	0	8.55823702	12.4336357	4.84487613	3.31785412	266.073319	40.5292598	4.88879759	5.88715305	15.3608669	14.75901319	8.54836156	13.88645935	KBTBD9	HGNC:HGNC:	2	2p24.1	kelch like fam	protein-codir	KLHL29	kelch like fam	O	kelch-like prc	20210302
MCTP2	ENSG00000140563	55784	1062.09	0.87	3.23	0.001242765	0.028063206	405.346587	431.287427	2911.21281	1195.39098	2644.14093	1065.47056	1253.79546	1584.8979	380.370364	688.221605	665.183114	469.757603	1202.47001	524.765823	561.24686	1009.956086	-	MIM:616297	15	15q26.2	multiple C2 a	protein-codir	MCTP2	multiple C2 a	O	multiple C2 a	20210417
LINC01550	ENSG00000246223	388011	134.01	-1.56	-3.23	0.001244453	0.028068027	167.061523	409.118944	0	57.123916	24.775104	78.8537882	35.5908447	44.1905219	197.727735	215.384854	115.074634	181.701606	112.565011	234.7558919	164.186693	106.1286242	C14orf64	HGNC:HGNC:	14	14q32.2	long intergen	ncRNA	LINC01550	long intergen	O	-	20210302
ZRSR2	ENSG00000169249	8233	153.34	0.75	3.23	0.001248398	0.02809042	122.22196	111.841915	204.482393	208.710488	262.421659	196.237135	297.207402	142.43438	74.4406112	126.512096	152.148308	100.721264	155.620844	122.5567272	94.5787213	81.30396258	U2AF1-RS2|U	MIM:300028	X	Xp22.2	zinc finger CC	protein-codir	ZRSR2	zinc finger CC	O	U2 small nucl	20210302
	ENSG00000224032		315.93	-1.06	-3.23	0.001257056	0.028218544	349.46229	321.386521	37.0416274	278.036837	78.4825213	289.133162	244.400609	171.265174	696.616818	494.745494	421.687643	463.861735	260.313278	292.9877197	333.966727	321.4927697											
DNAJB1	ENSG00000132002	3337	647.91	-0.85	-3.22	0.001260906	0.02827163	1161.07081	934.719278	226.008771	419.395642	189.703952	414.362769	344.476547	372.200557	951.097637	829.443915	855.48931	965.230504	461.740823	517.9798396	853.612566	870.0854528	HSPF1|Hdj1|	MIM:604572	19	19p13.12	DnaJ heat sho	protein-codir	DNAJB1	DnaJ heat sho	O	dnaJ homolog	20210307
TFB1M	ENSG00000029639	51106	76.08	-1.19	-3.22	0.001267035	0.02834227	113.939162	43.1867544	0	35.2931508	40.7672143	41.2532125	80.577299	42.8238553	188.159648	63.3898215	75.8639411	55.5247791	118.182717	86.02910957	135.968016	96.32015892	CGI-75|CGI75	MIM:607033	6	6q25.3	transcription	protein-codir	TFB1M	transcription	O	dimethylader	20210404
NCAM1	ENSG00000149294	4684	124.87	1.46	3.22	0.00127149	0.028408552	154.69627	48.3162579	529.783657	42.6248974	89.5998915	91.9136971	130.814924	367.461352	113.279434	56.334448	94.8171715	4.51703519	79.4108341	58.16685669	8.88592149	127.2184402	CD56|MSK39	MIM:116930	11	11q23.2	neural cell ad	protein-codir	NCAM1	neural cell ad	O	neural cell ad	20210418
	ENSG00000267165		10.75	-3.88	-3.22	0.001297067	0.028912147	1.19951618	4.43106485	3.81906959	0	0	0	0	0	51.0795575	83.3485483	0.82982509	0	3.63181675	3.123372171	8.49020842	12.0292309											
C11orf65	ENSG00000166323	160140	10.88	-3.33	-3.22	0.001296593	0.028912147	0	0.89096879	0	7.8149132	0	0.92253274	0.35950275	6.57637044	59.1858348	53.0843139	21.1932433	5.63825623	3.36154922	4.217543908	5.90586643	4.937178789	MFI	HGNC:HGNC:	11	11q22.3	chromosome	protein-codir	C11orf65	chromosome	O	protein MFI|	20210302
	ENSG00000268173		10.38	-4.90	-3.21	0.001305914	0.029075306	0	0	0	0	0	0	0	7.30947395	0	0	21.9594194	23.3947838	44.5834786	20.76642761	15.5782239	32.53615231											
NR2C2AP	ENSG00000184162	126382	20.91	-2.06	-3.21	0.001308315	0.029094736	4.55249815	27.5401154	0	2.49320437	7.42098177	0.74418897	25.506624	18.657097	14.2735715	46.0746411	29.462532	15.3179223	26.6961715	37.48757132	33.4123433	44.84094372	TRA16	MIM:608719	19	19p13.11	nuclear recep	protein-codir	NR2C2AP	nuclear recep	O	nuclear recep	20210302
DYNC2H1	ENSG00000187240	79659	507.04	0.91	3.21	0.001312487	0.02915344	717.48332	472.424267	760.281592	1395.33929	522.319948	308.245355	326.996141	414.70537	1486.60704	295.934054	325.381314	329.00977	221.283868	165.1247114	161.569851	209.8666606	ATD3|DHC1b	MIM:603297	11	11q22.3	dynein cytop	protein-codir	DYNC2H1	dynein cytop	O	cytoplasmic c	20210302
NPEPPS	ENSG00000141279	9520	1065.42	0.71	3.21	0.001314904	0.029173085	794.861025	562.885796	3109.08199	1094.27777	1455.36432	1921.74901	1245.31935	852.210109	700.725872	610.561449	756.538927	661.964548	876.619944	1120.449636	656.210173	627.8715366	AAP-S|MP10	MIM:606793	17	17q21.32	aminopeptida	protein-codir	NPEPPS	aminopeptida	O	puromycin-se	20210417
	ENSG00000279530		11.83	-3.56	-3.21	0.001321996	0.029264852	0	1.53620774	0	1.78034824	0	0	0	1.21193505	70.6248804	94.6653004	0.80668301	1.56301547	9.82679052	1.987121483	1.35355905	3.928233723											
LHFPL5	ENSG00000197753	222662	20.54	-2.49	-3.21	0.001322115	0.029264852	11.2715017	6.41647796	0	0	0	0.68104708	2.40288485	4.56668738	164.099116	111.513274	0	1.18092098	2.43038876	7.859121959	9.4241462	6.831462309	DFNB67|TMI	MIM:609427	6	6p21.31	LHFPL tetrasp	protein-codir	LHFPL5	LHFPL tetrasp	O	LHFPL tetrasp	20210410
TRBV7-2	ENSG00000282939	28596	22.17	-2.67	-3.21	0.001333676	0.029324342	43.9207996	33.7992967	0	13.9025462	0	3.74732329	6.0343702	0	17.0806102	41.0203036	62.0423966	39.8956763	13.1046639	31.47681092	34.9798932	13.75910128	TCRBV6S5A1	HGNC:HGNC:	7	7q34	T cell recepto	other	TRBV7-2	T cell recepto	O	T-cell recepto	20210302
CTSL	ENSG00000135047	1514	34.19	-2.65	-3.21	0.001333789	0.029324342	7.60576161	24.6860333	45.9436346	1.21322742	0	0	6.07799747	4.30411761	235.971724	35.9549597	14.6394163	5.73460667	109.990789	11.29459032	34.1022484	9.525346684	CATL|CTSL1|	MIM:116880	9	9q21.33	cathepsin L	protein-codir	CTSL	cathepsin L	O	procathepsin	20210418
H4C11	ENSG00000197238	8363	117.62	-2.43	-3.21	0.001333034	0.029324342	106.222988	4.5156038	0	0	74.1143101	66.3212005	145.644152	81.1843791	99.2561086	153.596663	147.263315	277.849671	214.182156	121.8559428	214.001491	175.9386639	H4-16|H4/e|	MIM:602826	6	6p22.1	H4 clustered	protein-codir	H4C11	H4 clustered	O	histone H4|H	20210302
SNORD56	ENSG00000229686	26793	53.58	-2.40	-3.21	0.001334988	0.029324342	127.584462	102.471762	0	8.51603217	0	0	0	0.82250877	207.788367	261.142857	80.3754445	50.7762852	0	0	5.4451186	12.38940211	RNU56|U56	HGNC:HGNC:	20	20p13	small nucleol	snoRNA	SNORD56	small nucleol	O	RNA, U56 sm	20210302
NLE1	ENSG00000073536	54475	45.37	-1.42	-3.21	0.001334274	0.029324342	36.5144695	33.1810184	0	43.8795745	29.9530942	12.3257667	20.7508271	35.233884	62.7668474	88.437315	28.2957776	84.6321281	53.0059231	78.76117683	87.882325	30.3675711	NLE	HGNC:HGNC:	17	17q12	notchless hor	protein-codir	NLE1	notchless hor	O	notchless prc	20210302
CENPF	ENSG00000117724	1063	371.61	1.40	3.21	0.001335585	0.029324342	339.318582	87.0504159	2566.94747	178.000841	889.360686	272.929075	182.089708	260.637413	87.7881792	61.1577598	62.8179822	62.5756179	420.932711	106.7526235	112.439603	254.9253625	CENF|CILD31	MIM:600236	1	1q41	centromere p	protein-codir	CENPF	centromere p	O	centromere p	20210302
RNFT1	ENSG00000189050	51136	173.96	0.65	3.21	0.001338867	0.02936253	161.04959	99.503351	242.201408	225.198994	300.994889	229.013639	230.842997	218.799686	109.009162	109.154989	178.127988	140.900085	160.595566	134.8836669	159.135204	83.87524316	PTD016	MIM:615172	17	17q23.1	ring finger pr	protein-codir	RNFT1	ring finger pr	O	E3 ubiquitin-p	20210302
NFE2L3	ENSG00000050344	9603	77.78	-1.99	-3.21	0.001345764	0.02941247	33.1725921	39.6771188	0	13.4701599	38.4145085	33.5306096	5.18005924	40.4561343	360.300469	317.256595	36.021351	15.0797208	33.3243763	59.08981998	180.345948	39.16190598	NRF3	MIM:604135	7	7p15.2	nuclear facto	protein-codir	NFE2L3	nuclear facto	O	nuclear facto	20210302
SNORD45A	ENSG00000207241	26805	93.22	-1.93	-3.21	0.001347325	0.02941247	257.269326	110.275775	0	21.5926766	0	20.3276153	25.363088	33.9113281	210.622197	321.311214	115.627914	131.677042	44.6806066	20.66639522	42.0829762	136.1185401	RNU45A|U45	HGNC:HGNC:	1	1p31.1	small nucleol	snoRNA	SNORD45A	small nucleol	O	RNA, U45A sr	20210302
LRRC8B	ENSG00000197147	23507	297.00	-0.81	-3.21	0.001345177	0.02941247	471.642761	244.821822	160.946484	179.850822	187.188697	396.167822	134.267642	58.2579574	465.370391	267.90445	351.318865	363.169116	210.791908	686.4290932	344.694797	229.2005303	TA-LRRP|TAL	MIM:612888	1	1p22.2	leucine rich r	protein-codir	LRRC8B	leucine rich r	O	volume-regul	20210302
LPCAT1	ENSG00000153395	79888	691.14	0.72	3.21	0.001347063	0.02941247	375.019933	362.350272	1489.07417	1408.77324	961.159272	995.125453	687.570592	711.908433	195.495658	404.356677	594.273761	481.018875	909.195012	409.7923595	469.060891	604.0228866	AGPAT10|AG	MIM:610472	5	5p15.33	lysophosphat	protein-codir	LPCAT1	lysophosphat	O	lysophosphat	20210307
EFHC1	ENSG00000096093	114327	192.56	1.40	3.20	0.001352446	0.029456702	171.940946	276.01766	691.919572	167.085971	178.602543	290.90501	57.7009251	122.953097	661.573599	129.252108	95.0888605	34.1384521	61.4584885	68.84755031	45.1975381	28.31916895	EJM1|POC9|	MIM:608815	6	6p12.2	EF-hand dom	protein-codir	EFHC1	EF-hand dom	O	EF-hand dom	20210417
GRAPL	ENSG00000189152	400581	28.35	-2.32	-3.20	0.001362924	0.029650995	33.9590075	0	0	18.4792279	0	22.5046298	23.2489498	10.4555415	34.6910451	23.7244971	27.4234456	28.8037762	62.5328227	51.53595146	86.0755689	30.2339198	-	HGNC:HGNC:	17	17p11.2	GRB2 related	protein-codir	GRAPL	GRB2 related	O	GRB2-related	20210302
ATP6AP1	ENSG00000071553	537	400.22	-0.73	-3.20	0.001385734	0.030010058	124.700177	441.944766	227.452621	501.505606	279.311979	189.861011	307.820869	455.576786	364.79516	396.968191	462.716189	498.840287	529.871125	512.0662712	486.743658	623.4019699	16A|ATP6IP1	MIM:300197	X	Xq28	ATPase H+ tra	protein-codir	ATP6AP1	ATPase H+ tra	O	V-type protor	20210417
LINC01624	ENSG00000227508	401289	4.39	-4.49	-3.20	0.001389108	0.030048923	0	2.40438258	0	0	0	0	0	0	20.2412503	8.52603671	1.7810451	1.88352523	14.4520667	4.347758901	10.4884315	6.098809824	-	HGNC:HGNC:	6	6q27	long intergen	ncRNA	LINC01624	long intergen	O	TCONS_0001	20210302
	ENSG00000279033		9.08	-3.50	-3.19	0.001400426	0.030225073	2.54293698	8.47342424	0	0	0	0	0	0	40.8136581	71.0521261	0.76079422	0.69621135	0.53987479	8.631884974	3.99508312	7.802699702											
TLR2	ENSG00000137462	7097	20.83	-2.30	-3.19	0.001399181	0.030225073	37.2847114	4.53423824	0	24.2491095	10.8309186	0.66700384	1.82639226	0	110.894633	5.21238882	12.1180238	32.8212463	43.6538274	36.97085484	8.18737485	3.989402815	CD282|TIL4	MIM:603028	4	4q31.3	toll like recep	protein-codir	TLR2	toll like recep	O	toll-like recep	20210418
MUL1	ENSG00000090432	79594	90.91	-0.83	-3.19	0.001408336	0.030327026	58.9510433	82.1058517	27.3948917	122.45042	41.971918	74.6845695	54.5477551	74.5115457	82.2692761	167.644514	96.2032805	130.647169	98.9281899	93.09777511	132.274171	116.9163666	C1orf166|GIE	MIM:612037	1	1p36.12	mitochondria	protein-codir	MUL1	mitochondria	O	mitochondria	20210404
SKAP2	ENSG00000005020	8935	310.12	0.87	3.19	0.001415918	0.030455831	387.622592	462.772205	644.978628	303.094995	241.280802	547.065324	254.729258	375.978565	276.456403	233.800882	129.034947	257.931381	234.340294	178.4790962	123.092624	311.3160842	PRAP|RA70|S	MIM:605215	7	7p15.2	src kinase ass	protein-codir	SKAP2	src kinase ass	O	src kinase-ass	20210302
TMEM220	ENSG00000187824	388335	17.44	-2.74	-3.18	0.001454546	0.031062638	14.5301382	1.44400901	0	0	38.8086402	5.6923269	6.74079225	0	48.4842589	73.2495	6.0482099	7.36754055	22.7808265	20.16226475	15.2941765	18.4252403	-	HGNC:HGNC:	17	17p13.1	transmembra	protein-codir	TMEM220	transmembra	O	transmembra	20210302
CMTM8	ENSG00000170293	152189	19.36	-2.29	-3.18	0.001455551	0.031062638	17.9183742	9.10379822	0	4.64572049	0	18.2898655	0.8469217	6.38863499	120.432565	13.2291082	16.651429	8.04239722	11.1538244	26.47823163	34.3882397	22.16434775	CKLFSF8|CKL	MIM:607891	3	3p22.3	CKLF like MA	protein-codir	CMTM8	CKLF like MA	O	CKLF-like MA	20210418
FAM157A	ENSG00000236438	728262	47.05	-1.97	-3.18	0.001454229	0.031062638	8.19320549	7.13847646	39.833911	4.03025649	53.3129038	129.63493	0.76870335	8.01235201	55.6775902	66.4946783	35.6917535	71.3116539	127.991843	100.4134406	34.1353202	10.1098925	GTF2IP18	HGNC:HGNC:	3	3q29	family with se	ncRNA	FAM157A	family with se	O	family with se	20210302
FCMR	ENSG00000162894	9214	1208.79	-0.91	-3.18	0.001449155	0.031062638	2172.883	1067.14295	917.09514	886.809741	295.15513	931.803994	641.167649	343.694047	1441.22834	1246.80867	1056.26826	1743.68858	772.792407	1893.043302	2455.70196	1475.405328	FAIM3|TOSO	MIM:606015	1	1q32.1	Fc fragment c	protein-codir	FCMR	Fc fragment c	O	fas apoptotic	20210302
SATB2	ENSG00000119042	23314	68.30	2.01	3.18	0.001455185	0.031062638	9.89339605	71.6928242	328.649288	60.2618848	68.4268107	37.4622248	144.621301	104.187823	0	82.7384033	5.99583861	20.8436721	83.5307832	8.500372185	8.73968947	57.27615571	GLSS	MIM:608148	2	2q33.1	SATB homeol	protein-codir	SATB2	SATB homeol	O	DNA-binding	20210417
EIF6	ENSG00000242372	3692	108.07	-1.07	-3.18	0.001460819	0.031105315	97.3417671	98.5524129	3.68242047	62.072236	58.880569	89.1207597	77.7156291	94.443974	243.784862	119.598915	114.948418	136.489348	113.641065	118.4563321	145.634232	154.788228	CAB|EIF3A|IT	MIM:602912	20	20q11.22	eukaryotic tra	protein-codir	EIF6	eukaryotic tra	O	eukaryotic tra	20210404
SLC14A1	ENSG00000141469	6563	56.15	1.39	3.18	0.001459705	0.031105315	73.3894857	25.9636764	178.047423	22.4731958	33.6441017	91.445787	169.46964	71.886486	31.0313489	22.6542555	55.9137981	10.5070216	48.8777535	9.376216427	28.3909149	25.31670697	HUT11|HsT1	MIM:613868	18	18q12.3	solute carrier	protein-codir	SLC14A1	solute carrier	O	urea transpor	20210302
NKG7	ENSG00000105374	4818	1887.82	0.70	3.18	0.001463945	0.03113704	1238.31047	1718.41951	2394.56395	3920.86126	2004.32467	1884.39819	2632.4204	2308.82296	563.717289	1368.30572	1774.54864	2557.05934	2019.60289	681.7643672	824.100611	2313.923592	GIG1|GMP-1	MIM:606008	19	19q13.41	natural killer	protein-codir	NKG7	natural killer	O	protein NKG7	20210302
RGMB	ENSG00000174136	285704	113.95	-2.02	-3.18	0.001469884	0.03122848	137.437264	116.453731	59.5137435	68.4546626	0	143.440807	20.7568411	2.16911915	137.549633	92.1807934	116.759744	236.391871	105.33852	243.9022681	230.866832	111.9798199	DRAGON	MIM:612687	5	5q15	repulsive guic	protein-codir	RGMB	repulsive guic	O	RGM domain	20210302
NOP16	ENSG00000048162	51491	34.27	-1.13	-3.18	0.001475118	0.031304738	51.4836251	26.1497682	12.5700745	27.6409337	9.80170003	16.2469704	7.78487553	29.6947015	64.6402478	83.1382322	37.6791239	50.4432029	22.2218492	37.36038257	30.866087	40.67806331	HSPC111|HSI	MIM:612861	5	5q35.2	NOP16 nucle	protein-codir	NOP16	NOP16 nucle	O	nucleolar pro	20210302
LGR6	ENSG00000133067	59352	148.17	0.95	3.18	0.001493222	0.031618441	52.4844758	161.938709	502.140649	163.048049	105.338634	192.516782	153.061548	181.539464	19.8497655	167.787166	264.530194	82.2399428	83.6104839	36.82785498	51.4643522	152.2773632	GPCR|VTS20	MIM:606653	1	1q32.1	leucine rich r	protein-codir	LGR6	leucine rich r	O	leucine-rich r	20210302
CD79A	ENSG00000105369	973	9.79	-3.69	-3.17	0.00150652	0.031829199	1.72814191	9.41738699	0	0	0	8.9953471	0	0	7.34446266	13.5927194	2.45605132	16.4093127	62.3684819	6.430485046	23.1708438	4.773152339	IGA|MB-1	MIM:112205	19	19q13.2	CD79a molec	protein-codir	CD79A	CD79a molec	O	B-cell antiger	20210302
ARHGEF35	ENSG00000213214	445328	22.82	-2.40	-3.17	0.001513392	0.031868295	16.0550212	1.26394459	0	3.47769313	12.2712946	0	8.13398432	2.53421213	238.342004	8.85749842	19.195221	21.8273919	8.77179292	6.045150626	9.05716091	9.355177078	ARHGEF5L	HGNC:HGNC:	7	7q35	Rho guanine	protein-codir	ARHGEF35	Rho guanine	O	rho guanine r	20210302
RGS9	ENSG00000108370	8787	109.45	0.96	3.17	0.001526512	0.032109045	119.399069	76.1798733	114.05756	169.900924	150.797348	89.1618617	200.28267	219.052243	108.956562	46.8506556	74.3620332	86.9514181	120.137923	35.73185104	31.8750054	107.5216657	PERRS|RGS9I	MIM:604067	17	17q24.1	regulator of G	protein-codir	RGS9	regulator of G	O	regulator of G	20210302
PTGDR2	ENSG00000183134	11251	16.98	-2.64	-3.17	0.001530528	0.032157979	0.57923059	8.03329634	5.78576695	0	0	30.7876035	1.61628462	0	2.54292699	10.4605796	19.6797803	9.01150998	12.0495623	155.8177337	3.79171446	11.49587585	CD294|CRTH	MIM:604837	11	11q12.2	prostaglandir	protein-codir	PTGDR2	prostaglandir	O	prostaglandir	20210302
PRR5-ARHGA	ENSG00000248405	553158	9.10	-3.68	-3.17	0.001536962	0.032222027	0	4.83477342	0	28.2537711	0	0	0	7.25419295	14.0137306	15.5977608	12.3765837	17.6786409	10.8779932	16.65202547	11.906774	6.231775798	-	HGNC:HGNC:	22	22q13.31	PRR5-ARHGA	protein-codir	PRR5-ARHGA	PRR5-ARHGA	O	PRR5-ARHGA	20210302
SLC8B1	ENSG00000089060	80024	227.39	-1.06	-3.17	0.001535604	0.032222027	215.860568	154.456055	333.805808	99.6828179	134.871585	231.088463	51.120796	58.3113776	448.463644	200.634291	225.901141	257.01318	294.443995	350.752584	416.108328	165.6649036	NCKX6|NCLX	MIM:609841	12	12q24.13	solute carrier	protein-codir	SLC8B1	solute carrier	O	mitochondria	20210307
KRT1	ENSG00000167768	3848	35.50	-2.71	-3.17	0.001540261	0.032255672	0	0	0	5.67911781	0	86.0920361	1.91108283	1.02288633	2.45066677	4.8282082	1.8817016	1.7268041	10.2906004	416.9332244	12.7433694	22.44437746	CK1|EHK|EH	MIM:139350	12	12q13.13	keratin 1	protein-codir	KRT1	keratin 1	O	keratin, type	20210410
	ENSG00000286444		4.14	-3.94	-3.17	0.001548116	0.032351424	2.4249417	0	0	0	0	0	0	0.76389117	14.329175	21.2044038	3.41964727	8.02743147	4.26506079	3.916539049	2.97525972	4.924582546											
SNORD116-1	ENSG00000206621	100033426	11.19	-2.90	-3.17	0.001548232	0.032351424	24.4931119	19.0694812	0	0	0	0	4.47424457	1.12692916	27.4036372	30.3808655	10.1826398	14.5328308	6.54688906	4.316522656	14.4969574	22.05825215	HBII-85-14	HGNC:HGNC:	15	15q11.2	small nucleol	snoRNA	SNORD116-1	small nucleol	O	-	20210302
	ENSG00000269243		23.22	-2.50	-3.17	0.001550467	0.032353432	12.7564119	3.79798474	0	0	0	2.10061633	6.05203878	3.61854682	103.592155	192.866466	0.77371876	0	8.47646495	11.48284918	12.712788	13.36252049											
	ENSG00000263072		188.23	0.60	3.16	0.001551727	0.032353432	245.233766	203.295408	286.690882	219.441004	228.759421	270.066045	183.281799	169.026569	104.053613	99.726883	162.256501	155.106937	152.007001	235.6192644	171.290526	125.8817777											
GOLGA2P7	ENSG00000225151	388152	67.87	-1.44	-3.16	0.001553964	0.032364611	39.1675648	63.2743537	19.8640356	3.76668499	13.2679474	136.726197	18.4886725	21.2482918	299.761208	39.7216473	47.4653926	51.7273252	39.8312028	191.3061973	58.9744387	41.38825334	-	HGNC:HGNC:	15	15q25.2	GOLGA2 pseu	pseudo	GOLGA2P7	GOLGA2 pseu	O	golgin A2 pse	20210302
CAMK4	ENSG00000152495	814	2900.51	-1.16	-3.16	0.001572035	0.03270521	5775.77746	3616.35385	326.621092	594.316974	1127.85829	2984.34572	1219.19955	2053.75018	3629.11748	3682.02321	2811.46989	3918.20123	2455.2059	4325.491769	4833.20693	3055.158928	CaMK IV|CaM	MIM:114080	5	5q22.1	calcium/calm	protein-codir	CAMK4	calcium/calm	O	calcium/calm	20210302
NR1D1	ENSG00000126368	9572	138.02	-0.91	-3.16	0.001587442	0.032971918	111.338084	124.273738	201.374788	132.998372	16.3897662	78.349166	99.9495128	40.2731994	356.352042	174.1563	146.340463	222.910006	51.8950241	128.8631743	191.595544	131.292673	EAR1|REVERI	MIM:602408	17	17q21.1	nuclear recep	protein-codir	NR1D1	nuclear recep	O	nuclear recep	20210316
SCARNA10	ENSG00000239002	692148	3112.61	-0.88	-3.16	0.001588377	0.032971918	3984.40892	3670.66973	2642.38718	3008.70163	242.589201	1405.08941	1308.31736	1672.60822	3172.51753	3980.46028	5864.75039	9975.83812	1297.72135	1549.768181	2089.16193	3936.73659	U85	MIM:615639	12	12p13.31	small Cajal bo	ncRNA	SCARNA10	small Cajal bo	O	-	20210302
CEP250	ENSG00000126001	11190	659.75	0.80	3.16	0.001590052	0.032971918	665.86821	279.590724	1389.38533	1035.86889	927.349451	691.342865	1115.71599	682.651515	252.699809	465.542965	479.469524	403.689742	812.69648	395.2546135	529.060903	429.7926257	C-NAP1|CEP2	MIM:609689	20	20q11.22	centrosomal	protein-codir	CEP250	centrosomal	O	centrosome-a	20210404
NET1	ENSG00000173848	10276	513.56	-1.44	-3.16	0.001595765	0.03302777	588.41429	160.519238	191.415545	23.4529949	14.0111	57.1619002	12.5365926	53.9343675	6405.83806	68.9351462	100.973756	89.1049854	96.4642255	178.0512758	66.3455638	109.8692821	ARHGEF8|NE	MIM:606450	10	10p15.1	neuroepithel	protein-codir	NET1	neuroepithel	O	neuroepithel	20210302
NUDC	ENSG00000090273	10726	208.26	-0.83	-3.16	0.001596215	0.03302777	202.728579	197.912858	42.9121206	120.896121	91.3358601	185.460303	187.041809	158.6555	637.968832	270.654132	200.526047	234.825342	166.297832	200.6895419	226.093986	208.220207	HNUDC|MNU	MIM:610325	1	1p36.11	nuclear distri	protein-codir	NUDC	nuclear distri	O	nuclear migra	20210404
	ENSG00000281571		50.16	-1.39	-3.15	0.001613343	0.033165881	19.5070073	29.0271628	0	12.2841827	41.1933745	83.8904764	39.8497697	22.8430547	120.621852	123.248369	22.5000347	31.198015	46.3272921	78.28650019	75.6449481	56.11324121											
LOC10050733	ENSG00000228793	100507336	6.57	-3.69	-3.15	0.00163123	0.033389352	1.10419653	12.7334448	0	0	0	0.37331696	0	0.54325541	44.5651332	2.29523314	1.99052953	8.71473904	4.80228434	10.68571672	14.874275	2.503517064	-	Ensembl:ENS	6	6p25.2	uncharacteriz	ncRNA	-	-	-	-	20210302

	ENSG00000228274		5.81	-3.40	-3.15	0.001627058	0.033389352	0	1.17361182	0	0	0	0.77740728	7.28699681	2.14594159	12.0556397	22.8881804	5.66444529	5.187771	6.7501698	17.72940717	3.75161174	7.53374468											
	ENSG00000269968		35.12	-1.93	-3.15	0.001627804	0.033389352	32.4341809	11.4248754	0	1.77460481	8.64858895	2.30567293	2.40226926	5.44767156	286.382097	165.154533	0.90672787	1.35129457	9.87143991	8.339364082	9.3717377	16.02794485											
TRIM3	ENSG00000110171	10612	76.14	1.06	3.15	0.001631096	0.033389352	55.8814506	51.5537416	246.379291	62.9130934	62.4762383	78.8879709	193.442861	107.970478	47.1416429	52.8376057	50.2527907	27.0669955	54.9107356	33.59813768	50.5901721	42.32609908	BERP|HAC1|	MIM:605493	11	11p15.4	tripartite mo	protein-codin	TRIM3	tripartite mo	O	tripartite mo	20210417
PTPN20	ENSG00000204179	26095	4.77	-4.15	-3.15	0.001636021	0.033415551	0	0	3.25100239	0	0	0	0	1.10134512	5.699143	12.9929642	3.38676212	15.0214584	1.39967299	25.85135798	4.58875983	2.965575023	CT126|PTPN	MIM:610630	10	10q11.22	protein tyros	protein-codin	PTPN20	protein tyros	O	tyrosine-prot	20210403
PARP15	ENSG00000173200	165631	680.62	0.76	3.15	0.001635614	0.033415551	550.740175	552.218709	341.187813	648.681701	1482.83791	883.996387	1212.3258	1267.77067	239.168287	486.411129	423.75013	366.131115	716.826187	752.3796891	636.191892	329.273783	ARTD7|BAL3	MIM:612066	3	3q21.1	poly(ADP-rib	protein-codin	PARP15	poly(ADP-rib	O	protein mon	20210302
	ENSG00000280537		7.98	-4.07	-3.15	0.001646211	0.033515797	16.1277006	0	0	0	0	0	0	2.47326399	6.0073634	1.60358897	8.44742135	16.3283481	3.84170355	42.73882095	17.6340465	12.41057848											
CDKN2B-AS1	ENSG00000240498	100048912	77.41	1.20	3.15	0.001645472	0.033515797	169.863091	54.1565503	215.635455	156.662412	147.754766	46.6634379	53.3375274	46.7376837	42.7584029	58.6835248	42.4400604	29.4379535	72.8805635	37.07809726	16.9857357	47.53876586	ANRIL|CDKN	MIM:613149	9	9p21.3	CDKN2B anti	ncRNA	CDKN2B-AS1	CDKN2B anti	O	CDKN2B anti	20210418
TALDO1	ENSG00000177156	6888	212.34	-0.77	-3.15	0.001649477	0.033546424	341.474057	190.09123	88.6327574	56.2072394	99.4461212	114.086762	168.831713	112.378117	959.538303	230.916942	134.858598	174.847384	226.868854	193.6077674	151.421357	154.2178524	TAL|TAL-H|T	MIM:602063	11	11p15.5	transaldolase	protein-codin	TALDO1	transaldolase	O	transaldolase	20210404
SNRPD2	ENSG00000125743	6633	208.58	-0.78	-3.15	0.001653509	0.033560675	309.111934	187.875268	67.4996912	173.860739	91.3164025	106.27526	127.127149	176.547381	393.684505	205.112952	344.365249	328.55404	166.965705	168.1679042	161.181633	329.6908048	SMD2|SNRP	MIM:601061	19	19q13.32	small nuclear	protein-codin	SNRPD2	small nuclear	O	small nuclear	20210404
LTBP4	ENSG00000090006	8425	352.85	0.91	3.15	0.001653704	0.033560675	396.944301	273.639349	223.46392	384.803569	534.569865	249.101499	1124.10917	571.798934	262.371383	316.467098	127.688566	117.126161	390.436867	240.5610312	258.287597	174.3082677	ARCL1C|LTBP	MIM:604710	19	19q13.2	latent transf	protein-codin	LTBP4	latent transf	O	latent-transf	20210410
IGLC2	ENSG00000211677	3538	9.65	-3.82	-3.15	0.00165863	0.033589028	0	6.05205438	6.28986965	0	0	0	1.91900541	0.57683215	48.4317866	0.72103186	4.27285269	5.89771494	44.2952101	17.81046597	10.0792617	8.031045867	IGLC	HGNC:HGNC:	22	22q11.22	immunoglob	other	IGLC2	immunoglob	O	C2 segment|	20210302
	ENSG00000250182		50.76	-1.08	-3.15	0.001658336	0.033589028	17.6316848	19.3660421	3.32834588	3.40574565	42.3962417	52.7691627	55.423501	61.2378454	18.4811379	28.5823757	15.5473077	17.4848447	70.9166397	138.0040111	102.7639	164.7819136											
CDC7	ENSG00000097046	8317	108.50	0.81	3.14	0.001666243	0.033707335	115.962257	260.338825	316.59661	109.045878	104.501264	72.9727113	119.659346	46.1471447	91.0802111	78.9002613	135.661604	81.2024396	41.5326306	55.72049447	62.1935641	44.42584979	CDC7L1|HsC	MIM:603311	1	1p22.2-p22.1	cell division c	protein-codin	CDC7	cell division c	O	cell division c	20210302
HIVEP2	ENSG00000010818	3097	1698.61	-0.80	-3.14	0.00167939	0.033901234	1553.31034	1428.63164	1789.76738	879.768208	518.726817	2070.84356	836.104978	1107.19364	1548.37584	1685.21933	2780.40246	3447.85987	1803.3463	1940.55907	2089.48496	1698.099594	HIV-EP2|MB	MIM:143054	6	6q24.2	HIVEP zinc fir	protein-codin	HIVEP2	HIVEP zinc fir	O	transcription	20210302
ZNF395	ENSG00000186918	55893	212.95	-1.03	-3.14	0.001682569	0.033929437	147.995781	135.506554	24.4403971	133.237417	136.960227	314.113309	115.372515	114.833815	318.784763	212.053373	267.016681	516.156645	244.375134	194.6360779	359.896787	171.8678194	HDBP-2|HDB	MIM:609494	8	8p21.1	zinc finger pr	protein-codin	ZNF395	zinc finger pr	O	zinc finger pr	20210302
DAPP1	ENSG00000070190	27071	106.53	-1.66	-3.14	0.001716708	0.034516875	124.623645	101.229093	0	13.7499908	35.9936233	118.817853	3.09598616	59.4438188	720.798008	81.4632763	58.1558768	64.735028	85.1568118	128.4909145	42.4547471	66.29308976	BAM32	MIM:605768	4	4q23	dual adaptor	protein-codin	DAPP1	dual adaptor	O	dual adapter	20210302
PMAIP1	ENSG00000141682	5366	83.27	-0.93	-3.14	0.00171714	0.034516875	109.829703	83.7879667	30.2617084	29.4026213	6.89231655	84.6250757	74.9865238	57.56223	235.044415	109.64958	71.7971311	101.257959	39.3435684	103.7297673	98.1201275	95.97026414	APR|NOXA	MIM:604959	18	18q21.32	phorbol-12-m	protein-codin	PMAIP1	phorbol-12-m	O	phorbol-12-m	20210406
POLR2J3-UPK	ENSG00000168255	123258399	223.41	-0.60	-3.14	0.001715908	0.034516875	171.763366	164.685246	176.566601	122.819748	170.759802	279.450992	140.561826	183.776327	233.931278	300.56998	326.044091	240.914604	389.260218	287.444624	162.95109	223.1100739											
FCGR3A	ENSG00000203747	2214	732.34	1.01	3.13	0.001722779	0.03455723	93.2843932	375.08575	1053.64457	763.544852	1237.42128	1051.36189	1686.93569	1694.17486	167.213444	334.680124	328.211704	247.525346	959.167839	235.9503835	388.365768	1100.889141	CD16|CD16A	MIM:146740	1	1q23.3	Fc fragment c	protein-codin	FCGR3A	Fc fragment c	O	low affinity ir	20210418
SLA	ENSG00000155926	6503	1058.36	0.58	3.13	0.001727162	0.034608698	318.043944	727.352644	1409.04656	1604.68642	2015.76442	1244.00729	1637.39742	1372.52884	417.990113	479.099853	657.915091	885.595165	1396.85289	899.4461503	733.019267	1134.966071	SLA1|SLAP	MIM:601099	8	8q24.22	Src like adapt	protein-codin	SLA	Src like adapt	O	src-like-adapt	20210302
TRBV21-1	ENSG00000251578	28566	7.80	-3.78	-3.13	0.001738308	0.0347954	1.12459466	22.7801468	0	0	0	3.53688862	0	0	8.71490392	23.8968853	22.1191509	10.92481	3.54602316	15.97032052	5.50405817	6.684578212	TCRBV10S1P	HGNC:HGNC:	7	7q34	T cell recepto	pseudo	TRBV21-1	T cell recepto	O	-	20210302
SEPSECS	ENSG00000109618	51091	323.52	0.68	3.13	0.001743511	0.034826311	428.002328	411.363935	307.291033	653.632873	517.340754	343.110337	321.552045	223.224616	128.17061	257.928859	307.653038	350.277463	252.388462	255.7638865	197.68161	220.9058022	LP|PCH2D|S	MIM:613009	4	4p15.2	Sep (O-phosp	protein-codin	SEPSECS	Sep (O-phosp	O	O-phosphose	20210302
	ENSG00000281852		34.11	-2.09	-3.13	0.001745458	0.03482865	86.135066	20.1078415	0	8.35619507	2.36823475	22.1983363	10.3487651	5.72074076	28.1705808	19.3299997	29.00764	80.5184987	61.3453265	65.29314079	88.1551671	18.65130614											
MYO1F	ENSG00000142347	4542	1495.64	0.71	3.13	0.001748423	0.034851281	918.834872	1127.1602	808.462399	2350.34383	2986.31013	1737.38662	2860.65332	2321.01225	512.65095	1034.60504	1185.69314	827.759224	1836.19494	1171.94741	1093.8621	1157.436258	-	MIM:601480	19	19p13.2	myosin IF	protein-codin	MYO1F	myosin IF	O	unconventior	20210302
AGMAT	ENSG00000116771	79814	18.30	-2.60	-3.13	0.001750637	0.034858925	44.3478585	22.8470468	12.3739789	0	0	3.4482158	0.70076236	2.58033775	44.5944046	19.9766535	9.00410967	25.0765248	27.3072971	30.40558438	27.5350956	22.60174425	-	MIM:617887	1	1p36.21	agmatinase	protein-codin	AGMAT	agmatinase	O	agmatinase,	20210302
	ENSG00000249209		5.82	-4.18	-3.13	0.001753685	0.034883116	2.51266342	15.9896836	0	0	0	0	0	0	11.7029071	9.93397096	1.49123877	5.96487047	14.1183224	12.21120262	8.58104228	10.54058999											
MYO10	ENSG00000145555	4651	44.58	3.24	3.13	0.001765151	0.035074539	8.26092264	1.89657171	62.5375434	24.6995106	19.7594778	3.42441847	1.84238189	8.22704836	566.967476	0	4.65087775	0.856608	10.2024012	0	0	0	-	MIM:601481	5	5p15.1	myosin X	protein-codin	MYO10	myosin X	O	unconventior	20210406
GPR146	ENSG00000164849	115330	9.52	-2.74	-3.13	0.00177326	0.035198937	0.30781403	1.68401531	1.58699585	6.17504779	0	10.7004258	0	7.40571648	17.451843	2.58704875	19.0336954	11.6161883	7.76663802	40.78191574	14.2587865	10.95152836	PGR8	HGNC:HGNC:	7	7p22.3	G protein-co	protein-codin	GPR146	G protein-co	O	probable G-p	20210302
	ENSG00000286970		8.44	-4.23	-3.13	0.001776938	0.035235195	0	0	0	0	20.880576	0	0	4.14816409	35.2131082	40.340643	3.74767314	2.11273243	6.1364838	8.803987251	9.48968363	4.107951278											
SMC4	ENSG00000113810	10051	867.80	0.68	3.12	0.001782974	0.035318104	788.138334	979.031594	2916.10332	1028.75276	690.139128	822.43465	913.052727	677.669907	1055.86275	574.076346	857.110861	503.153861	570.790066	438.4598963	616.760712	453.2053445	CAP-C|CAPC|	MIM:605575	3	3q25.33	structural ma	protein-codin	SMC4	structural ma	O	structural ma	20210404
PRDM1	ENSG00000057657	639	1012.01	0.72	3.12	0.001784918	0.035319861	656.365006	726.281395	1303.60411	1283.63481	877.049924	1444.173	1612.12266	2150.05871	1099.19606	473.023697	512.901343	626.852479	610.136221	851.8865307	570.445076	1394.451557	BLIMP1|PRD	MIM:603423	6	6q21	PR/SET doma	protein-codin	PRDM1	PR/SET doma	O	PR domain zi	20210322
TMEM123	ENSG00000152558	114908	2435.52	-0.92	-3.12	0.001795823	0.035461915	4099.98229	3479.9411	1622.07751	860.352539	525.277904	2384.83711	936.187929	1177.06784	3168.60167	3080.61727	2870.1482	4565.5382	1710.88597	3067.622782	3241.33632	2177.832809	KCT3|PORIM	MIM:606356	11	11q22.2	transmembra	protein-codin	TMEM123	transmembra	O	porimin|KCT-	20210417
PLK3	ENSG00000173846	1263	245.23	-1.02	-3.12	0.001807627	0.035658022	451.934015	525.043741	131.626113	73.2018875	70.9510931	132.921069	70.6668816	105.787847	338.725768	434.852998	225.082916	313.224559	255.928592	305.5436673	299.802648	188.3080829	CNK|FNK|PL	MIM:602913	1	1p34.1	polo like kina	protein-codin	PLK3	polo like kina	O	serine/threor	20210406
	ENSG00000263535		4.61	-4.02	-3.12	0.001823878	0.035941346	4.72641749	0	0	0	0	0	0	3.4768567	4.67106053	5.30432035	10.205648	10.3122182	9.84536801	9.025701598	6.44505237	9.791587703											
NKX3-1	ENSG00000167034	4824	31.63	-1.74	-3.11	0.001847493	0.036369058	37.1019778	12.4681526	0	6.04347638	13.0026924	3.73766856	6.66768255	2.79700977	297.541767	25.1508169	11.0593429	21.1046621	8.99206696	19.71823013	36.8533952	3.806908345	BAPX2|NKX3	MIM:602041	8	8p21.2	NK3 homeob	protein-codin	NKX3-1	NK3 homeob	O	homeobox pr	20210302
	ENSG00000272109		6.97	-3.40	-3.11	0.001853738	0.0364543	0	1.55665497	0	0	0	0	1.26417763	1.73548612	31.4499566	47.1652199	1.47082249	2.06493424	6.61633642	1.342627848	2.042824	14.83744836											
IGHA1	ENSG00000211895	3493	15.75	-4.42	-3.11	0.001858794	0.036516	1.20956957	1.02997447	6.29416654	0	0	0	0	0	106.113688	12.7289423	0	1.54937597	104.059191	0	8.37508297	10.58177973	IgA1	MIM:146900	14	14q32.33	immunoglob	other	IGHA1	immunoglob	O	immunoglob	20210302
PYCR3	ENSG00000104524	65263	12.01	-3.03	-3.11	0.001863072	0.036524648	0	22.6365555	0	10.5939996	0	0	5.82530905	5.60969818	16.6251501	14.6937682	18.6009093	8.47473555	21.9560884	11.014549	17.9794058	38.16822537	PYCRL	MIM:616408	8	8q24.3	pyrroline-5-c	protein-codin	PYCR3	pyrroline-5-c	O	pyrroline-5-c	20210302
PLEKHO1	ENSG00000023902	51177	450.02	-0.64	-3.11	0.001867599	0.036575732	334.898869	644.042814	378.707351	217.055129	183.610717	338.564607	359.153119	388.479613	384.151211	808.10726	403.938538	359.383598	633.304368	539.5746831	718.28198	509.0076224	CKIP-1|CKIP1	MIM:608335	1	1q21.2	pleckstrin ho	protein-codin	PLEKHO1	pleckstrin ho	O	pleckstrin ho	20210302
ADAM19	ENSG00000135074	8728	44.23	-1.47	-3.11	0.001870892	0.036602576	0	28.5850801	7.42216952	15.6613157	24.3015921	44.341248	17.412443	68.7079472	11.0250822	41.7501838	44.0351271	23.8447309	104.752213	168.8048166	53.9340112	53.13220587	FKSG34|MAD	MIM:603640	5	5q33.3	ADAM metall	protein-codin	ADAM19	ADAM metall	O	disintegrin ar	20210302
PRXL2B	ENSG00000157870	127281	69.00	-1.53	-3.11	0.001876835	0.036681141	76.8472913	74.0544281	0	7.35830341	46.6111795	74.593042	15.4333736	29.0493688	298.57773	71.8316567	50.7612234	81.2465612	52.4164167	105.5447578	65.8197505	53.84050724	C1orf93|FAM	HGNC:HGNC:	1	1p36.32	peroxiredoxi	protein-codin	PRXL2B	peroxiredoxi	O	prostamide/p	20210302
KCNQ3	ENSG00000184156	3786	5.93	-3.76	-3.11	0.001892854	0.036918408	7.96584695	4.00316324	0	0	0	0	0	0.82792998	8.71085603	2.64040466	3.91895406	4.14561108	19.716497	9.039477348	8.56130196	25.41666232	BFNC2|EBN2	MIM:602232	8	8q24.22	potassium vo	protein-codin	KCNQ3	potassium vo	O	potassium vo	20210410
	ENSG00000234332		7.43	-3.02	-3.11	0.001895389	0.036930009	0	3.25916786	0	0	0	7.86467534	2.50031504	0.55854362	33.4475784	29.6100732	10.3254383	3.63188369	3.9766679	14.49523602	5.67434078	3.598275942											
GYPC	ENSG00000136732	2995	393.88	-0.65	-3.11	0.001899952	0.036981076	783.685385	450.202171	252.840055	239.760051	183.68411	243.678146	261.066738	196.347557	576.906409	547.879966	377.626267	588.446663	333.269176	405.005666	466.505122	395.124452	CD236|CD23	MIM:110750	2	2q14.3	glycophorin	protein-codin	GYPC	glycophorin	O	glycophorin-	20210302
	ENSG00000263823		3.81	-3.63	-3.10	0.001913131	0.037199558	0	0.9088256	0	1.6293663	0	0	0	1.56262781	5.18886645	16.3636372	1.41008859	3.09614937	3.4116159	5.810700488	7.96264822	13.54325175											
ZNF415	ENSG00000170954	55786	10.71	-1.98	-3.10	0.00191704	0.037237519	16.0976318	2.00704862	0	1.07370779	7.93402572	0.94229653	3.93003241	3.82831033	56.2854581	11.2044852	19.2308982	6.62087893	11.2753264	3.919274124	20.8565436	6.205656618	Pact|ZfLp	HGNC:HGNC:	19	19q13.42	zinc finger pr	protein-codin	ZNF415	zinc finger pr	O	zinc finger pr	20210302
TMTC2	ENSG00000179104	160335	76.88	-1.64	-3.10	0.001924829	0.037350717	74.2591617	42.3150808	0	6.91965358	5.88498579	54.3626491	44.6104333	21.7783011	618.54007	70.699315	49.0438264	39.5899878	39.3908392	86.72251514	61.3022706	14.65474848	IBDBP1	MIM:615856	12	12q21.31	transmembra	protein-codin	TMTC2	transmembra	O	protein O-ma	20210302
SMYD5	ENSG00000135632	10322	75.88	-1.05	-3.10	0.001937522	0.037553074	68.5610423	75.1218304	53.8020608	20.1531191	24.2653741	102.185165	36.3089431	34.5163835	93.2112381	89.609026	99.796758	114.183757	83.7383585	134.0839814	135.85933	48.62741347	NN8-4AG|RA	MIM:619114	2	2p13.2	SMYD family	protein-codin	SMYD5	SMYD family	O	SET and MYN	20210302
TBKBP1	ENSG00000198933	9755	168.95	0.95	3.10	0.001939203	0.037553074	72.7836532	98.5407116	580.907329	240.803912	241.247607	190.892005	197.197577	203.536908	41.5524029	143.828028	112.465845	113.572224	212.435794	78.1561666	91.3590296	83.99286507	ProSAPiP2|S	MIM:608476	17	17q21.32	TBK1 binding	protein-codin	TBKBP1	TBK1 binding	O	TANK-binding	20210307
PEX6	ENSG00000124587	5190	84.94	-0.75	-3.10	0.001943248	0.037593167	57.6045924	40.3100624	34.1381826	31.4452716	56.3408684	89.4100306	91.8859351	103.925787	77.6505843	109.680888	61.5126649	38.779699	139.254124	146.8130574	112.107589	168.1226104	HMLR2|PAF-	MIM:601498	6	6p21.1	peroxisomal	protein-codin	PEX6	peroxisomal	O	peroxisome b	20210410
ANKRD49	ENSG00000168876	54851	506.93	0.66	3.10	0.001946996	0.037627436	748.337553	739.8986	1091.87376	552.399529	563.990523	386.422637	499.269898	394.305595	198.970086	445.704841	625.780291	487.341024	369.295751	393.1877031	371.796072	242.365015	FGIF|GBIF	HGNC:HGNC:	11	11q21	ankyrin repea	protein-codin	ANKRD49	ankyrin repea	O	ankyrin repea	20210302
TNFRSF25	ENSG00000215788	8718	314.05	-1.57	-3.10	0.00196176	0.037759416	357.880085	118.488002	0	87.0009603	172.588829	429.045907	208.499633	88.7568904	160.498472	335.884806	274.531874	223.897191	675.406022	687.0487543	1003.93827	201.3658609	APO-3|DDR3	MIM:603366	1	1p36.31	TNF receptor	protein-codin	TNFRSF25	TNF receptor	O	tumor necros	20210418
FAM153CP	ENSG00000204677	653316	90.03	-1.14	-3.10	0.001961344	0.037759416	55.2338707	179.044828	21.5927448	17.6938513	27.6931864	81.1778324	48.8077455	56.0139164	45.0027285	123.764962	51.1137876	66.6598297	113.044227	241.2871255	180.608685	131.7523638	FAM153C|N	HGNC:HGNC:	5	5q35.3	protein FAM	pseudo	FAM153CP	family with s	O	family with s	20210302
ZNF566	ENSG00000186017	84924	127.00	-1.00	-3.10	0.001958499	0.037759416	16.6671271	97.4656837	203.080082	84.1980361	53.1879396	65.4010796	105.114688	101.937075	62.0397131	241.441636	130.746156	245.555379	200.2016	146.5389181	160.137994	118.3407106	-	HGNC:HGNC:	19	19q13.12	zinc finger pr	protein-codin	ZNF566	zinc finger pr	O	zinc finger pr	20210302
	ENSG00000269987		8.90	-3.55	-3.09	0.001975385	0.03798327	4.13674136	0	0	0	0	0	0	1.11982261	57.5867661	62.9400869	1.96439798	0.85877824	2.05982197	2.476924272	6.53962821	2.786154576											
ZBTB18	ENSG00000179456	10472	504.08	-0.88	-3.09	0.001992755	0.038261831	871.673233	498.875957	373.964488	273.114267	284.037777	427.78623	183.835831	155.568105	614.310722	589.263379	456.553037	723.769347	531.400536	727.9387912	806.640217	546.5305342	C2H2-171|M	MIM:608433	1	1q44	zinc finger an	protein-codin	ZBTB18	zinc finger an	O	zinc finger an	20210302
SNORA2C	ENSG00000221491	677815	10.39	-2.15	-3.09	0.002017263	0.038632403	2.11381136	9.29741325	0	0	2.27158349	4.15530579	2.32207857	13.7496453	11.9969482	24.6831761	3.90571117	0.81524067	12.0039839	15.77168359	30.6540189	32.54896674	ACA34|MIR1	MIM:615487	12	12q13.11	small nucleol	snoRNA	SNORA2C	small nucleol	O	ACA34 snoRN	20210302
LOC1006529	ENSG00000257550	100652999	30.68	-2.09	-3.09	0.002016808	0.038632403	18.2154368	22.9122357	0	0	0	2.38944335	8.29018289	2.36348571	341.773206	56.1585727	1.42688023	1.33120891	6.56845515	9.387659711	8.6324982	11.49192744	-	Ensembl:ENS	12	12q13.13	uncharacteri	ncRNA	-	-	-	-	20210302
	ENSG00000254760		12.40	2.00	3.09	0.002020002	0.038645995	11.3092643	11.8815734	0	6.93331351	60.5175412	12.0913779	50.0589306	7.8902621	2.18516959	1.35922444	2.75201096	1.69703022	16.1996549	6.784121212	5.94053475	0.848881273											
PTK2	ENSG00000169398	5747	74.52	-2.19	-3.09	0.002025733	0.0386463	199.889686	55.1950303	42.5336434	1.51227364	17.0114346	6.21521509	3.00479523	0	604.186324	18.0650374	22.5713969	21.9779128	70.2556383	71.96478478	22.5014856	35.45032351	FADK|FADK 1	MIM:600758	8	8q24.3	protein tyros	protein-codin	PTK2	protein tyros	O	focal adhesio	20210417
CXCR6	ENSG00000172215	10663	193.68	-0.98	-3.09	0.002026428	0.0386463	173.024373	164.55831	60.5428794	146.62132	96.9193471	92.2993113	142.916835	103.588178	164.919248	211.902376	278.841788	250.041133	111.462008	130.4039295	462.255422	508.5732439	BONZO|CD1	MIM:605163	3	3p21.31	C-X-C motif c	protein-codin	CXCR6	C-X-C motif c	O	C-X-C chemol	20210418
ATR	ENSG00000175054	545	1141.17	0.62	3.09	0.002028139	0.0386463	663.365656	1072.21917	3119.57528	1763.65063	1656.94816	1197.53614	937.815189	1061.79244	658.205836	750.874865	885.87743	1072.86588	964.892814	831.0689926	910.408032	711.6366734	FCTCS|FRP1|	MIM:601215	3	3q23	ATR serine/th	protein-codin	ATR	ATR serine/th	O	serine/threor	20210418
BLTP1	ENSG00000138688	84162	4483.36	0.67	3.08	0.002042633	0.038883564	3915.38301	4547.05947	2749.6352	6403.86378	8104.23279	7616.97435	5362.74881	5378.85167	1753.03119	3302.98044	4817.0352	3374.43766	4581.08062	3250.494319	3799.96079	2775.965972											
CCDC39	ENSG00000284862	339829	20.80	-2.77	-3.08	0.002046173	0.038912033	5.35094835	8.29928689	0	0	2.02009916	0	0	2.26301378	153.927819	146.491862	1.56773278	0.56539915	1.39243121	6.297972526	1.7063605	2.98073687	CFAP59|CILD	MIM:613798	3	3q26.33	coiled-coil do	protein-codin	CCDC39	coiled-coil do	O	coiled-coil do	20210410
NUTF2	ENSG00000102898	10204	137.03	-0.86	-3.08	0.002054962	0.039040184	157.749922	106.570413	107.785951	78.2422802	103.921305	47.4467571	87.1101354	105.04454	142.217748	180.846532	135.200646	219.31191	124.579622	200.7657176	193.722446	202.0210956	NTF-2|NTF2|	MIM:605813	16	16q22.1	nuclear trans	protein-codin	NUTF2	nuclear trans	O	nuclear trans	20210302
CD28	ENSG00000178562	940	911.62	-2.74	-3.08	0.002067223	0.039194876	1736.72236	1557.65113	0	149.191063	6.43477668	1060.15216	263.645597	67.8691784	844.14222	1308.9353	1077.11071	1553.41066	500.031424	2196.620607	1568.93287	695.1320372	Tp44	MIM:186760	2	2q33.2	CD28 molecu	protein-codin	CD28	CD28 molecu	O	T-cell-specifi	20210418
	ENSG00000283189		35.31	-1.91	-3.08	0.002066037	0.039194876	5.87722059	11.389498	0	12.1986378	7.15483302	7.53681315	36.2157429	76.1192056	20.4197677	100.608109	67.7516197	22.9118063	25.9864233	60.84937362	82.9634373	26.92979222											
GIMAP1	ENSG00000213203	170575	768.66	-0.68	-3.08	0.002073122	0.039249987	1149.86312	746.17148	356.986518	540.676458	497.754373	731.979096	473.341603	395.457026	733.753663	831.310367	852.178405	859.356845	966.532041	1396.02741	953.181661	814.0305558	HIMAP1|IAN	MIM:608084	7	7q36.1	GTPase, IMA	protein-codin	GIMAP1	GTPase, IMA	O	GTPase IMAP	20210302
AHRR	ENSG00000063438	57491	31.96	-1.95	-3.08	0.002092242	0.039433589	48.2064977	32.9422713	0	0	4.45841665	14.8273099	37.7767985	6.50211129	37.1999191	78.6311977	11.607066	39.6126836	67.3716536	50.08132712	63.8542916	18.28766984	AHH|AHRR|	MIM:606517	5	5p15.33	aryl-hydrocar	protein-codin	AHRR	aryl-hydrocar	O	aryl hydrocar	20210313
SEH1L	ENSG00000085415	81929	234.37	-1.00	-3.08	0.002090609	0.039433589	411.706933	268.061351	37.7287493	91.5310237	69.416586	211.614332	173.282808	143.063507	332.37587	256.157213	277.881286	344.606583	195.554408	325.5950987	350.626845	260.6457968	SEC13L|SEH1	MIM:609263	18	18p11.21	SEH1 like nuc	protein-codin	SEH1L	SEH1 like nuc	O	nucleoporin	20210302
CANT1	ENSG00000171302	124583	249.57	-0.81	-3.08	0.002087557	0.039433589	132.344385	78.7524114	125.651176	234.464435	130.529447	258.024067	243.254074	235.92122	551.0453	229.687044	156.245637	204.895367	243.118337	317.6635705	483.134698	368.3880453	DBQD|DBQD	MIM:613165	17	17q25.3	calcium activ	protein-codin	CANT1	calcium activ	O	soluble calciu	20210324
ZBTB12	ENSG00000204366	221527	7.88	-2.65	-3.08	0.002100696	0.039514667	9.27489083	1.81663405	0	3.32135543	0	0	1.66939486	2.24056598	36.6083994	21.366095	1.05109265	4.51501925	7.58823773	15.35997413	14.7185552	6.607204669	Bat9|C6orf4	HGNC:HGNC:	6	6p21.33	zinc finger an	protein-codin	ZBTB12	zinc finger an	O	zinc finger an	20210302
AK5	ENSG00000154027	26289	70.63	-3.29	-3.07	0.002107122	0.039518404	386.808463	176.324491	0	0	0	88.589422	0	2.52974973	76.7526134	59.8620221	27.5969369	104.21575	18.6473125	70.13558703	100.879626	17.69639447	AK6	MIM:608009	1	1p31.1	adenylate kin	protein-codin	AK5	adenylate kin	O	adenylate kin	20210302
MAST4	ENSG00000069020	375449	250.33	-1.35	-3.08	0.002103138	0.039518404	354.934934	326.311359	44.9883906	41.538605	49.668709	458.797274	18.5158241	84.8842682	1023.22987	259.930477	174.538052	205.830403	149.741228	362.2431988	344.93377	105.2392656	-	MIM:618002	5	5q12.3	microtubule	protein-codin	MAST4	microtubule	O	microtubule-	20210302
TRBV27	ENSG00000211752	28560	120.60	-1.66	-3.07	0.002109633	0.03952654	116.299164	125.127915	0	157.369391	11.0252395	26.6860231	24.3464184	104.79677	46.5257658	90.6552509	87.9980171	390.996107	80.3349596	97.38668465	117.648569	452.3780567	TCRBV14S1|	HGNC:HGNC:	7	7q34	T cell recepto	other	TRBV27	T cell recepto	O	V_segment tr	20210302
	ENSG00000263917		15.34	-4.34	-3.07	0.002122484	0.039728226	0	0	0	0	0	29.3340278	32.7989454	4.80560486	18.7810628	31.6239348	24.1124799	40.273001	31.1732275	16.24620198	16.2913708	0											
SNORA8	ENSG00000207304	654320	223.53	-1.84	-3.07	0.002126337	0.039761257	154.695495	145.640567	0	0	29.9380591	226.124604	201.196501	321.386767	79.1930469	124.944023	50.9030348	54.4621956	382.803815	449.5831327	476.991244	878.5767966	ACA8	HGNC:HGNC:	11	11q21	small nucleol	snoRNA	SNORA8	small nucleol	O	ACA8 small n	20210302
DUSP4	ENSG00000120875	1846	66.37	-1.22	-3.07	0.002136924	0.039880875	19.9975008	128.490558	2.93673981	34.9614221	107.323254	1.67549363	19.5848269	56.5684922	21.4473207	151.969378	33.5782566	67.1237584	229.72597	19.21883137	45.7710221	121.4784789	HVH2|MKP-2	MIM:602747	8	8p12	dual specifici	protein-codin	DUSP4	dual specifici	O	dual specifici	20210302
SINHCAF	ENSG00000139146	58516	398.69	-0.71	-3.07	0.002151351	0.040110791	318.723852	310.71663	461.952122	217.37778	109.072421	428.928311	313.233131	284.424665	735.268836	383.244089	439.592707	693.721256	242.137441	424.8158704	618.397593	397.3895854	C12orf14|FA	MIM:615027	12	12p11.21	SIN3-HDAC c	protein-codin	SINHCAF	SIN3-HDAC c	O	SIN3-HDAC c	20210302
TWF2	ENSG00000247596	11344	162.08	-0.66	-3.07	0.002158608	0.040206714	112.025451	131.554373	59.8236014	121.535895	136.981627	79.0524428	194.445198	182.5608	130.258874	175.197997	152.792043	146.263546	228.648904	215.2121298	250.615037	276.3683227	A6RP|A6r|M	MIM:607433	3	3p21.2	twinfilin acti	protein-codin	TWF2	twinfilin acti	O	twinfilin-2|A	20210302
ABCA5	ENSG00000154265	23461	570.82	0.81	3.06	0.002205081	0.040992104	448.495824	328.476538	426.101424	1035.51169	1184.45245	716.031986	811.020289	912.455396	230.485878	364.15025	390.351353	264.728893	508.701366	580.6027839	662.753001	268.8635079	ABC13|EST9	MIM:612503	17	17q24.3	ATP binding c	protein-codin	ABCA5	ATP binding c	O	cholesterol tr	20210410
CCDC136	ENSG00000128596	64753	82.65	1.05	3.06	0.002211166	0.041065137	126.848431	45.7206518	96.1132614	63.8174935	173.906284	138.641961	134.019746	107.953648	23.116323	68.0328234	38.3948816	41.9962327	113.720033	37.99802223	48.2077447	63.9243982	NAG6	MIM:611902	7	7q32.1	coiled-coil do	protein-codin	CCDC136	coiled-coil do	O	coiled-coil do	20210302
	ENSG00000262633		6.00	-3.57	-3.06	0.002235634	0.04143868	0	0	0	2.29271065	0	0	17.6822186	0.3787254	5.94024489	2.76109663	1.89345894	27.2088785	6.45745039	6.076759008	9.28915769	16.08431963											
NME9	ENSG00000181322	347736	4.90	-3.41	-3.06	0.002244878	0.041569554	0	1.71526622	0	0	0	2.92917012	0.36092143	1.30414328	11.0941673	15.3650527	8.52628971	4.31084089	10.1100006	3.080339533	18.8037628	0.838541307	NM23-H9|N	MIM:618584	3	3q22.3	NME/NM23 f	protein-codin	NME9	NME/NM23 f	O	thioredoxin d	20210302
RGS1	ENSG00000090104	5996	115.06	-1.17	-3.06	0.002248414	0.041594566	152.462892	271.163889	10.3920818	38.1417606	39.175845	48.9010904	62.6674648	23.5236556	129.4756	269.881899	274.992569	121.799143	101.248063	127.3845666	103.930855	65.86374962	1R20|BL34|H	MIM:600323	1	1q31.2	regulator of	protein-codin	RGS1	regulator of	O	regulator of	20210302
RBKS	ENSG00000171174	64080	11.85	-2.10	-3.05	0.00229102	0.042300532	11.3185308	1.5829442	0	8.34106785	1.32172197	2.7324635	5.95046582	2.37317231	75.6089009	10.2463114	8.92814003	16.3527566	5.39833299	17.4532036	4.50167895	17.48037016	RBSK|RK	MIM:611132	2	2p23.2	ribokinase	protein-codin	RBKS	ribokinase	O	ribokinase	20210302
PTGFRN	ENSG00000134247	5738	33.68	-3.03	-3.05	0.002301911	0.042378313	3.46382121	1.09972845	0	0	2.18897473	0	0	1.11406729	494.944699	3.37163185	4.25002666	0	21.4525533	3.487748145	2.77199794	0.781141905	CD315|CD9P	MIM:601204	1	1p13.1	prostaglandi	protein-codin	PTGFRN	prostaglandi	O	prostaglandi	20210302
HIVEP1	ENSG00000095951	3096	741.70	-0.88	-3.05	0.002300554	0.042378313	1420.76276	604.582113	354.06437	926.908606	98.1686155	438.039154	411.087691	380.230369	968.91337	871.667149	1352.83951	1108.55795	417.863044	809.0670325	1215.99506	488.403931	CIRIP|CRYBP	MIM:194540	6	6p24.1	HIVEP zinc fir	protein-codin	HIVEP1	HIVEP zinc fir	O	zinc finger pr	20210302
LOC1027244	ENSG00000275993	102724428	14.66	-3.04	-3.05	0.002311368	0.042511298	11.9026255	0	0	22.4830394	0	19.3666785	0	4.75506293	31.3895557	17.3524109	23.9616699	12.4023463	34.1276924	15.39464143	29.5979953	11.85627968											
H2AJ	ENSG00000246705	55766	75.03	-0.99	-3.05	0.00232276	0.042679594	30.4909953	53.0528808	28.6541817	56.2056268	40.1240451	34.4731881	87.5726922	76.2382129	169.691722	91.8369957	70.2530063	92.7126529	103.500182	79.13667095	78.8145539	107.6529228	H2AFJ	HGNC:HGNC:	12	12p12.3	H2A.J histone	protein-codin	H2AJ	H2A.J histone	O	histone H2A.	20210302
RPS13	ENSG00000110700	6207	4408.12	-0.67	-3.05	0.002325416	0.042687196	4459.63035	3821.08255	5412.44999	3217.56274	1369.67387	3717.24046	2459.31327	2351.29255	10685.9404	2793.68414	5394.55474	8465.06997	2604.13366	4407.808213	4947.36306	4423.170549	S13	MIM:180476	11	11p15.1	ribosomal pr	protein-codin	RPS13	ribosomal pr	O	40S ribosoma	20210404

	ENSG00000223799		6.99	-3.44	-3.04	0.002333677	0.042797561	2.67974414	1.32935583	3.13897486	0	0	0	0	0.62973961	36.8326396	41.4008625	0.69227046	0	5.04355756	7.691313603	4.6044534	7.866330294											
	ENSG00000278330		16.07	-2.21	-3.04	0.002342483	0.042876456	3.26051079	10.1435399	0	1.6164377	7.36463918	0.8156803	2.69631355	6.17468985	50.2201553	106.580969	0	2.39231724	22.8216525	22.73177108	6.87673754	13.49069883											
CASP4LP	ENSG00000235505	643733	112.05	0.81	3.04	0.002345093	0.042882979	77.4946372	81.6536486	338.629457	113.568098	241.683058	134.713885	64.5398466	112.687191	22.4167718	60.2413179	142.922972	90.5262989	110.687483	49.23180384	66.478194	85.32723291	CASP17P	HGNC:HGNC:	11	11q22.3	caspase 4 like	pseudo	CASP4LP	caspase 4 like	O	caspase 17, p	20210302
ZNF627	ENSG00000198551	199692	112.24	-0.84	-3.04	0.002368331	0.04322488	84.0101375	121.243286	22.8355815	61.0723236	94.291752	97.7017759	89.2826615	87.7241173	120.09255	155.516084	137.313306	169.949309	149.462128	119.5441627	163.386683	122.3753025	-	MIM:612248	19	19p13.2	zinc finger pr	protein-codir	ZNF627	zinc finger pr	O	zinc finger pr	20210302
DDX28	ENSG00000182810	55794	75.11	-1.19	-3.04	0.002379198	0.043381628	48.0627663	27.146572	15.2089343	35.5387925	55.7279388	64.8725333	54.6261549	63.6692506	141.316068	105.361487	141.492933	154.66936	80.4224168	63.41481643	61.985662	88.30761831	MDDX28	MIM:607618	16	16q22.1	DEAD-box he	protein-codir	DDX28	DEAD-box he	O	probable ATF	20210302
LSR	ENSG00000105699	51599	109.14	-1.31	-3.04	0.002387174	0.043443835	97.2630651	174.113572	12.4488459	81.1931856	5.4375957	119.075566	28.8689959	73.6481435	153.269803	187.753362	111.246919	103.7367	105.783175	197.2063121	178.645433	116.5194409	ILDR3|LISCH7	MIM:616582	19	19q13.12	lipolysis stim	protein-codir	LSR	lipolysis stim	O	lipolysis-stim	20210417
SLX4	ENSG00000188827	84464	146.54	0.69	3.04	0.002386831	0.043443835	110.995214	96.7256182	204.328017	132.759125	257.400372	259.685093	226.324343	167.99721	77.227439	120.541255	130.687305	76.8602291	185.211781	110.192784	91.5430091	96.19256887	BTBD12|FAN	MIM:613278	16	16p13.3	SLX4 structur	protein-codir	SLX4	SLX4 structur	O	structure-spe	20210410
ASIC1	ENSG00000110881	41	8.27	-3.35	-3.04	0.002390484	0.04346251	13.5746399	2.01184338	0	0	0	12.7089023	0	0.50559701	10.0995415	20.9977969	10.9037448	8.19654802	12.5057202	6.009815187	29.9243329	4.953828725	ACCN2|ASIC|	MIM:602866	12	12q13.12	acid sensing i	protein-codir	ASIC1	acid sensing i	O	acid-sensing i	20210302
PUS7	ENSG00000091127	54517	41.67	-1.34	-3.04	0.002401398	0.043564201	40.2634871	65.720174	24.4483229	8.00628904	4.93458558	13.8828104	21.351837	21.8958108	136.127274	50.4451863	43.3609305	71.3593457	15.0003498	66.70022118	30.4773465	52.73442651	IDDABS	MIM:616261	7	7q22.3	pseudouridin	protein-codir	PUS7	pseudouridin	O	pseudouridyl	20210302
RETREG1	ENSG00000154153	54463	213.22	-1.13	-3.04	0.002400138	0.043564201	337.454814	151.131753	216.984491	98.564954	37.8105285	202.671007	60.3725506	69.7794902	292.859612	220.213529	227.922639	445.258562	156.636645	226.01392	468.757624	199.0946887	FAM134B|JK	MIM:613114	5	5p15.1	reticulophagy	protein-codir	RETREG1	reticulophagy	O	reticulophagy	20210418
CNKSR2	ENSG00000149970	22866	101.75	-2.15	-3.03	0.002408703	0.04362708	239.567691	123.625419	0	17.4624617	0	237.410944	20.9189386	14.4341847	189.087979	77.511457	56.5210946	148.872383	109.740588	125.8581373	196.596529	70.42098341	CNK2|KSR2|I	MIM:300724	X	Xp22.12	connector en	protein-codir	CNKSR2	connector en	O	connector en	20210302
APRT	ENSG00000198931	353	102.75	-0.84	-3.03	0.00242561	0.043891548	97.8494966	98.5539769	13.3094539	107.591176	44.1520223	122.878401	82.3259803	51.8677037	150.250207	132.293991	102.03601	151.57038	87.1778561	173.2023787	111.917003	117.0919693	AMP|APRTD	MIM:102600	16	16q24.3	adenine phos	protein-codir	APRT	adenine phos	O	adenine phos	20210410
NOB1	ENSG00000141101	28987	159.30	-0.61	-3.03	0.002432191	0.043968833	125.124173	194.318424	83.5360925	90.4517479	125.712438	170.026195	125.993412	105.384446	156.591586	215.780935	145.98903	217.792576	155.461589	219.4070375	220.263553	196.9721351	ART-4|MST15	MIM:613586	16	16q22.1	NIN1 (RPN12	protein-codir	NOB1	NIN1 (RPN12	O	RNA-binding	20210302
KIF16B	ENSG00000089177	55614	117.65	-0.80	-3.03	0.002445036	0.044117254	104.737436	65.9275816	18.1812444	110.658052	146.02592	145.209084	80.0942839	41.5241466	229.758181	135.260117	61.8692197	128.227762	210.724188	217.3913897	70.9242068	115.9035596	C20orf23|KIS	MIM:618171	20	20p12.1	kinesin family	protein-codir	KIF16B	kinesin family	O	kinesin-like p	20210316
SHISA2	ENSG00000180730	387914	8.12	-4.06	-3.02	0.002495166	0.044894129	0	0	71.363734	0	0	0.88795958	0	0	4.4045707	3.78273604	7.24463267	15.5017685	4.31568918	5.919761469	11.8641108	4.58050453	C13orf13|PR	MIM:617324	13	13q12.13	shisa family r	protein-codir	SHISA2	shisa family r	O	protein shisa-	20210302
LTA4H	ENSG00000111144	4048	359.03	-0.62	-3.02	0.002499766	0.044934418	529.602084	476.614777	254.762993	195.188253	212.078233	213.674634	199.303368	256.812738	597.880612	426.854547	366.023053	556.345481	391.471242	366.0478555	368.8061	333.0666091	-	MIM:151570	12	12q23.1	leukotriene A	protein-codir	LTA4H	leukotriene A	O	leukotriene A	20210404
SPATA1	ENSG00000122432	100505741	10.83	-3.25	-3.02	0.002502455	0.044940315	1.61265596	0	0	5.04390235	0	16.7619139	0	0.96721571	52.9259355	56.2879661	0.65891206	8.50412028	10.8042744	8.360642082	4.48934785	6.802446031	SP-2|SPAP1	HGNC:HGNC:	1	1p22.3	spermatogen	protein-codir	SPATA1	spermatogen	O	spermatogen	20210302
COL11A2	ENSG00000204248	1302	8.79	-3.17	-3.02	0.002515272	0.045085417	6.51046861	2.78040366	0	0	2.78223262	0	0.33903156	2.59699041	49.7106959	3.02137783	15.4504419	18.3187486	6.955334	12.0204218	19.4977162	0.683453945	DFNA13|DFN	MIM:120290	6	6p21.32	collagen type	protein-codir	COL11A2	collagen type	O	collagen alph	20210410
OLA1	ENSG00000138430	29789	230.71	-0.67	-3.02	0.002523125	0.045098782	209.57085	196.341536	111.374823	231.765618	75.6309686	167.723262	291.209384	170.543376	366.215668	210.424571	209.734423	320.533214	222.326497	266.6016608	262.757742	378.5794194	DOC45|GBP4	MIM:611175	2	2q31.1	Obg like ATPa	protein-codir	OLA1	Obg like ATPa	O	obg-like ATPa	20210417
NDUFAF5	ENSG00000101247	79133	135.39	0.72	3.02	0.00252185	0.045098782	125.20064	118.916383	457.861889	205.71736	195.69307	91.0176864	106.18767	87.7445732	58.5036278	105.820848	189.789762	121.771855	76.4843219	104.5112415	70.8912008	50.16400448	C20orf7|MC1	MIM:612360	20	20p12.1	NADH:ubiqui	protein-codir	NDUFAF5	NADH:ubiqui	O	arginine-hydr	20210417
PDZD4	ENSG00000067840	57595	93.60	1.17	3.02	0.002529688	0.045173674	103.081101	29.4041511	48.2333278	118.208227	224.449394	126.744477	211.27206	193.371529	56.4556138	64.9416413	30.3763396	39.2979351	112.481841	31.26111454	34.0415207	74.05188589	LNX5|LU1|PI	MIM:300634	X	Xq28	PDZ domain c	protein-codir	PDZD4	PDZ domain c	O	PDZ domain-	20210302
STXBP1	ENSG00000136854	6812	81.35	-1.22	-3.02	0.002537589	0.045272299	74.3213996	125.274351	18.1702898	25.6821891	3.67156489	92.4482911	76.2068936	15.1686396	313.063767	91.7440327	44.3276916	28.8935724	34.0502798	154.886264	142.404698	61.3077292	DEE4|MUNC	MIM:602926	9	9q34.11	syntaxin bind	protein-codir	STXBP1	syntaxin bind	O	syntaxin-binc	20210418
	ENSG00000227262		3.77	-3.98	-3.02	0.002554402	0.045486988	0.51804004	2.6333827	0	0	0	0	0	0	25.7949335	6.12956103	3.61931096	3.07143156	1.76759313	8.935276116	4.19086835	3.633144835											
TBC1D31	ENSG00000156787	93594	461.30	0.58	3.02	0.002553756	0.045486988	434.227895	516.980981	824.190494	482.410565	556.004397	319.937057	743.882904	514.813122	178.068497	416.963903	381.381879	362.078811	530.37378	212.3938443	377.239086	529.8684236	Gm85|WDR6	HGNC:HGNC:	8	8q24.13	TBC1 domain	protein-codir	TBC1D31	TBC1 domain	O	TBC1 domain	20210302
SNORA49	ENSG00000208892	677829	254.48	-1.67	-3.02	0.002563364	0.045561342	1203.6116	634.919861	17.2102945	55.9411392	0	22.7367753	7.74261071	23.8350864	514.161488	544.963409	373.459222	531.949131	28.9146861	36.24542752	33.308281	42.63023745	ACA49	HGNC:HGNC:	12	12q24.33	small nucleol	snoRNA	SNORA49	small nucleol	O	ACA49 snoRN	20210302
USP35	ENSG00000118369	57558	72.04	-1.00	-3.01	0.002586759	0.045887745	22.2364315	54.7205396	28.4093225	25.4213281	31.3867584	28.9900009	63.9005856	140.708333	32.2010915	112.89944	53.3470252	36.2911534	162.221677	105.7007933	111.431276	142.8273708	-	HGNC:HGNC:	11	11q14.1	ubiquitin spe	protein-codir	USP35	ubiquitin spe	O	ubiquitin carl	20210302
TMTC3	ENSG00000139324	160418	319.82	0.63	3.01	0.002585641	0.045887745	297.49855	255.434218	714.335919	466.237183	492.387684	374.541843	274.711629	285.002826	128.867758	241.726265	246.377994	324.715163	265.308479	245.4542729	234.647383	269.7986009	LIS8|SMILE	MIM:617218	12	12q21.32	transmembra	protein-codir	TMTC3	transmembra	O	protein O-ma	20210302
CCDC191	ENSG00000163617	57577	87.58	0.80	3.01	0.00258896	0.045887745	78.8781288	36.7521549	133.800746	176.959698	109.491338	135.639279	151.115697	75.761825	77.9660959	39.5558112	92.6779839	79.6891805	63.0673856	58.78920686	61.4296604	29.72872761	KIAA1407	HGNC:HGNC:	3	3q13.31	coiled-coil do	protein-codir	CCDC191	coiled-coil do	O	coiled-coil do	20210302
	ENSG00000269984		14.21	-2.76	-3.01	0.002598384	0.046006618	5.51375028	5.7486313	0	0	0	0	0.65637091	2.4717086	78.5381828	117.729004	0	2.17029305	3.42004361	4.849430664	2.09454841	4.11492844											
ACSBG1	ENSG00000103740	23205	13.43	-3.40	-3.01	0.002621672	0.046253205	4.73819406	2.76156289	0	0	0	0	0	0	90.6256045	103.1843	0	2.54389303	0.60994173	1.477069167	8.13704378	0.840411729	BG|BG1|BGM	MIM:614362	15	15q25.1	acyl-CoA synt	protein-codir	ACSBG1	acyl-CoA synt	O	long-chain-fa	20210302
TNFRSF11A	ENSG00000141655	8792	15.53	-2.59	-3.01	0.002621728	0.046253205	0	14.5647343	0	0	0	20.3635982	0	0.84962127	0	32.3911533	2.58705324	2.40287465	17.3741706	152.8406993	1.54109861	3.549469663	CD265|FEO|I	MIM:603499	18	18q21.33	TNF receptor	protein-codir	TNFRSF11A	TNF receptor	O	tumor necros	20210418
ETFDH	ENSG00000171503	2110	347.87	0.60	3.01	0.002620616	0.046253205	426.818385	475.626171	454.217732	591.354202	407.959533	406.690624	225.016206	394.638998	312.067183	253.009271	282.634048	265.862875	266.886531	257.6420451	318.287343	227.2263493	ETFQO|MAD	MIM:231675	4	4q32.1	electron tran	protein-codir	ETFDH	electron tran	O	electron tran	20210410
HPCAL1	ENSG00000115756	3241	175.32	-0.95	-3.01	0.002640614	0.046543263	263.724358	187.216087	164.51387	90.1285041	73.8400935	147.298444	51.9771675	56.8827357	270.427366	210.645883	153.128904	201.150097	172.747609	272.4521065	273.791882	215.2677352	BDR1|HLP2|'	MIM:600207	2	2p25.1	hippocalcin li	protein-codir	HPCAL1	hippocalcin li	O	hippocalcin-li	20210417
FBXO41	ENSG00000163013	150726	90.37	-1.14	-3.01	0.002652092	0.046702343	44.1419958	70.7914905	7.15900949	47.902682	69.7392529	67.2354822	59.3566475	113.00212	98.5638496	127.851626	120.006294	96.7390312	173.129569	125.6803482	146.993118	77.55784964	FBX41	MIM:609108	2	2p13.2	F-box protein	protein-codir	FBXO41	F-box protein	O	F-box only pr	20210302
HSPA7	ENSG00000225217	3311	18.30	2.12	3.00	0.002659981	0.046754747	1.928231	12.4534279	0	8.27725473	58.6977781	21.667832	66.7839221	54.1386041	2.5607307	3.87176186	1.6758292	0.98921149	47.0967903	0.834369065	7.65452029	4.187299114	HSP70B	MIM:140556	1	1q23.3	heat shock pr	pseudo	HSPA7	heat shock pr	O	heat shock 7(	20210404
CSKMT	ENSG00000214756	751071	90.17	-1.68	-3.00	0.002666486	0.046825861	428.258693	109.41673	6.78961226	41.4096356	0.81479282	25.5612799	14.6632283	35.4337036	100.21649	160.895761	123.175022	109.532945	26.8128247	75.80107698	74.5734509	109.4196142	CS-KMT|MET	MIM:617897	11	11q12.3	citrate syntha	protein-codir	CSKMT	citrate syntha	O	citrate syntha	20210302
	ENSG00000288349		7.98	-4.08	-3.00	0.002669745	0.04682883	0	0	0	0	0	0	5.7332994	2.40139423	28.5909151	19.6592707	0	1.11297889	27.4767763	17.09864101	9.13159814	16.50227293											
PECAM1	ENSG00000261371	5175	438.63	-1.14	-3.00	0.002671575	0.04682883	799.306736	520.97137	280.698096	75.9339537	153.167087	481.72224	206.174305	92.3631382	549.339001	468.398697	481.082669	695.798256	431.736035	581.0422965	737.759438	462.5722401	CD31|CD31/I	MIM:173445	17	17q23.3	platelet and e	protein-codir	PECAM1	platelet and e	O	platelet endo	20210404
FOS	ENSG00000170345	2353	85.29	-2.21	-3.00	0.002677511	0.046889694	61.7549218	46.8178526	16.8755562	23.2464745	0	0	6.9449737	4.13695426	1006.89121	44.407458	11.9005686	6.7156454	34.2427039	5.948375369	60.7154812	34.01336521	AP-1|C-FOS|	MIM:164810	14	14q24.3	Fos proto-onc	protein-codir	FOS	Fos proto-onc	O	proto-oncoge	20210404
	ENSG00000254721		9.00	-3.82	-3.00	0.00268007	0.046891372	10.6746359	0	0	0	0	0	0	0.57884316	30.8691079	70.5847387	1.82627569	0	5.33932231	5.717190026	7.14315782	11.31611707											
	ENSG00000210156		9.67	3.57	3.00	0.002685852	0.046949387	43.0217202	20.4267382	11.255442	46.9980183	0	9.27074777	0.93788587	1.75084828	0	0	17.18739	1.59209948	0	2.32238259	0	0											
	ENSG00000284057		10.98	-8.99	-3.00	0.002699559	0.047102474	0	0	0	0	0	0	0	109.482897	0	0	16.0152414	0	0	27.27592483	22.8514906	0											
MRPS25	ENSG00000131368	64432	167.74	-0.86	-3.00	0.002697718	0.047102474	299.977358	62.8108352	66.6066804	69.2355937	98.3133909	65.7791165	89.0349345	79.1790337	859.900313	195.837576	70.8025155	69.6900626	141.299543	245.9200679	185.531088	83.87149171	COXPD50|MI	MIM:611987	3	3p25.1	mitochondria	protein-codir	MRPS25	mitochondria	O	28S ribosoma	20210302
BMAL1	ENSG00000133794	406	768.77	0.60	3.00	0.002702631	0.04711289	739.658293	630.487387	1310.38617	1475.25969	984.873684	738.056183	840.250372	684.272547	278.713235	704.552685	959.134977	573.707466	680.554597	611.2756882	530.733712	558.4395005											
NT5E	ENSG00000135318	4907	236.49	-1.53	-3.00	0.002714037	0.047268438	373.480367	340.879304	89.836747	6.08202047	8.52987872	334.643059	65.9532654	125.392972	460.906485	308.654917	143.312977	340.930153	113.287032	463.933536	341.55446	266.4457384	CALJA|CD73|	MIM:129190	6	6q14.3	5'-nucleotida	protein-codir	NT5E	5'-nucleotida	O	5'-nucleotida	20210418
TCEA3	ENSG00000204219	6920	107.29	-2.30	-3.00	0.002719211	0.047272052	137.94422	19.2310592	385.325569	34.5044212	0	25.6317723	5.00901226	0	753.056709	37.2732393	22.332821	69.2819799	32.3470917	103.7905713	48.9955635	41.8880507	TFIIS|TFIIS.H	MIM:604128	1	1p36.12	transcription	protein-codir	TCEA3	transcription	O	transcription	20210417
SPRY1	ENSG00000164056	10252	34.42	-1.68	-3.00	0.002725629	0.047340393	66.7784329	12.0355171	28.14513	14.7319676	0	25.0030832	2.86613301	10.4673233	122.581626	29.3441819	37.7211802	55.0608039	40.7808607	27.5109174	52.1611875	25.56809485	hSPRY1	MIM:602465	4	4q28.1	sprouty RTK s	protein-codir	SPRY1	sprouty RTK s	O	protein sprou	20210302
	ENSG00000263756		7.50	-3.04	-3.00	0.002740459	0.047511268	2.41569257	9.38890531	0	3.69588323	0	0	0	0.76179277	26.7149504	45.8728894	2.2094394	1.29266981	11.3880831	2.744300677	5.80078402	7.786986511											
RPS21	ENSG00000171858	6227	1801.59	-0.72	-2.99	0.002771372	0.047959855	1841.54232	2095.77098	1758.20763	1596.28831	424.228884	1223.54666	931.136121	1012.75089	1419.35343	1627.53361	3601.40791	4518.63127	1110.62895	1714.499049	1786.06289	2163.778592	HLDF|S21	MIM:180477	20	20q13.33	ribosomal pro	protein-codir	RPS21	ribosomal pro	O	40S ribosoma	20210307
	ENSG00000279159		84.57	-1.81	-2.99	0.002782919	0.04803602	57.004217	22.212815	9.66962799	11.3312192	17.6040297	7.2108134	12.049301	24.8737917	442.037785	529.96783	20.5751059	0.67158849	57.0098356	43.69188819	49.9386907	47.20218656											
SPIN1	ENSG00000106723	10927	622.43	-0.58	-2.99	0.002783344	0.04803602	492.397836	586.855186	316.551788	530.187038	562.017768	429.467803	527.559173	451.925896	1421.43288	828.091218	677.516863	804.228436	614.374758	637.8096401	522.683468	555.7948586	SPIN|TDRD24	MIM:609936	9	9q22.1	spindlin 1	protein-codir	SPIN1	spindlin 1	O	spindlin-1|ov	20210404
PDE9A	ENSG00000160191	5152	38.64	-2.43	-2.99	0.002790071	0.048096572	76.7762501	29.4308731	0	0	0	54.7966202	50.2512504	1.70521585	96.9194288	39.4991869	34.7894495	59.8080575	36.5361299	48.57551441	62.5981795	26.58663807	HSPDE9A2	MIM:602973	21	21q22.3	phosphodiest	protein-codir	PDE9A	phosphodiest	O	high affinity c	20210302
SANBR	ENSG00000162929	84542	92.04	1.08	2.99	0.002791905	0.048096572	142.272605	95.1853278	147.090841	39.4173209	133.841663	183.851542	111.59233	132.993679	107.276315	65.2775539	47.9675368	47.3141388	113.247343	27.1174582	41.5629781	36.60219822	KIAA1841	HGNC:HGNC:	2	2p15	SANT and BTI	protein-codir	SANBR	SANT and BTI	O	uncharacteriz	20210413
PLAG1	ENSG00000181690	5324	120.25	-1.85	-2.99	0.002811691	0.048393621	238.561987	131.919862	0	40.0734798	22.5074465	139.564746	16.9434707	23.9932655	95.3316558	114.851473	286.448651	221.000665	118.951244	215.719314	157.155606	100.9147077	PSA|SGPA|SI	MIM:603026	8	8q12.1	PLAG1 zinc fi	protein-codir	PLAG1	PLAG1 zinc fi	O	zinc finger pr	20210417
	ENSG00000244257		204.10	-1.58	-2.99	0.002814297	0.048394722	109.719127	108.28157	0	102.130957	173.53422	64.5682201	62.8752846	471.504814	161.234309	395.556439	191.677081	181.80986	428.977919	319.6628193	325.576265	168.4378581											
	ENSG00000271533		192.66	0.75	2.99	0.002823711	0.048512789	131.465373	99.2515033	301.46884	230.692823	518.112816	286.392244	185.666597	191.332788	54.9587841	88.852845	121.444103	183.839082	198.815245	177.2532201	230.569701	82.40690954											
TAMALIN	ENSG00000161835	160622	18.39	-2.64	-2.98	0.00283742	0.048704344	107.020053	25.2832875	0	0.47133865	0	6.34100983	0	2.19066803	42.2186464	28.8828951	9.66634985	12.4127906	7.18197234	16.50821328	18.6360253	17.42856578	GRASP	MIM:612027	12	12q13.13	trafficking reg	protein-codir	TAMALIN	trafficking reg	O	protein TAMA	20210302
KDM7A-DT	ENSG00000260231	100134229	78.67	-0.64	-2.98	0.002857339	0.048913942	49.0433454	90.2366422	58.5915757	70.8282947	51.6325339	66.167784	61.6413617	40.9663952	65.6113123	150.961311	87.4442682	103.596787	101.008009	74.25302862	120.667224	66.00562454	JHDM1D-AS1	HGNC:HGNC:	7	7q34	KDM7A diver	ncRNA	KDM7A-DT	KDM7A diver	O	JHDM1D anti	20210302
TTLL12	ENSG00000100304	23170	78.40	-0.94	-2.98	0.002898977	0.049537642	48.8253679	57.4272419	3.87302398	53.6962148	62.0975382	50.0194382	84.0664977	83.7087611	126.206084	79.3204421	57.4519604	72.8283234	131.903116	137.3705191	113.154248	92.51802563	dJ526I14.2	HGNC:HGNC:	22	22q13.2	tubulin tyrosi	protein-codir	TTLL12	tubulin tyrosi	O	tubulin--tyros	20210302
ACAT2	ENSG00000120437	39	102.00	-1.18	-2.98	0.002915942	0.049738247	136.977563	108.670996	0	69.4328684	40.1212447	44.8935911	44.3003784	85.2938591	358.987089	94.566642	86.130818	135.66227	68.7368597	124.0151809	81.7735023	152.3835422	-	MIM:100678	6	6q25.3	acetyl-CoA ac	protein-codir	ACAT2	acetyl-CoA ac	O	acetyl-CoA ac	20210302
ZNF830	ENSG00000198783	91603	246.63	0.67	2.98	0.002915943	0.049738247	398.943865	413.787899	615.76797	241.243293	189.429974	206.482924	191.327925	236.377395	145.472461	227.426005	222.581542	186.079212	156.8166	134.7178561	214.818295	164.8827023	CCDC16|OM	HGNC:HGNC:	17	17q12	zinc finger pr	protein-codir	ZNF830	zinc finger pr	O	zinc finger pr	20210302
TAPT1-AS1	ENSG00000263327	202020	25.89	-1.81	-2.98	0.002924338	0.049836798	17.7719332	17.2686993	0	0	33.9262577	8.88870771	10.1809736	24.0950881	39.4902345	59.0248395	22.1129788	26.1036224	55.886128	20.3705604	60.0471019	19.1125109	-	HGNC:HGNC:	4	4p15.32	TAPT1 antise	ncRNA	TAPT1-AS1	TAPT1 antise	O	-	20210302
	ENSG00000231799		6.63	-3.44	-2.98	0.002927935	0.049853454	2.28100468	16.8719312	0	0	2.25978074	0	0	0	14.8566327	29.7995385	3.02114515	1.35430538	12.4017515	11.54868671	5.47712883	6.186193899											
